# Derivatisation of parthenolide to address chemoresistant chronic lymphocytic leukaemia†
†Electronic supplementary information (ESI) available: General experimental details, aspects relating to cultivation of *feverfew*, the PTL extraction protocol, synthesis, biological assays computational information and spectrums are detailed in the supporting information. Methods described and references cited therein,^45^ should be referred to in regard of the docking, crystallographic, synthetic and toxicological studies detailed therein. See DOI: 10.1039/c9md00297a


**DOI:** 10.1039/c9md00297a

**Published:** 2019-08-01

**Authors:** Xingjian Li, Daniel T. Payne, Badarinath Ampolu, Nicholas Bland, Jane T. Brown, Mark J. Dutton, Catherine A. Fitton, Abigail Gulliver, Lee Hale, Daniel Hamza, Geraint Jones, Rebecca Lane, Andrew G. Leach, Louise Male, Elena G. Merisor, Michael J. Morton, Alex S. Quy, Ruth Roberts, Rosanna Scarll, Timothy Schulz-Utermoehl, Tatjana Stankovic, Brett Stevenson, John S. Fossey, Angelo Agathanggelou

**Affiliations:** a School of Chemistry, University of Birmingham, Edgbaston, Birmingham, West Midlands B15 2TT, UK. Email: j.s.fossey@bham.ac.uk; b Sygnature Discovery, The Discovery Building, BioCity, Pennyfoot Street, Nottingham, NG1 1GR, UK; c Institute for Cancer and Genomic Sciences, University of Birmingham, Edgbaston, Birmingham, West Midlands B15 2TT, UK. Email: a.agathanggelou@bham.ac.uk; d Winterbourne Botanic Garden, University of Birmingham, 58 Edgbaston Park Road, Edgbaston, Birmingham, West Midlands B15 2RT, UK; e School of Pharmacy and Biomolecular Sciences, Liverpool John Moores University, Byrom Street, Liverpool, L3 3AF, UK; f X-Ray Crystallography Facility, School of Chemistry, University of Birmingham, Edgbaston, Birmingham, West Midlands B15 2TT, UK; g ApconiX Ltd, Alderly Park, Nether Alderly, Cheshire, SK10 4TG, UK; h School of Biosciences, University of Birmingham, Edgbaston, Birmingham, West Midlands B15 2TT, UK

## Abstract

Parthenolide is a natural product that exhibits anti-leukaemic activity, however, its clinical use is limited by its poor bioavailability. It may be extracted from *feverfew* and protocols for growing, extracting and derivatising it are reported*.* A novel parthenolide derivative with good bioavailability and pharmacological properties was identified through a screening cascade based on *in vitro* anti-leukaemic activity and calculated “drug-likeness” properties, *in vitro* and *in vivo* pharmacokinetics studies and hERG liability testing. *In vitro* studies showed the most promising derivative to have comparable anti-leukaemic activity to DMAPT, a previously described parthenolide derivative. The newly identified compound was shown to have pro-oxidant activity and *in silico* molecular docking studies indicate a prodrug mode of action. A synthesis scheme is presented for the production of amine **7** used in the generation of **5f**.

## Abstract 2

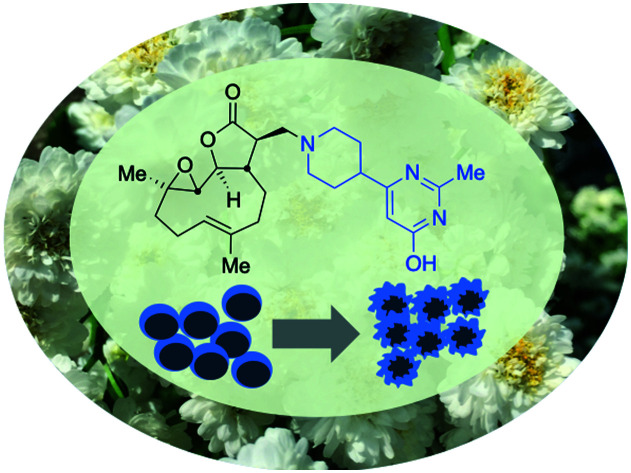
A parthenolide-derivative with favourable pharmacokinetic properties and good activity against drug-resistant chronic lymphocytic leukaemia is reported.

## Introduction

Chronic lymphocytic leukaemia (CLL) is the most common adult leukaemia with >3000 new cases in the UK annually.^1^ Its variable clinical course ranges from stable indolent disease that does not require treatment, to rapidly progressive disease that necessitates immediate therapeutic intervention.^2^ Clinical response rates to current therapies are strongly influenced by genetic changes including disruption of DNA damage response (DDR) genes ATM and p53.^3^ Tumours with a DDR defect are refractory to chemotherapeutics because they are unable to initiate apoptosis in response to therapy-induced DNA damage. This means tumours with or that develop this DDR defect are not treatable with typical DNA-damaging chemotherapies. Furthermore, since most CLL patients are over the age of 60 (median age >70) many have comorbidities that preclude the use of aggressive chemotherapeutic regimens. This highlights a need to develop alternative, less aggressive, therapies for the treatment of CLL.^4^

**Fig. 1:**
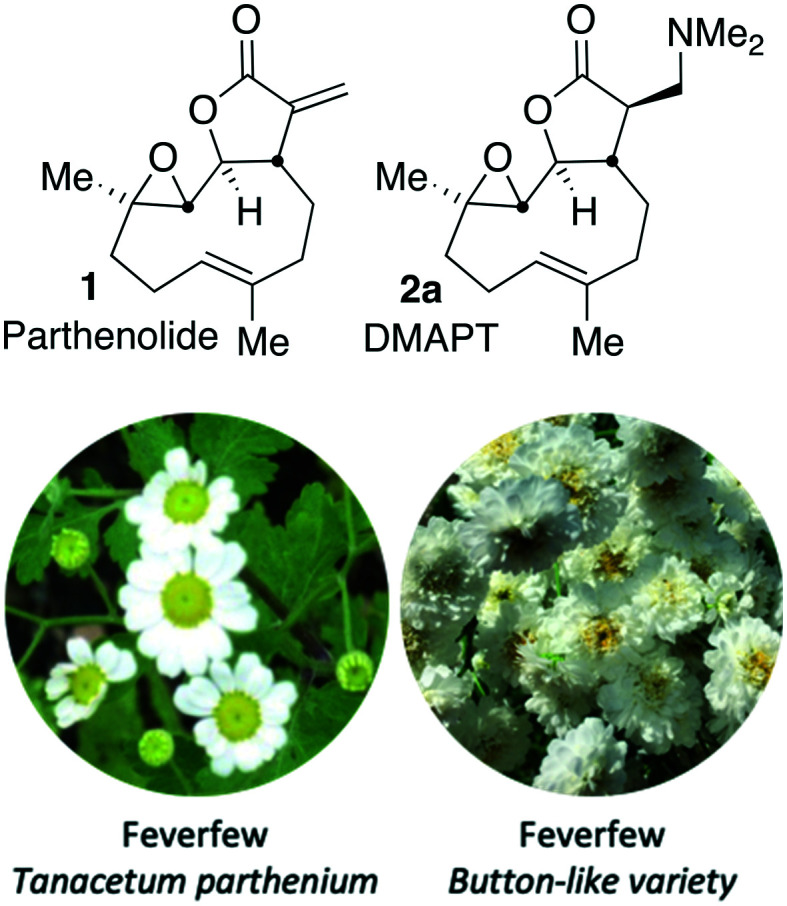
Upper: Structure of parthenolide (PTL, **1**) and dimethyl amino-PTL (DMAPT) (**2a**) respectively; lower flowers of feverfew and a button-like (*Tanacetum parthenium* ‘Flore Pleno’) feverfew varietal.

An attractive potential therapeutic is parthenolide (PTL, **1**), Fig. 1, upper. PTL (**1**) is a natural product isolated from *feverfew* (*Tanacetum parthenium*) and varietals thereof (Fig. 1, lower),^5^*feverfew* being so-named due to its use in traditional remedies.^6^ PTL (**1**) demonstrates effective and selective anti-CLL activity *in vitro*,^7^ and through its pro-oxidant activity, can target CLL cells independently of their p53 status.^8^ Furthermore, co-authors of this report^9^ are among those who have shown that ATM-deficiency disrupts redox homeostasis, increasing further the sensitivity of this DDR-defective subtype of CLL to PTL.^10^ In addition to inducing oxidative stress, PTL has been shown to target tumour cells by suppressing pro-survival and proliferation signalling through the NF-κB pathway,^11^ by inhibiting JAK–STAT kinase activity and thereby preventing STAT-mediated transcription of anti-apoptotic genes and by inhibiting DNMT1 and HDACs leading to activation of epigenetically silenced tumour suppressor genes.^12^ Furthermore, through inhibition of NF-κB, PTL is able to reverse resistance to a number of chemotherapeutics including paclitaxel, oxaliplatin, doxorubicin and metoxantrone.^13^ Despite numerous beneficial anti-tumour activities demonstrated *in vitro*, the clinical utility of PTL is limited by its poor bioavailability and pharmacokinetics.^14^

Crooks and co-workers pioneered the derivatisation of PTL and identified a promising candidate for the treatment of acute myeloid leukaemia (AML),^15^ a dimethyl amino-PTL (DMAPT) derivative, compound **2a** depicted in Fig. 1.^14^,^16^ This derivative of PTL (**1**), DMAPT (**2a**) and related compounds, have found utility across different diseases with activities against a range of cancer-types reported.16*u* PTL has been shown to induce oxidative stress in cancer cells,^17^ and recently DMAPT (**2a**) was confirmed to induce oxidative stress in CLL cells.^9^ There are a number of reports on the use and derivatisation of PTL, with a view to addressing various cancer types,16z,^18^ but the only PTL derivative to be applied to CLL remains compound **2a**.^19^ Previous studies have used molecular docking to investigate **1** and **2a** which have suggested that interaction with IKKβ is a plausible mechanism for their action against haematological and solid tumours.18*g*

It is reasoned that retro-Michael-type chemistry, that reveals PTL at the site of interest, may be at the origin of the activity of amino-PTL derivatives, meaning amino-PTL derivatives (such as **2a**) serve as excellent prodrugs for PTL delivery.16ac,ad Our interest in drug-like nitrogen-containing motifs^20^ prompted us to investigate analogues of DMAPT (**2a**) in the search for increased activity, favourable pharmacokinetic properties and minimal toxicological burdens, with the ambition of addressing drug-resistant CLL in elderly patients who may suffer more intensely from adverse effects of other therapies. Herein a synthesis cascade to explore the SAR of PTL derivatives is described and findings pertaining to these aims presented.

## Results and discussion

### Source of Parthenolide

**Fig. 2:**
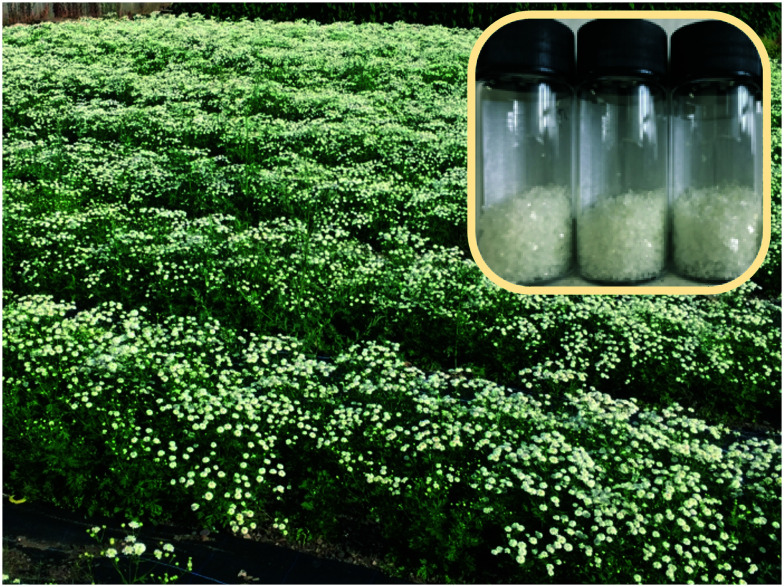
Feverfew cultivation effort, Winterbourne Botanic Garden, prior to harvesting feverfew; (inset) recrystallised PTL **1**.

Since 2013 *feverfew* and varietals have been cultivated at *Winterbourne House and Garden*,^21^ a UK visitor attraction, museum and heritage centre adjunct to the University of Birmingham campus (UK), located 52.4527° N. Annual crops of plant material have been obtained (Fig. 2), and PTL extracted. Plants in the late stage of flowering were found to contain the highest extractable content of PTL.

An extraction procedure previously reported by co-authors of this report was used (detailed in ESI†)^9^ to access recrystalised, analytically pure, PTL (**1**), (see ESI†).

### Derivatisation of parthenolide

The addition of nucleophilic primary and secondary amines to PTL has been reported to proceed with high diastereoselectivity *via* a conjugate addition to the exocyclic *Michael acceptor* double bond part of the lactone unit.^14^,16a,18f,^22^ The high selectivity for reaction of nucleophilic amines at the α,β-unsaturated lactone provides evidence of the compatibility of the epoxide motif contained within PTL and its derivatives thus obtained within a drug discovery programme. That is, epoxides display unwanted reactivity towards nucleophiles, and when this reactivity is unleashed *in vivo* off-target effects can render them undesirable motifs in medicinal chemistry.^23^ In related research, Long *et al.* reported the semi-synthesis of PTL and its cyclopropyl (in place of the epoxide part) analogue,^24^ the epoxide-free analogue was more stable to acid-mediated degradation, showing there may be scope to improve the drug-like properties of PTL analogues by further scaffold manipulation (although that was not probed herein).

**Scheme 1:**
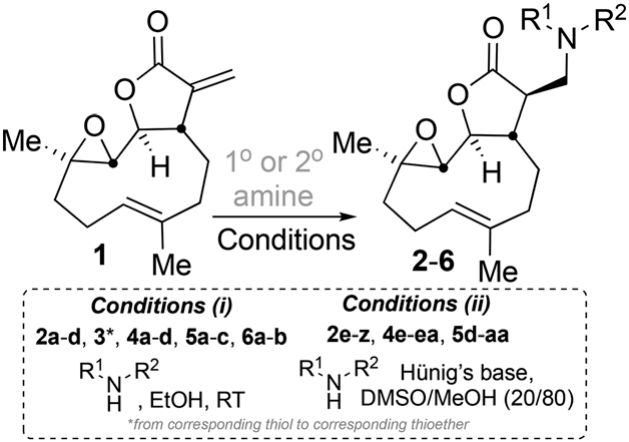
Synthesis of PTL derivatives **2** (tertiary amines), **3** (a thioether), **4** (acyclic secondary amines), **5** (cyclic tertiary amines) and **6** (amino acid derivatives). Two protocols: Conditions (i) – Addition of nucleophile in ethanol at room temperature; conditions (ii) – addition of nucleophile in dimethylsulphoxide/methanol (20/80) with Hünig's base at room temperature. All product R^1^ and R^2^ groups shown in Fig. 3.

**Fig. 3:**
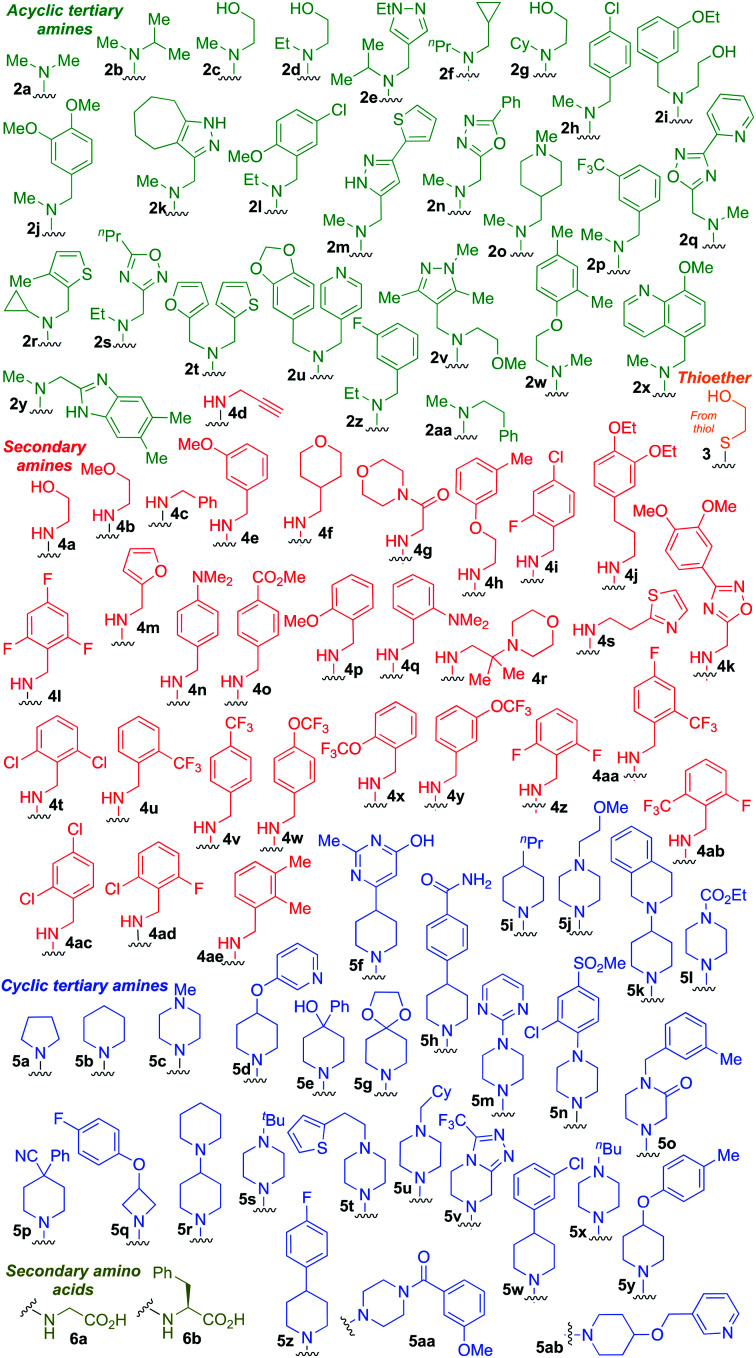
Summary of PTL derivatives' amine (and one thioether) parts, prepared according to Scheme 1.

Taking advantage of the ease of derivatisation of the lactone part of parthenolide a small initial library of amino (and related) PTL derivatives was generated using an established synthetic protocol (Scheme 1 – *conditions i* and Fig. 3), resulting in the isolation of compounds **2a–d**, **3**, **4a–d**, **5a–c** and **6a** & **b**. Following the successful synthesis of this small collection of PTL derivatives (Scheme 1 - *conditions i*), and in order to rapidly expand the library of amino-PTL derivatives available, a more high-throughput approach that allows for a library of products to be created with a range of functionalities and desirable drug-like properties was sought.^25^

Under slightly modified conditions from those previously employed,^9^,^26^ PTL was reacted with 120 primary or secondary amines that were selected based on calculated properties of the potential products. Used as supplied or obtained (whether neutral or as salts thereof) the amines reacted with PTL **1** in ethanol (Scheme 1 – *conditions i*) or in DMSO (20%) /methanol (80%) in the presence of Hünig's base (Scheme 1 – *conditions ii*).

The reaction mixtures were filtered and purified by reverse-phase HPLC of the 120 attempted synthesise, 76 successfully delivered products of sufficient purity (ideally >85% purity; 65 of which were obtained in >95% purity), as judged by HPLC or proton NMR spectroscopy). The materials obtained (shown in Fig. 3) were thus deemed suitable to progress to *in vitro* activity screening for anti CLL activity. Of the 76 compounds successfully synthesised and progressed to *in vitro* screening eleven have been previously reported elsewhere in the peer reviewed or patent literature, namely: **2a**,16*af***2b**,^14^**2c**,^14^,16a **3**,16a,w,^27^ **4a**,^14^**4c**,16*a***4d**,^28^**5a**,^14^,16w,^27^–^29^ **5b**,^14^,^27^–^29^ **5c**27b,29b and **6a**.16*y*

### Anti-chronic lymphocytic leukaemia activity

Defective p53-signalling increases genomic instability and drives tumour progression. A high frequency of p53 inactivation is found in relapsed, treatment-resistant, high risk CLL.^30^ The MEC1 cell line (obtained from the American type culture collection (Manassas, VA 20110 USA) expresses a mutated form of p53 and as previously shown, is resistant to clinically relevant DNA-damaging agents such as cyclophosphamide and is therefore representative of treatment-refractory, progressive CLL for which there is an urgent unmet clinical need.^31^ The *in vitro* antileukaemic activity of the PTL-derivatives against the MEC1 CLL cell line was determined using the alamarBlue® (ThermoFischer Scientific) assay and the results are summarised in Table S2.† 32 Among the active compounds **2c**, **2d**, **2e**, **2f**, **2g**, **2j**, **2aa**, **5a**, **5b**, **5d**, **5e**, **5f**, **5h**, **5ab** and **6b** (Table S2,† entries 4 to 8, 11, 12, 29, 62, 63, 65 to 67, 69, 89 and 91 respectively) gave EC_50_ values <15 μM. Owing to resource availability a subset of up to ten of these compounds was prioritised for further investigation. Compounds **2d**, **2e**, **2j**, **2aa**, **5a**, **5d**, **5e**, **5f**, **5ab** and **6b** were selected on the basis of reasons including ease of synthetic access, diversity across the chemical space probed and novelty. Those prepared under *conditions (i)*Scheme 1 (**2d**, **5a** and **6b**) were available on a scale and in a purity to directly permit follow-up studies, whereas compounds prepared under *conditions (ii)*Scheme 1 were prepared on a small scale so were resynthesised. These seven compounds (**2e**, **2j**, **2aa**, **5d**, **5e**, **5f** and **5ab**) were isolated on >30 mg scale in high purity and progressed to more following studies.

### *In vitro* DMPK

Compounds **2d**, **2e**, **2f**, **2g**, **2j**, **2aa**, **5a**, **5d**, **5e**, **5f**, **5ab** and **6b** were subjected to *in vitro* DMPK testing. Seven of the ten PTL derivatives (**2e**, **5a**, **5b**, **5d**, **5f**, **5ab** and **6b**) exhibited greater than two hours stability in aqueous solution and were retained for further investigation. The other three (**2d**, **2j** and **2aa**) were not retained for further investigation due to poor stability (*T*_1/2_ at pH 7.4: 13, 107 and 76 min, Table 1, entries 1, 3 and 4 respectively). One obvious aspect contributing to observed stability is the propensity for PTL derivatives of this type to undergo a retro-amination (reverse-Michael-type mechanism), indeed this reverse reaction is often claimed as a pro-drug mode of action.29a,^33^ Whether poor stability in this test is as a result of retro-amination or any other process was not determined, either way the compound of interest does not survive long enough in solution to be retained for further study. It is a reasonable assertion that activity observed for these unstable compounds could well be ascribed to free PTL generated rapidly in the assay.

**Table 1:** Screening cascade for favourable drug-like properties of the ten most promising compounds ([—] indicates not tested)

Entry	Compound	EC_50_ ave./μM	LipE pEC_50_–clog *P*	Stability pH 7.4 *T*_1/2_/min	Microsomal stability (mouse)		Hepatocyte stability (mouse)		Caco-2 pH 7.4 mean Papp (10^–6^ cm s^–1^)		
					A to B	A to B	CL_int_/μL min^–1^ per million cells	*T*_1/2_/min	A to B	B to A	Efflux ratio
1	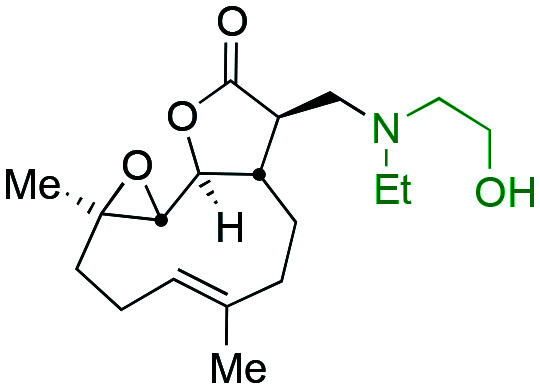	4.7	3.31	13	—	—	—	—	—	—	—
2	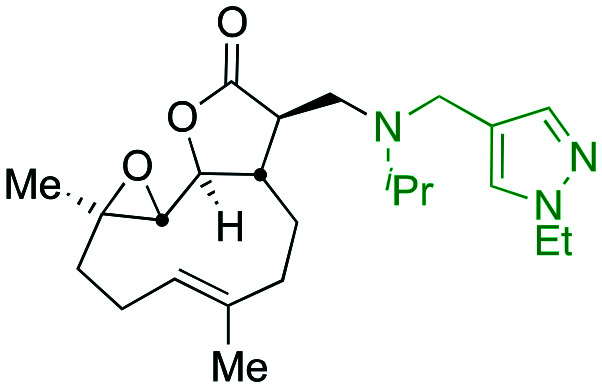	5.6	1.61	>120	828	2	—	—	—	—	—
3	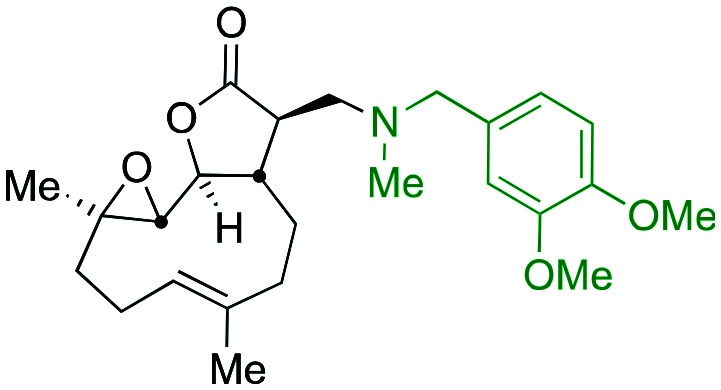	5.6	1.48	107	—	—	—	—	—	—	—
4	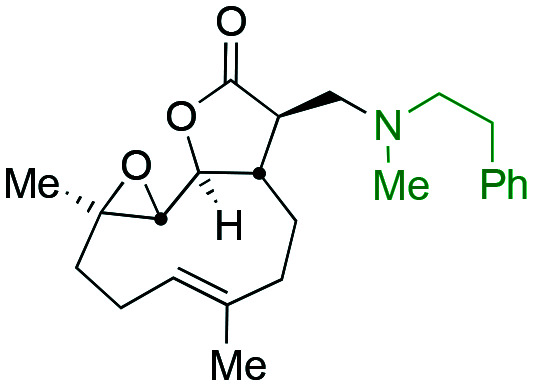	5.9	2.52	76	—	—	—	—	—	—	—
5	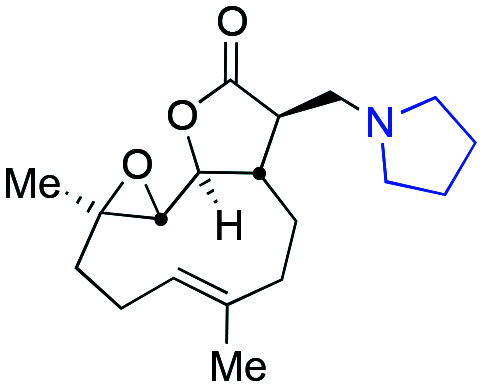	7.7	2.35	>120	19	75	22.0	65.0	15.9	16.2	1.0
6	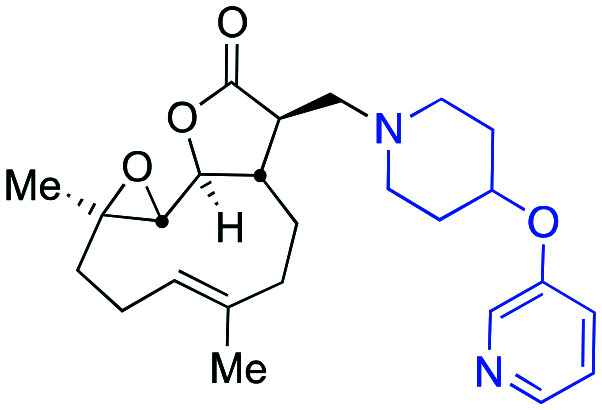	5.4	2.48	>120	251	6	—	—	—	—	—
7	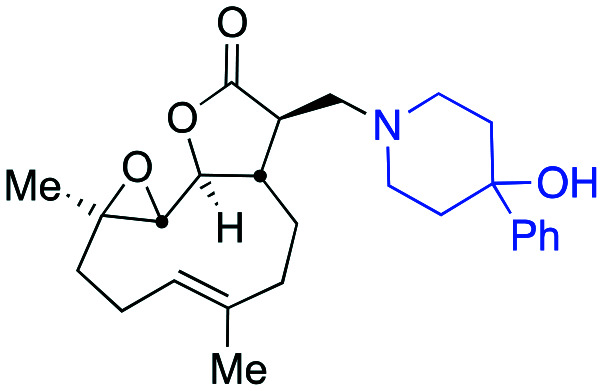	6.0	1.90	>120	391	4	—	—	—	—	—
8	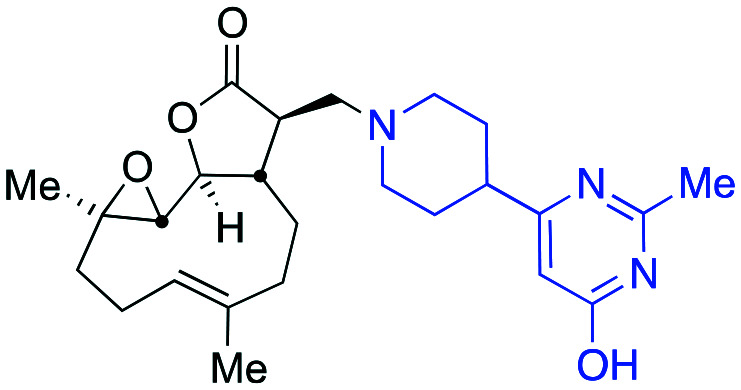	7.7	3.69	>120	18	77	22.0	64.0	2.7	2.8	1.1
9	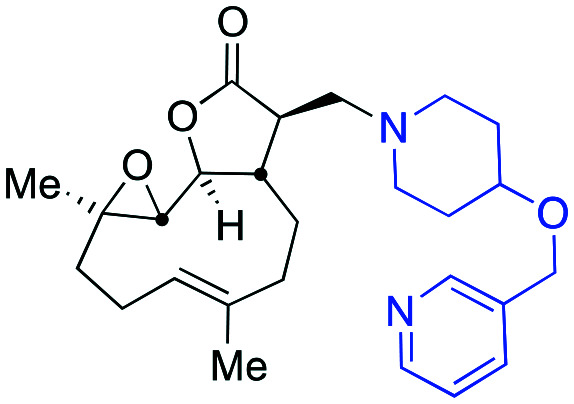	7.6	2.29	>120	128	11	—	—	10.9	9.1	0.8
10	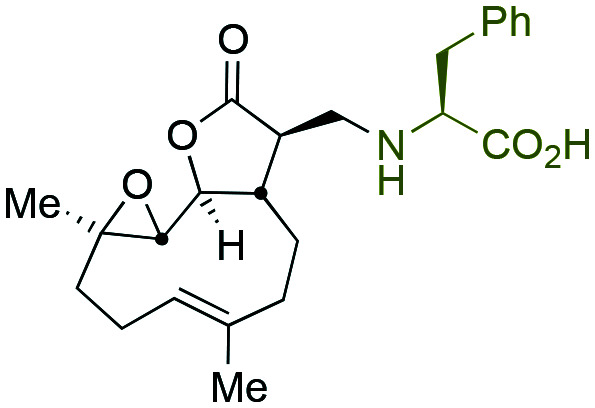	13.5	3.66	>120	9	160	16.0	88.0	1.5	4.2	2.7

Microsomal and hepatocyte stability assays are *in vitro* ADME assays used to determine metabolic stability of compounds through measuring intrinsic clearance (CL_int_) by liver microsomes or by liver hepatocytes. Compounds **2e**, **5d**, **5e** and **5ab** (Table 1, entries 2, 6, 7 and 9) exhibited CL_int_ of >100 μL min^–1^ mg^–1^ in the microsomal stability assay indicating rapid clearance and as such, were removed from further investigation. Compounds **5a**, **5f** and **6b** displayed the lowest microsomal intrinsic clearances, 19 μL, 18 μL and 9 μL min^–1^ mg^–1^, respectively (Table 1, entries 5, 8 and 10) and were therefore retained for further study.^34^ As a secondary screen for metabolic stability, compounds **5a**, **5f** and **6b** were subjected to the hepatocyte stability assay and this confirmed their suitability for progression to the Caco-2 permeability assay (Table 1, entries 5, 8 and 10). In order to determine the likelihood of the retained compounds being suitable for oral dosing a Caco-2 permeability assay, which predicts intestinal permeability and drug efflux, was conducted.^35^ Compounds **5a** and **5f** (Table 1, entries 5 and 8) gave efflux ratios close to unity (1.0 and 1.1 respectively), whereas compound **6b** (Table 1, entry 10) gave an efflux ratio of 2.7. Thus, compounds **5a** and **5f** were retained and selected for *in vivo* pharmacokinetic studies.

The parameter lipophilic ligand efficiency (LipE) allows activity of a compound in a given assay to be tensioned against the lipophilicity of the compound. The LipE parameter has become increasingly useful and important in medicinal chemistry drug discovery decision making.^36^ LipE allows the quality of hits to be compared, *i.e.* deconvolution of activity arising due to *better chemistry* away from increased activity due to enhancement in lipophilicity alone. It has been claimed that consideration of LipE alongside other chemoinformatic parameters in a drug discovery programme can result in the identification of compounds with superior *in vivo* properties.^37^ As such the calculated log *P* values (see Table S2†) were in determination of the LipE for the active compounds detailed in Table 1. Notably, the two compounds identified as superior through the conducted screening cascade of this manuscript (**5a** and **5f**) have the same 7.7 μM EC_50_ (pEC_50_ 5.11) values against the MEC1 cell line, whereas the calculated log *P* values differ by more than one log unit 2.76 *versus* 1.42 (respectively). Thus, the corresponding LipE values of 2.35 *versus* 3.69 for **5a** and **5f** respectively in the probed MEC1 cell line assay (Table 1, entries 5 and 8) reveal that whilst compound **5f** has an equivalent activity to **5a**, its activity may arise from advantageous chemical features and not from lipophilicity alone.^38^ Coupled with the data presented in Table S2,† consideration of the LipE indicates compound **5f** to offer a potential advantage in a drug discovery programme.

### *In vivo* pharmacokinetics

**Fig. 4:**
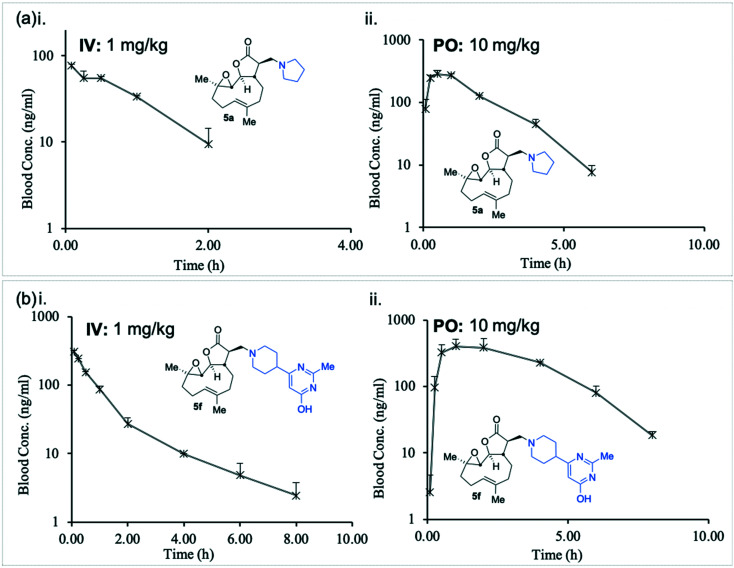
Graphical representation of murine *in vivo* pharmacokinetic study, concentrations of **5a** (a) and **5f** (b) i. IV (1 mg kg^–1^); ii. PO (10 mg kg^–1^). Data summarised in Table 2.

In order to determine **5a** and **5f** are suitable for use as pharmaceutical agents their pharmacokinetic (PK) parameters were next probed. A murine intravenous PK study of compounds **5a** and **5f** at 1.0 mg kg^–1^ was conducted and their concentrations in blood were measured with time (Fig. 4(a)i and (b)i and Table 2).

**Table 2:** Data obtained from murine *in vivo* pharmacokinetic study, concentrations of **5a** and **5f** (IV (1 mg kg^–1^) and PO (10 mg kg^–1^)). Data represented graphically in Fig. 4

Entry	Compound	EC_50_ ave./μM	Mouse PK (IV)				Mouse PK (PO)			
			Cl/mL min^–1^ kg^–1^	*T*_1/2_/h	VSS/L kg^–1^	UC (0-inf)/ng h mL^–1^	*T*_max_/h	*C*_max_/ng mL^–1^	F%	UC (0-inf)/ng h mL^–1^
1	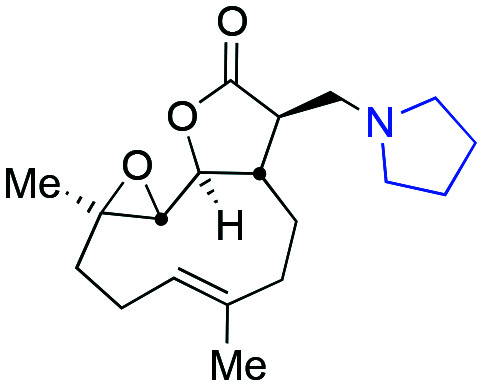	7.7	207	0.66	11.6	80.5	0.5	283	95.8	711
2	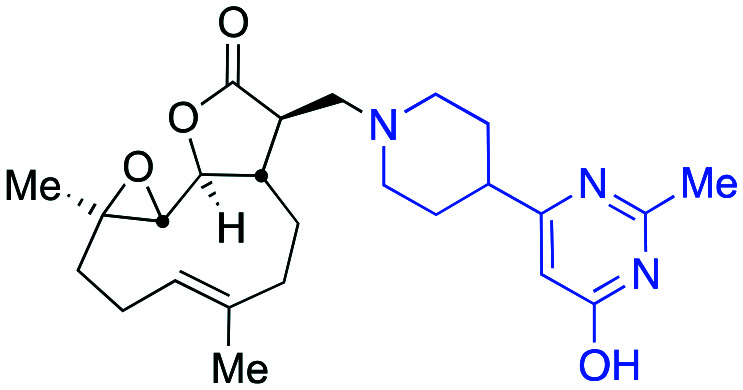	7.7	58	1.14	4.62	289	1	404	58.5	1696

Unlike the similarity between *in vitro* intrinsic clearance between **5a** and **5f** (Table 1), the *in vivo* clearance of **5a** was nearly four times that of **5f** (207 *versus* 58 mL min^–1^ kg^–1^, Table 2). The plasma half-life (*T*_1/2_) of **5f** was approximately twice that of **5a** (1.14 *versus* 0.66 hours, Table 2), and the total body exposure to **5f** was more than three times greater than **5a** (289 *versus* 80.5 ng h mL^–1^, Table 2).

The *in vivo* oral bioavailability of compounds **5a** and **5f** was compared in a murine PK study at 10 mg kg^–1^ (Fig. 4(a)ii and (b)ii and Table 2). In keeping with the *intravenous* findings, the murine PO half-life of **5f** was twice as long as **5a** (1.0 *versus* 0.5 hours, Table 2) and the *C*_max_ attained by **5f** was approximately 40% higher than that achieved by **5a** (404 *versus* 283 ng mL^–1^). The total body exposure was over two times greater for **5f** than **5a** (1696 *versus* 711 ng h mL^–1^, Table 2), indicating superior bioavailability. Compound **5f** was therefore identified as a promising potential new agent with desirable pharmacological properties towards a therapy for drug-resistant CLL.

### Comparison of the activity of **5f** with **1** (PTL) and **2a** (DMAPT)

**Fig. 5:**
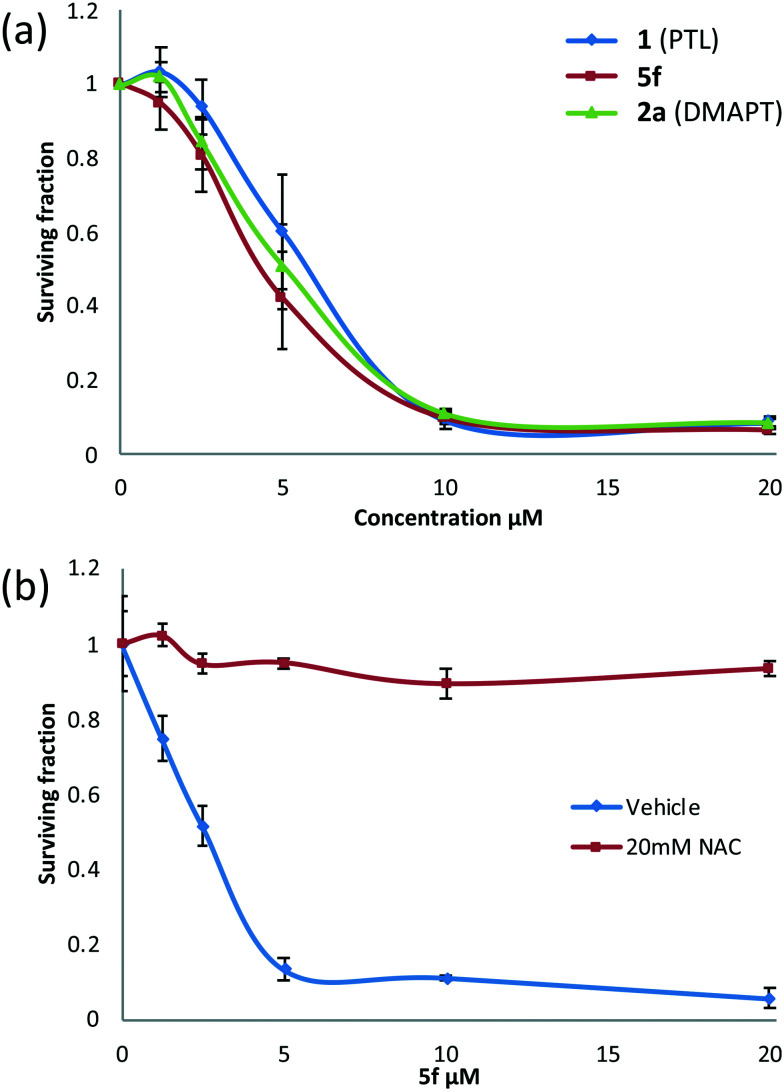
Concentration *versus* activity plots (alamarBlue® assay) showing (a) anti-leukaemic activity of **5f***versus* PTL and DMAPT in MEC1 cells and (b) the effect of NAC pretreatment on the activity of **5f**.

Following the identification of **5f** as the most promising drug-like derivative of PTL from the aforementioned parallel/high-throughput screening, the anti-leukaemic activity of **5f** was tested once more and compared directly with that of PTL (**1**) and DMAPT (**2a**), which serve as comparators and positive controls. Fig. 5(a) shows that the activity of **5f** (*n* = 5) (EC_50_ = 4.5 μM) was not significantly different to the activity of PTL (*n* = 5) or DMAPT (*n* = 5) with EC_50_ values of 6.2 μM and 5.6 μM, respectively (Table S2,† entries 1 and 2) indicating that it has comparable activity with the parent and literature compounds (**1** and **2a** respectively). Pre-treatment with *N*-acetylcysteine (NAC) abrogated activity demonstrating that similar to PTL and other derivatives, **5f** is a pro-oxidant and induces cell death through induction of oxidative stress, Fig. 5(b). Furthermore, *in silico* modelling suggests that **5f** is likely to function as a pro-drug and deliver PTL to also inhibit IKKβ and NF-κB, Fig. S3 and S4.†

### *In vitro* hERG liability testing

Early toxicological screening is an important step in derisking drug discovery programmes and is increasingly accessible.^39^ Since an unacceptable human ether a go-go related gene (hERG) liability is an all too common reason for failure at the candidate selection stage, a hERG assay was conducted to ensure the compounds being developed did not carry this liability.^40^

Three PTL derivatives (**2a**, **5a** and **5f**) were tested for inhibition of the hERG K^+^ channel (against cisapride as a positive control) using *IonWorks* patch clamp electrophysiology. In assessment of any hERG liability, compound **5a** was compared to compound **5f** which had already been identified as a superior pre-candidate compound; whilst **2a** (DMAPT) is specifically not the focus of this manuscript it was deemed appropriate to contrast any hERG liability of this the literature-reported anti-AML compound.16*af* Eight-point concentration-response curves were generated using three-fold serial dilutions from a maximum final test concentration of 100 μM, results are summarised in Fig. S6.†

The three PTL derivatives compared (**2a**, **5a** and **5f**) in this hERG liability assay all performed well displaying IC_50_ values >100 μM (*i.e.* inhibition of less than 50% at the top 100 μM test concentration). At said top (100 μM) test concentration the novel compound **5f** elicited only a 24% inhibition, whereas compounds **2a** and **5a** performed slightly less well with inhibitions of 40 and 45% (Fig. S6 and Table S4,† entries 1 and 2) respectively. Thus, hERG liability study, confirms PTL derivatives tested to represent a minimal risk according to results of the *in vitro* tests conducted. Among the compounds tested **5f** displayed the lowest hERG liability and was flagged as inactive (Table S4,† entry 3) confirming compound **5f** as the most promising lead from the studies conducted herein.

### Synthetic access to compound **5f**

**Scheme 2:**
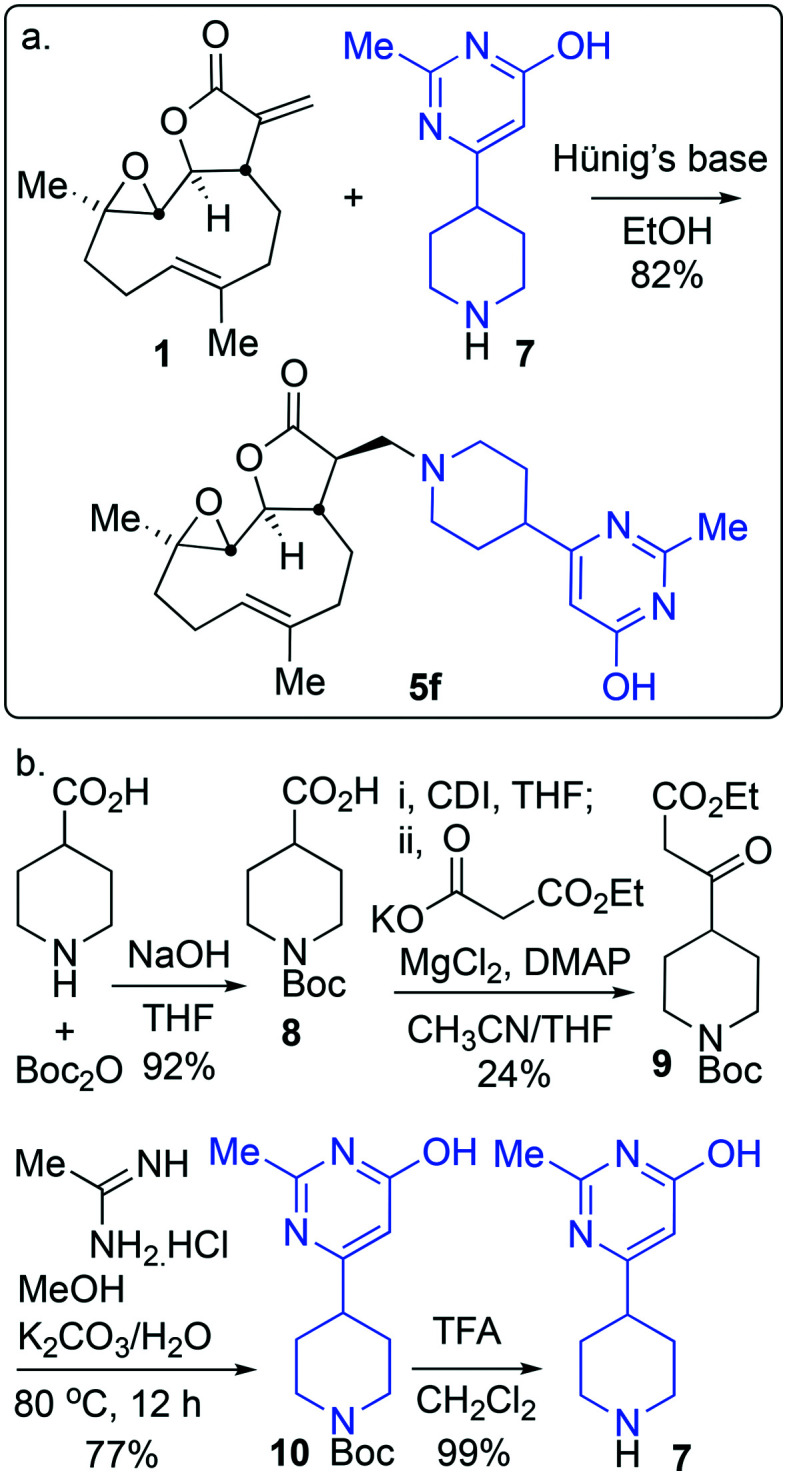
(a) The optimised synthesis of **5f**; (b) the designed and executed plan for the synthesis of the required amine **7**.

Through identification of compound **5f** as the resulting pre-candidate from the described screening cascade in earlier sections the requirement to provide more material for this and onwards studies presented a new and unexpected problem. Whilst it was possible to synthesise compound **5f** in 83% isolated yield in high purity *via* a hybrid of the earlier mentioned protocols (Scheme 2a), the availability of amine **7** caused some issues, namely during the course of this work resupply of amine **7** (or salts thereof) became challenging. This uncertain supply chain led us to probe the synthesis and set about synthesising our own material. Despite the compound being listed in the catalogues of numerous suppliers, a literature search (*SciFinder*) revealed very few instances of the motif occurring in the peer reviewed^41^ or patent literature.^42^ The synthesis of amine **7** had not, to the best of our knowledge at the time of writing this report, been reported in the peer reviewed literature. As such a synthetic protocol was proposed (Scheme 2b).

**Fig. 6:**
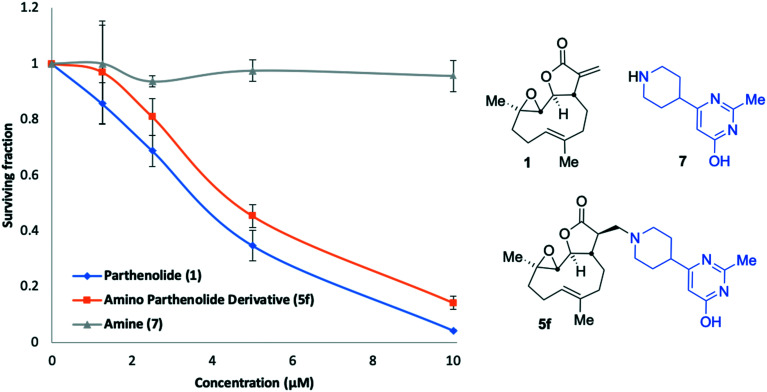
Concentration *versus* activity plots (alamarBlue® assay) showing anti-leukaemic activity of **1** (PTL), **5f** and amine **7** in MEC1 cells.

Amine **7** was successfully prepared as follows: 4-Piperidinecarboxylic acid was *N*-Boc protected to deliver **8** in 92% yield.^43^ In order to confidently assign the NMR spectra of compound **8** J-MOD, HSQC and ^1^H–^1^H COSY were helpful, particularly in establishing that a broad resonance in the proton-decoupled ^13^C NMR spectrum, centred on 42.8 ppm, corresponds to the ring-carbons bonded to nitrogen. The carboxylic acid of **8** was converted to the corresponding ethyl-3-keto-propanoate **9** in 24% yield. Conversion of **9** to the *N*-Boc protected congener **10**, was achieved by treatment with acetamidine hydrochloride and potassium carbonate, furnishing **10** in 77% yield. Treatment with trifluoroacetic acid delivered desired compound **7** in 99% yield for the Boc-deprotection step (scale 200 mg, 17% over four linear steps). Thus, evidencing accessibility of amine **7** and with it in hand, it was timely to probe any activity arising from the amine alone. MEC1 sensitivity to **1**, **5f** and **7** was compared in parallel using the alamarBlue® assay (Fig. 6). EC_50_ values were determined (*CalcuSyn* Version 2.11, *BIOSOFT*) for **1** (3.6 μM ± 0.46) and **5f** (4.3 μM ± 0.60). In contrast, an EC_50_ could not be determined for amine **7** since MEC1 cells were unaffected by equivalent concentrations of it indicating that **7** is not a source of cytotoxicity observed in this assay. A pre-peer-reviewed preprint of this article was deposited and may be viewed elsewhere.^44^

## Conclusions

The cultivation of *feverfew*, from which PTL **1** was extracted, was described. The isolation of PTL (**1**) permitted the synthesis of a library of derivatives, through smooth 1,4-addition to PTL's exocyclic Michael-acceptor C=C double bond. The library thus synthesised displayed good coverage of medicinally relevant chemical space. The compounds synthesised were tested for activity against the MEC1 CLL cell line. These compounds showed no particular trend in activity across the window of already acceptable medicinal chemistry parameters, such as p*K*_a_, cLog *D*, cLog *P*, TPSA, Fsp^3^ and molecular weight. The DMPK screening cascade described identified compound **5f** as the most promising from activity, safety profile and ADME property stand-points. Direct *in vitro* comparison showed **5f** exhibited comparable activity to DMAPT against the MEC1 cell line and pre-treatment with NAC suggested a pro-oxidant mechanism of action. Molecular docking studies with components of the NF-κB pathway also support a pro-drug mode of action of the compounds involving release of **1** and covalent interaction with one or more proteins involved in that pathway. This is partially supported by the lack of activity of the thioether adduct, compound **3**, that is far less prone to undergo a reverse reaction to reveal **1**, although lack of activity cannot be ascribed to this phenomenon alone. Taken together, these findings confirm **5f** as a promising motif for further study and elaboration towards treatments for drug resistant CLL.

## Notes

Ruth Roberts and Michael J. Morton are co-founders and co-directors of Apconix Ltd, an integrated toxicology and ion channel company that provides expert advice on nonclinical aspects of drug discovery and drug development to academia, industry and non-for-profit organisations. The remaining authors declare no competing interests.

## Author Contributions

All authors contributed critically to devising and executing this research. A detailed description of the specific contribution of each author is given in the ESI.†

## Conflicts of interest

There is no conflict of interest to declare.

## Supplementary Material

Supplementary information
