# Supplementary material for: Derivatisation of parthenolide to address chemoresistant chronic lymphocytic leukaemia
Source: Medchemcomm. 2019 Aug 1;10(8):1379–90. doi: 10.1039/c9md00297a (PMC7478165; doi:10.1039/c9md00297a)
Supplement: Supplementary file 1 [file MD-010-C9MD00297A-s001.pdf]

Electronic Supplementary Material (ESI) for MedChemComm.
This journal is © The Royal Society of Chemistry 2019

Supplementary Material

Derivatisation of parthenolide to address chemoresistant chronic lymphocytic leukaemia

Xingjian Li,[a†] Daniel T. Payne,[a†] Badarinath Ampolu,[b,c] Nicholas Bland,[b,d] Jane T. Brown,[b] Mark J. Dutton,[a] Catherine A. Fitton,[e] Abigail Gulliver,[f] Lee Hale,[f] Daniel Hamza,[b] Geraint Jones,[b] Rebecca Lane,[b] Andrew G. Leach,[g] Louise Male,[h] Elena G. Merisor,[b] Michael J. Morton,[i] Alex S. Quy,[a] Ruth Roberts,[i,j] Rosanna Scarll,[e] Timothy Schulz-Utermoehl,[b] Tatjana Stankovic,[e] Brett Stevenson,[b] John S. Fossey,[a†]* Angelo Agathanggelou,[e†]*

(a) *School of Chemistry, University of Birmingham, Edgbaston, Birmingham, West Midlands, B15 2TT, UK.*
(b) *Sygnature Discovery, The Discovery Building, BioCity, Pennyfoot Street, Nottingham, NG1 1GR, UK.*
(c) *Present address: BioMedha Limited, Biocity, Innovation Building, Pennyfoot Street, Nottingham NG1 1GF, UK.*
(d) *Present address: Excellerate Bioscience Limited, Discovery Building, Biocity, Pennyfoot Street, Nottingham, NG1 IGR, UK.*
(e) *Institute for Cancer and Genomic Sciences, University of Birmingham, Edgbaston, Birmingham, West Midlands, B15 2TT, UK.*
(f) *Winterbourne Botanic Garden, University of Birmingham, 58 Edgbaston Park Road, Edgbaston, Birmingham, West Midlands, B15 2RT, UK.*
(g) *School of Pharmacy and Biomolecular Sciences, Liverpool John Moores University, Byrom Street, Liverpool, L3 3AF, UK.*
(h) *X-Ray Crystallography Facility, School of Chemistry, University of Birmingham, Edgbaston, Birmingham, West Midlands, B15 2TT, UK.*
(i) *ApconiX Ltd, Alderly Park, Nether Alderly, Cheshire, SK10 4TG, UK.*
(j) *School of Biosciences, University of Birmingham, Edgbaston, Birmingham, West Midlands, B15 2TT, UK.*

Table of Contents 1
Author Contributions 2
General equipment and apparatus 2
Chemistry efforts at University of Birmingham 2
Chemistry efforts at Sygnature Discovery (where differing to University of Birmingham) 3
Biological efforts at University of Birmingham 3
Biological efforts at Sygnature Discovery (where differing to University of Birmingham) 4
Cultivation of feverfew varieties 4
Growing medium 4
Seed sources 4
Seed Sowing 5
Potting on 5
Planting out 5
Parthenolide extraction procedure 6
Synthesis 7
Model reactions 7
General procedure 1 8
General procedure 2 8
Synthesis of parthenolide derivatives 9
alamarBlue® assay 36
Calculated properties of parthenolide derivatives 39
Computational study 41
In silico ligand binding study 41
Docking scores 44
Inhibitor structure 44
Toxicology efforts hERG liability assay at Apconix Ltd 45
Spectroscopy and spectrometry 47
NMR spectrums obtained from efforts by University of Birmingham 47
Proton NMR spectrums obtained from higher throughput synthesis efforts by Sygnature Discovery 78
Mass spectrometry of lead compound 5f 116
References 118

Author Contributions

AA, JSF and XL wrote most of the text and all authors contributed to critical aspects of the research and decision-making processes presented in this manuscript. AA (University of Birmingham, School of Cancer Sciences) conceived the overarching project and led the biological aspects of the research programme. JSF (University of Birmingham, School of Chemistry) led the chemistry aspects of the research programme. JSF and AA co-supervised the research and worked jointly on all aspects. DTP, XL and MJD (University of Birmingham, School of Chemistry) conducted synthetic chemistry research, MJD conducted critical feasibility studies, DTP and XL extracted and synthesised a series of parthenolide derivatives within and out with this manuscript, XL also conducted biological screening of samples thereof. CF, RL and RS (University of Birmingham, School of Cancer Sciences) conducted preliminary assays for anti-CLL activity and contributed to developing the protocols adopted in this manuscript. TS (University of Birmingham, School of Cancer Sciences) leads the laboratory in which the overarching anti-CLL project is conducted, provided the framework, advice and resources that underpinned the biological aspects and provided critical input at project junctures. AGL conducted the *in-silico* ligand binding studies and led on that section of the supplementary material. MJM and RR (ApconiX) devised and conducted hERG liability screening of the supplementary material. AQ (University of Birmingham, School of Chemistry) devised and executed the synthesis of compound **7**. LM with the (University of Birmingham, School of Chemistry) collected and analysed single crystal XRD data discussed in this manuscript. AG and LH (Winterbourne Botanic Garden) led the effort to the cultivate *feverfew* and varieties thereof from which parthenolide has been extracted. GJ, DH, JTB, BS, EM (Sygnature Discovery) were involved in the design and medicinal and synthetic chemistry activities to parthenolide derivatives. BA, NB & RL (Sygnature Discovery) optimised the alamarBlue® assay to a 384-well format and determined the activity of most of the parthenolide derivatives in this manuscript against the MEC1 cell line. TSU (Sygnature Discovery) led the DMPK activities. In addition, AA, JSF, TS-U, GJ and JTB collaboratively evaluated results and selected compounds for further progression.

General equipment and apparatus

Chemistry efforts at University of Birmingham

All commercially available solvent, catalysts and reagents were purchased and used from suppliers without any further purification. At the *University of Birmingham*, proton NMR spectra were recorded at 300 MHz on a Bruker AVIII300 NMR spectrometer or at 400 MHz on a Bruker AVIII400 NMR spectrometer. Carbon NMR spectra are proton decoupled and were recorded at 101 MHz on a Bruker AVIII400 NMR spectrometer at room temperature. Various one- and two-dimensional NMR spectroscopy techniques, including J-MOD, PENDANT, UDEFT, HSQC and COSY, were used to confirm assignments in some cases. Chemical shifts (δ) were reported in ppm relative to TMS (δ 0.00) for ^{1}H NMR and to chloroform (δ 77.16) for ^{13}C NMR spectroscopy; coupling constants (*J*) are expressed in Hertz (Hz). The following abbreviations are used for multiplicities: s = singlet, d = doublet, t = triplet, q = quartet, quint = quintet, m = multiplet, pent = pentet, hex = hextet, and br = broad. At the *University of Birmingham* mass

spectra were recorded on an electrospray MS Waters LCT Time of Flight Mass Spectrometer and with EI (GC/MS) Waters GCT Premier Time of Flight Mass Spectrometer. Infrared Spectra Varian 660-IR FT-IR spectrometer at room temperature using an ATR attachment. Melting points were measured using a StuartTM digital melting point apparatus (SMP10) and reported as a range. Specific optical rotations were recorded on an Optical PolAAr 2001 automatic polarimeter at room temperature. The X-ray crystal structure included was previously reported; for information, it was determined using an Agilent SuperNova X-ray diffractometer with an Atlas detector (wavelength 1.5418 Å). Column chromatography was carried out using standard flash column chromatography and a Combiflash Rf 200i (stationary phase silica), chromatograms were recorded by evaporative light scattering detector (ELSD) and absorbance at two wavelengths (254 nm and 280 nm). Reactions were monitored by thin layer chromatography (TLC) on Merck silica gel 60 F254 plates. TLC plates were visualised by either UV light with 254 nm / 365 nm, a methanolic solution of ninhydrin or with potassium permanganate. Where dry solvents were required, pre-treatment with molecular sieves 3 Å was sufficient to permit reactions to proceed with good conversations.

Chemistry efforts at Sygnature Discovery (where differing to University of Birmingham)

NMR spectra were recorded on a Bruker AVIII 400MHz NMR spectrometer at room temperature. Mass spectra were recorded on Waters Acquity UPLC, CSH C18, 1.7 µm, 2.1 x 30 mm column, 3 minutes acidic method as described follows: Analytical UPLC/MS was carried out using a Waters Acquity UPLC, CSH C18, 1.7 µm, 2.1 x 30 mm column eluting with a gradient of 0.1% formic acid in MeCN in 0.1% formic acid in water. The gradient is structured with a starting point of 5% MeCN held from 0.0-0.11 minutes. The gradient from 5-95% occurs between 0.11-2.15 minutes with a flush from 2.15-2.56 minutes. A column re-equilibration to 5% MeCN is from 2.56-2.83 minutes. UV spectra of the eluted peaks were measured using an Acquity PDA (scan from 150-800 nm) and mass spectra were recorded using an Acquity QDa detector with ESI pos/neg switching.

Biological efforts at University of Birmingham

Tissue culture, MEC1 cells were obtained from the American Type Culture Collection (Manassas, VA 20110 USA) and were cultured in RPMI 1640 medium (Sigma-Aldrich, Irvine, UK) with 10% fetal bovine serum (Sigma-Aldrich). The alamarBlue® cytotoxicity assay was conducted by seeding MEC1 cells in triplicate at density of 25000 cells/well in a 96 well plate, final volume of 200 µL. Following treatment with test compound, viability was determined by measuring the reduction of resazurin. Resazurin solution was added to each well at a final concentration of 50 µg/mL and incubated for 3 hours at 37 °C with 5% CO_2. Reduction of resazurin was determined by measuring absorbance at 590 nM using a PheraSTAR FS plate reader (BMG Labtech). Cell viability was calculated as a fraction of the untreated cells after subtracting background fluorescence of resazurin in media only. Data is presented as the mean of five independent experiments and significance was determined by Student's t-test.

Biological efforts at Sygnature Discovery (where differing to University of Birmingham)

MEC1 cells were obtained from the American Type Culture Collection (Manassas, VA 20110 USA) and were routinely cultured in RPMI 1640 medium (Sigma-Aldrich) supplemented with 1% L-glutamine (Sigma-Aldrich), 1% Penicillin/Streptomycin (Sigma-Aldrich) and 10% FBS (Gibco). The MEC1 proliferation assay was conducted by performing a re-feed of the MEC1 cells 24 h prior to cell seeding. On the day of the assay, cells were seeded into a 384-well assay plate at a density of 12,500 cells/well in 25 µL of complete MEC1 medium. Compound treatment was performed on the day of cell seeding, with compounds prepared as duplicate compound response curves (final DMSO concentration of 0.6%). Post compound treatment, MEC1 assay plates were returned to a 37 °C/5% CO_2 incubator for 24 h of incubation. Following compound treatment, cellular viability was determined by measuring reduction of alamarBlue® reagent (Invitrogen). alamarBlue® reagent (2.5 µL) was added to each assay well to give a 1:10 dilution of the neat stock and assay plates were incubated for 3 h at 37 °C/5% CO_2. Reduction of alamarBlue® was determined using an Envision Xcite plate reader (Perkin Elmer) with an excitation wavelength of 560 nm set and an emission wavelength of 590 nm set. Cell viability was determined by normalising raw fluorescent data to a cell only high control and Parthenolide treated low control to provide percentage viability readout. Data was plotted and EC_{50} values extracted and reported EC_{50}s represent an average of at least two independent assays.

Cultivation of feverfew varieties

Growing medium

Seeds were sown in Petersfield peat-free supreme compost; a general seed and potting compost produced from well composted finely screened bark. Compost produced by W E Hewitt & Son.

Seed sources

A variety of sources of *feverfew*, seeds have been exploited over the period 2013-2018. The commercial supplier and the products purchased are listed below, the suppliers' name for the seeds are given. Feverfew of the family: asteraceae; genus: *Tanacetum* can be annuals, evergreen or herbaceous perennials or sub-shrubs, with simple or pinnately divided leaves and solitary or clustered, terminal, daisy-like or button-like flower-heads; detail: *T. parthenium* is a short-lived bushy perennial with pungently aromatic, ovate, pinnately lobed leaves and daisy-like flowerheads 2.5 cm across, with white rays and yellow disk florets in summer; plant range: Balkans,[1] is also known by alternative common names including Altamisa, Bachelor's Buttons, Featherfew, Featherfoil, Flirtwort Midsummer Daisy, Grande Camomille and Santa Maria. *Tanacetum parthenium* is recognised as having had 26 synonyms.[2]

Seeds purchased were listed by seed merchants as *Tanacetum parthenium* (feverfew), which has daisy-like flowers. Since 2017 seeds sown consistently produced button-like flowers (*Tanacetum parthenium 'Flore Pleno'*) instead of the single flowers described for the species (*Tanacetum parthenium*), regardless of the supplier.

Supplementary Table 1

Entry	Supplier	Supplier's Seed Name	Year Sown
1	CN Seeds	Green Feverfew; *Tanacetum parthenium*	2013
2	CN Seeds	Green Feverfew; *Tanacetum parthenium*	2014
3	Chase Garden Seeds	Feverfew	2015
4	Kings Seeds	Feverfew; *Tanacetum parthenium*	2015
4	Nicky's Herbs	Feverfew	2016
6	Kings Seeds	Feverfew; *Tanacetum parthenium*	2017
7	Seed Parade	Feverfew; *Tanacetum parthenium*	2018

Seed Sowing

Seed was grown in an unheated glasshouse. The sowing date was determined by temperature. *Tanacetum* germinates in 7-10 days at temperatures between 15-22 °C.[3] Sowing dates varied between January and April. Seed was sown into plastic modular trays of 10 x 5 modules (each tray 51.5 cm long x 29.5 cm wide by 4.5 cm in depth). Three to four seeds were sown per module and left uncovered. Mains tap water was used for watering the seed trays, which were checked daily. Watering was carried out using a Haws 170/1.5 watering can with fine brass rose. Following germination, seedlings were thinned out using forceps. One seedling was left per module to grow on.

Potting on

When the seedlings had developed roots that filled each module, they were pricked out into 10 cm pots (Teku VDC 10 plastic pots) containing Petersfield peat-free supreme compost. The feverfew in 10 cm pots were moved to a sheltered position in between two glasshouses to harden off (subject to weather conditions) once they had put on growth. Growth was assessed by increase in height/width of plant and tapping individual plants out of their pots to check root growth.

Planting out

From the outset of the project, once plants had a well-developed root system, they were planted out on to a west facing, sloping and free draining site. The site was previously used to grow another member of the compositae; *Anthemis*. The soil had previously had grit added but no feeding or mulching had taken place for several years. Plants were spaced 50 cm apart, with 50 cm between rows. They were watered in once planted and checked for watering while they were establishing roots, and in periods of hot weather.

Weeds were a problem on the site, so plants were mulched with woodchip to reduce weeding. A large number of plants would grow for two years on this site-feverfew tends to be grown as an annual but will grow as a short-lived perennial in ideal conditions.

In 2017 the growing area changed to accommodate more plants. The new site was also on a west facing slope. It was less free draining than the previous site and has been regularly improved with feed and compost, for use growing vegetables in the past. Feverfew plants were grown at 30 cm spacings to provide dense cover with the intention of

shading out weed competition. Plants grew well but did not come through the winter of 2017/18 making it necessary to replant the field.

Parthenolide extraction procedure

Plant material (*feverfew*) was chopped into 1 to 5 cm pieces by hand and allowed to dry, laid out on plastic sheets in a glass house. Once dry to touch, and taking care not to discard any seeds, the dried material was transferred to vacuum sealed containers, and stored until an appropriate time to conduct the next, extraction step. To the dried plant material hot water (10 mL per 1 g plant matter) was added and the mixture stirred at 80 °C for 10 minutes. The solution was allowed to cool to room temperature and the cool broth thus obtained decanted, this procedure was repeated twice more on the same batches of plant material. The combined aqueous broth extracts were filtered. The filtrate was extracted three times with chloroform (3 x vol 1:1). The majority of the solvent from the combined organic extracts was removed *in vacuo* to afford a dark green sticky residue. The residue thus obtained was purified by flash chromatography (hexane/ethyl acetate) and recrystalised to purity from with hexane/ethyl acetate to afford colourless crystals of parthenolide **1**. A typical procedure used 6.5 kg of dried plant material delivered 8.53 g of analytically pure parthenolide (0.13 wt%).

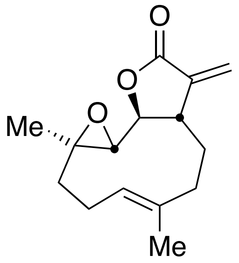

Parthenolide was isolated following the above extraction and isolation procedure. ^{1}H NMR (300 MHz, $CDCl_3$) δ 6.35 (d, *J* 3.7, 1H, C=C*H*$_2$), 5.64 (d, *J* 3.3, 1H, C=C*H*$_2$), 5.23 (dd, *J* 12.0 & 2.4, 1H, CH_3C=C*H*), 3.88 (t, *J* 8.6, 1H, C*H*OCO), 2.93-2.68 (m, 2H), 2.51-2.34 (m, 2H), 2.21-2.12 (m, 3H), 1.81-1.71 (m, 4H), 1.37-1.14 (m, 5H); ^{13}C NMR (101 MHz, $CDCl_3$) δ 17.0, 17.3, 24.2, 30.6, 36.4, 41.2, 47.7, 61.6, 66.4, 82.5, 121.3, 125.3, 134.6, 139.2, 169.3); IR (neat, cm^{-1}): 1753 (C=O); MS (EI+) m/z: $[M+Na]^+$ 271.2; HRMS ES+ *m/z*: $[M+Na]^+$ calcd. for $C_{15}H_{20}O_3Na^+$: 271.1305, found: 271.1312. mp: 115-117 °C. The characterisation data matched that from a sample previously obtained by where the XRD single crystal structure (Supplementary Figure 1) was previously deposited and noted in an earlier manuscript (CCDC 1012153),[4] the structure is stereochemically consistent with that reported in other CDCC deposited structures.[5]

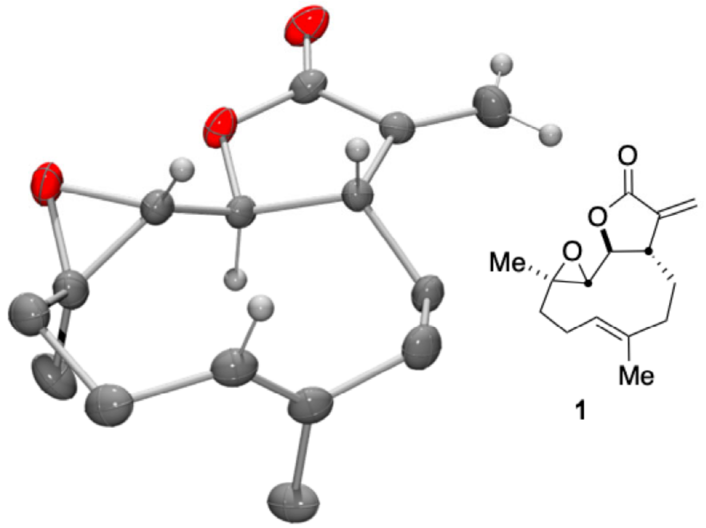

Supplementary Figure 1. *The crystal structure (left) and chemical structure (right) of parthenolide, as previously shown by co-authors of this report (CCDC 1012153),[4] included here for completeness.*

Synthesis

Model reactions

Tulipane (**S1**) was used as a model substrate to assess conditions for amination (by 1,4-conjugate addition) of 3-methylene gamma lactones (Supplementary Scheme 1). It was confirmed by proton NMR spectroscopy that a 1:1 ratio of electrophile (**S1**) to test-nucleophile (benzylamine) in methanol at room temperature cleanly yielded desired 1,4-aminated product **S2**. Furthermore, treatment of tulipane with excess (4 equivalents) of benzylamine, under otherwise parallel conditions, resulted in a ring-opened, double aminated species **S3**. Furthermore, the use of base (e.g. potassium carbonate) proved to be non-deleterious, meaning amine salts may be converted to their free bases *in situ* by addition of a suitable inorganic base.

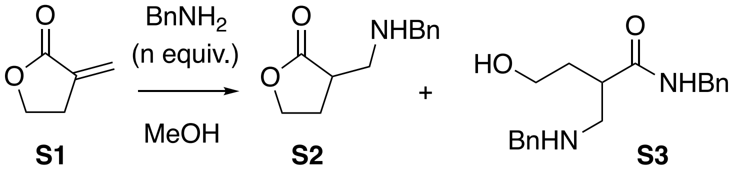

n	S2:S3
1	1:0
1.5	1:1
4	0:1

Supplementary Scheme 1. *Model reactions of tulipane (S1) showing excess amine leads to formation of less desired product (S2) and more of an undesired ring opened product (S3).*

These preliminary experiments helped establish conditions for general amination of such rings and confirmed forcing conditions with excess amines might be suboptimal in terms of conversion to and yield of desired 1,4-aminated products.

*Synthesis of 3-((benzylamino)methyl)dihydrofuran-2(3H)-one (**S2**)*

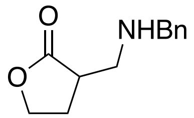

Benzylamine (83 mg, 0.77 mmol) was added to a stirred solution of tulipane (75 mg, 0.77 mmol) in methanol (5 cm^3) for at rt for 66 h. The resulting reaction mixture was concentrated *in vacuo* and purified by column chromatography (silica, $CHCl_3$:MeOH 10:1) to afford **S2** as a pale yellow oil (98 mg, 62%). ^{1}H NMR (300 MHz, $CDCl_3$) δ 7.35-7.13 (m, 6H), 4.30 (td, *J* 8.8 & 2.9, 1H), 4.21-4.10 (m, 1H), 3.75 (d, *J* 2.4, 2H), 2.94-2.61 (m, 3H), 2.36-2.23 (m, 1H), 2.16-2.01 (m, 1H); IR (neat) cm^{-1} 3316 (NH), 3029, 1762; TOF-MS (ESI+) *m/z* 206.1 $[M+H]^+$, 228.1 $[M+Na]^+$. HRMS (ES+) m/z: [M+H]+ calcd. for $C_{12}H_{16}NO_2^+$, 206.1176; found: 206.1181.

*Synthesis of N-benzyl-2-((benzylamino)methyl)-4-hydroxybutanamide (**S3**)*

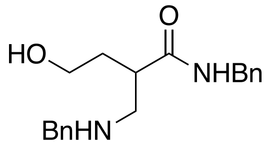

Benzylamine (330 mg, 3.08 mmol) was added to a stirred solution of tulipane (75 mg, 0.77 mmol) in methanol (5 cm^3) for 66 h at rt. The resulting reaction mixture was reduce *in vacuo* and purified by column chromatography (silica, $CHCl_3$:MeOH 10:1) to afford **S3** as a clear crystalline solid (0.162 g, 68%). $C_{19}H_{24}O_2N_2$; ^{1}H NMR (400 MHz, $CDCl_3$) δ 7.40-7.15 (m, 10H), 4.43 (d, *J* 5.5, 2H), 3.79-3.75 (m, 2H), 3.69 (t, *J* 5.5, 2H), 2.90 (ddd, *J* 16.5, 12.1 & 6.0, 2H), 2.74-2.56 (m, 4H), 2.04-1.66 (m, 2H); ^{13}C NMR (101 MHz, $CDCl_3$) δ 175.2, 138.4, 128.7, 128.6, 128.3, 127.8, 127.5, 127.4, 127.2, 126.9, 60.69, 53.8, 50.9, 43.5, 33.6; TOF-MS (ES+) *m/z* 313.2 $[M+H]^+$, 326.2 $[M+Na]^+$, 335.2 $[M+H+MeOH]^+$.

General procedure 1

To a methanol solution (typically 10 mL) of parthenolide (**1**, 1.0 equiv.) the corresponding primary or secondary amine (1.2 equiv.) and potassium carbonate (2.0 equiv.) were added. The resulting solution was stirred at room temperature for 24 hours. Consumption of **1** was confirmed by TLC analysis. The solvent was removed *in vacuo* and the product was purified by flash chromatography (silica gel).

General procedure 2

Synthesis

The corresponding amine (or salt thereof, 0.210 mmol) was weighed into reaction tubes (matrix 1.4 mL tubes), to which parthenolide (**1**, 40 mg, 0.161 mmol) was added; methanol (0.75 mL) and Hünig's base (29.5 μL, 0.169 mmol) were added thereafter. The reaction mixtures were sonicated for an appropriate time to obtain dispersed solutions or mixtures (transparent or opaque). The resulting samples were shaken at room temperature for 17 h. Consumption of reactants was confirmed by UPLC analysis. At which point dimethylsulfoxide (200 μL) was added to the reaction vessel. The reaction mixtures were filtered, and the product was purified by acidic reverse-phase HPLC, as described below.

Purification by acidic reverse-phase HPLC (Waters Acquity)

Waters X-Select CSH column C18, 5 µm (19 x 50 mm), flow rate 26.5 mL min-1 eluting with H_2O-MeCN gradient containing 0.1% v/v formic acid over 6.5 min using UV detection across all wavelengths with PDA as well as a QDa Mass Detector. At-Column Dilution pump gives 1.5 mL min^{-1} MeCN over the entire method, which is included in the following MeCN percentages. Gradient information: 0.0-0.2 min, 20% MeCN; 0.2-5.5 min, ramped from 20% MeCN to 40% MeCN; 5.5-5.6 min, ramped from 40% MeCN to 95% MeCN; 5.6–6.5 min, held at 95% MeCN.

Synthesis of parthenolide derivatives

*Synthesis of (3R,3aS,9aR,10aR,10bS,E)-3-((dimethylamino)methyl)-6,9a-dimethyl-3a,4,5,8,9,9a,10a,10b-octahydrooxireno[2',3':9,10]cyclodeca[1,2-b]furan-2(3H)-one (**2a**)* [6]

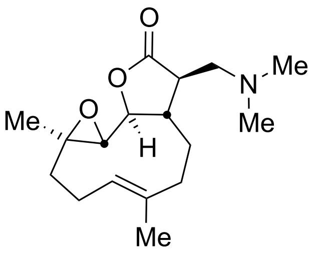

Following *General Procedure 1*, a mixture of parthenolide (400 mg, 1.61 mmol), dimethylamine (2.40 mmol, 1.2 mL, 2M in MeOH) and potassium carbonate (445 mg, 3.22 mmol), was stirred at room temperature in methanol (10 mL) for 24 hours. The title compound was obtained as a colourless solid in 74% yield (351 mg, 1.20 mmol). Data were in agreement with that previously reported.[6] ^{1}H NMR (400 MHz, $CDCl_3$): δ 5.21 (1H, dd, *J* 11.9 & 2.2, C*H*), 3.83 (1H, t, *J* 9.0, C*H*), 2.78-2.70 (2H, m), 2.63 (2H, dd, *J* 13.2 & 4.8, CH_2), 2.47-2.32 (2H, m), 2.28-2.01 (12H, m), 1.70 (2H, s), 1.69-1.59 (1H, m), 1.30 (3H, s, Me), 1.22 (1H, m); ^{13}C NMR (101 MHz, $CDCl_3$): δ 176.5, 134.7, 125.0, 82.1, 66.5, 61.5, 57.7, 47.9, 46.5, 46.2, 41.1, 36.7, 29.9, 24.1, 17.2, 16.9; mp: 145-147 °C; IR (neat, cm^{-1}): 1754 (C=O); TOF-MS (ES+): *m/z* 294.2 [M+H]+; HRMS (ES+) *m/z*: $[M+H]^+$ calcd. for $C_{17}H_{28}NO_3^+$, 294.2064; found: 294.2064.

*Synthesis of (3R,3aS,9aR,10aR,10bS,E)-3-((isopropyl(methyl)amino)methyl)-6,9a-dimethyl-3a,4,5,8,9,9a,10a,10b-octahydrooxireno[2',3':9,10]cyclodeca[1,2-b]furan-2(3H)-one (**2b**)* [9]

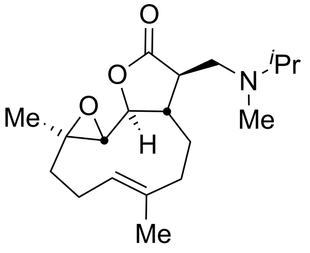

Following *General Procedure 1*, a mixture of parthenolide (100 mg, 0.40 mmol), *N*-isopropylmethylamine (0.48 mmol, 0.05 mL) and potassium carbonate (112 mg, 0.81 mmol), was stirred at room temperature in methanol (10 mL) for 24 hours. The title compound was obtained as a colourless solid in 36% yield (46 mg, 0.14 mmol). 1H NMR (400 MHz, $CDCl_3$): δ 5.21 (1H, dd, *J* 12, 2, CH), 3.82 (1H, t, J 8.8, CH), 2.85-2.65 (4H, m), 2.50-2.01 (10H, m), 1.70 (3H, s, Me), 1.65-1.55 (1H, m), 1.30 (3H, s, NMe), 1.29-1.19 (2H, m,), 1.10 (6H, m); ^{13}C NMR (101 MHz, $CDCl_3$): δ, 176.9, 134.8, 124.9, 82.2, 66.7, 61.5, 54.7, 52.0, 47.8, 46.6, 41.1, 37.3, 36.7, 30.1, 24.1, 17.9, 17.8, 17.2, 17.0; mp: 111-113 °C; $[\alpha]^{18}_{D}$ = -12.40° (c 1, chloroform); IR (neat, cm^{-1}): 1766 (C=O); MS (EI+) m/z: [M+Na]+ 344.50; HRMS (ES+) *m/z*: $[M+H]^+$ calcd. for $C_{19}H_{23}NO_3^+$, 322.2377; found: 322.2383.

*Synthesis of (3R,3aS,9aR,10aR,10bS,E)-3-(((2-hydroxyethyl)(methyl)amino)methyl)-6,9a-dimethyl-3a,4,5,8,9,9a,10a,10b-octahydrooxireno[2',3':9,10]cyclodeca[1,2-b]furan-2(3H)-one (**2c**)* [7]

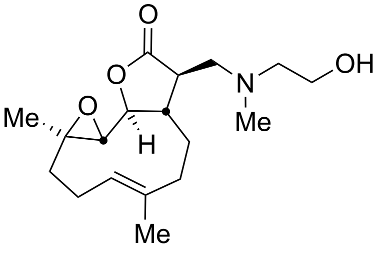

Following *General Procedure 1*, a mixture of parthenolide (100 mg, 0.40 mmol), (2-methoxyethyl)methylamine (0.48 mmol, 0.052 mL) and potassium carbonate (112 mg, 0.81 mmol), was stirred at room temperature in methanol (10 mL) for 24 hours. The title compound was obtained as a yellow semi-solid in 16% yield (21 mg, 0.07 mmol). ^{1}H NMR (400 MHz, $CDCl_3$): δ 5.20 (1H, dd, *J* 2.0 & 12.0, C*H*), 3.80 (1H, t, *J* 9, OC*H*), 3.50 (2H, t, *J* 5.2, OCH_2), 3.35 (3H, t, OMe), 2.85 (1H, dd, *J* 4.8 & 13.6, NCH_2), 2.80-2.70 (2H, m), 2.65 (2H, t, *J* 5.6, NCH_2), 2.45-2.00 (8H, m), 1.70 (3H, s, Me), 1.75-1.65 (1H, m), 1.30 (3H, s, Me), 1.25-1.15 (1H, m); ^{13}C NMR (101 MHz, $CDCl_3$): δ 176.7, 134.7, 124.9, 82.2, 71.0, 66.5, 61.5, 58.8, 57.7, 56.1, 47.6, 46.7, 43.5, 41.0, 36.7, 29.9, 29.7, 24.1, 17.2, 17.0; $[\alpha]^{18}_D$ = -10.01° (c 1, chloroform); IR (neat, cm^{-1}): 1768 (C=O); TOF-MS (EI+) *m/z*: 324.3 $[M+H]^+$, 346.3 $[M+Na]^+$; HRMS (ES+), calcd. *m/z* for $C_{18}H_{29}NNaO_4^+$ $[M+Na]^+$ 346.1989, obs. *m/z* 346.1996.

*Synthesis of (3R,3aS,9aR,10aR,10bS,E)-3-((ethyl(2-hydroxyethyl)amino)methyl)-6,9a-dimethyl-3a,4,5,8,9,9a,10a,10b-octahydrooxireno[2',3':9,10]cyclodeca[1,2-b]furan-2(3H)-one (**2d**)*

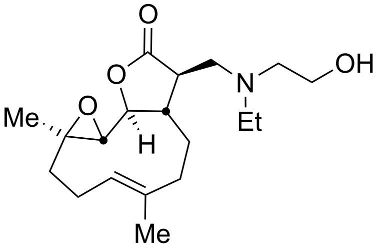

Following *General Procedure 1*, a mixture of parthenolide (100 mg, 0.40 mmol), 2-(ethylamino)ethanol (0.48 mmol, 0.047 mL) and potassium carbonate (112 mg, 0.81 mmol), was stirred at room temperature in methanol (10 mL) for 24 hours. The title compound was obtained as a pale yellow oil in 21% yield (28 mg, 0.08 mmol). ^{1}H NMR (400 MHz, $CDCl_3$): δ 5.20 (1H, dd, *J* 2.0, 12.0, C*H*), 3.85 (1H, t, *J* 9.0, C*H*O), 3.60 (2H, t, *J* 5.2, CH_2O), 2.80 (1H, dd, *J* 4.8, 13.6, CH_2N), 2.75-2.60 (5H, m), 2.45-2.05 (9H, m), 1.70 (3H, s, Me), 1.75-1.65 (1H, m), 1.30 (3H, s, Me), 1.28-1.18 (1H, m), 1.06 (3H, t, *J* 7.2, Me); ^{13}C NMR (101 MHz, $CDCl_3$): δ 176.5, 134.3, 125.3, 82.1, 66.4, 61.5, 58.9, 55.4, 52.4, 48.2, 47.7, 46.5, 41.1, 36.6, 30.0, 24.1, 23.1, 17.2, 16.9, 11.4; $[\alpha]^{18}_D$ = -26.01° (c 1, $CHCl_3$); IR (neat, cm^{-1}): 3418 (OH), 1763 (C=O); MS (EI+) *m/z*: $[M+H]^+$ 338.20.

*Synthesis of (3R,3aS,9aR,10aR,10bS,E)-3-((((1-ethyl-1H-pyrazol-4-yl)methyl)(isopropyl)amino)methyl)-6,9a-dimethyl-3a,4,5,8,9,9a,10a,10b-octahydrooxireno[2',3':9,10]cyclodeca[1,2-b]furan-2(3H)-one (**2e**)*

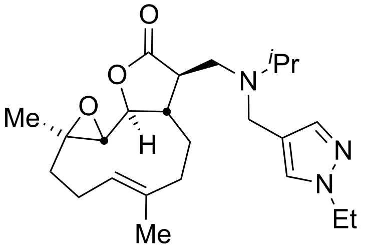

Following the *General Procedure 2*. ^{1}H NMR (400 MHz, DMSO-d_6): δ 7.56 (1H, s), 7.29 (1H, s), 5.13 (1H, d, *J* 6.0, C*H*), 4.07 (2H, q, *J* 8.0, CH_2), 3.92 (1H, t, *J* 9.1), 2.90-2.61 (5H, m), 2.59-2.51 (1H, m), 2.41-2.27 (1H, m), 2.23-1.86 (7H, m), 1.64 (3H, s, Me), 1.63-1.53 (1H, m), 1.34 (3H, t, *J* 7.2, Me), 1.19 (3H, s, Me),

1.15-1.06 (1H, m), 0.99 (3H, d, *J* 6.6, Me), 0.95 (3H, d, *J* 6.6, Me); LC-MS *m/z* $[M+H]^+$ = 416.568.

*Synthesis of (3R,3aS,9aR,10aR,10bS,E)-3-(((cyclopropylmethyl)(propyl)amino)methyl)-6,9a-dimethyl-3a,4,5,8,9,9a,10a,10b-octahydrooxireno[2',3':9,10]cyclodeca[1,2-b]furan-2(3H)-one (**2f**)*

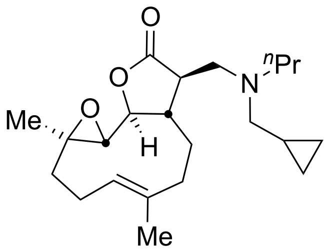

Following *General Procedure 2*. ^{1}H NMR (400 MHz, DMSO-d$_6$): δ 5.11 (1H, d, *J* 8.0, C*H*), 3.88 (1H, t, *J* 9.2), 2.79-2.61 (3H, m), 2.54-2.11 (7H, m), 2.09-1.85 (5H, m), 1.61-1.50 (1H, m), 1.57 (3H, s, Me), 1.42-1.21 (2H, m), 1.12 (3H, s, Me), 1.10-0.98 (1H, m), 0.77 (4H, t, *J* 7.3), 0.38 (2H, m), 0.01 (2H, d, *J* 4.0); LC-MS *m/z* $[M+H]^+$ = 362.41.

*Synthesis of (3R,3aS,9aR,10aR,10bS,E)-3-((cyclohexyl(2-hydroxyethyl)amino)methyl)-6,9a-dimethyl-3a,4,5,8,9,9a,10a,10b-octahydrooxireno[2',3':9,10]cyclodeca[1,2-b]furan-2(3H)-one (**2g**)*

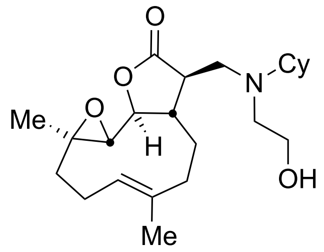

Following *General Procedure 2*. ^{1}H NMR (400 MHz, DMSO-d$_6$): δ 5.20 (1H, d, *J* 6.1, C*H*), 3.94 (1H, t, *J* 9.3), 2.92-2.86 (1H, m), 2.80-2.70 (2H, m), 2.55-1.43 (7H, m), 2.43-2.24 (3H, m), 2.20-1.94 (5H, m), 1.80-1.51 (5H, m), 1.64 (3H, s, Me), 1.25-0.99 (5H, m), 1.19 (3H, s, Me); LC-MS *m/z* $[M+H]^+$ = 392.514.

*Synthesis of (3R,3aS,9aR,10aR,10bS,E)-3-(((4-chlorobenzyl)(methyl)amino)methyl)-6,9a-dimethyl-3a,4,5,8,9,9a,10a,10b-octahydrooxireno[2',3':9,10]cyclodeca[1,2-b]furan-2(3H)-one (**2h**)*

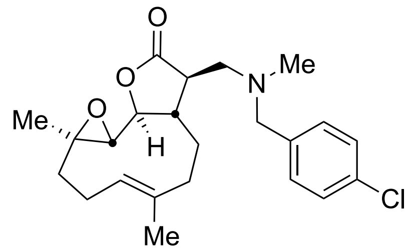

Following *General Procedure 2*. ^{1}H NMR (400 MHz, DMSO-d$_6$): δ 7.35-7.20 (4H, m), 5.17 (1H, dd, *J* 12.2 & 2.4, C*H*), 3.81 (1H, t, *J* 9.0), 3.52 (2H, s, C*H*$_2$), 2.90-2.63 (3H, m), 2.52-2.32 (2H, m), 2.28-1.96 (7H, m), 1.69 (3H, s, Me), 1.68-1.51 (3H, m), 1.29 (3H, s, Me), 1.22 (1H, td, *J* 13.0 & 5.8); LC-MS *m/z* $[M+H]^+$ = 404.345.

*Synthesis of (3R,3aS,9aR,10aR,10bS,E)-3-(((3-ethoxybenzyl)(2-hydroxyethyl)amino)methyl)-6,9a-dimethyl-3a,4,5,8,9,9a,10a,10b-octahydrooxireno[2',3':9,10]cyclodeca[1,2-b]furan-2(3H)-one (**2i**)*

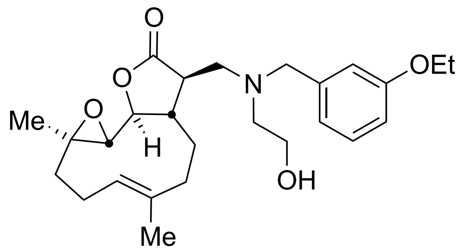

Following *General Procedure 2*. ^{1}H NMR (400 MHz, DMSO-d$_6$): δ 7.22 (1H, t, *J* 7.8, Ar*H*), 6.91 (1H, s, Ar*H*), 6.87 (1H, d, *J* 7.3, Ar*H*), 6.78 (1H, d, *J* 7.9, Ar*H*), 5.14 (1H, d, *J* 11.0, C*H*), 4.48 (1H, s), 4.07-3.89 (3H, m), 3.69-3.45 (4H, m), 2.90-2.56 (4H, m), 2.49-2.19 (3H, m), 2.13-1.92 (5H, m), 1.68-1.53 (1H, m), 1.63 (3H, s, Me), 1.32 (3H, t, *J* 6.9, Me), 1.19 (3H, s, Me), 1.09 (1H, td, *J* 12.6 & 6.0); LC-MS *m/z* $[M+H]^+$ = 444.573.

*Synthesis of (3R,3aS,9aR,10aR,10bS,E)-3-(((3,4-dimethoxybenzyl)(methyl)amino)methyl)-6,9a-dimethyl-3a,4,5,8,9,9a,10a,10b-octahydrooxireno[2',3':9,10]cyclodeca[1,2-b]furan-2(3H)-one (**2j**)*

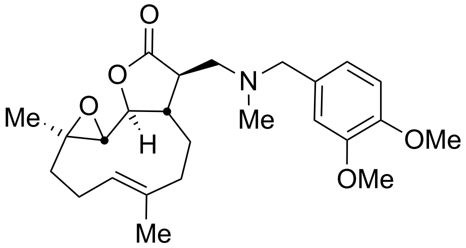

Following *General Procedure 2.* ^{1}H NMR (400 MHz, DMSO-d$_6$): δ .17 (1H, s), 6.93-6.86 (1H, m), 6.80 (1H, dd, *J* 8.2 & 1.9), 5.18 (1H, d, *J* 11.5), 3.96 (1H, t, *J* 9.1), 3.74 (3H, s, Me), 3.73 (3H, s, Me), 3.48-3.36 (2H, m), 2.79-2.56 (4H, m), 2.41-2.15 (3H, m), 2.11 (3H, s), 2.15-1.90 (4H, m), 1.63 (3H, s) 1.70-1.55 (1H, m), 1.20 (3H, s), 1.20-1.00 (1H, m); LC-MS *m/z* [M+H]$^+$ = 430.588.

*Synthesis of (3R,3aS,9aR,10aR,10bS,E)-3-((((1,4,5,6,7,8-hexahydrocyclohepta[c]pyrazol-3-yl)methyl)(methyl)amino)methyl)-6,9a-dimethyl-3a,4,5,8,9,9a,10a,10b-octahydrooxireno[2',3':9,10]cyclodeca[1,2-b]furan-2(3H)-one (**2k**)*

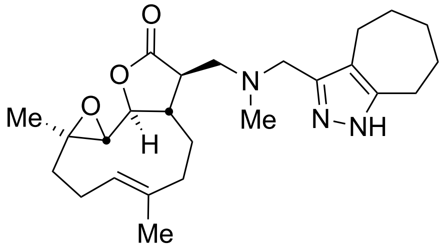

Following *General Procedure 2.* ^{1}H NMR (400 MHz, DMSO-d$_6$): δ 5.16 (1H, dd, *J* 10.1 & 1.4, C*H*), 3.96 (1H, t, *J* 9.1), 3.40 (2H, s, C*H*$_2$), 2.80-2.52 (7H, m), 2.41-2.21 (2H, m), 2.13-1.73 (8H, m), 2.09 (3H, s, Me), 1.63 (3H, s, Me), 1.61-1.48 (5H, m), 1.19 (3H, s, Me), 1.11 (1H, td, *J* 12.4 & 5.9); LC-MS *m/z* [M+H]$^+$ = 428.52.

*Synthesis of (3R,3aS,9aR,10aR,10bS,E)-3-(((5-chloro-2-methoxybenzyl)(ethyl)amino)methyl)-6,9a-dimethyl-3a,4,5,8,9,9a,10a,10b-octahydrooxireno[2',3':9,10]cyclodeca[1,2-b]furan-2(3H)-one (**2l**)*

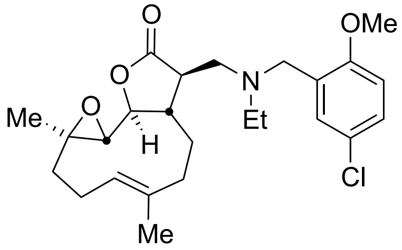

Following *General Procedure 2.* ^{1}H NMR (400 MHz, DMSO-d$_6$): δ 7.37 (1H, d, *J* 2.7, Ar*H*), 7.27 (1H, dd, *J* 8.7 & 2.7, Ar*H*), 7.01 (1H, d, *J* 8.8, Ar*H*), 5.08 (1H, dd, *J* 12.3 & 3.1, C*H*), 3.95 (1H, t, *J* 9.0), 3.78 (3H, s), 3.54 (2H, q, *J* 9.9, C*H*$_2$), 2.87-2.57 (4H, m), 2.45-2.29 (2H, m), 2.24-1.85 (7H, m), 1.70-1.57 (1H, m), 1.63 (3H, s, Me), 1.19 (3H, s, Me), 1.13-1.05 (1H, m), 1.02 (3H, t, *J* 7.0, Me); LC-MS *m/z* [M+H]$^+$ = 448.474.

*Synthesis of (3R,3aS,9aR,10aR,10bS,E)-6,9a-dimethyl-3-((methyl((3-(thiophen-2-yl)-1H-pyrazol-5-yl)methyl)amino)methyl)-3a,4,5,8,9,9a,10a,10b-octahydrooxireno[2',3':9,10]cyclodeca[1,2-b]furan-2(3H)-one (**2m**)*

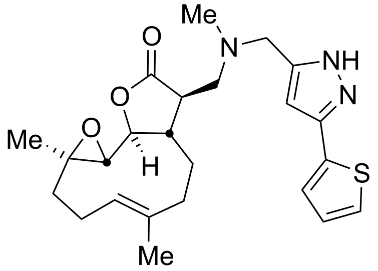

Following *General Procedure 2.* ^{1}H NMR (400 MHz, DMSO-d$_6$): δ 7.44 (1H, s), 7.36 (1H, s), 7.08 (1H, t, *J* 4.5), 6.46 (1H, s), 5.18 (1H, d, *J* 11.3), 3.96 (1H, t, *J* 9.1), 3.59 (2H, d, *J* 9.7), 2.78 (1H, d, *J* 9.0), 2.76-2.58 (2H, m), 2.34 (1H, dd, *J* 8.2 & 5.4), 2.21 (3H, s), 2.25-1.88 (5H, m), 1.68-1.58 (1H, m), 1.63 (3H, s), 1.20 (3H, s), 1.09 (1H, td, *J* 12.7 & 6.1); LC-MS *m/z* [M+H]$^+$ = 442.473.

*Synthesis of (3R,3aS,9aR,10aR,10bS,E)-6,9a-dimethyl-3-((methyl((5-phenyl-1,3,4-oxadiazol-2-yl)methyl)amino)methyl)-3a,4,5,8,9,9a,10a,10b-octahydrooxireno[2',3':9,10]cyclodeca[1,2-b]furan-2(3H)-one (**2n**)*

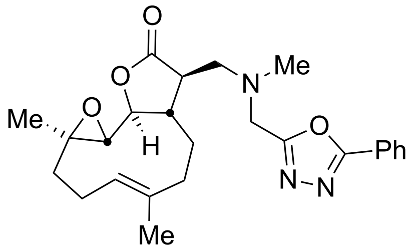

Following *General Procedure 2.* ^{1}H NMR (400 MHz, DMSO-d$_6$): δ 8.03-7.98 (2H, m), 7.68-7.57 (3H, m), 5.14 (1H, d, *J* 11.4), 4.05-3.92 (3H, m), 2.86 (2H, t, *J* 5.5), 2.78 (1H, d, *J* 9.0), 2.70-2.62 (1H, m), 2.34 (3H, s), 2.39-2.22 (2H, m), 2.10-1.91 (5H, m), 1.70-1.60 (1H, m), 1.62 (3H, s), 1.20 (3H, s), 1.08 (1H, td, *J* 12.3 & 5.6); LC-MS [M+H]$^+$ = 438.522.

*Synthesis of (3R,3aS,9aR,10aR,10bS,E)-6,9a-dimethyl-3-((methyl((1-methylpiperidin-4-yl)methyl)amino)methyl)-3a,4,5,8,9,9a,10a,10b-octahydrooxireno[2',3':9,10]cyclodeca[1,2-b]furan-2(3H)-one (**2o**)*

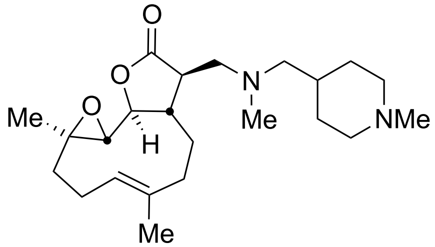

Following *General Procedure 2.* ^{1}H NMR (400 MHz, DMSO-d$_6$): δ 5.19 (1H, dd, *J* 9.8 & 2.5, C*H*), 3.96 (1H, t, *J* 9.1), 2.93-2.56 (6H, m), 2.55 (3H, s, Me), 2.40-1.87 (16H, m), 1.75-1.61 (5H, m), 1.52-1.39 (1H, m), 1.20 (3H, s, Me), 1.19-1.06 (2H, m); LC-MS *m/z* [M+H]$^+$ = 391.414.

*Synthesis of (3R,3aS,9aR,10aR,10bS,E)-6,9a-dimethyl-3-((methyl(3-(trifluoromethyl)benzyl)amino)methyl)-3a,4,5,8,9,9a,10a,10b-octahydrooxireno[2',3':9,10]cyclodeca[1,2-b]furan-2(3H)-one (**2p**)*

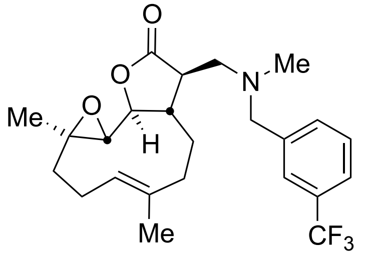

Following *General Procedure 2.* ^{1}H NMR (400 MHz, DMSO-d$_6$): δ 7.70-7.56 (4H, m), 5.17 (1H, d, *J* 11.6), 3.97 (1H, t, *J* 9.1), 3.63 (2H, q, *J* 13.8), 2.80-2.64 (4H, m), 2.42-2.17 (2H, m), 2.15 (3H, s), 2.14-1.90 (5H, m), 1.70-1.60 (1H, m), 1.64 (3H, s), 1.20 (3H, s), 1.10 (1H, td, *J* 12.6 & 5.4); LC-MS [M+H]$^+$ = 438.472.

*Synthesis of (3R,3aS,9aR,10aR,10bS,E)-6,9a-dimethyl-3-((methyl((3-(pyridin-2-yl)-1,2,4-oxadiazol-5-yl)methyl)amino)methyl)-3a,4,5,8,9,9a,10a,10b-octahydrooxireno[2',3':9,10]cyclodeca[1,2-b]furan-2(3H)-one (**2q**)*

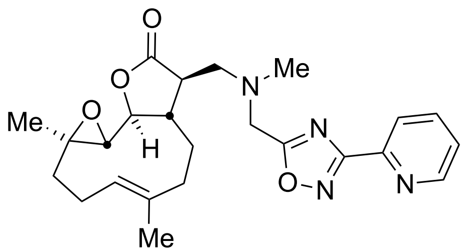

Following *General Procedure 2.* ^{1}H NMR (400 MHz, DMSO-d$_6$): δ 8.78 (1H, dq, *J* 4.8 & 1.6), 8.11 (1H, dt, *J* 7.8 & 1.2), 8.04 (1H, td, *J* 7.7 & 1.8), 7.62 (1H, ddd, *J* 7.5, 4.8 & 1.3), 5.20 (1H, d, *J* 11.3), 4.11 (2H, q, *J* 15.7), 3.99 (1H, t, *J* 9.1), 2.91 (2H, dd, *J* 7.7 & 5.1), 2.80 (1H, d, *J* 9.1), 2.72-2.64 (1H, m), 2.37 (3H, s), 2.42-2.27 (2H, m), 2.15-1.95 (5H, m), 1.72-1.62 (1H, m), 1.64 (3H, s), 1.20 (3H, s), 1.10 (1H, td, *J* 12.9 & 6.1); LC-MS *m/z* [M+H]$^+$ = 439.522.

*Synthesis of (3R,3aS,9aR,10aR,10bS,E)-3-((cyclopropyl((3-methylthiophen-2-yl)methyl)amino)methyl)-6,9a-dimethyl-3a,4,5,8,9,9a,10a,10b-octahydrooxireno[2',3':9,10]cyclodeca[1,2-b]furan-2(3H)-one (**2r**)*

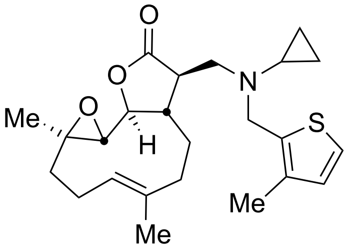

Following *General Procedure 2.* ^{1}H NMR (400 MHz, DMSO-d_6): δ 7.65 (1H, dd, *J* 1.9 & 0.8), 7.48 (1H, dd, *J* 5.0 & 1.3), 7.06-6.95 (2H, m), 6.45 (1H, dd, *J* 3.2 & 1.9), 6.34 (1H, d, *J* 3.1), 5.17 (1H, d, *J* 9.9), 3.97 (1H, t, *J* 9.1), 3.80 (2H, dd, *J* 30.1 & 13.7), 3.63 (2H, dd, *J* 18.6 & 14.5), 2.90-2.81 (2H, m), 2.75-2.67 (2H, m), 2.35 (1H, qd, *J* 13.7 & 4.9), 2.23 (1H, dt, *J* 11.9 & 8.7), 2.14-1.92 (5H, m), 1.65 (4H, s), 1.20 (3H, s), 1.11 (1H, td, *J* 12.8 & 5.8); LC-MS *m/z* ($[M+H]^+$) 416.468.

*Synthesis of (3R,3aS,9aR,10aR,10bS,E)-3-((ethyl((5-propyl-1,2,4-oxadiazol-3-yl)methyl)amino)methyl)-6,9a-dimethyl-3a,4,5,8,9,9a,10a,10b-octahydrooxireno[2',3':9,10]cyclodeca[1,2-b]furan-2(3H)-one (**2s**)*

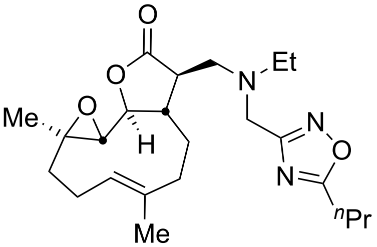

Following *General Procedure 2.* ^{1}H NMR (400 MHz, DMSO-d_6): δ 5.19 (1H, d, *J* 11.3), 3.97 (1H, t, *J* 9.1), 3.78 (2H, q, *J* 14.5), 2.90 (2H, t, *J* 7.4), 2.87 (2H, dd, *J* 5.3 & 2.7), 2.78 (1H, d, *J* 9.1), 2.67-2.46 (2H, m), 2.42-2.27 (2H, m), 2.13-1.93 (5H, m), 1.75 (2H, h, *J* 7.4), 1.67-1.57 (1H, m), 1.65 (3H, s), 1.20 (3H, s), 1.12 (1H, td, *J* 12.6 & 5.8), 1.02 (3H, t, *J* 7.1), 0.95 (3H, t, *J* 7.4); LC-MS *m/z* $[M+H]^+$ = 418.469.

*Synthesis of (3R,3aS,9aR,10aR,10bS,E)-3-(((furan-2-ylmethyl)(thiophen-2-ylmethyl)amino)methyl)-6,9a-dimethyl-3a,4,5,8,9,9a,10a,10b-octahydrooxireno[2',3':9,10]cyclodeca[1,2-b]furan-2(3H)-one (**2t**)*

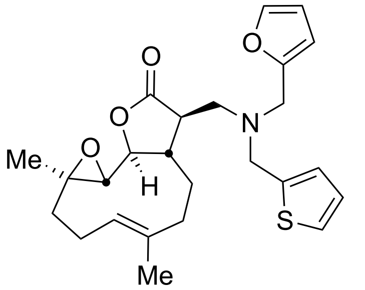

Following *General Procedure 2.* ^{1}H NMR (400 MHz, DMSO-d_6): δ 7.65 (1H, dd, *J* 1.9 & 0.8), 7.48 (1H, dd, *J* 5.0 & 1.3), 7.06-6.95 (2H, m), 6.45 (1H, dd, *J* 3.2 & 1.9), 6.34 (1H, d, *J* 3.1), 5.17 (1H, d, *J* 9.9), 3.97 (1H, t, *J* 9.1), 3.87 to 3.55 (4H, m), 2.90-2.81 (2H, m), 2.75-2.67 (2H, m), 2.42 to 2.29 (1H, m), 2.23 (1H, dt, *J* 11.9 & 8.7), 2.14-1.92 (5H, m), 1.65 (4H, s), 1.20 (3H, s), 1.11 (1H, td, *J* 12.8 & 5.8); LC-MS *m/z* $[M+H]^+$ = 442.423.

*Synthesis of (3R,3aS,9aR,10aR,10bS,E)-3-(((benzo[d][1,3]dioxol-5-ylmethyl)(pyridin-4-ylmethyl)amino)methyl)-6,9a-dimethyl-3a,4,5,8,9,9a,10a,10b-octahydrooxireno[2',3':9,10]cyclodeca[1,2-b]furan-2(3H)-one (**2u**)*

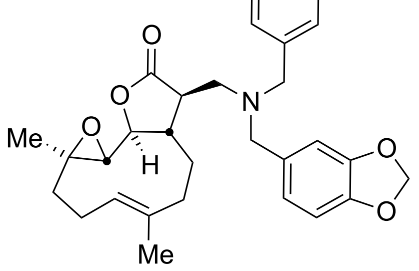

Following *General Procedure 2.* ^{1}H NMR (400 MHz, DMSO-d_6): δ 8.65 (2H, d, *J* 5.8), 7.58 (2H, d, *J* 6.1), 6.93 (1H, d, *J* 1.6), 6.88 (1H, d, *J* 7.9), 6.81 (1H, dd, *J* 7.9 & 1.6), 6.00 (2H, s), 5.14 (1H, d, *J* 10.3), 3.94 (1H, t, *J* 9.1), 3.82 to 3.72 (1H, m), 3.67 to 3.50 (2H, m), 2.88-2.64 (4H, m), 2.42-2.27 (2H, m), 2.12-1.96 (4H, m), 1.83 (2H, t, *J* 12.6), 1.63 (4H, s), 1.18 (3H, s), 1.09 (1H, td, *J* 12.7 & 5.9); LC-MS *m/z* $[M+H]^+$ = 491.433.

*Synthesis of (3R,3aS,9aR,10aR,10bS,E)-3-(((2-methoxyethyl)((1,3,5-trimethyl-1H-pyrazol-4-yl)methyl)amino)methyl)-6,9a-dimethyl-3a,4,5,8,9,9a,10a,10b-octahydrooxireno[2',3':9,10]cyclodeca[1,2-b]furan-2(3H)-one (**2v**)*

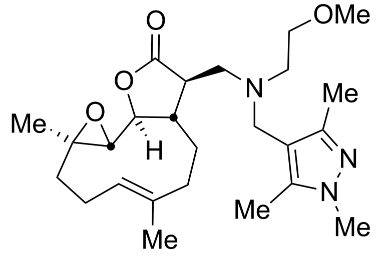

Following *General Procedure 2.* ^{1}H NMR (400 MHz, DMSO-d_6): δ 5.12 (1H, d, *J* 10.8), 3.93 (1H, t, *J* 9.1), 3.61 (3H, s), 3.45-3.25 (12H, m), 2.76-2.64 (3H, m), 2.34 (1H, qd, *J* 13.0 & 5.3), 2.19-2.12 (1H, m), 2.17 (3H, s), 2.08 (3H, s), 2.09-1.97 (3H, m), 1.82-1.72 (2H, m), 1.66-1.50 (1H, m), 1.62 (3H, s), 1.19 (3H, s), 1.10 (1H, td, *J* 12.8 & 5.9); LC-MS *m/z* $[M+H]^+$ = 446.524.

*Synthesis of (3R,3aS,9aR,10aR,10bS,E)-3-(((2-(2,4-dimethylphenoxy)ethyl)(methyl)amino)methyl)-6,9a-dimethyl-3a,4,5,8,9,9a,10a,10b-octahydrooxireno[2',3':9,10]cyclodeca[1,2-b]furan-2(3H)-one (**2w**)*

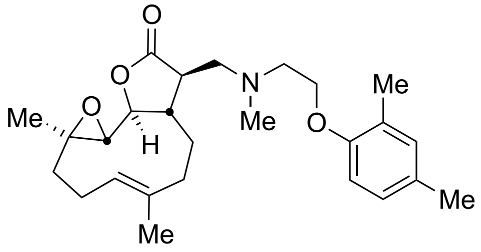

Following *General Procedure 2.* ^{1}H NMR (400 MHz, DMSO-d_6): δ 8.16 (1H, s), 6.93 (2H, d, *J* 6.9), 6.83 (1H, m), 5.01 (1H, d, *J* 10.6), 4.03 (2H, t, *J* 5.6), 3.94 (1H, t, *J* 9.1), 2.83-2.74 (3H, m), 2.66 (1H, d, *J* 9.1), 2.60-2.54 (1H, m), 2.30 (3H, s), 2.19 (3H, s), 2.38-2.17 (2H, m), 2.12 (3H, s), 2.09-1.88 (5H, m), 1.61 (4H, s), 1.18 (3H, s), 1.05 (1H, td, *J* 12.6 & 5.7); LC-MS m/z: $[M+H]^+$ = 428.47.

*Synthesis of (3R,3aS,9aR,10aR,10bS,E)-3-((((8-methoxyquinolin-5-yl)methyl)(methyl)amino)methyl)-6,9a-dimethyl-3a,4,5,8,9,9a,10a,10b-octahydrooxireno[2',3':9,10]cyclodeca[1,2-b]furan-2(3H)-one (**2x**)*

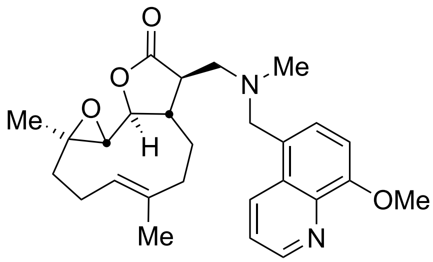

Following *General Procedure 2.* ^{1}H NMR (400 MHz, DMSO-d_6): δ 8.84 (1H, dd, *J* 4.1 & 1.7), 8.63 (1H, dd, *J* 8.5 & 1.7), 8.17 (1H, s), 7.55 (1H, dd, *J* 8.5 & 4.1), 7.42 (1H, d, *J* 7.9), 7.11 (1H, d, *J* 7.9), 4.93 (1H, d, *J* 10.7), 3.96 (3H, s) 3.91-3.76 (3H, m), 2.81-2.66 (2H, m), 2.65-2.54 (2H, m), 2.27 (1H, td, *J* 13.0 & 5.3), 2.15 (3H, s), 2.03-1.92 (3H, m), 1.72 (1H, dd, *J* 12.1 & 6.3), 1.68-1.58 (1H, m), 1.57-1.36 (1H, m), 1.54 (3H, s), 1.15 (3H, s), 1.03 (1H, dt, *J* 12.5 & 6.3); LC-MS *m/z*: $[M+H]^+$ = 451.475.

*Synthesis of (3R,3aS,9aR,10aR,10bS,E)-3-((((5,6-dimethyl-1H-benzo[d]imidazol-2-yl)methyl)(methyl)amino)methyl)-6,9a-dimethyl-3a,4,5,8,9,9a,10a,10b-octahydrooxireno[2',3':9,10]cyclodeca[1,2-b]furan-2(3H)-one (**2y**)*

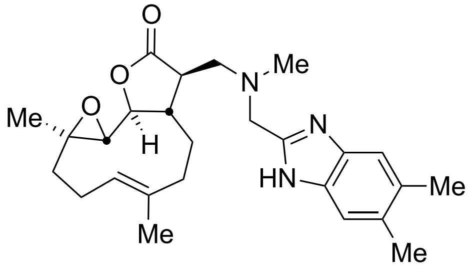

Following *General Procedure 2.* ^{1}H NMR (400 MHz, DMSO-d_6): δ 11.97 (1H, s, NH), 7.27 (1H, s), 5.06 (1H, d, *J* 11.3), 3.94 (1H, t, *J* 9.1), 3.74 (2H, d, *J* 2.6), 2.85-2.71 (2H, m), 2.73 (1H, d, *J* 8.9), 2.58 (1H, dt, *J* 12.0 & 4.4), 2.27 (6H, s, Me), 2.07-1.95 (3H, m), 1.92-1.77 (2H, m), 1.60 (3H, s), 1.18 (3H, s), 1.07 (1H, td, *J* 13.4 & 4.5); LC-MS *m/z* $[M+H]^+$ = 438.522.

*Synthesis of (3R,3aS,9aR,10aR,10bS,E)-3-((ethyl(3-fluorobenzyl)amino)methyl)-6,9a-dimethyl-3a,4,5,8,9,9a,10a,10b-octahydrooxireno[2',3':9,10]cyclodeca[1,2-b]furan-2(3H)-one (**2z**)*

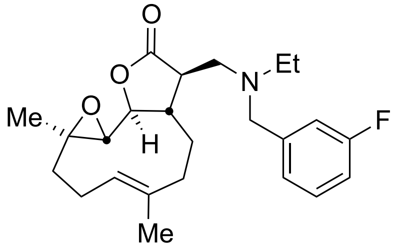

Following *General Procedure 2.* ^{1}H NMR (400 MHz, DMSO-d$_6$): δ 7.38 (1H, q, *J* 8.0), 7.21-7.04 (3H, m), 5.15 (1H, d, *J* 10.0), 3.95 (1H, t, *J* 9.1), 3.60 (2H, dd, *J* 47.1 & 14.4), 2.82-2.60 (4H, m), 2.54-2.28 (3H, m), 2.23-1.90 (6H, m), 1.64 (4H, s), 1.19 (3H, s), 1.10 (1H, td, *J* 12.8 & 5.9), 1.02 (3H, t, *J* 7.0); LC-MS *m/z* [M+H]$^+$ = 402.516.

*Synthesis of (**2aa**) Synthesis of (3R,3aS,9aR,10aR,10bS,E)-6,9a-dimethyl-3-((methyl(phenethyl)amino)methyl)-3a,4,5,8,9,9a,10a,10b-octahydrooxireno[2',3':9,10]cyclodeca[1,2-b]furan-2(3H)-one (**2aa**)*

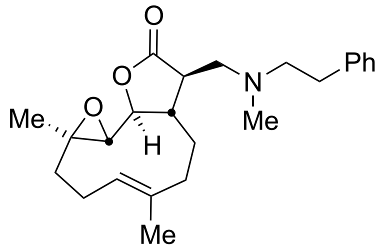

Following the *General Procedure 2.* ^{1}H NMR (400 MHz, DMSO-d$_6$): δ 7.23 (5H, m), 5.12 (1H, dd, *J* 12.0 & 4.0, C*H*), 3.92 (1H, t, *J* 8.0), 2.77-2.63 (5H, m), 2.61-2.51 (3H, m), 2.38-2.29 (1H, m), 2.26 (3H, s, Me), 2.18-1.97 (4H, m), 1.92-1.79 (2H, m), 1.61 (3H, s, Me), 1.60-1.52 (1H, m), 1.18 (3H, s, Me), 1.16-1.06 (1H, td, *J* 12.0 & 8.0); LC-MS *m/z*: [M+H]$^+$ 384.413.

*Synthesis of (3S,3aS,9aR,10aR,10bS,E)-3-(((2-hydroxyethyl)thio)methyl)-6,9a-dimethyl-3a,4,5,8,9,9a,10a,10b-octahydrooxireno[2',3':9,10]cyclodeca[1,2-b]furan-2(3H)-one (**3**)* [7b, 8]

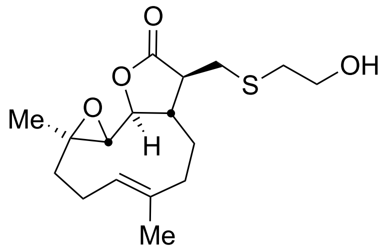

Following *General Procedure 1*, using thiol 2-mercaptoethanol in place of an amine, a mixture of parthenolide (30 mg, 0.12 mmol), 2-mercaptoethanol (14 mg, 0.18 mmol) and triethylamine (18 mg, 0.18 mmol) was stirred in methanol (4 ml) for 26 h at rt. The resulting reaction mixture was reduce *in vacuo* and purified by column chromatography (silica, EtOAc) to afford an impure sample of the title compound obtained as an off-white solid, which was subject to further purification *via* column chromatography (silica, $CHCl_3$:MeOH 20:1) to afford compound **3** as fine colourless needles (17 mg, 44%); mp: 142-144 °C. ^{1}H NMR (300 MHz, $CDCl_3$): δ 5.16 (1H, dd, *J* 12.0 & 2.5,C*H*), 3.88-3.71 (m, 3H), 3.06-2.68 (m, 5H), 2.58 (1H, dt, *J* 12.2 & 4.4, C*H*), 2.45-1.50 (m, 14H), 1.26-1.10 (m, 4H); ^{13}C NMR (101 MHz, $CDCl_3$): δ 175.7, 134.4, 125.3, 82.5, 66.3, 61.6, 61.0, 48.3, 47.5, 41.0, 36.6, 36.4, 29.9, 29.3, 24.1, 17.2, 16.9; IR (neat, cm^{-1}): 3306 (NH), 3189 (OH), 1750 (C=O). TOF-MS (ES+) *m/z* 310.3 [M-OH+H]$^+$, 332.3 [M-OH+Na]$^+$; HRMS (ES+), calcd. *m/z* for $C_{17}H_{25}NaO_3S^+$ [M-OH+Na]$^+$ 332.1417, obs. 332.1851.

*Synthesis of (3R,3aS,9aR,10aR,10bS,E)-3-(((2-hydroxyethyl)amino)methyl)-6,9a-dimethyl-3a,4,5,8,9,9a,10a,10b-octahydrooxireno[2',3':9,10]cyclodeca[1,2-b]furan-2(3H)-one (**4a**)* [7a]

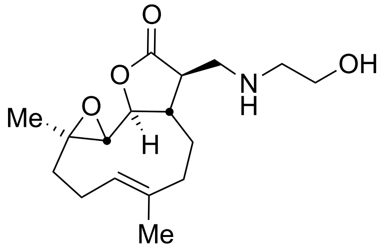

Following *General Procedure 1*, a mixture of parthenolide (100 mg, 0.40 mmol), ethanolamine (0.48 mmol, 0.03 mL) and potassium carbonate (112 mg, 0.81 mmol), was stirred at room temperature in methanol (10 mL) for 24 hours. The title compound was obtained as a pale yellow oil in 77% yield (95 mg, 0.31 mmol); mp: 104-108 °C. ^{1}H NMR (400 MHz, $CDCl_3$): δ 5.35 (1H, dd, *J* 2.0, 12.0, C*H*), 3.90 (1H, t, *J* 9.2, C*H*), 3.65 (2H, t, *J* 4.8, OCH_2), 3.00 (1H, dd, *J* 4.8, 13.6, NCH_2), 2.90-2.70 (4H, m), 2.65-1.85 (8H, m), 1.70 (3H, s, Me), 1.75-1.65 (1H, m), 1.30 (3H, s, Me), 1.28-1.18 (1H, m); ^{13}C NMR (101 MHz, $CDCl_3$): δ 177.0, 134.5, 125.1, 82.6, 66.3, 61.7, 60.8, 51.4, 47.9, 46.8, 41.1, 36.6, 29.9, 24.1, 17.2, 16.9; $[\alpha]^{18}_D$ = -38.12° (c 1, $CHCl_3$); IR (neat, cm^{-1}): 3306 (NH), 3189 (OH), 1750 (C=O); MS (EI+) *m/z*: $[M+H]^+$ 310.23; TOF-MS (ES+) *m/z* 310.3 $[M+H]^+$, 332.3 $[M+Na]^+$; HRMS (ES+), calcd. *m/z* for $C_{17}H_{27}NNaO_4^+$ $[M+Na]^+$ 332.1832, obs. 332.1851.

*Synthesis of (3R,3aS,9aR,10aR,10bS,E)-3-(((2-methoxyethyl)amino)methyl)-6,9a-dimethyl-3a,4,5,8,9,9a,10a,10b-octahydrooxireno[2',3':9,10]cyclodeca[1,2-b]furan-2(3H)-one (**4b**)*

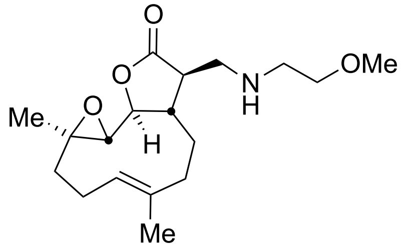

Following *General Procedure 1*, a mixture of parthenolide (100 mg, 0.40 mmol), 2-methyoxyethylamine (0.48 mmol, 0.047 mL) and potassium carbonate (112 mg, 0.81 mmol), was stirred at room temperature in methanol (10 mL) for 24 hours. The title compound was obtained as a yellow solid in 57% yield (74 mg, 0.23 mmol). ^{1}H NMR (400 MHz, DMSO-d_6): δ 5.20 (1H, d, *J* 11.2, C*H*), 3.99 (1H, t, *J* 9.1), 3.46-3.32 (3H, m), 3.24 (3H, s, Me), 2.87-2.59 (5H, m), 2.43-1.92 (6H, m), 1.91-1.78 (1H, m), 1.73-1.61 (1H, m), 1.64 (3H, s, Me), 1.19 (3H, s, Me), 1.17-1.04 (1H, m); ^{1}H NMR (400 MHz, $CDCl_3$): δ 5.18 (1H, dd, *J* 12.2 & 2.1, C*H*), 3.85 (1H, t, *J* 9.1, C*H*), 3.50 (2H, t, *J* 5.2, OCH_2), 3.35 (3H, s, OMe), 3.00 (1H, dd, *J* 13.6 & 4.8, NCH_2), 2.90-2.70 (4H, m), 2.50-2.05 (7H, m), 1.95 (1H, dd, *J* 14.8 & 6.1, CH_2), 1.70 (3H, s, Me), 1.75-1.65 (1H, m), 1.30 (3H, s, Me), 1.25-1.15 (1H, m); ^{13}C NMR (101 MHz, $CDCl_3$): δ 176.7, 134.5, 125.1, 82.5, 72.0, 66.3, 61.5, 58.8, 49.5, 47.9, 47.4, 46.8, 46.7, 41.1, 36.6, 30.0, 24.1, 17.2, 16.9; mp: = 105-107 °C; $[\alpha]^{18}_D$ = -49.60° (c 1, $CHCl_3$); IR (neat, cm^{-1}): 3332 (NH), 1763 (C=O); MS (EI+) *m/z*: $[M+H]^+$ 324.22; MS (E3+) *m/z*: $[M+H]^+$ 324.824 (no UV abs). Compound **4b** was prepared by both *General Procedure 1* and *General Procedure 2* it is from that prepared by *General Procedure 1* that the NMR spectrums in $CDCl_3$ were obtained and from *General Procedure 2* that the NMR spectrum in DMSO-d_6 and the ES+ mass spectrum were obtained, other data reported were collected from the batch resulting from *General Procedure 1*.

*Synthesis of (3R,3aS,9aR,10aR,10bS,E)-3-((benzylamino)methyl)-6,9a-dimethyl-3a,4,5,8,9,9a,10a,10b-octahydrooxireno[2',3':9,10]cyclodeca[1,2-b]furan-2(3H)-one (**4c**)* [7b]

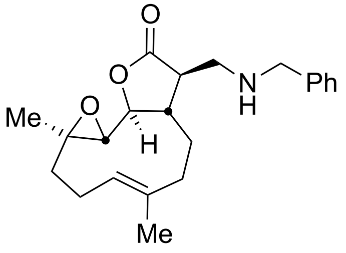

Following *General Procedure 1*, a mixture of parthenolide (50 mg, 0.22 mmol), benzylamine (25 mg, 0.23 mmol) and potassium carbonate (60 mg, 0.44 mmol), was stirred at room temperature in methanol (10 mL) for 16 hours. The title compound was obtained as a pale-yellow solid in 79% yield (62 mg, 0.17 mmol). ^{1}H NMR (400 MHz, $CDCl_3$): δ 7.36-7.29 (4H, m, Ar*H*), 7.28-7.21 (1H, m, Ar*H*), 5.15 (1H, dd, *J* 12.3 & 2.3, C*H*), 3.87 (1H, d, *J* 13.5, C*H*$_2$), 3.83 (1H, t, *J* 9.1, C*H*), 3.75 (1H, d, *J* 13.5, C*H*$_2$), 3.00 (1H, dd, *J* 12.3 & 3.6, C*H*$_2$), 2.76 (1H, dd, *J* 12.2 & 6.1, C*H*$_2$), 2.71 (1H, d, *J* 8.8, C*H*, epoxide moiety), 2.47-2.07 (6H, m), 2.02 (1H, br s, N*H*), 1.98-1.88 (1H, m, C*H*$_2$), 1.79 (1H, dd, *J* 15.0 & 6.2, C*H*$_2$), 1.67 (3H, s, Me), 1.65-1.56 (1H, m, C*H*$_2$), 1.27 (3H, s, Me), 1.20 (1H, td, *J* 12.8 & 5.7, C*H*$_2$); ^{13}C NMR (101 MHz, $CDCl_3$): δ 176.7, 140.1, 134.5, 128.8, 128.0, 127.0, 125.0, 82.5, 66.3, 61.5, 53.8, 48.2, 46.4, 46.0, 40.9, 36.6, 29.9, 24.0, 17.2, 16.9; mp: 160-162 °C; $[\alpha]^{20}_D$ = -29.74° (c 3.8, $CHCl_3$); IR (neat, cm^{-1}): 3338 (NH), 1763 (C=O); HRMS (EI+) *m/z*: $[M]^+$ calcd. for $C_{22}H_{29}NO_3^+$: 356.2142, found: 355.2150; HRMS (ES+) *m/z*: $[M+H]^+$ calcd. for $C_{22}H_{30}NO_3^+$: 356.2220, found: 356.2224.

*Synthesis of (3R,3aS,9aR,10aR,10bS,E)-6,9a-dimethyl-3-((prop-2-yn-1-ylamino)methyl)-3a,4,5,8,9,9a,10a,10b-octahydrooxireno[2',3':9,10]cyclodeca[1,2-b]furan-2(3H)-one (**4d**)* [9]

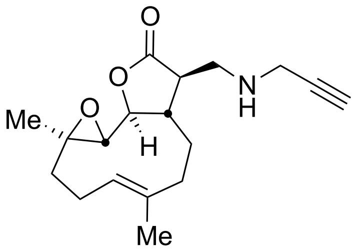

Following *General Procedure 1*, a mixture of parthenolide (100 mg, 0.40 mmol), propargylamine (0.48 mmol, 0.03 mL) and potassium carbonate (112 mg, 0.81 mmol), was stirred at room temperature in methanol (10 mL) for 24 hours. The title compound was obtained as a yellow solid in 36% yield (44 mg, 0.14 mmol). ^{1}H NMR (400 MHz, $CDCl_3$): δ 5.30 (1H, dd, *J* 12.0 & 2.0, C*H*), 3.80 (1H, t, *J* 9.2, C*H*), 3.48 (2H, d, *J* 2.4, NC*H*$_2$), 3.08 (1H, dd, *J* 13.6 & 4.8, NC*H*$_2$), 2.90 (2H, dd, *J* 14.4 & 5.6, C*H*), 2.78 (1H, d, *J* 8.8, C*H*), 2.55-1.85 (8H, m), 1.73 (3H, s, Me), 1.70-1.65(1H, m), 1.31 (3H, s, Me), 1.28-1.18 (1H, m); ^{13}C NMR (101 MHz, $CDCl_3$): δ 176.5, 134.4, 125.2, 82.6, 81.7, 71.7, 66.3, 61.5, 48.0, 46.7, 46.1, 41.1, 38.6, 36.6, 30.0, 24.1, 17.2, 16.9; $[\alpha]^{18}_D$ = -34.03° (c 1, $CHCl_3$); IR (neat, cm^{-1}): 3279 (NH), 1762 (C=O); MS (EI+) *m/z*: $[M+H]^+$ 304.19.

*Synthesis of 3R,3aS,9aR,10aR,10bS,E)-3-(((3-methoxybenzyl)amino)methyl)-6,9a-dimethyl-3a,4,5,8,9,9a,10a,10b-octahydrooxireno[2',3':9,10]cyclodeca[1,2-b]furan-2(3H)-one (**4e**)*

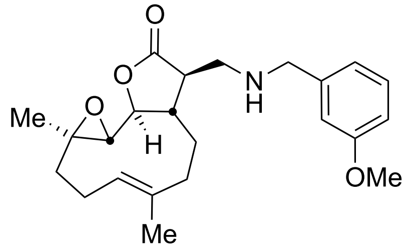

Following *General Procedure 2.* ^{1}H NMR (400 MHz, DMSO-d_6): δ 7.23 (1H, t, *J* 8.0, Ar*H*), 6.96-6.87 (2H, m, Ar*H*), 6.79 (1H, dd, *J* 8.2 & 1.9, Ar*H*), 5.20 (1H, dd, *J* 11.9 & 1.5, C*H*), 3.97 (1H, t, *J* 9.1), 3.75 (3H, s, Me), 3.71 (2H, m, CH_2), 2.84-2.66 (3H, m), 2.40-2.23 (2H, m), 2.16-1.85 (5H, m), 1.83-1.61 (2H, m), 1.64 (3H, s, Me), 1.20 (3H, s, Me), 1.18-1.03 (1H, m); LC-MS *m/z* $[M+H]^+$ = 386.413.

*Synthesis of (3R,3aS,9aR,10aR,10bS,E)-6,9a-dimethyl-3-((((tetrahydro-2H-pyran-4-yl)methyl)amino)methyl)-3a,4,5,8,9,9a,10a,10b-octahydrooxireno[2',3':9,10]cyclodeca[1,2-b]furan-2(3H)-one (**4f**)*

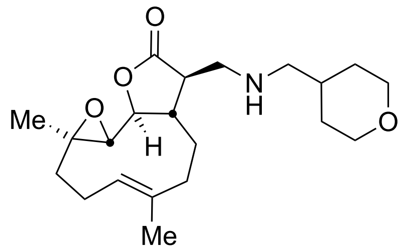

Following *General Procedure 2.* ^{1}H NMR (400 MHz, DMSO-d_6): δ 5.19 (1H, d, *J* 8.0, C*H*), 3.98 (1H, t, *J* 9.1), 3.83 (2H, dd, *J* 11.4 & 3.7), 3.31-3.22 (4H, m), 2.84-2.70 (3H, m), 2.45-1.78 (8H, m), 1.75-1.52 (3H, m), 1.64 (3H, s, Me), 1.19 (3H, s, Me), 1.18-1.03 (2H, m); LC-MS *m/z* $[M+H]^+$ = 364.495 (no UV abs).

*Synthesis of (3R,3aS,9aR,10aR,10bS,E)-6,9a-dimethyl-3-(((2-morpholino-2-oxoethyl)amino)methyl)-3a,4,5,8,9,9a,10a,10b-octahydrooxireno[2',3':9,10]cyclodeca[1,2-b]furan-2(3H)-one (**4g**)*

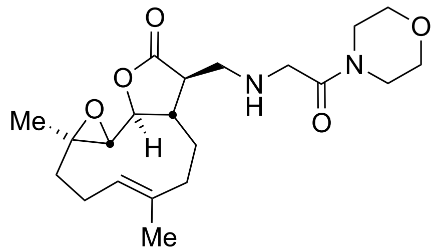

Following *General Procedure 2.* ^{1}H NMR (400 MHz, DMSO-d_6): δ 5.20 (1H, d, *J* 12.0, C*H*), 3.99 (1H, t, *J* 9.1), 3.61-3.51 (3H, m), 3.50-3.35(5H, m), 3.31-3.28 (1H, m), 2.88-2.72 (3H, m), 2.41-1.77 (9H, m), 1.76-1.58 (1H, m), 1.64 (3H, s, Me), 1.20 (3H, s, Me), 1.11 (1H, td, *J* 12.1 & 6.1); LC-MS *m/z* $[M+H]^+$ = 393.200 (poor UV abs).

*Synthesis of (3R,3aS,9aR,10aR,10bS,E)-6,9a-dimethyl-3-(((2-(m-tolyloxy)ethyl)amino)methyl)-3a,4,5,8,9,9a,10a,10b-octahydrooxireno[2',3':9,10]cyclodeca[1,2-b]furan-2(3H)-one (**4h**)*

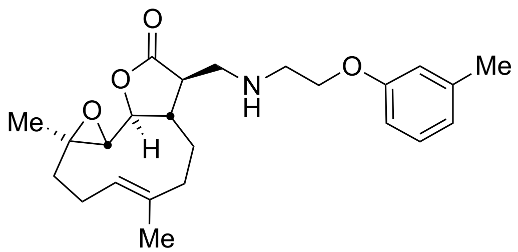

Following *General Procedure 2.* ^{1}H NMR (400 MHz, DMSO-d_6): δ 7.16 (1H, t, *J* 7.7, Ar*H*), 6.79-6.67 (3H, m, Ar*H*), 5.13 (1H, d, *J* 12.0, C*H*), 4.06-3.91 (3H, m), 2.95-2.75 (4H, m), 2.74 (1H, d, *J* 9.1), 2.68-2.53 (1H, m), 2.44-2.20 (2H, m), 2.26 (3H, s, Me), 2.18-1.78 (5H, m), 1.72-1.58 (1H, m), 1.62 (3H, s, Me), 1.19 (3H, s, Me), 1.18-1.03 (1H, m); LC-MS *m/z* $[M+H]^+$ = 400.415.

*Synthesis of (3R,3aS,9aR,10aR,10bS,E)-3-(((4-chloro-2-fluorobenzyl)amino)methyl)-6,9a-dimethyl-3a,4,5,8,9,9a,10a,10b-octahydrooxireno[2',3':9,10]cyclodeca[1,2-b]furan-2(3H)-one (**4i**)*

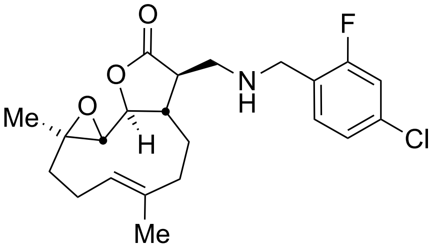

Following *General Procedure 2.* ^{1}H NMR (400 MHz, DMSO-d$_6$): δ 7.35 (1H, t, *J* 8.0, Ar*H*), 7.16-7.06 (2H, m, Ar*H*), 5.17 (1H, dd, *J* 12.2 & 2.6, C*H*), 3.94-3.81 (3H, m), 3.04 (1H, dd, *J* 12.2 & 3.7), 2.82-2.70 (2H, m), 2.60-2.08 (6H, m), 2.03-1.78 (3H, m), 1.74-1.62 (1H, m), 1.68 (3H, s, Me), 1.29 (3H, s, Me), 1.21 (1H, td, *J* 12.7 & 5.8); LC-MS *m/z* [M+H]$^+$ = 408.324.

*Synthesis of (3R,3aS,9aR,10aR,10bS,E)-3-(((3-(3,4-diethoxyphenyl)propyl)amino)methyl)-6,9a-dimethyl-3a,4,5,8,9,9a,10a,10b-octahydrooxireno[2',3':9,10]cyclodeca[1,2-b]furan-2(3H)-one (**4j**)*

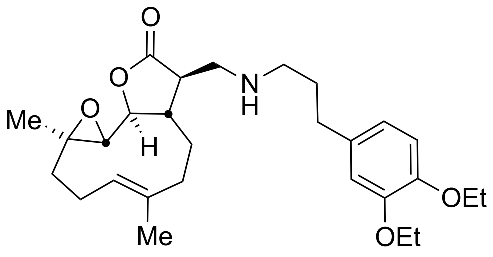

Following *General Procedure 2.* ^{1}H NMR (400 MHz, DMSO-d$_6$): δ 6.83 (1H, d, *J* 8.2, Ar*H*), 6.78 (1H, d, *J* 1.9, Ar*H*), 6.67 (1H, dd, *J* 8.0 & 1.8, Ar*H*), 5.19 (1H, d, *J* 11.2, C*H*), 4.05-3.91 (5H, m), 2.83-2.69 (3H, m), 2.43-1.78 (8H, m), 1.74-1.58 (3H, m), 1.64 (3H, s, Me), 1.34-1.25 (5H, m), 1.20 (3H, s, Me), 1.11 (1H, td, *J* 12.8 & 6.1); LC-MS *m/z* [M+H]$^+$ = 472.579.

*Synthesis of (3R,3aS,9aR,10aR,10bS,E)-3-((((3-(3,4-dimethoxyphenyl)-1,2,4-oxadiazol-5-yl)methyl)amino)methyl)-6,9a-dimethyl-3a,4,5,8,9,9a,10a,10b-octahydrooxireno[2',3':9,10]cyclodeca[1,2-b]furan-2(3H)-one (**4k**)*

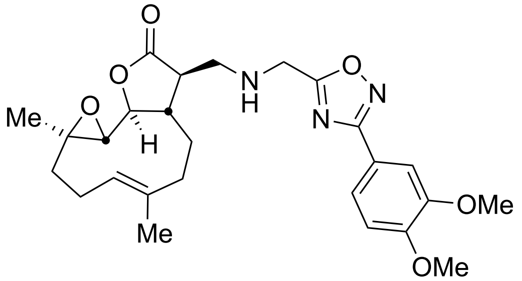

Following *General Procedure 2.* ^{1}H NMR (400 MHz, DMSO-d$_6$): δ 7.62 (1H, dd, *J* 8.4 & 1.9, Ar*H*), 7.50 (1H, d, *J* 2.0, Ar*H*), 7.14 (1H, d, *J* 8.5, Ar*H*), 5.19 (1H, dd, *J* 12.1 & 1.9, C*H*), 4.18-3.95 (3H, m), 3.84 (6H, s, Me), 3.01-2.85 (2H, m), 2.76 (1H, d, *J* 9.1), 2.61-2.52 (1H, m), 2.41-2.26 (2H, m), 2.16-1.97 (4H, m), 1.89-1.80 (1H, m), 1.73-1.63 (1H, m), 1.64 (3H, s, Me), 1.20 (3H, s, Me), 1.10 (1H, td, *J* 12.4 & 5.8); LC-MS *m/z* [M+H]$^+$ = 484.531.

*Synthesis of (3R,3aS,9aR,10aR,10bS,E)-6,9a-dimethyl-3-(((2,4,6-trifluorobenzyl)amino)methyl)-3a,4,5,8,9,9a,10a,10b-octahydrooxireno[2',3':9,10]cyclodeca[1,2-b]furan-2(3H)-one (**4l**)*

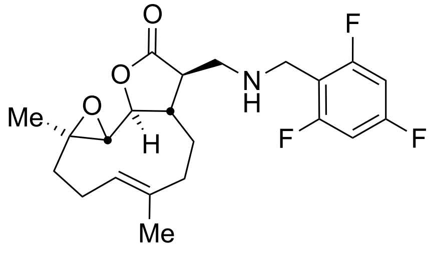

Following *General Procedure 2.* ^{1}H NMR (400 MHz, DMSO-d$_6$): δ 7.31-7.13 (2H, m, Ar*H*), 5.16 (1H, d, *J* 11.0, C*H*), 3.97 (1H, d, *J* 9.1),3.79-3.66 (2H, m), 3.45-3.37 (1H, m), 2.84-2.64 (3H, m), 2.40-1.71 (7H, m), 1.69-1.57 (1H, m), 1.62 (3H, s, Me), 1.18 (3H, s, Me), 1.09 (1H, td, *J* 12.8 & 5.8); LC-MS *m/z* [M+H]$^+$ = 410.367.

*Synthesis of 3R,3aS,9aR,10aR,10bS,E)-3-(((furan-2-ylmethyl)amino)methyl)-6,9a-dimethyl-3a,4,5,8,9,9a,10a,10b-octahydrooxireno[2',3':9,10]cyclodeca[1,2-b]furan-2(3H)-one (**4m**)*

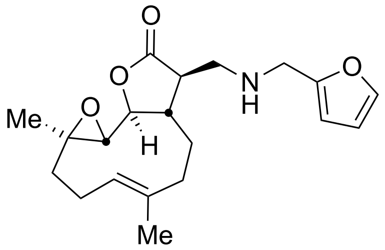

Following *General Procedure 2.* ^{1}H NMR (400 MHz, DMSO-d$_6$): δ 7.61 (1H, dd, *J* 8.4 & 1.9, Ar*H*), 7.50 (1H, d, *J* 2.0, Ar*H*), 7.14 (1H, d, *J* 8.5, Ar*H*), 5.18 (1H, d, *J* 10.2, C*H*), 4.19-3.95 (3H, m), 3.01-2.85 (2H, m), 2.78 (1H, d, *J* 9.1), 2.63-2.51 (1H, m), 2.42-2.25 (2H, m), 2.14-1.98 (4H, m), 1.91-1.79 (1H, m), 1.75-1.60 (1H, m), 1.63 (3H, s, Me), 1.20 (3H, s, Me), 1.11 (1H, td, *J* 12.8 & 5.8), LC-MS *m/z* [M+H]$^+$ = 346.395.

*Synthesis of (3R,3aS,9aR,10aR,10bS,E)-3-(((4-(dimethylamino)benzyl)amino)methyl)-6,9a-dimethyl-3a,4,5,8,9,9a,10a,10b-octahydrooxireno[2',3':9,10]cyclodeca[1,2-b]furan-2(3H)-one (**4n**)*

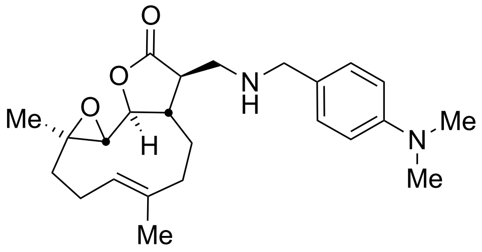

Following *General Procedure 2.* ^{1}H NMR (400 MHz, DMSO-d$_6$): δ 7.14 (2H, d, *J* 8.7, Ar*H*), 6.70 (2H, d, *J* 8.7, Ar*H*), 5.20 (1H, d, *J* 11.4, C*H*), 3.98 (1H, t, *J* 9.1), 3.70-3.53 (2H, m), 2.92-2.66 (2H, m), 2.86 (6H, s, Me), 2.42-2.19 (3H, m), 2.13-1.89 (5H, m), 1.83-1.57 (2H, m), 1.64 (3H, s, Me), 1.20 (3H, s, Me), 1.18-1.05 (1H, m); LC-MS *m/z* [M]$^+$ = 399.167 (shoulder).

*Synthesis of methyl 4-(((((3R,3aS,9aR,10aR,10bS,E)-6,9a-dimethyl-2-oxo-2,3,3a,4,5,8,9,9a,10a,10b-decahydrooxireno[2',3':9,10]cyclodeca[1,2-b]furan-3-yl)methyl)amino)methyl)benzoate (**4o**)*

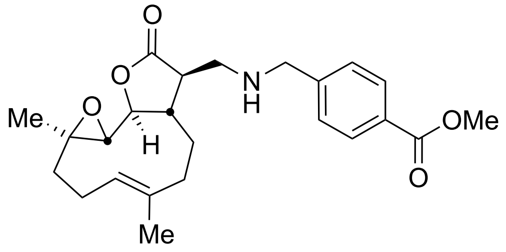

Following *General Procedure 2.* ^{1}H NMR (400 MHz, DMSO-d$_6$): δ 7.93 (2H, d, *J* 8.3), 7.50 (2H, d, *J* 8.3), 5.20 (1H, d, *J* 12.0), 3.99 (1H, t, *J* 9.1), 3.85 (3H, s), 3.81 (1H, d, *J* 5.5), 2.86-2.67 (3H, m), 2.56-2.53 (1H, m), 2.40-2.25 (2H, m), 2.17-1.90 (4H, m), 1.79 (1H, dd, *J* 14.9 & 6.1), 1.71-1.60 (3H, m), 1.20 (3H, s), 1.19-1.05 (1H, m); LC-MS *m/z* [M+H]$^+$ = 414.468.

*Synthesis of (3R,3aS,9aR,10aR,10bS,E)-3-(((2-methoxybenzyl)amino)methyl)-6,9a-dimethyl-3a,4,5,8,9,9a,10a,10b-octahydrooxireno[2',3':9,10]cyclodeca[1,2-b]furan-2(3H)-one (**4p**)*

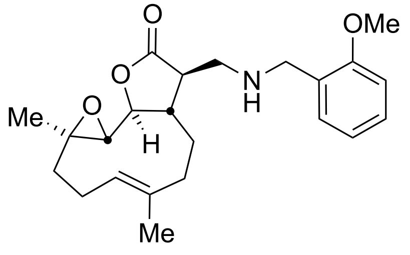

Following *General Procedure 2.* ^{1}H NMR (400 MHz, DMSO-d$_6$): δ 8.19 (1H, s), 7.32 (1H, dd, *J* 7.4 & 1.8), 7.23 (1H, td, *J* 7.8 & 1.8), 6.98 (1H, dd, *J* 8.1 & 1.1), 6.92 (1H, td, *J* 7.4v 1.1), 5.20 (1H, d, *J* 11.9), 3.99 (1H, t, *J* 9.1), 3.79 (3H, s), 3.70 (2H, s), 2.84-2.70 (4H, m), 2.42-2.22 (3H, m), 2.15-1.94 (5H, m), 1.82 (1H, dd, *J* 14.7 & 6.3), 1.72-1.65 (1H, m), 1.64 (3H, s), 1.20 (3H, s), 1.11 (1H, td, *J* 12.8 & 6.1); LC-MS *m/z* [M+H]$^+$ = 386.413.

*Synthesis of (3R,3aS,9aR,10aR,10bS,E)-3-(((2-(dimethylamino)benzyl)amino)methyl)-6,9a-dimethyl-3a,4,5,8,9,9a,10a,10b-octahydrooxireno[2',3':9,10]cyclodeca[1,2-b]furan-2(3H)-one (**4q**)*

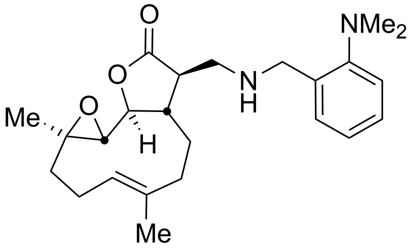

Following *General Procedure 2.* ^{1}H NMR (400 MHz, DMSO-d_6): δ 8.21 (1H, s), 7.39 (1H, dd, *J* 7.5 & 1.7), 7.20 (1H, td, *J* 7.8 & 1.7), 7.09 (1H, dd, *J* 8.2 & 1.3), 7.02 (1H, td, *J* 7.3 & 1.3), 5.19 (1H, d, *J* 11.1), 3.99 (1H, t, *J* 9.1), 3.78 (2H, d, *J* 2.1), 2.85-2.72 (3H, m), 2.64 (6H, s), 2.57-2.51 (1H, m), 2.38-2.25 (3H, m), 2.15-1.94 (5H, m), 1.83 (1H, dd, *J* 14.9 & 6.3), 1.72-1.62 (1H, m), 1.64 (3H, s), 1.20 (3H, s), 1.11 (1H, td, *J* 13.0 & 6.2); LC-MS *m/z* $[M+H]^+$ = 399.465.

*Synthesis of (3R,3aS,9aR,10aR,10bS,E)-6,9a-dimethyl-3-(((2-methyl-2-morpholinopropyl)amino)methyl)-3a,4,5,8,9,9a,10a,10b-octahydrooxireno[2',3':9,10]cyclodeca[1,2-b]furan-2(3H)-one (**4r**)*

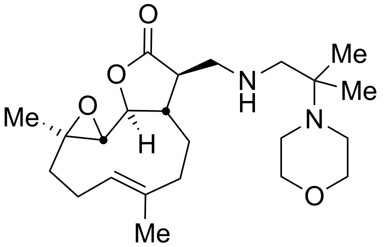

Following *General Procedure 2.* ^{1}H NMR (400 MHz, DMSO-d_6): δ 5.19 (1H, d, *J* 10.9, C*H*), 4.00 (1H, t, *J* 9.1), 3.60-3.50 (4H, m), 2.84-2.72 (3H, m), 2.48-2.10 (9H, m), 2.09-1.92 (4H, m), 1.89-1.79 (1H, m), 1.74-1.61 (1H, m), 1.64 (3H, s, Me), 1.20 (3H, s, Me), 1.18-1.05 (1H, m), 0.97 (6H, s, Me); LC-MS *m/z* $[M+H]^+$ = 407.517 (the mass spec was obtained from the pre-purified material).

*Synthesis of (3R,3aS,9aR,10aR,10bS,E)-6,9a-dimethyl-3-(((2-(thiazol-2-yl)ethyl)amino)methyl)-3a,4,5,8,9,9a,10a,10b-octahydrooxireno[2',3':9,10]cyclodeca[1,2-b]furan-2(3H)-one (**4s**)*

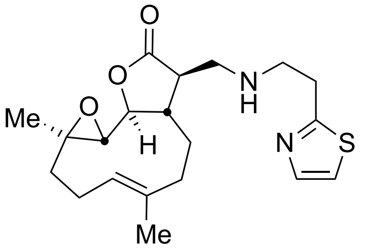

Following *General Procedure 2.* ^{1}H NMR (400 MHz, DMSO-d_6): δ 7.76 (1H, d, *J* 3.3, Ar*H*), 7.68 (1H, d, *J* 3.3, Ar*H*), 5.17 (1H, d, *J* 10.9, C*H*), 4.15 (1H, t, *J* 9.0), 3.47-3.24 (4H, m), 3.02-2.87 (1H, m), 2.79 (1H, d, *J* 9.0), 2.44-1.82 (7H, m), 1.81-1.57 (1H, m), 1.65 (3H, s, Me), 1.32-1.24 (2H, m), 1.21 (3H, s, Me), 1.10 (1H, td, *J* 12.8 & 5.8); LC-MS *m/z* $[M+H]^+$ = 377.412.

*Synthesis of (3R,3aS,9aR,10aR,10bS,E)-3-(((2,6-dichlorobenzyl)amino)methyl)-6,9a-dimethyl-3a,4,5,8,9,9a,10a,10b-octahydrooxireno[2',3':9,10]cyclodeca[1,2-b]furan-2(3H)-one (**4t**)*

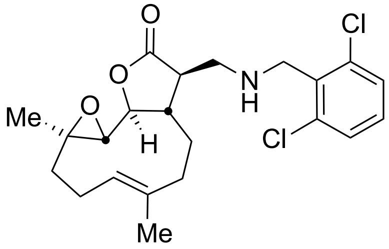

Following *General Procedure 2.* ^{1}H NMR (400 MHz, DMSO-d_6): δ 7.49 (2H, d, *J* 8.0), 7.34 (1H, dd, *J* 8.6 & 7.5), 5.16 (1H, d, *J* 11.8), 4.03-3.93 (3H, m), 2.90 (1H, dd, *J* 12.5 & 4.3), 2.76 (1H, t, *J* 6.0), 2.74 (1H, t, *J* 2.5), 2.57-2.53 (1H, m), 2.41-2.22 (3H, m), 2.05 (3H, ddd, *J* 17.9, 12.0 & 6.8), 1.89 (1H, t, *J* 12.3), 1.80 (1H, dd, *J* 14.7 & 6.2), 1.71-1.65 (1H, m), 1.63 (3H, s), 1.19 (3H, s), 1.10 (1H, td, *J* 12.7 & 5.8); LC-MS $[M+H]^+$ = 424.37.

*Synthesis of (3R,3aS,9aR,10aR,10bS,E)-6,9a-dimethyl-3-(((2-(trifluoromethyl)benzyl)amino)methyl)-3a,4,5,8,9,9a,10a,10b-octahydrooxireno[2',3':9,10]cyclodeca[1,2-b]furan-2(3H)-one (**4u**)*

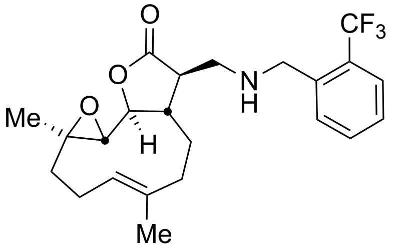

Following *General Procedure 2.* ^{1}H NMR (400 MHz, DMSO-d$_6$): δ 7.81 (1H, d, *J* 7.7), 7.73-7.65 (2H, m), 7.46 (1H, t, *J* 7.6), 5.20 (1H, d, *J* 11.4), 4.00 (1H, t, *J* 9.1), 3.90 (2H, s), 2.92-2.70 (4H, m), 2.57-2.53 (1H, m), 2.44-2.25 (3H, m), 2.15-1.94 (4H, m), 1.64 (3H, s), 1.20 (3H, s), 1.12 (1H, td, *J* 12.7 & 5.8); LC-MS *m/z* [M+H]$^+$ = 424.47.

*Synthesis of (3R,3aS,9aR,10aR,10bS,E)-6,9a-dimethyl-3-(((4-(trifluoromethyl)benzyl)amino)methyl)-3a,4,5,8,9,9a,10a,10b-octahydrooxireno[2',3':9,10]cyclodeca[1,2-b]furan-2(3H)-one (**4v**)*

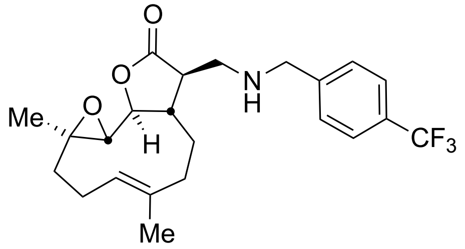

Following *General Procedure 2.* ^{1}H NMR (400 MHz, DMSO-d$_6$): δ 7.69 (2H, d, *J* 8.0), 7.58 (2H, d, *J* 8.0), 5.19 (1H, d, *J* 12.2), 3.99 (1H, t, *J* 9.1), 3.89-3.76 (2H, m), 2.78 (2H, d, *J* 9.1), 2.77 (1H, ddd, *J* 21.7, 12.9 & 4.4), 2.56-2.53 (1H, m), 2.41-2.25 (2H, m), 2.12-1.92 (3H, m), 2.08 (1H, s), 1.78 (1H, dd, *J* 14.7 & 6.2), 1.64 (3H, s), 1.20 (3H s), 1.12 (1H, td, *J* 12.9 & 5.9); LC-MS *m/z* [M+H]$^+$ = 424.37.

*Synthesis of (3R,3aS,9aR,10aR,10bS,E)-6,9a-dimethyl-3-(((4-(trifluoromethoxy)benzyl)amino)methyl)-3a,4,5,8,9,9a,10a,10b-octahydrooxireno[2',3':9,10]cyclodeca[1,2-b]furan-2(3H)-one (**4w**)*

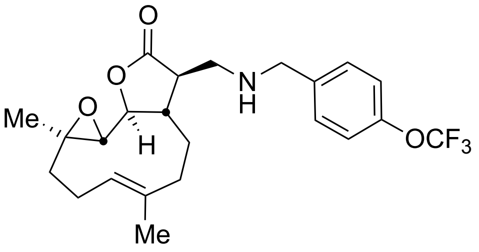

Following *General Procedure 2.* ^{1}H NMR (400 MHz, DMSO-d$_6$): δ 7.47 (2H, d, *J* 8.6), 7.32 (2H, d, *J* 8.4), 5.20 (1H, d, *J* 12.0), 3.98 (1H, t, *J* 9.1), 3.84 to 3.65 (2H, m), 2.84-2.68 (3H, m), 2.55-2.53 (1H, m), 2.41-2.25 (2H, m), 2.12-1.92 (4H, m), 1.76 (1H, dd, *J* 13.9 & 6.1), 1.64 (3H, s), 1.20 (3H, s), 1.12 (1H, td, *J* 13.1 & 5.8); LC-MS *m/z* [M+H]$^+$ = 440.373.

*Synthesis of (3R,3aS,9aR,10aR,10bS,E)-6,9a-dimethyl-3-(((2-(trifluoromethoxy)benzyl)amino)methyl)-3a,4,5,8,9,9a,10a,10b-octahydrooxireno[2',3':9,10]cyclodeca[1,2-b]furan-2(3H)-one (**4x**)*

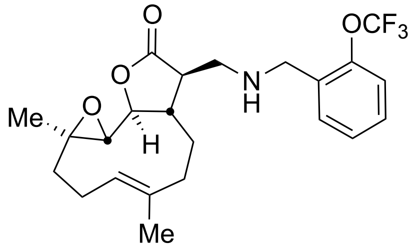

Following *General Procedure 2.* ^{1}H NMR (400 MHz, DMSO-d$_6$): δ 7.68-7.58 (1H, m), 7.46-7.28 (3H, m), 5.20 (1H, d, *J* 11.7), 3.99 (1H, t, *J* 9.1), 3.80 (2H, s), 2.89-2.68 (3H, m), 2.55 (1H, dd, *J* 8.2 & 4.0), 2.42-2.26 (2H, m), 2.18-1.92 (4H m), 1.81 (1H, dd, *J* 14.7 & 6.1), 1.72-1.64 (1H, m), 1.64 (3H, s), 1.20 (3H, s), 1.12 (1H, td, *J* 13.0 & 6.2); LC-MS *m/z* [M+H]$^+$ = 440.473.

*Synthesis of (3R,3aS,9aR,10aR,10bS,E)-6,9a-dimethyl-3-(((3-(trifluoromethoxy)benzyl)amino)methyl)-3a,4,5,8,9,9a,10a,10b-octahydrooxireno[2',3':9,10]cyclodeca[1,2-b]furan-2(3H)-one (**4y**)*

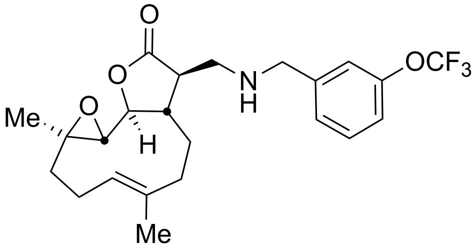

Following *General Procedure 2.* ^{1}H NMR (400 MHz, DMSO-d$_6$): δ 7.46 (1H, t, *J* 7.8), 7.41-7.24 (2H, m), 7.23 (1H, d, *J* 7.7), 5.19 (1H, d, *J* 12.1), 3.99 (1H, t, *J* 9.1), 3.83-3.72 (2H, m), 2.82-2.70 (3H, m), 2.57-2.53 (1H, m), 2.33 (2H, ddd, *J* 13.5 & 9.2 & 3.7), 2.14 to 1.92 (4H, m), 1.83 to 1.76 (1H, m), 1.64 (3H, s), 1.20 (3H, s), 1.12 (1H, td, *J* 12.6 & 5.7); LC-MS *m/z* [M+H]$^+$ = 440.423.

*Synthesis of (3R,3aS,9aR,10aR,10bS,E)-3-(((2,6-difluorobenzyl)amino)methyl)-6,9a-dimethyl-3a,4,5,8,9,9a,10a,10b-octahydrooxireno[2',3':9,10]cyclodeca[1,2-b]furan-2(3H)-one (**4z**)*

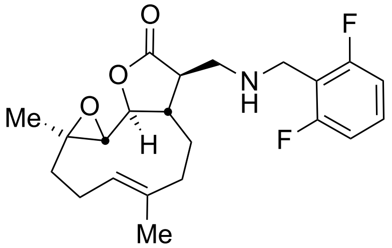

Following *General Procedure 2.* ^{1}H NMR (400 MHz, DMSO-d$_6$): δ 7.44-7.37 (1H, m, Ar*H*), 7.15-7.05 (2H, m, Ar*H*), 5.16 (1H, d, *J* 11.0, C*H*), 3.97 (1H, d, *J* 9.1), 3.87-3.70 (2H, m), 2.85-2.67 (3H, m), 2.41-1.71 (8H, m), 1.69-1.57 (1H, m), 1.63 (3H, s, Me), 1.19 (3H, s, Me), 1.09 (1H, td, *J* 12.8 & 5.8); LC-MS *m/z* [M+H]$^+$ = 392.462.

*Synthesis of 3R,3aS,9aR,10aR,10bS,E)-3-(((4-fluoro-2-(trifluoromethyl)benzyl)amino)methyl)-6,9a-dimethyl-3a,4,5,8,9,9a,10a,10b-octahydrooxireno[2',3':9,10]cyclodeca[1,2-b]furan-2(3H)-one (**4aa**)*

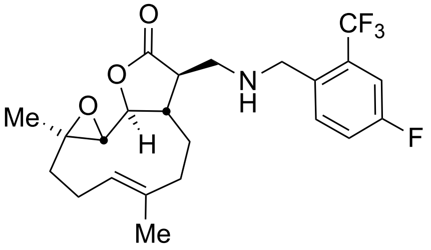

Following *General Procedure 2.* ^{1}H NMR (400 MHz, DMSO-d$_6$): δ 7.85 (1H, t, *J* 7.1), 7.63-7.53 (2H, m), 5.20 (1H d, *J* 11.9), 4.00 (1H, td, *J* 9.1 & 1.9) 3.87 (2H, s), 2.88-2.72 (3H, m), 2.56 (1H, s), 2.41-2.26 (2H, m), 2.15-1.92 (5H, m), 1.80 (1H, dd, *J* 14.5 & 6.2), 1.72-1.62 (1H, m), 1.64 (3H, s), 1.20 (3H, s), 1.12 (1H, td, *J* 12.5 & 5.4); LC-MS *m/z* [M+H]$^+$ = 442.373.

*Synthesis of (3R,3aS,9aR,10aR,10bS,E)-3-(((2-fluoro-6-(trifluoromethyl)benzyl)amino)methyl)-6,9a-Dimethyl-3a,4,5,8,9,9a,10a,10b-octahydrooxireno[2',3':9,10]cyclodeca[1,2-b]furan-2(3H)-one (**4ab**)*

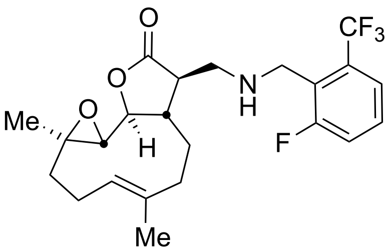

Following *General Procedure 2.* ^{1}H NMR (400 MHz, DMSO-d$_6$): δ 7.63-7.54 (3H, m) 5.16 (1H, d, *J* 11.1), 3.99 (1H, t, *J* 9,1), 3.87 (2H, q, *J* 13.1), 2.89 (1H, dd, *J* 12.4 & 4.2), 2.79 (1H, dd, *J* 12.7 & 4.6), 2.74 (1H, d, *J* 9.0), 2.57-2.53 (1H, m), 2.40-2.22 (2H, m), 2.15-1.97 (4H, m), 1.91 (1H, t, *J* 12.3), 1.80 (1H, dd, *J* 14.8 & 6.4), 1.72-1.62 (1H, m), 1.64 (3H, s), 1.19 (3H, s), 1.10 (1H, td, *J* 12.7 & 5.8); LC-MS *m/z* [M+H]$^+$ = 442.473.

*Synthesis of (3R,3aS,9aR,10aR,10bS,E)-3-(((2,4-dichlorobenzyl)amino)methyl)-6,9a-dimethyl-3a,4,5,8,9,9a,10a,10b-octahydrooxireno[2',3':9,10]cyclodeca[1,2-b]furan-2(3H)-one (**4ac**)*

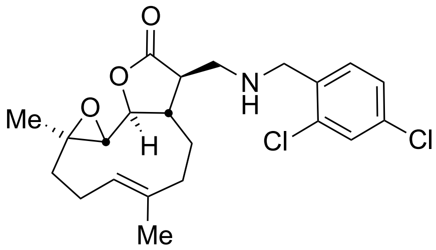

Following *General Procedure 2*. ^{1}H NMR (400 MHz, DMSO-d$_6$): δ 7.63-7.53 (2H, m), 7.44 (1H, dd, *J* 8.3 & 2.1), 5.19 (1H, d, *J* 11.8), 3.99 (1H, t, *J* 9.1), 3.79 (2H, s), 2.89-2.71 (3H, m), 2.57-2.52 (1H, m), 2.43-2.23 (3H, m), 2.17-1.92 (4H, m), 1.81 (1H, dd, *J* 14.8 & 6.2), 1.72-1.62 (1H, m), 1.64 (3H, s), 1.20 (3H, s), 1.11 (1H, td, *J* 12.8 & 5.1); LC-MS *m/z* [M+H]$^+$ = 424.37.

*Synthesis of (3R,3aS,9aR,10aR,10bS,E)-3-(((2-chloro-6-fluorobenzyl)amino)methyl)-6,9a-Dimethyl-3a,4,5,8,9,9a,10a,10b-octahydrooxireno[2',3':9,10]cyclodeca[1,2-b]furan-2(3H)-one (**4ad**)*

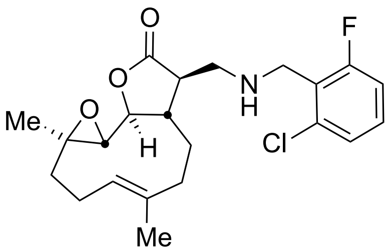

Following *General Procedure 2*. ^{1}H NMR (400 MHz, DMSO-d$_6$): δ 7.42-7.33 (2H, m), 7.27-7.22 (1H, m), 5.16 (1H, d, *J* 9.9), 3.98 (1H, t, *J* 9.1), 3.86 (2H, m), 2.85 (1H, dd, *J* 12.4 & 4.3), 2.79-2.69 (2H, m), 2.56-2.52 (1H, m), 2.29 (2H, m), 2.16-1.95 (3H, m), 1.89 (1H, t, *J* 12.3), 1.79 (1H, dd, *J* 14.8 & 6.3), 1.70-1.60 (1H, m), 1.63 (3H, s), 1.19 (3H, s), 1.25 (1H, tt, *J* 12.6 & 6.2); LC-MS *m/z* [M+H]$^+$ = 408.417.

*Synthesis of (3R,3aS,9aR,10aR,10bS,E)-3-(((2,3-dimethylbenzyl)amino)methyl)-6,9a-dimethyl-3a,4,5,8,9,9a,10a,10b-octahydrooxireno[2',3':9,10]cyclodeca[1,2-b]furan-2(3H)-one (**4ae**)*

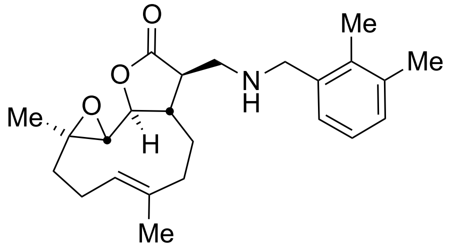

Following *General Procedure 2*. ^{1}H NMR (400 MHz, DMSO-d$_6$): δ 7.12 (1H, dd, *J* 6.8 & 2.2), 7.08-6.99 (2H, m), 5.16 (1H, d, *J* 11.7), 3.97 (1H, t, *J* 9.1), 3.74 (1H, d, *J* 13.2), 3.65 (1H, d, *J* 13.2), 2.88 (1H, dd, *J* 12.6 & 4.3), 2.80-2.70 (2H, m), 2.41-2.18 (2H, m), 2.22 (6H, d, *J* 12.0), 2.10-1.98 (4H, m), 1.88 (1H, t, *J* 13.3), 1.76 (1H, dd, *J* 8.5 & 5.6), 1.70-1.60 (1H, m), 1.63 (3H, s), 1.19 (3H, s), 1.11 (1H, td, *J* 12.8 & 5.8); LC-MS *m/z* [M+H]$^+$ = 383.413.

*Synthesis of (3R,3aS,9aR,10aR,10bS,E)-6,9a-dimethyl-3-(pyrrolidin-1-ylmethyl)-3a,4,5,8,9,9a,10a,10b-octahydrooxireno[2',3':9,10]cyclodeca[1,2-b]furan-2(3H)-one (**5a**)* [9,134-139]

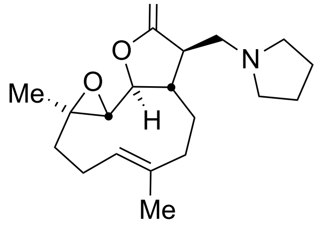

Following *General Procedure 1*, a mixture of parthenolide (100 mg, 0.40 mmol), pyrrolidine (0.48 mmol, 0.04 mL) and potassium carbonate (112 mg, 0.81 mmol), was stirred at room temperature in methanol (10 mL) for 24 hours. The title compound was obtained as a colourless solid in 85% yield (108 mg, 0.34 mmol). ^{1}H NMR (400 MHz, CDCl$_3$): δ 5.20 (1H, d, *J* 12.1 & 2.4, CH), 3.82 (1H, t, *J* 9.3, CH), 2.93 (1H, dd, *J* 13.1 & 4.8, CH$_2$), 2.89 (1H, dd, *J* 13.1 & 4.8, CH$_2$), 2.74 (1H, d, *J* 8.8, CH), 2.60-2.50 (4H, m), 2.45-2.01 (8H, m), 1.80-1.58 (8H, m), 1.31 (3H, s, Me), 1.23 (1H, m); ^{13}C NMR (101 MHz, CDCl$_3$): δ 17.0, 17.2, 23.8,

24.1, 30.0, 36.7, 41.1, 47.4, 47.6, 53.7, 54.8, 61.5, 66.6, 82.1, 125.0, 134.8, 176.6; IR (neat) cm^{-1} 1768 (C=O); mp: = 128-129 °C; $[\alpha]^{18}_{D}$ = -16.40° (c 1, $CHCl_3$); IR (neat, cm^{-1}): 1768 (C=O); MS (EI+) *m/z*: $[M+H]^+$ 320.50; HRMS (ES+) *m/z*: $[M+H]^+$ calcd. for $C_{19}H_{30}NO_3^+$, 320.2220; found: 320.2225.

*Synthesis of (3R,3aS,9aR,10aR,10bS,E)-6,9a-dimethyl-3-(piperidin-1-ylmethyl)-3a,4,5,8,9,9a,10a,10b-octahydrooxireno[2',3':9,10]cyclodeca[1,2-b]furan-2(3H)-one (**5b**)* [7a, 8b, 8c, 9-10]

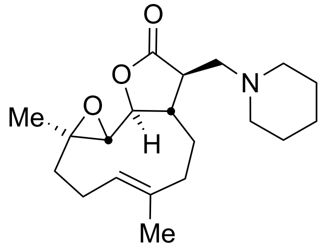

Following *General Procedure 1*, a mixture of parthenolide (100 mg, 0.40 mmol), piperidine (0.48 mmol, 0.047 mL) and potassium carbonate (112 mg, 0.81 mmol), was stirred at room temperature in methanol (10 mL) for 24 hours. The title compound was obtained as a colourless solid in 80% yield (106 mg, 0.32 mmol). ^{1}H NMR (400 MHz, $CDCl_3$): δ 5.21 (1H, dd, *J* 12.0 & 2.0, C*H*), 3.75 (1H, t, *J* 9, C*H*), 2.75 (2H, td, *J* 9.2 & 4.8, C*H*2), 2.60 (1H, dd, *J* 13.2 & 5.6, NC*H*2), 2.50-2.35 (6H, m), 2.30-2.05 (6H, m), 1.70 (3H, s, Me), 1.68-1.50 (5H, m), 1.48-1.38 (2H, m), 1.30 (3H, s, Me), 1.23 (1H, m); ^{13}C NMR (101 MHz, $CDCl_3$): δ 17.1, 17.2, 24.1, 14.2, 26.1, 30.1, 36.7, 41.2, 46.1, 48.2, 55.1, 57.5, 61.4, 66.7, 82.3, 124.7, 134.9, 176.8; mp: = 140-141 °C; $[\alpha]^{18}_{D}$ = -4.80° (c 1, $CHCl_3$); IR (neat, cm^{-1}): 1764 (C=O); MS (EI+) *m/z*: $[M+H]^+$ 334.33; HRMS (ES+) *m/z*: $[M+H]^+$ calcd. for $C_{20}H_{32}NO_3^+$, 334.2377; found: 322.2379.

*Synthesis of (3R,3aS,9aR,10aR,10bS,E)-6,9a-dimethyl-3-((4-methylpiperazin-1-yl)methyl)-3a,4,5,8,9,9a,10a,10b-octahydrooxireno[2',3':9,10]cyclodeca[1,2-b]furan-2(3H)-one (**5c**)* [8c, 10b]

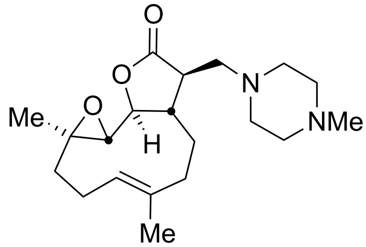

Following *General Procedure 1*, a mixture of parthenolide (100 mg, 0.40 mmol), 1-methylpiperazine (0.48 mmol, 0.05 mL) and potassium carbonate (112 mg, 0.81 mmol), was stirred at room temperature in methanol (10 mL) for 24 hours. The title compound was obtained as a colourless solid in 37% yield (51 mg, 0.15 mmol). ^{1}H NMR (400 MHz, $CDCl_3$): δ 5.20 (1H, dd, *J* 12.0 & 2.2, C*H*), 3.81 (1H, t, *J* 9.2, C*H*), 2.80 (1H, dd, *J* 13.6 & 4.8, NC*H*2), 2.75 (2H, dd, *J* 14.4 & 5.6, CH_2), 2.60-2.05 (18H, m), 1.80 (1H, s), 1.70 (3H, s, Me), 1.68-1.55 (1H, m), 1.28 (3H, s, Me), 1.27-1.16 (1H, m); ^{13}C NMR (101 MHz, $CDCl_3$): δ 17.1, 24.1, 30.1, 36.7, 41.2, 46.0, 46.3, 48.0, 53.8, 55.2, 56.4, 61.5, 66.7, 82.3, 124.9, 134.7, 176.5; mp: =163-164 °C; $[\alpha]^{18}_{D}$ = -2.01° (c 1, $CHCl_3$); IR (neat, cm^{-1}): 1769 (C=O); MS (EI+) *m/z*: $[M+H]^+$ 349.21.

*Synthesis of (3R,3aS,9aR,10aR,10bS,E)-6,9a-dimethyl-3-((4-(pyridin-3-yloxy)piperidin-1-yl)methyl)-3a,4,5,8,9,9a,10a,10b-octahydrooxireno[2',3':9,10]cyclodeca[1,2-b]furan-2(3H)-one (**5d**)*

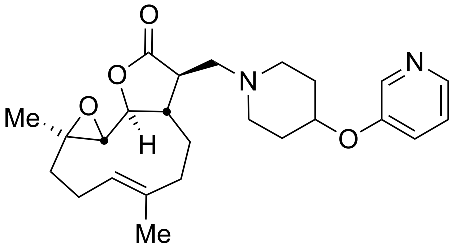

Following *General Procedure 2*. ^{1}H NMR (400 MHz, DMSO-d$_6$): δ 8.28 (1H, d, *J* 2.8, Ar*H*), 8.14 (1H, dd, *J* 4.6 & 1.3, Ar*H*), 7.42 (1H, ddd, *J* 8.5, 3.0 & 1.3, Ar*H*), 7.31 (1H, dd, *J* 8.9 & 5.1, Ar*H*), 5.22 (1H, d, *J* 13.4, C*H*), 4.52-4.39 (1H, m), 3.96 (1H, t, *J* 12.1), 2.84-2.75 (5H, m), 2.42-1.87 (10H, m), 1.70-1.55 (2H, m), 1.65 (3H, s, Me), 1.20 (3H, s, Me), 1.18-1.07 (1H, m); LC-MS *m/z* [M+H]$^+$ = 427.57.

*Synthesis of (3R,3aS,9aR,10aR,10bS,E)-3-((4-hydroxy-4-phenylpiperidin-1-yl)methyl)-6,9a-dimethyl-3a,4,5,8,9,9a,10a,10b-octahydrooxireno[2',3':9,10]cyclodeca[1,2-b]furan-2(3H)-one (**5e**)*

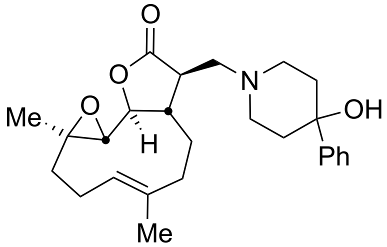

Following *General Procedure 2*. ^{1}H NMR (400 MHz, DMSO-d$_6$): δ 7.50-7.46 (2H, m, Ar*H*), 7.35-7.30 (2H, m, Ar*H*), 7.23-7.18 (1H, m, Ar*H*), 5.24 (1H, d, *J* 8.0, C*H*), 4.95-4.69 (1H, br, s), 3.98 (1H, t, *J* 9.0), 2.82 (1H, d, *J* 9.1), 2.80-2.53 (7H, m), 2.45-2.12 (3H, m), 2.11-1.82 (6H, m), 1.71-1.52 (3H, m), 1.66 (3H, s, Me), 1.21 (3H, s, Me), 1.15 (1H, td, *J* 12.9 & 6.1); LC-MS *m/z* [M+H]$^+$ = 426.47.

*Synthesis of (3R,3aS,9aR,10aR,10bS,E)-3-((4-(6-hydroxy-2-methylpyrimidin-4-yl)piperidin-1-yl)methyl)-6,9a-dimethyl-3a,4,5,8,9,9a,10a,10b-octahydrooxireno[2',3':9,10]cyclodeca[1,2-b]furan-2(3H)-one (**5f**)*

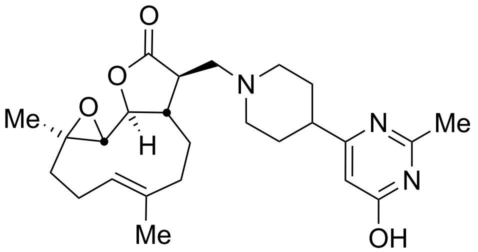

Following a procedure adapted from *General Procedure 1*, a mixture of parthenolide (0.500 g, 2.01 mmol), amine **7** (0.389 g, 2.00 mmol) and Hünig's base (0.520 g, 4.03 mmol), was stirred at room temperature in ethanol (20 mL) for 24 hours. The title compound was obtained as a colourless solid in 82% yield (0.714 g, 1.62 mmol). Rf = 0.30 (dichloromethane:methanol, 9:1), mp: 189-191 °C; $[\alpha]_D^{20}$ = 23.20 (c 1.6, $CHCl_3$); ^{1}H NMR (400 MHz, $CDCl_3$): δ 13.27 (1H, br s, OH), 6.17 (1H, s, ArH), 5.21 (1H, dd, *J* 12.0 & 2.2, CH), 3.84 (1H, t, *J* 9.0, CH), 3.00-2.90 (2H, m, CH_2), 2.83 (1H, dd, *J* 13.5 & 4.8, CH_2), 2.73 (1H, d, *J* 9.0, CH, epoxide moiety), 2.72 (1H, dd, *J* 13.5 & 5.6, CH_2), 2.47 (3H, s, Me), 2.47-2.35 (3H, m), 2.30-2.15 (6H, m), 2.14-2.03 (2H, m), 1.96-1.85 (2H, m, CH_2), 1.71 (3H, s, Me), 1.69-1.56 (3H, m), 1.30 (3H, s, Me), 1.24 (1H, td, *J* 12.9 & 5.9, CH_2); ^{13}C NMR (101 MHz, $CDCl_3$): δ 176.6 (C=O), 173.1 (C_q), 166.0 (C_q), 158.3 (C_q), 134.7 (C_q), 124.9 (CH), 107.4 (CH), 82.3 (CH), 66.7 (CH, epoxide), 61.5 (C_q), 56.7 (CH_2), 54.8 (CH_2), 53.9 (CH_2), 48.1 (CH), 46.4 (CH), 43.3 (CH), 41.2 (CH_2), 36.7 (CH_2), 30.8 (CH_2), 30.7 (CH_2), 30.1 (CH_2), 24.1 (CH_2), 21.7 (CH_3), 17.2 (CH_3), 17.0 (CH_3); IR (neat, cm^{-1}): 1659 (C=O); MS (ES+) *m/z*: [M+H]$^+$ = 442.523. HRMS (ES+) *m/z*: [M+H]$^+$ calcd. for $C_{25}H_{36}N_3O_4^+$: 442.2700, found: 442.2712. Compound **5f** was preliminarily prepared by *General Procedure 2* and it is from that preparation that the NMR

spectrum in DMSO-d_6 and the low res. ES+ mass spectrum were obtained, other data were consistent between batches obtained *via* both procedures.

*Synthesis of (3R,3aS,9aR,10aR,10bS,E)-3-((1,4-dioxa-8-azaspiro[4.5]decan-8-yl)methyl)-6,9a-dimethyl-3a,4,5,8,9,9a,10a,10b-octahydrooxireno[2',3':9,10]cyclodeca[1,2-b]furan-2(3H)-one (**5g**)*

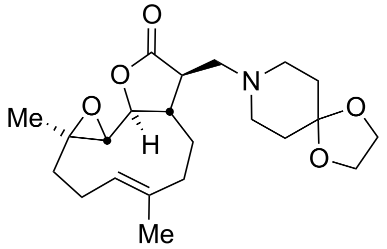

Following *General Procedure 2*. ^{1}H NMR (400 MHz, DMSO-d_6): δ 5.22 (1H, d, *J* 8.0, C*H*), 3.96 (1H, t, *J* 9.1), 3.85 (2H, s), 2.80 (1H, d, *J* 8.0), 2.73-1.86 (17H, m), 1.64 (3H, s, Me), 1.60 (4H, t, *J* 5.6), 1.20 (3H, s, Me), 1.13 (1H, td, *J* 12.0 & 8.0); LC-MS *m/z* [M+H]$^+$ = 392.414.

*Synthesis of 4-(1-(((3R,3aS,9aR,10aR,10bS,E)-6,9a-dimethyl-2-oxo-2,3,3a,4,5,8,9,9a,10a,10b-decahydrooxireno[2',3':9,10]cyclodeca[1,2-b]furan-3-yl)methyl)piperidin-4-yl)benzamide (**5h**)*

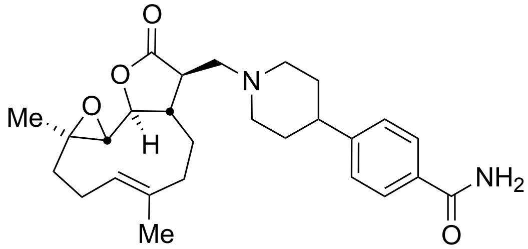

Following *General Procedure 2*. ^{1}H NMR (400 MHz, DMSO-d_6): δ 7.90 (1H, br, N*H*), 7.80 (2H, d, *J* 8.3, Ar*H*), 7.30 (2H, d, *J* 8.3, Ar*H*), 7.27 (1H, br, N*H*), 5.24 (1H, dd, *J* 13.0 & 3.5, C*H*), 3.98 (1H, t, *J* 9.1), 3.00-2.78 (4H, m), 2.74-2.57 (4H, m), 2.41-2.12 (4H, m), 2.11-1.97 (5H, m), 1.81-1.72 (2H, m), 1.71-1.60 (5H, m), 1.21 (3H, s, Me), 1.14 (1H, td, *J* 12.6 & 5.1); LC-MS *m/z* [M+H]$^+$ = 453.525.

*Synthesis of (3R,3aS,9aR,10aR,10bS,E)-6,9a-dimethyl-3-((4-propylpiperidin-1-yl)methyl)-3a,4,5,8,9,9a,10a,10b-octahydrooxireno[2',3':9,10]cyclodeca[1,2-b]furan-2(3H)-one (**5i**)*

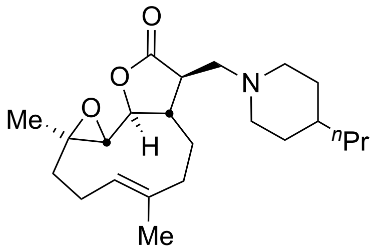

Following *General Procedure 2*. ^{1}H NMR (400 MHz, DMSO-d_6):): δ 5.21 (1H, d, *J* 11.7, C*H*), 4.07 (2H, q, *J* 8.0, CH_2), 3.96 (1H, t, *J* 9.2), 2.87-2.53 (6H, m), 2.43-1.85 (8H, m), 1.70-1.52 (2H, m), 1.65 (3H, s, Me), 1.35-0.99 (8H, m), 1.20 (3H, s, Me), 0.86 (3H, t, *J* 7.1, Me); LC-MS *m/z* [M+H]$^+$ = 376.411.

*Synthesis of (3R,3aS,9aR,10aR,10bS,E)-3-((4-(2-methoxyethyl)piperazin-1-yl)methyl)-6,9a-dimethyl-3a,4,5,8,9,9a,10a,10b-octahydrooxireno[2',3':9,10]cyclodeca[1,2-b]furan-2(3H)-one (**5j**)*

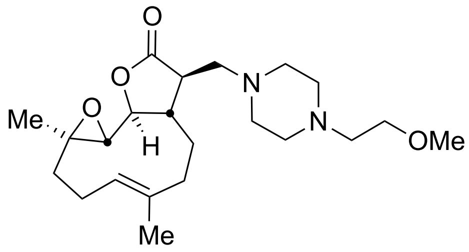

Following *General Procedure 2*. ^{1}H NMR (400 MHz, DMSO-d_6): δ 5.18 (1H, dd, *J* 9.4 & 2.4, C*H*), 3.83 (1H, t, *J* 9.0), 3.68 (2H, br), 3.35 (3H, s, Me), 2.94-2.65 (10H, m), 2.51-1.95 (9H, m), 1.69 (3H, s, Me), 1.67-1.57 (1H, m), 1.29 (3H, s, Me), 1.22 (3H, d, *J* 12.8 & 5.9, Me); LC-MS *m/z* [M+H]$^+$ = 393.3.

*Synthesis of (3R,3aS,9aR,10aR,10bS,E)-3-((4-(3,4-dihydroisoquinolin-2(1H)-yl)piperidin-1-yl)methyl)-6,9a-dimethyl-3a,4,5,8,9,9a,10a,10b-octahydrooxireno[2',3':9,10]cyclodeca[1,2-b]furan-2(3H)-one (**5k**)*

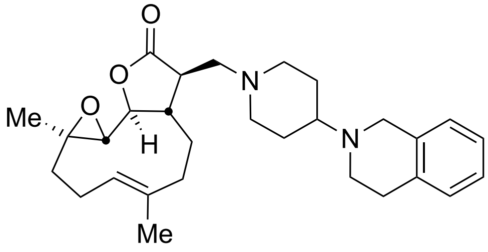

Following *General Procedure 2*. ^{1}H NMR (400 MHz, DMSO-d$_6$): δ 7.35-7.15 (4H, m, Ar*H*), 5.18 (1H, d, *J* 11.7), 4.55-4.32 (2H, m), 4.15-3.91 (1H, m), 3.80-3.50 (3H, m), 3.30-3.0 (5H, m), 2.80 (1H, d, *J* 9.1), 2.50-1.95 (11H, m), 1.75-1.60 (1H, m), 1.67 (3H, s, Me), 1.30-1.05 (1H, m), 1.22 (3H, s, Me) ; LC-MS *m/z* [M+H]$^+$ = 465.528.

*Synthesis of ethyl 4-(((3R,3aS,9aR,10aR,10bS,E)-6,9a-dimethyl-2-oxo-2,3,3a,4,5,8,9,9a,10a,10b-decahydrooxireno[2',3':9,10]cyclodeca[1,2-b]furan-3-yl)methyl)piperazine-1-carboxylate (**5l**)*

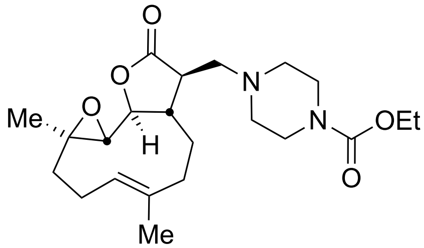

Following *General Procedure 2*. ^{1}H NMR (400 MHz, DMSO-d$_6$): δ 5.21 (1H, d, *J* 8.0, C*H*), 4.08-3.92 (3H, m), 2.81 (1H, d, *J* 9.1), 2.73-2.56 (3H, m), 2.46-1.88 (11H, m), 1.73-1.55 (1H, m), 1.64 (3H, s, Me), 1.20 (3H, s, Me), 1.18 (3H, t, *J* 7.1, Me), 1.15-1.07 (1H, m); LC-MS *m/z* [M+H]$^+$ = 407.385.

*Synthesis of (3R,3aS,9aR,10aR,10bS,E)-6,9a-dimethyl-3-((4-(pyrimidin-2-yl)piperazin-1-yl)methyl)-3a,4,5,8,9,9a,10a,10b-octahydrooxireno[2',3':9,10]cyclodeca[1,2-b]furan-2(3H)-one (**5m**)*

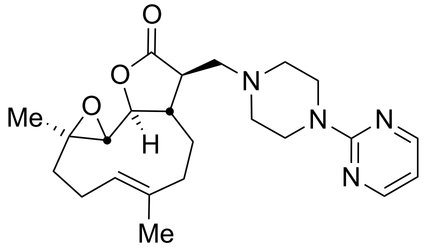

Following *General Procedure 2*. ^{1}H NMR (400 MHz, DMSO-d$_6$): δ 8.36 (2H, d, *J* 4.0, Ar*H*), 6.63 (1H, t, *J* 4.0, Ar*H*), 5.24 (1H, d, *J* 12.0, C*H*), 3.98 (1H, t, *J* 12.1), 3.77-3.64 (4H, m), 2.82 (1H, d, *J* 8.0), 2.77-2.59 (3H, m), 2.57-2.46 (2H, m), 2.47-1.94 (9H, m), 1.74-1.55 (1H, m), 1.66 (3H, s, Me), 1.20 (3H, s, Me), 1.13 (1H, td, *J* 12.1 & 4.0); LC-MS *m/z* [M]$^+$ = 413.468.

*Synthesis of (3R,3aS,9aR,10aR,10bS,E)-3-((4-(2-chloro-4-(methylsulfonyl)phenyl)piperazin-1-yl)methyl)-6,9a-dimethyl-3a,4,5,8,9,9a,10a,10b-octahydrooxireno[2',3':9,10]cyclodeca[1,2-b]furan-2(3H)-one (**5n**)*

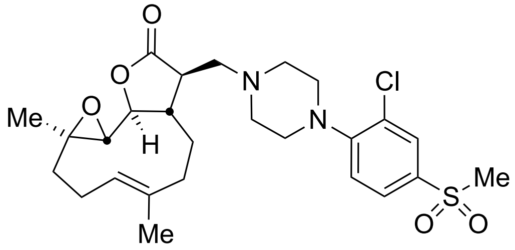

Following *General Procedure 2*. ^{1}H NMR (400 MHz, DMSO-d$_6$): δ 7.84 (1H, d, *J* 2.6, Ar*H*), 7.79 (1H, dd, *J* 8.5 & 2.6, Ar*H*), 7.66 (1H, d, *J* 8.5, Ar*H*), 5.23 (1H, d, *J* 9.8, C*H*), 3.99 (1H, t, *J* 9.1), 3.43 (3H, s, Me), 3.05-2.54 (9H, m), 2.43-2.12 (4H, m), 2.11-1.94 (6H, m), 1.73-1.58 (1H, m), 1.66 (3H, s, Me), 1.21 (3H, t, *J* 7.1, Me), 1.14 (1H, td, *J* 12.7 & 6.0); LC-MS *m/z* [M+H]$^+$ = 523.389.

*Synthesis of 4-(((3R,3aS,9aR,10aR,10bS,E)-6,9a-dimethyl-2-oxo-2,3,3a,4,5,8,9,9a,10a,10b-decahydrooxireno[2',3':9,10]cyclodeca[1,2-b]furan-3-yl)methyl)-1-(3-methylbenzyl)piperazin-2-one (**5o**)*

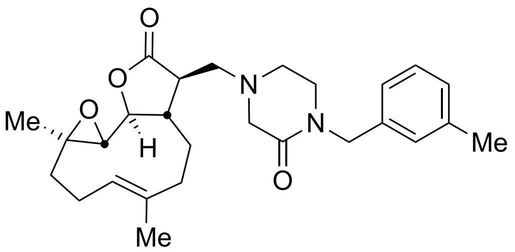

Following *General Procedure 2*. ^{1}H NMR (400 MHz, DMSO-d$_6$): δ 7.56 (1H, t, *J* 8.0, Ar*H*), 7.29 (1H, s), 7.08 (1H, d, *J* 8.0, Ar*H*), 7.03-6.98 (1H, m, Ar*H*), 5.19 (1H, d, *J* 10.1, C*H*), 4.48 (2H, s), 3.96 (1H, t, *J* 9.1), 3.22-3.09 (4H, m), 2.83-2.54 (6H, m), 2.41-2.17 (2H, m), 2.28 (3H, s, Me), 2.11-1.85 (5H, m), 1.71-1.50 (1H, m), 1.63 (3H, s, Me), 1.18 (3H, s, Me), 1.10 (1H, td, *J* 13.0 & 5.8); LC-MS *m/z* [M+H]$^+$ = 453.641.

*Synthesis of 1-(((3R,3aS,9aR,10aR,10bS,E)-6,9a-dimethyl-2-oxo-2,3,3a,4,5,8,9,9a,10a,10b-decahydrooxireno[2',3':9,10]cyclodeca[1,2-b]furan-3-yl)methyl)-4-phenylpiperidine-4-carbonitrile (**5p**)*

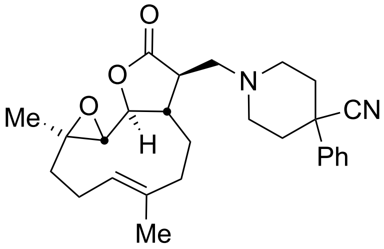

Following *General Procedure 2*. ^{1}H NMR (400 MHz, DMSO-d$_6$): δ 7.56-7.51 (2H, m, Ar*H*), 7.48-7.42 (2H, m, Ar*H*), 7.40-7.34 (1H, m, Ar*H*), 5.22 (1H, d, *J* 8.0, C*H*), 3.98 (1H, t, *J* 9.1), 3.03-2.58 (6H, m), 2.47-2.21 (4H, m), 2.20-1.86 (9H, m), 1.71-1.58 (1H, m), 1.64 (3H, s, Me), 1.19 (3H, s, Me), 1.12 (1H, td, *J* 12.6 & 6.0); LC-MS *m/z* [M+H]$^+$ = 435.522.

*Synthesis of (3R,3aS,9aR,10aR,10bS,E)-3-((3-(4-fluorophenoxy)azetidin-1-yl)methyl)-6,9a-dimethyl-3a,4,5,8,9,9a,10a,10b-octahydrooxireno[2',3':9,10]cyclodeca[1,2-b]furan-2(3H)-one (**5q**)*

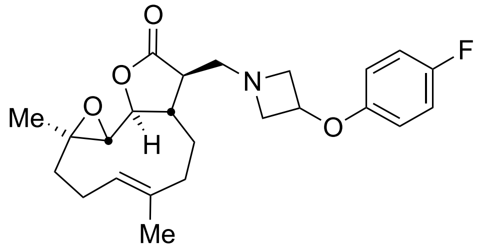

Following *General Procedure 2*. ^{1}H NMR (400 MHz, DMSO-d$_6$): δ 7.14-7.06 (2H, m, Ar*H*), 6.88-6.80 (2H, m, Ar*H*), 5.23 (1H, d, *J* 8.0, C*H*), 4.79-4.70 (1H, m), 3.95 (1H, t, *J* 9.1), 3.90-3.71 (2H, m), 3.12-3.02 (2H, m), 2.87-2.77 (3H, m), 2.47-2.11 (4H, m), 2.06-1.91 (3H, m), 1.90-1.81 (1H, m), 1.71-1.57 (1H, m), 1.64 (3H, s, Me), 1.19 (3H, s, Me), 1.12 (1H, td, *J* 12.6 & 6.0); LC-MS *m/z* [M+H]$^+$ = 416.468.

*Synthesis of (3R,3aS,9aR,10aR,10bS,E)-3-([1,4'-bipiperidin]-1'-ylmethyl)-6,9a-dimethyl-3a,4,5,8,9,9a,10a,10b-octahydrooxireno[2',3':9,10]cyclodeca[1,2-b]furan-2(3H)-one (**5r**)*

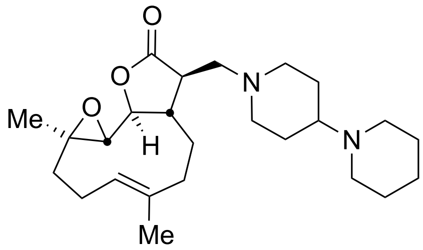

Following *General Procedure 2*. ^{1}H NMR (400 MHz, DMSO-d$_6$): δ 8.17 (1H, s), 5.21 (1H, d, *J* 12.0), 3.95 (1H, t, *J* 9.2), 2.90-2.75 (3H, m), 2.68-2.52 (5H, m), 2.41-1.87 (9H, m), 1.73 (1H, d, *J* 11.7), 1.64 (3H, s), 1.56-1.33 (5H, m), 1.19 (4H, s), 1.12 (1H, dt, *J* 12.7 & 6.7); LC-MS *m/z* [M+H]$^+$ = 417.518.

*Synthesis of (3R,3aS,9aR,10aR,10bS,E)-3-((4-(tert-butyl)piperazin-1-yl)methyl)-6,9a-dmethyl-3a,4,5,8,9,9a,10a,10b-octahydrooxireno[2',3':9,10]cyclodeca[1,2-b]furan-2(3H)-one (**5s**)*

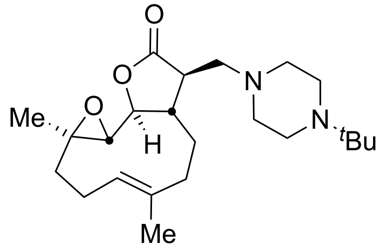

Following *General Procedure 2.* ^{1}H NMR (400 MHz, DMSO-d$_6$): δ 5.21 (1H, d, *J* 11.0), 3.97 (1H, d, *J* 9.1), 2.79 (1H, d, *J* 9.1), 2.68-2.54 (5H, m), 2.49-2.27 (4H, m), 2.27-1.93 (5H, m), 1.67-1.56 (1H, m), 1.65 (3H, s), 1.20 (3H, s), 1.12 (1H, td, *J* 12.2 & 6.1), 1.02 (9H, s); LC-MS *m/z* [M+H]$^+$ = 391.514.

*Synthesis of (3R,3aS,9aR,10aR,10bS,E)-6,9a-dimethyl-3-((4-(2-(thiophen-2-yl)ethyl)piperazin-1-yl)methyl)-3a,4,5,8,9,9a,10a,10b-octahydrooxireno[2',3':9,10]cyclodeca[1,2-b]furan-2(3H)-one (**5t**)*

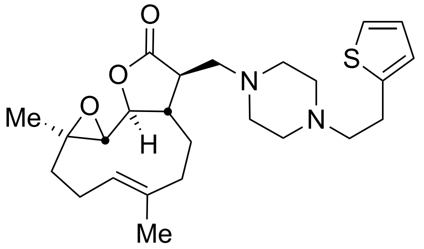

Following *General Procedure 2.* ^{1}H NMR (400 MHz, DMSO-d$_6$): δ 7.30 (1H, dd, *J* 5.1 & 1.3), 6.93 (1H, dd, *J* 5.1 & 3.4), 6.89-6.86 (2H, m), 5.20 (1H, d, *J* 11.8), 3.97 (1H, t, *J* 9.1), 2.95 (2H, t, *J* 7.4), 2.79 (1H, d, *J* 9.1), 2.74-2.53 (4H, m), 2.50-2.32 (10H, m), 2.30-1.95 (5H, m), 1.66-1.56 (1H, m), 1.65 (3H, s), 1.20 (3H, s), 1.12 (1H, d, *J* 5.9); LC-MS *m/z* [M+H]$^+$ = 445.474.

*Synthesis of (3R,3aS,9aR,10aR,10bS,E)-3-((4-(cyclohexylmethyl)piperazin-1-yl)methyl)-6,9a-dimethyl-3a,4,5,8,9,9a,10a,10b-octahydrooxireno[2',3':9,10]cyclodeca[1,2-b]furan-2(3H)-one (**5u**)*

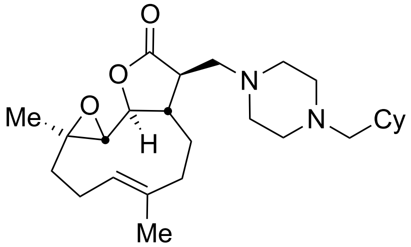

Following *General Procedure 2.* ^{1}H NMR (400 MHz, DMSO-d$_6$): δ 5.20 (1H, d, *J* 11.0), 3.96 (1H, t, *J* 9.1), 2.78 (1H, d, *J* 9.1), 2.71-2.54 (3H, m), 2.46-2.18 (10H, m), 2.17-1.85 (7H, m), 1.74-1.51 (6H, m), 1.64 (3H, s, Me), 1.53-1.36 (1H, m), 1.25-1.06 (4H, m), 1.20 (3H, s, Me), 0.88-0.73 (2H, m); LC-MS *m/z* [M+H]$^+$ = 431.571.

*Synthesis of (3R,3aS,9aR,10aR,10bS,E)-6,9a-dimethyl-3-((3-(trifluoromethyl)-5,6-dihydro-[1,2,4]triazolo[4,3-a]pyrazin-7(8H)-yl)methyl)-3a,4,5,8,9,9a,10a,10b-octahydrooxireno[2',3':9,10]cyclodeca[1,2-b]furan-2(3H)-one (**5v**)*

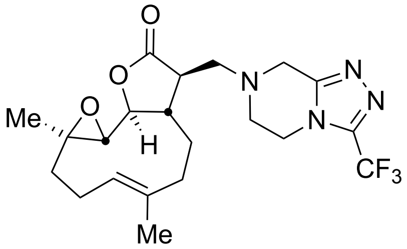

Following *General Procedure 2.* ^{1}H NMR (400 MHz, DMSO-d$_6$): δ 5.22 (1H, d, *J* 11.7), 4.18 (1H, t, *J* 5.5), 4.00 (1H, t, *J* 9.1), 3.95 (1H, s), 3.59 (1H, dd, *J* 8.7 & 3.4), 3.29 (1H, s), 3.05-2.87 (3H, m), 2.85 (1H, d, *J* 9.3), 2.81-2.70 (2H, m), 2.41-2.24 (2H, m), 2.16-1.82 (5H, m), 1.73-1.65 (1H, m), 1.64 (3H, s), 1.21 (3H, s), 1.12 (1H, td, *J* 13.0 & 5.6); LC-MS *m/z* [M+H]$^+$ = 441.373.

*Synthesis of (3R,3aS,9aR,10aR,10bS,E)-3-((4-(3-chlorophenyl)piperidin-1-yl)methyl)-6,9a-dimethyl-3a,4,5,8,9,9a,10a,10b-octahydrooxireno[2',3':9,10]cyclodeca[1,2-b]furan-2(3H)-one (**5w**)*

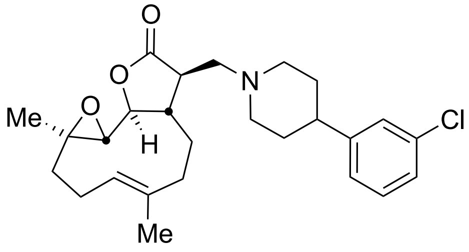

Following *General Procedure 2*. ^{1}H NMR (400 MHz, DMSO-d$_6$): δ 7.37-7.20 (4H, m), 5.24 (1H, d, *J* 11.4), 3.98 (1H, t, *J* 9.1), 2.96 (1H, d, *J* 11.4), 2.87 (1H, d, *J* 11.5), 2.82 (1H, d, *J* 9.1), 2.75-2.52 (1H, m), 2.39-1.95 (4H, m), 1.76 (2H, d, *J* 13.0), 1.66 (3H, s), 1.67-1.55 (1H, m), 1.21 (3H, s), 1.14 (1H, td, *J* 12.9 & 5.7); LC-MS [M + H]$^+$ = 444.473.

*Synthesis of (3R,3aS,9aR,10aR,10bS,E)-3-((4-butylpiperazin-1-yl)methyl)-6,9a-dimethyl-3a,4,5,8,9,9a,10a,10b-octahydrooxireno[2',3':9,10]cyclodeca[1,2-b]furan-2(3H)-one (**5x**)*

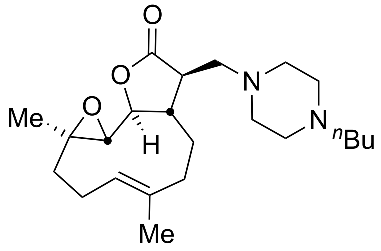

Following *General Procedure 2*. ^{1}H NMR (400 MHz, DMSO-d$_6$): δ 5.21 (1H, d, *J* 10.4), 3.96 (1H, t, *J* 9.1), 2.78 (1H, d, *J* 9.1), 2.69-2.55 (3H, m), 2.46-2.29 (8H, m), 2.26 (2H, t, *J* 6.9), 2.22-1.92 (6H, m), 1.67-1.56 (1H, m), 1.65 (3H, s), 1.39 (2H, tt, *J* 7.6 & 6.0), 1.33-1.24 (2H, m), 1.20 (3H, s), 1.13 (1H, td, *J* 13.3 & 5.6), 0.88 (3H, t, *J* 7.3); LC-MS *m/z* [M+H]$^+$ = 391.464.

*Synthesis of (3R,3aS,9aR,10aR,10bS,E)-6,9a-dimethyl-3-((4-(p-tolyloxy)piperidin-1-yl)methyl)-3a,4,5,8,9,9a,10a,10b-octahydrooxireno[2',3':9,10]cyclodeca[1,2-b]furan-2(3H)-one (**5y**)*

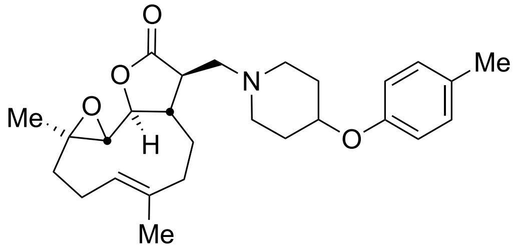

Following *General Procedure 2*. ^{1}H NMR (400 MHz, DMSO-d$_6$): δ 7.06 (2H, d, *J* 8.5), 6.83 (2H, dt, *J* 8.6 & 2.9), 5.23 (1H, d, *J* 11.2), 4.31 (1H, tt, *J* 8.0 & 3.7), 3.97 (1H, t, *J* 9.1), 2.81 (1H, d, *J* 9.1), 2.78-2.54 (3H, m), 2.41-2.12 (8H, m), 2.11-1.86 (7H, m), 1.70-1.52 (6H, m), 1.20 (3H, s), 1.14 (1H, td, *J* 12.7 & 5.8); LC-MS *m/z* [M+H]$^+$ = 440.473.

*Synthesis of (3R,3aS,9aR,10aR,10bS,E)-3-((4-(4-fluorophenyl)piperidin-1-yl)methyl)-6,9a-dimethyl-3a,4,5,8,9,9a,10a,10b-octahydrooxireno[2',3':9,10]cyclodeca[1,2-b]furan-2(3H)-one (**5z**)*

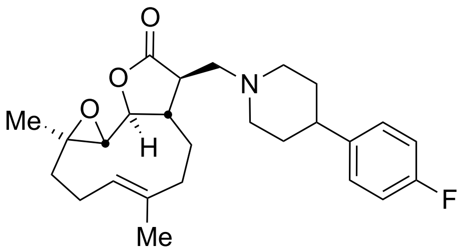

Following *General Procedure 2*. ^{1}H NMR (400 MHz, DMSO-d$_6$): δ 7.28 (2H, dd, *J* 8.6 & 5.7), 7.11 (2H, t, *J* 8.9), 5.24 (1H, d, *J* 12.0), 3.98 (1H, t, *J* 9.1), 2.91 (2H, m), 2.82 (1H, d, *J* 9.1), 2.75-2.58 (3H, m), 2.40-1.93 (4H, m), 1.74 (2H, d, *J* 12.5), 1.66 (3H, s), 1.65-1.53 (1H, m), 1.21 (3H, s), 1.14 (1H, td, *J* 12.7 & 6.2); LC-MS *m/z* [M+H]$^+$ = 428.52.

*Synthesis of (3R,3aS,9aR,10aR,10bS,E)-3-((4-(3-methoxybenzoyl)piperazin-1-yl)methyl)-6,9a-dimethyl-3a,4,5,8,9,9a,10a,10b-octahydrooxireno[2',3':9,10]cyclodeca[1,2-b]furan-2(3H)-one (**5aa**)*

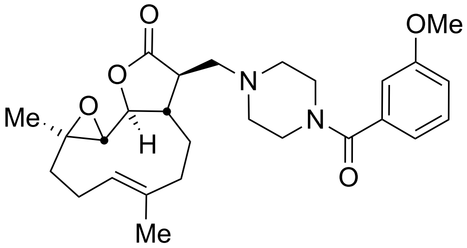

Following *General Procedure 2.* ^{1}H NMR (400 MHz, DMSO-d$_6$): δ 7.36 (1H, t, *J* 7.9), 7.02 (1H, dd, *J* 8.0 & 2.0), 6.93 (2H, d, *J* 10.7), 5.22 (1H, d, *J* 11.2), 3.97 (1H, t, *J* 9.1), 3.78 (3H, s), 3.60 (2H, s), 2.81 (1H, d, *J* 9.1), 2.68 (3H, m), 2.56-2.09 (7H, m), 2.08 (1H, s), 2.08-1.90 (5H, m), 1.65 (4H, s), 1.20 (3H, s), 1.12 (1H, td, *J* 12.4 & 5.6); LC-MS *m/z* [M+H]$^+$ = 469.478.

*Synthesis of (3R,3aS,9aR,10aR,10bS,E)-6,9a-dimethyl-3-((4-(pyridin-3-ylmethoxy)piperidin-1-yl)methyl)-3a,4,5,8,9,9a,10a,10b-octahydrooxireno[2',3':9,10]cyclodeca[1,2-b]furan-2(3H)-one (**5ab**)*

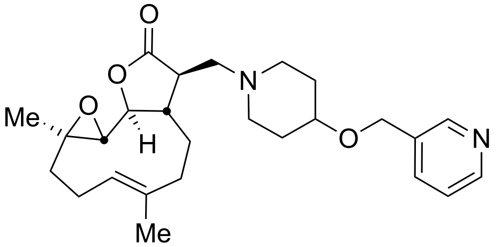

Following *General Procedure 2.* ^{1}H NMR (400 MHz, DMSO-d$_6$): δ 8.53 (1H, d, *J* 4.0, Ar*H*), 8.49 (1H, dd, *J* 4.8 & 1.7, Ar*H*), 7.76-7.71 (1H, m, Ar*H*), 7.40-7.35 (1H, m, Ar*H*), 5.22 (1H, d, *J* 6.0, C*H*), 4.53 (2H, s), 3.95 (1H, t, *J* 9.1), 3.46to 3.35 (1H, m), 2.84-2.53 (5H, m), 2.42-1.75 (12H, m), 1.70-1.38 (3H, m), 1.63 (3H, s, Me), 1.18 (3H, s, Me), 1.12 (1H, td, *J* 12.6 & 6.1); LC-MS *m/z* [M+H]$^+$ = 441.573.

*Synthesis of (((3R,3aS,9aR,10aR,10bS,E)-6,9a-dimethyl-2-oxo-2,3,3a,4,5,8,9,9a,10a,10b-decahydrooxireno[2',3':9,10]cyclodeca[1,2-b]furan-3-yl)methyl)glycine (**6a**)* [11]

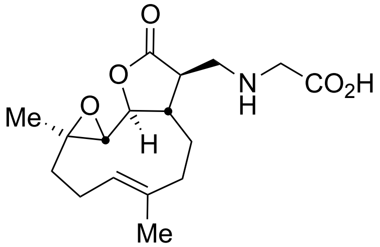

Following *General Procedure 1* (using a 1:1 mixture of methanol and water as solvent), a mixture of parthenolide (143 mg, 0.58 mmol), glycine (0.48 mmol, 36 mg) and potassium carbonate (133 mg, 0.96 mmol), was stirred at room temperature in methanol and water mixture (10 mL) for 24 hours. The title compound was obtained as a colourless crystalline solid in 23% yield (36 mg, 0.11 mmol). ^{1}H NMR (400 MHz, DMSO-d$_6$): .25 (1H, dd, *J* 12.0 & 2.2, C*H*), 3.80 (1H, t, *J* 9.2, C*H*), 2.78 (4H, m), 2.55-1.85 (9H, m), 1.65 (3H, s, Me), 1.70-1.60 (1H, m), 1.20 (3H, s, Me), 1.15-1.05 (1H, m); ^{13}C NMR (101 MHz, DMSO-d$_6$): δ 16.6, 16.8, 23.7, 28.9, 36.1, 40.5, 45.8, 47.1, 47.6, 55.0, 61.1, 65.5, 81.5, 124.4, 134.4, 173.1, 176.8; mp: = 164-165 °C; $[\alpha]^{18}_{D}$ = -27.60° (c 1, water); IR (neat, cm^{-1}): 3339 (OH), 2976 (NH), 1578 and 1757 (C=O); MS (EI+) *m/z*: [M+Na]$^+$ 346.24; HRMS (ES+) *m/z*: [M+Na]$^+$ calcd. for $C_{17}H_{25}NNaO_5^+$, 346.1625; found: 346.1642.

*Synthesis of (((3R,3aS,9aR,10aR,10bS,E)-6,9a-dimethyl-2-oxo-2,3,3a,4,5,8,9,9a,10a,10b-decahydrooxireno[2',3':9,10]cyclodeca[1,2-b]furan-3-yl)methyl)-L-phenylalanine (**6b**)*

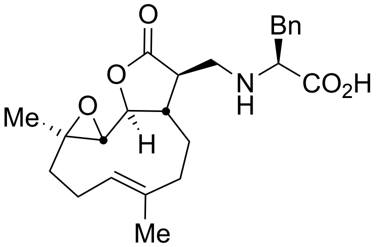

Following *General Procedure 1* (using a 1:1 mixture of methanol and water as solvent), a mixture of parthenolide (143 mg, 0.58 mmol), L-phenylalanine (0.48 mmol, 80 mg) and potassium carbonate (133 mg, 0.96 mmol), was stirred at room temperature in methanol and water mixture (10 mL) for 24 hours. The title compound was obtained as a colourless crystalline solid in 28% yield (56 mg, 0.14 mmol). ^{1}H NMR (400 MHz, DMSO-d$_6$): δ 7.25 (5H, m, Ph), 5.25 (1H, dd, *J* 12.0 & 2.0, C*H*), 3.90 (1H, t, *J* 9.2, C*H*), 3.45 (2H, m), 2.9 (2H, m), 2.8 (1H, dd, *J* 13.6 & 4.8, NC*H*$_2$), 2.69-2.60 (2H, m), 2.55-2.05 (5H, m), 1.90 (1H, t, *J* 12.1, C*H*$_2$), 1.75 (1H, dd, *J* 14.8 & 5.6, C*H*$_2$), 1.70-1.59 (1H, m), 1.60 (3H, s, Me), 1.20 (3H, s, Me), 1.15-1.05 (1H, m); ^{13}C NMR (101 MHz, DMSO-d$_6$): δ 16.6, 16.8, 23.6, 28.8, 36.1, 38.6, 38.9, 39.1, 44.8, 45.5, 47.4, 61.0, 62.9, 65.4, 81.6, 124.3, 126.1, 128.0, 129.2, 134.4, 138.5, 148.0, 175.1, 176.8; mp: = 150-152 °C; $[\alpha]^{18}_D$ = -14.05° (c 1, water); IR (neat, cm^{-1}): 3427 (OH), 2978 (NH), 1600 and 1762 (C=O); MS (EI+) *m/z*: [M+H]$^+$ 414.22; HRMS (ES+) *m/z*: [M+H]$^+$ calcd. for $C_{24}H_{32}NO_5^+$, 414.2275; found: 414.2283.

*Synthesis of 2-methyl-6-(piperidin-4-yl)pyrimidin-4-ol (**7**)*

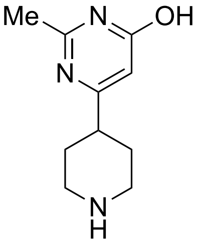

In an ice slush bath-cooled flask, compound **10** (detailed below) was dissolved in dichloromethane (3.0 mL) and treated with trifluoroacetic acid (3.0 mL, 0.04 mmol) which was added dropwise over six hours. The solvent was removed *in vacuo* and ethyl acetate (5.0 mL) was added resulting in the formation of a white solid which was collected by filtration and washed with chloroform (in which the material was not soluble). The material thus obtained was dried *in vacuo* to furnish compound **7** as a white solid (77 mg, 99%). ^{1}H NMR (400 MHz, Methanol-*d*$_4$) δ 6.19 (1H, s, Ar C*H*), 3.51 (2H, dt, *J* 11.9 & 3.0), 3.12 (2H, td, *J* 13.0 & 3.1), 2.80 (1H, tt, *J* 11.7 & 3.6, (CH$_2$)$_2$C*H*-Ar), 2.43-2.35 (2H, m), 2.13 (1H, s), 2.10 (1H, s), 2.00-1.87 (2H, m); ^{13}C NMR (101 MHz, Methanol-*d*$_4$) δ 169.2, 164.4, 161.3, 159.7, 107.9, 43.5, 40.2, 26.8, 19.9, 19.7. HRMS (ES+) *m/z*: [M+H]$^+$ calcd. for $C_{10}H_{16}N_3O^+$: 194.1288, found: 194.1292.

*Synthesis of N-Boc-piperidine-4-carboxylic acid (**8**)* [12]

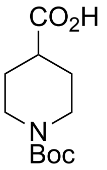

To an ice slush bath-cooled aqueous solution of sodium hydroxide (50 mL, 1 M) a solution of piperidine-4-carboxylic acid (6.46 g, 50 mmol) in THF (30 mL) was added dropwise. To which, a solution of di-*tert*-butyl carbamate (19.10 g, 87.5 mmol) in THF (30 mL) was next added, dropwise. After stirring for 30 min, the solution was allowed to warm to RT and was stirred for ~18 h for convenience. The resulting mixture was concentrated *in vacuo* to half its original volume. The resulting mixture was adjusted to pH 5-6 (universal indicator paper) by addition of aqueous HCl (1 M). This resulted in the formation of a white precipitate that was collected by filtration and washed with water. In order to remove any insoluble material, the precipitate was dissolved

in diethyl ether (~100 mL), decanted and solvent removed *in vacuo* for an extended period (6 h) to insure residual water was removed. Compound **8** was thus isolated as a white powder (10.56 g, 92%). ^{1}H NMR (400 MHz, $CDCl_3$) δ 4.20-3.80 (2H, br possible app d, app *J* ~10, N(CH*H*)$_2$), 2.95-2.77 (2H, br app t, app *J* ~12.6, N(C*H*H)$_2$), 2.51 (1H, tt, *J* 11.0 & 3.9, C*H*), 1.92 (2H,br app dd, app ~*J* 13.1 & 2.6, (C*H*H)$_2$CH), 1.67 (2H, ddt, (CH*H*)$_2$CH, *J* 13.3, 11.1 & 4.2), 1.46 (9H, s, *t*-Bu C*H*$_3$); ^{13}C NMR (101 MHz, $CDCl_3$) δ 170.1 (HO-*C*=O), 154.6 (N-*C*=O), 79.9 (O*C*(CH$_3$)$_3$, 43.7-41.7 (br s, N(*C*H$_2$)$_2$), 42.0 (*C*H), 28.4 ((*C*H$_3$)$_3$), 27.4 ((*C*H$_2$)$_2$CH); IR ν (cm^{-1}) 3187 (O-H) 2975 (C-H) 1801 (C=O) 1679 (C=O); MS (ES+) *m/z*: [M$^+$] 229.

*Synthesis of ethyl potassium malonate (**S4**)* [13]

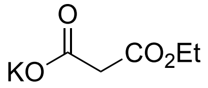

Potassium hydroxide (5.61 g, 0.10 mol) was dissolved in ethanol (100 mL) and added dropwise to a stirred solution of diethyl malonate (15 mL, 0.10 mol) dissolved in ethanol (100 mL), over 30 min. A voluminous white precipitate formed. The mixture was stirred vigorously for ~18 h. The solvent was removed *in vacuo* and the white crystalline residue mobilised from the flask using diethyl ether (in which the material was not soluble) and collected on a filter paper and dried under a flow of air (Büchner funnel) and product **S4** was collected as a white crystalline solid (14.28 g, 84%). ^{1}H NMR (400 MHz, D_2O) δ 4.08 (2H, q, *J* 7.2), 3.19 (s, 2H), 1.16 (3H, t, *J* 7.2); ^{13}C NMR (101 MHz, D_2O) δ 174.3, 171.5, 62.09, 44.7, 13.3; IR ν (cm^{-1}) 2984 (C-H) 1726 (C=O) 1595 (C=O) mp: 195-196 °C; MS (ES-) *m/z*: [M-K$^+$] 131.

*Synthesis of tert-butyl 4-(3-ethoxy-3-oxopropanoyl)piperidine-1-carboxylate (**9**)* [12b, 14]

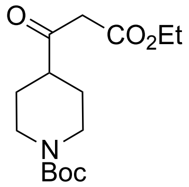

To an ice slush bath-cooled 2:1 mixture of tetrahydrofuran and acetonitrile (60 mL) compound **S4** (0.85 g, 5 mmol), magnesium chloride (0.95 g, 10 mmol, 2.0 eq.) and DMAP (61 mg, 0.8 mmol, 0.1 eq.) were added. The mixture was allowed to warm to room temperature and was stirred for 5 h. In an additional ice slush bath-cooled flask containing tetrahydrofuran (40 mL), compound **8** (1.14 g, 5 mmol, 1.0 eq.) and carbonyl diimidazole (1.05 g, 6.5 mmol, 1.3 eq.) were added. This flask was also allowed to warm to room temperature and was stirred for 4 h. The mixtures were cooled to 0 °C and the latter was added to the former, the resulting mixture was stirred for 18 h (for convenience). The resulting mixture was concentrated *in vacuo* to half its original volume. The resulting mixture was adjusted to pH 4 (universal indicator paper) by addition of aqueous HCl (1 M). The resulting mixture was extracted into ethyl acetate (3 x 50 mL), the combined organic extracts were then washed with aqueous HCl solution (1M, 50 mL), then aqueous NaOH solution (1 M, 50 mL) and finally with brine (50 mL). The organic fraction was dried over anhydrous magnesium sulphate, filtered and concentrated *in vacuo*. The yellow oil thus obtained was subjected to flash column chromatography (silica, hexane / ethylacetate gradient elution) resulting in isolation of product **9** as a colourless oil (0.36 g, 24%). ^{1}H NMR (300 MHz, $CDCl_3$) δ 4.37-4.05 (2H, m), 3.50 (1H, s), 2.62 (1H, tt, *J* 11.3 & 3.7), 2.05 (1H, s), 1.85 (1H, d, *J* 12.9), 1.78-1.48 (3H, m), 1.46 (12H, d, *J* 2.0), 1.43-1.23 (3H, m), 1.25 (1H, d, *J* 6.1); ^{13}C NMR (101 MHz, $CDCl_3$) 204.1, 167.3,

154.6, 79.5, 61.2, 48.7, 47.3, 43.1, 28.4, 27.2, 14.2; IR ν (cm^{-1}) 2975 (C-H) 1747, 1684 (C=O); MS (ES+) *m/z* [M+Na$^+$] 322.

*Synthesis of tert-butyl-4-(6-hydroxy-2-methylpyrimidin-4-yl)piperidine-1-carboxylate (**10**)* [15]

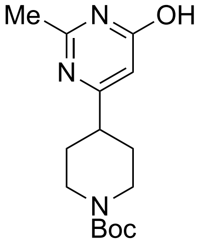

Acetamidine hydrochloride (260 mg, 2.75 mmol) and potassium carbonate (633 mg, 4.58 mmol) were dissolved in water (7 mL). To which a solution of compound **9** (549 mg, 1.83 mmol) in methanol (7 mL) was added. The mixture was stirred at 70 °C for 12 hours, after which time a saturated aqueous solution of ammonium chloride (7 mL) was added. The resulting mixture was extracted with dichloromethane (3 x 10 mL) and washed with water. The combined organic fractions were dried over anhydrous sodium sulphate,[16] and solvent removed *in vacuo*. The resulting white material was subjected to flash column chromatography (silica, dichloromethane / methanol gradient elution) to give **10** as a colourless powder (412 mg, 77%). ^{1}H NMR (400 MHz, $CDCl_3$) δ δ 13.14 (1H, s), 6.15 (1H, s), 4.23 (2H, s), 2.79 (2H, s), 2.54 (1H, tt, *J* 11.9 & 3.4), 2.47 (3H, s, Me), 1.89 (2H, d, *J* 13.0), 1.56 (2H, qd, *J* 12.7 & 4.6), 1.49 (9H, s); ^{13}C NMR (101 MHz, $CDCl_3$) δ 172.5, 165.9, 158.4, 154.8, 107.6, 79.6, 43.8, 30.3, 28.5, 21.7; IR ν (cm^{-1}) 3250 (br), 2931, 1685, 1658; MS (ES+) *m/z* 316 [M$^+$+Na$^+$].

alamarBlue® assay

The EC_{50} data contained from each repeat analysis of the activity of compounds discussed against the MEC1 cell line, as judged by the alamarBlue® assay is detailed in Supplementary Table 2. As expected, parthenolide (**1**, Supplementary Table 2, entry 1, EC_{50} = 3.4 μM) displayed good activity against Mec1 cells *in vitro*.[4] Among the tertiary amine derivatives, twelve showed sub 20 μM EC_{50} activity (Supplementary Table 2, entries 4-14 and 29). Thioether compound **3**, which results from 1,4-thiol addition to parthenolide **1**, displayed no activity against the MEC1 CLL cell line (Supplementary Table 2, entry 30). Since tertiary and secondary amine analogues (**2d** and **4a**) displayed good and acceptable activity respectively, the inactivity of thioether **3** is not inconsistent with a prodrug hypothesis where a retro-Michael-like reaction reveals parthenolide, although mechanistic conclusion cannot be drawn from this observation alone. In this assay secondary amine products (**4a-ae**, Supplementary Table 2, entries 31-61) displayed insufficient activity to warrant further investigation. Products that contain a cyclic tertiary amine (**5a** to **5ab**) include eleven active products with an EC_{50} <25.0 μM against the MEC-I CLL cell line (Supplementary Table 2, entries 62, 63, 65-72, 74 and 89).

Supplementary Table 2. *Parthenolide derivatives synthesised, calculated properties (CDD Vault) and measured EC_{50} against the MEC1 CLL line using the alamarBlue® (ThermoFischer Scientific) assay. References against compound numbers indicate citations to the compounds that have been previously reported (SciFinder).*

Entry [a]	Compound	Mwt (g/mol)	log P	TPSA (Å^2)	Fsp^3	alamarBlue® E_{50} (μM) n1	n2	n3	n4	n5	average	Std. dev.
1 [b]	**1**	248.32	3.07	38.83	0.67	6.5	6.7	6.3	8.4	2.8	6.2 [d]	2.0
2 [b]	**2a** [6]	293.41	2.36	42.07	0.82	5.5	6.4	8.2	3.1	3.1	5.3 [d]	2.2

3 [b]	**2b** [7a]	321.46	3.13	42.07	0.84	> 30.0	> 30.0	-	-	-
4 [b]	**2c** [7]	323.43	1.67	62.30	0.83	6.2	4.2	-	5.2	1.4
5 [b]	**2d**	337.46	2.02	62.30	0.84	5.5	3.9	-	4.7	1.1
6	**2e**	415.58	3.64	59.89	0.75	4.7	7.8	4.3	10.6	1.9
7	**2f**	361.53	4.02	42.07	0.86	15.8	8.8	11.9	12.2	3.5
8	**2g**	391.55	3.47	62.3	0.87	16.8	4.9	8.4	10.0	6.1
9	**2h**	403.95	4.69	42.07	0.61	17.3	24.2	13.5	18.3	5.4
10	**2i**	443.58	3.59	71.53	0.65	21.0	22.5	14.1	19.2	4.5
11	**2j**	429.56	3.77	60.53	0.64	16.3	5.2	7.7	9.7	5.8
12 [c]	**2j**	429.56	3.77	60.53	0.64	4.0	7.6	5.3	7.9	1.8
13	**2k**	427.59	4.13	70.75	0.76	20.9	22.0	15.0	19.3	3.8
14	**2l**	448.00	4.89	51.30	0.64	18.0	23.0	18.5	19.8	2.8
15	**2m**	441.59	4.12	70.75	0.58	18.0	> 30.0	-	-	-
16	**2n**	437.54	3.16	80.99	0.56	> 30.0	> 30.0	-	-	-
17	**2o**	390.57	2.85	45.31	0.87	16.5	> 30.0	17.7	-	-
18	**2p**	437.50	4.96	42.07	0.62	> 30.0	> 30.0	-	-	-
19	**2q**	438.53	3.27	93.88	0.58	> 30.0	> 30.0	-	-	-
20	**2r**	415.59	4.97	42.07	0.71	> 30.0	> 30.0	-	-	-
21	**2s**	417.55	3.69	80.99	0.78	> 30.0	> 30.0	-	-	-
22	**2t**	441.59	4.78	55.21	0.56	> 30.0	> 30.0	-	-	-
23	**2u**	490.60	4.21	73.42	0.52	> 30.0	> 30.0	-	-	-
24	**2v**	445.60	2.79	69.12	0.76	21.5	> 30.0	-	-	-
25	**2w**	427.59	5.03	51.30	0.65	21.0	> 30.0	-	-	-
26	**2x**	450.58	4.08	64.19	0.56	16.5	> 30.0	-	-	-
27	**2y**	437.58	4.47	70.75	0.62	> 30.0	> 30.0	-	-	-
28	**2z**	401.52	4.58	42.07	0.62	17.0	> 30.0	-	-	-
29	**2aa**	383.53	4.37	42.07	0.62	5.2	7.7	4.7	5.9	1.6
30 [b]	**3** [7b, 8]	326.45	2.34	59.06	0.82	> 30.0	> 30.0	-	-	-
31 [b]	**4a** [7a]	309.41	1.28	71.09	0.82	20.4	17.6	-	19.0	2.0
32 [b]	**4b**	323.43	1.93	60.09	0.83	> 30.0	> 30.0	-	-	-
33 [b]	**4c** [7b]	355.48	3.70	50.86	0.59	23.4	17.3	-	20.4	4.3
34 [b]	**4d** [9]	303.40	2.20	50.86	0.72	> 30.0	> 30.0	-	-	-
35	**4e**	385.50	3.54	60.09	0.61	17.3	> 30.0	23.3	-	-
36	**4f**	363.50	2.40	60.09	0.86	24.5	20.9	24.6	23.3	2.1
37	**4g**	392.50	0.87	80.40	0.81	17.0	> 30.0	-	-	-
38	**4h**	399.53	4.13	60.09	0.62	18.9	20.6	26.2	21.9	3.8
39	**4i**	407.91	4.45	50.86	0.59	> 30.0	> 30.0	-	-	-
40	**4j**	471.64	4.83	69.32	0.68	17.2	> 30.0	-	-	-
41	**4k**	483.57	3.31	108.24	0.58	> 30.0	> 30.0	-	-	-
42	**4l**	409.45	4.13	50.86	0.59	> 30.0	> 30.0	-	-	-
43	**4m**	345.44	2.76	64.00	0.65	> 30.0	> 30.0	-	-	-
44	**4n**	398.55	3.81	54.10	0.62	> 30.0	> 30.0	-	-	-
45	**4o**	413.51	3.70	77.16	0.58	21.6	> 30.0	-	-	-
46	**4p**	385.50	3.54	60.09	0.61	> 30.0	> 30.0	-	-	-
47	**4q**	398.55	3.81	54.10	0.62	> 30.0	> 30.0	-	-	-

48	**4r**	406.57	2.47	63.33	0.87	> 30.0	> 30.0	-	-	-
49	**4s**	376.52	2.67	63.75	0.7	> 30.0	> 30.0	-	-	-
50	**4t**	424.36	4.91	50.86	0.59	> 30.0	> 30.0	-	-	-
51	**4u**	423.48	4.58	50.86	0.61	> 30.0	> 30.0	-	-	-
52	**4v**	423.48	4.58	50.86	0.61	> 30.0	> 30.0	-	-	-
53	**4w**	439.48	5.13	60.09	0.61	> 30.0	> 30.0	-	-	-
54	**4x**	439.48	5.13	60.09	0.61	> 30.0	> 30.0	-	-	-
55	**4y**	439.48	5.13	60.09	0.61	> 30.0	> 30.0	-	-	-
56	**4z**	391.46	3.98	50.86	0.59	> 30.0	> 30.0	-	-	-
57	**4aa**	441.47	4.72	50.86	0.61	> 30.0	> 30.0	-	-	-
58	**4ab**	441.47	4.72	50.86	0.61	> 30.0	> 30.0	-	-	-
59	**4ac**	424.36	4.91	50.86	0.59	> 30.0	> 30.0	-	-	-
60	**4ad**	407.91	4.45	50.86	0.59	> 30.0	> 30.0	-	-	-
61	**4ae**	383.53	4.73	50.86	0.62	16.8	> 30.0	-	-	-
62 [b]	**5a** [7a, 8-10]	319.45	2.76	42.07	0.84	9.9	6.0	-	7.7	2.8
63 [b]	**5b** [7a, 8b, 8c, 9-10]	333.47	3.21	42.07	0.85	10.1	5.5	-	7.8	3.3
64 [b]	**5c** [8c, 10b]	348.49	2.20	45.31	0.85	>30	>30	-	-	-
65	**5d**	426.56	2.79	64.19	0.68	4.8	3.8	7.6	8.7	2.0
66	**5e**	425.57	3.32	62.30	0.65	4.7	3.7	9.6	9.8	3.2
67	**5f**	441.57	1.42	83.53	0.72	7.3	5.9	9.8	10.6	2.0
68	**5g**	391.51	3.02	60.53	0.86	18.2	14.0	13.3	15.2	2.7
69	**5h**	452.60	3.48	85.16	0.63	17.0	11.0	13.5	13.8	3.0
70	**5i**	375.55	4.38	42.07	0.87	25.0	24.0	17.6	22.2	4.0
71	**5j**	392.54	2.16	54.54	0.86	17.7	23.0	17.7	19.5	3.1
72	**5k**	464.65	4.15	45.31	0.69	17.9	12.7	18.3	16.3	3.1
73	**5l**	406.52	2.41	71.61	0.82	29.6	> 30.0	-	-	-
74	**5m**	412.53	2.85	71.09	0.7	23.0	29.5	21.9	24.8	4.1
75	**5n**	523.09	3.54	79.45	0.65	27.8	> 30.0	-	-	-
76	**5o**	452.60	3.54	62.38	0.63	> 30.0	> 30.0	-	-	-
77	**5p**	434.58	4.18	65.86	0.63	> 30.0	> 30.0	-	-	-
78	**5q**	415.51	4.03	51.30	0.62	19.0	> 30.0	-	-	-
79	**5r**	416.61	3.23	45.31	0.88	> 30.0	> 30.0	-	-	-
80	**5s**	390.57	3.26	45.31	0.87	> 30.0	> 30.0	-	-	-
81	**5t**	444.63	4.13	45.31	0.72	> 30.0	> 30.0	-	-	-
82	**5u**	430.63	4.32	45.31	0.88	> 30.0	> 30.0	-	-	-
83	**5v**	440.47	2.38	72.78	0.76	> 30.0	> 30.0	-	-	-
84	**5w**	444.01	5.23	42.07	0.65	22.0	> 30.0	-	-	-
85	**5x**	390.57	3.53	45.31	0.87	> 30.0	> 30.0	-	-	-
86	**5y**	439.60	4.52	51.30	0.67	19.0	> 30.0	-	-	-
87	**5z**	427.56	4.77	42.07	0.65	18.0	> 30.0	-	-	-
88	**5aa**	468.60	3.13	71.61	0.63	> 30.0	> 30.0	-	-	-
89	**5ab**	440.58	2.83	64.19	0.69	10.0	8.8	3.9	11.6	3.2
90 [b]	**6a** [11]	323.39	-1.02	88.16	0.76	> 30.0	15.8	-	-	-
91 [b]	**6b**	413.51	1.21	88.16	0.58	16.0	11.0	-	13.5	3.5

[a] Synthesised as per Scheme 3 (main text) conditions (ii), unless otherwise indicated. All EC_{50} values in this table were (unless otherwise stated) determined in a higher-throughput fashion (n = 3) by Sygnature Discovery; [b] Synthesised as per Scheme 3 (main text), conditions (i); [c] Repetition of previous entry; [d] Determined by a manual technique at University of Birmingham (n =5).

Calculated properties of parthenolide derivatives

A graphical summary of calculated properties for the synthesised compounds is presented in Supplementary Figure 2, where molecular weight, calculated logP, fraction of sp^3 atoms and topological polar surface area are surveyed (in each plot the red cross = parthenolide **1**).[17] Plots of Supplementary Figure 2 (a)i. and (b)i. represent the proposed virtual molecule set (squares coloured blue) from which were selected for synthesis. Plots Supplementary Figure 2 (a)ii. and (b)ii. (yellow/orange) triangles show the compounds that were synthesised (listed in Supplementary Figure 2). Plots of Supplementary Figure 2 (a)iii. and (b)iii. show the compounds that displayed an EC_{50} <25.0 μM (active) against the MEC1 cell line using the alamarBlue® assay (biological activity is discussed in the following section). These plots confirm that the virtual set of parthenolide derivatives populates a broad area of chemical space of relevance to orally bioavailable drug discovery and that the synthesised compounds are distributed throughout that space.[18] Notably, the parthenolide core confers a high degree of sp^3 character and three-dimensionality to the compound collection herein.[19] Compounds with an EC_{50} <25.0 μM (active) are indicated as circular (green) points in the property distribution plots of Supplementary Figure 2. The distribution of hits across the chemical space described in those plots evidences the appropriateness of hits for further development in a drug discovery programme.

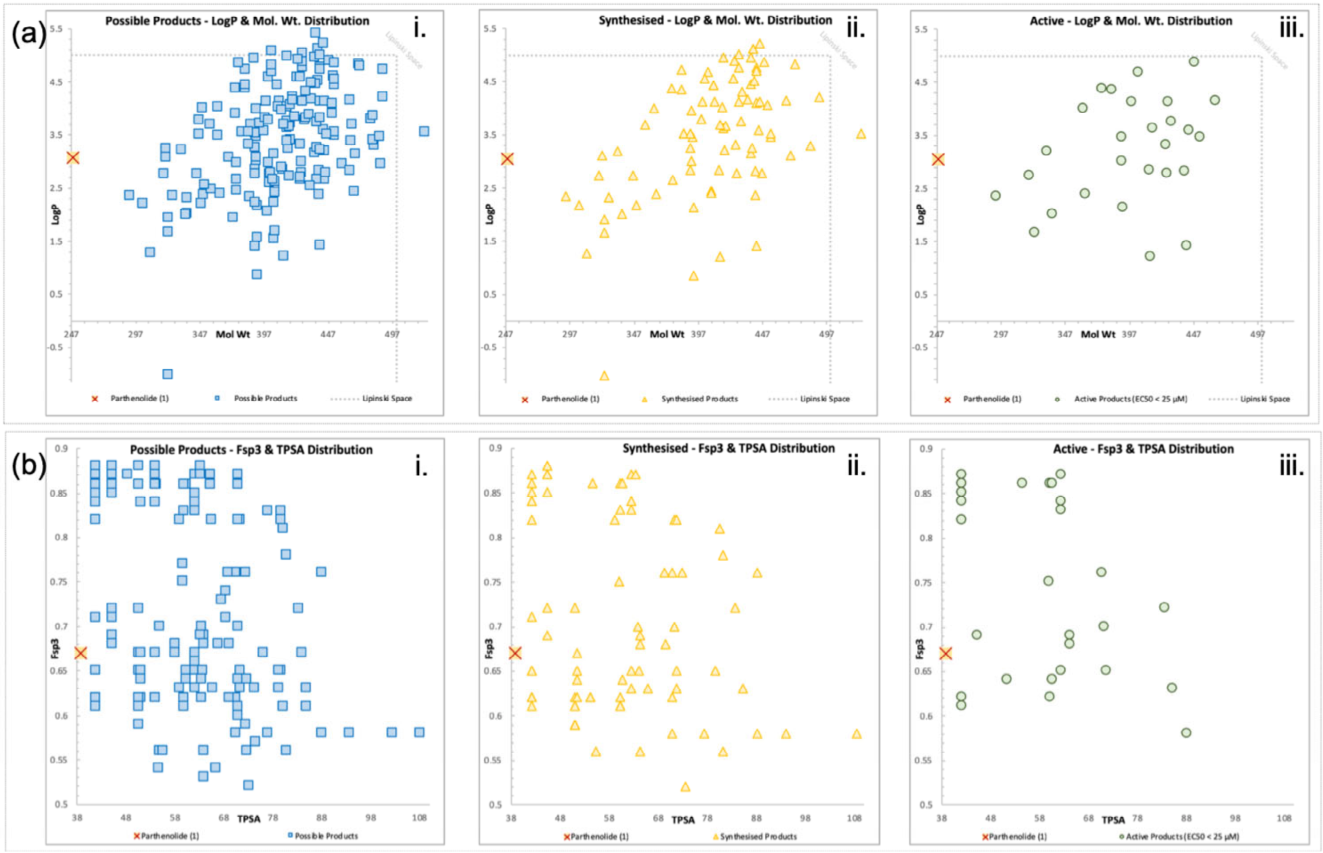

Supplementary Figure 2. (a) *Calculated logP (CDD Vault)* versus *molecular weight &* ***(b)*** *Fraction of sp*^{3} *atoms* versus *topological polar surface area for:* ***i:*** *set of possible* virtual *products;* ***ii:*** *the compounds synthesised;* ***iii:*** *the compounds with activity (*EC_{50} *< 25.0 μM). The parent scaffold, parthenolide* ***1****, shown as a (red) cross in each plot for reference.*

Computational study

In silico *ligand binding study*

In order to explore some plausible mechanisms for the activity of the selected amino parthenolide derivatives, docking studies were performed. One aspect of interest is whether the compounds act as prodrugs, that function to deliver parthenolide to a site of action, and this was explored by docking derivatives **2a**, **5a** and **5f** and also the drug they all feasibly liberate, parthenolide **1** (Supplementary Scheme 2). One possibility that has been considered is that the prodrugs will fragment in a protein binding site yielding the drug if there are appropriate conditions to facilitate the proton-shuffling required for the elimination of the amine. Further reactivity is likely if this elimination occurs in the presence of a cysteine thiol. When reactions occur in the diffusion limiting environment in protein binding sites, the eliminated amine could act as a base to deprotonate the thiol and promote its addition to the Michael-acceptor that had been revealed by loss of the amine.

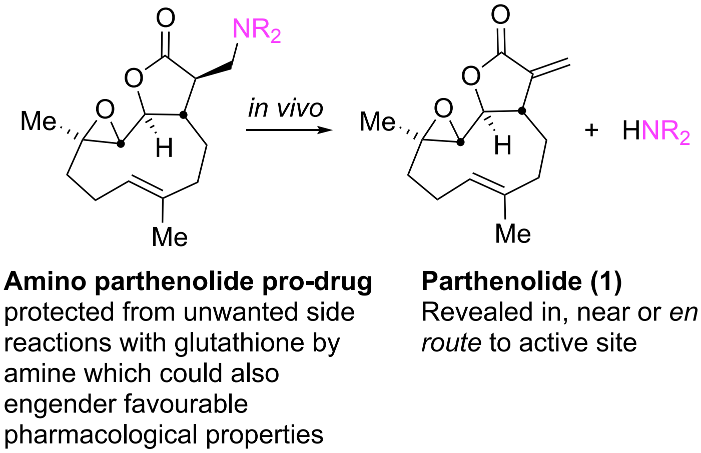

Supplementary Scheme 2. *Proposed retro-Michael-type reaction that delivers parthenolide (**1**) from amino parthenolide derivatives.*

Two biological mechanisms by which the active compounds might act have been considered computationally; one or both of these might contribute to the activity reported above. The first mode of action is inhibition of the binding between DNA and the p65 domain of NF-κB. This mechanism has been shown to contribute to the anti-inflammatory activity of **1** and other reactive compounds, it is dependent upon Cys 38, presumably alkylation of its thiol lies at the origin of this observation.[20] Double reactivity involving a second reaction with Cys 120 had been proposed but mutation studies supported only a minor role for interaction with this residue. In order to investigate and illustrate this mode of action, a relevant protein structure was sought. The highest resolution structure of the p65 subunit of NF-κB in which Cys 38 is present was found by searching the protein data bank and has the structure code 2RAM.[21] The receptor structure was prepared by deleting the DNA and selecting only protein chain A. Docking employed the Autodock Vina code,[22] with protein structures prepared using the suite of Autodock tools using default settings.[23] The binding site was defined by a box centred on the sulfur atom in Cys 38. The box was selected to have length 18 Å based on a longest atom-atom distance of 9 Å in the crystal structure of **1** (detailed in Supplementary Figure 1). The

conformation found in this crystal structure was used as the input geometry for this ligand while the other structures were obtained by editing this structure using the Gaussview[24] program and converting them to the appropriate input format with Open Babel.[25]

The docking pose that was scored best for each compound (**1**, **2a**, **5a** and **5f**) is shown in Supplementary Figure 3 (all compounds achieved docking scores in the range -4.0 to -5.0 where more negative scores are preferred). This reveals that parthenolide **1** can bind in close proximity to Cys 38 in such a way that it is primed to react; the relevant intermolecular S-C distance is highlighted and is 4.1 Å. There is a salt bridge between Glu 39 and Arg 187 that is in close proximity to Cys 38 and the three amines that have been docked (**2a**, **5a** and **5f**) are all found to bind in identical binding modes in which the protonated amine is proximal to Glu 39. This mode could plausibly permit the sidechain carboxylate of Glu 39 to deprotonate at the α-carbon adjacent to the carbonyl in each of these derivatives and therefore promote elimination to form **1** *in situ*.

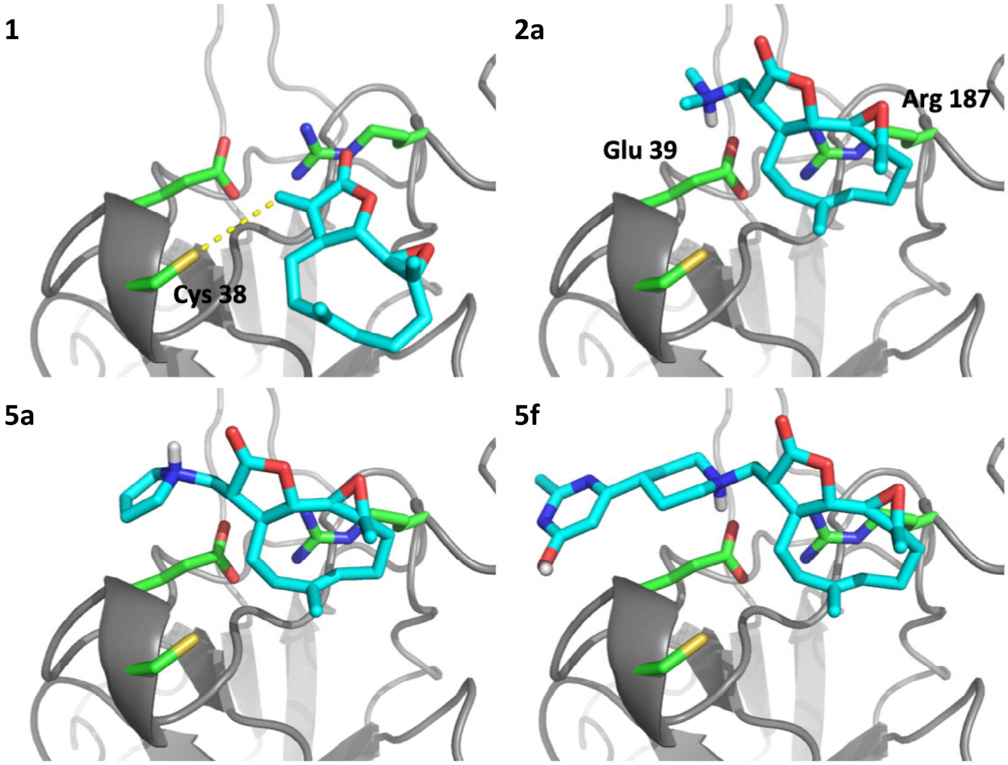

Supplementary Figure 3. *Docking poses arising from docking in proximity to Cys 38 in the protein with structure code 2RAM. The identity of the ligand in each case is indicated next to the illustration: Upper left **1**; upper right **2a**; lower left **5a**; lower right **5f**.*

The second mode of action that was considered is inhibition of the Inhibitor of κB Kinase, IKKβ. IKKβ phosphorylates IκB targeting it for proteosomal degradation, thus releasing the NF-κB heterodimer p50/p65 which translocates to the nucleus. Parthenolide **1** and related compounds have been shown to reduce phosphorylation of p65 in cells taken from acute myelogeous leukaemia (AML) patients and inhibition of IKKβ would contribute to this effect.[26] A structure of this kinase in complex with a pyrimidine derivative inhibitor is reported, protein data bank entry code 3RZF, the inhibitor's structure is depicted in the supporting information (compound **S5**, Supplementary Figure 5).[27] This

structure reveals that despite having a hinge-binding motif (adjacent donor and acceptor, a typical binding mode for kinases), the inhibitor in structure 3RZF does not exploit this. The pyrimidine core of that literature compound (**S5**) binds adjacent to Cys 99, which raised the possibility that parthenolide (and derivatives thereof) described above might act covalently. In order to investigate these possibilities, docking was performed using the coordinates of the sulfur atom in Cys 99 as the centre of the box with a box side of length 18 Å. The docking scores for the poses discussed below for **1**, **2a** and **5a** were in the range -5.8 to -6.1 while that for **5f** was -4.5.

The nine best scoring poses of **1** were examined to identify the one that places the Michael acceptor in closest proximity to the cysteine thiol of Cys 99 (Supplementary Figure 4: upper left). The cysteine in this case has its sidechain rotated in towards the rest of the protein and so long as its motion is not hindered, it could presumably swing out. The distance between the sulfur atom and the β-carbon of the Michael acceptor is 6.5 Å. For the amino parthenolide derivatives considered (**2a**, **5a** and **5f**), one docking pose was identified that is common to all of the compounds studied (Supplementary Figure 4: upper right, lower left and lower right respectively). During a supposed *in situ* formation of parthenolide **1** from these compounds, the only amino acid sidechain that might facilitate fragmentation is Asp 103 (as labelled in Supplementary Figure 4: upper right). It is placed on the appropriate face to deprotonate the α-carbon of the amine derivatives to promote their fragmentation (*via* a retro-Michael-type mechanism) and the liberated amine would be trapped in the binding site proximal to the cysteine where it could facilitate deprotonation of the cysteine should it attack the Michael acceptor in a subsequent Michael-type reaction. This binding mode could also operate in a non-covalent fashion such that compounds exert their effect by simply occupying the binding mode shown, although this is unlikely to explain the activity of **5f**, which is a poor fit to the binding site; given the good activity of this compound, this supports a prodrug mode of action.

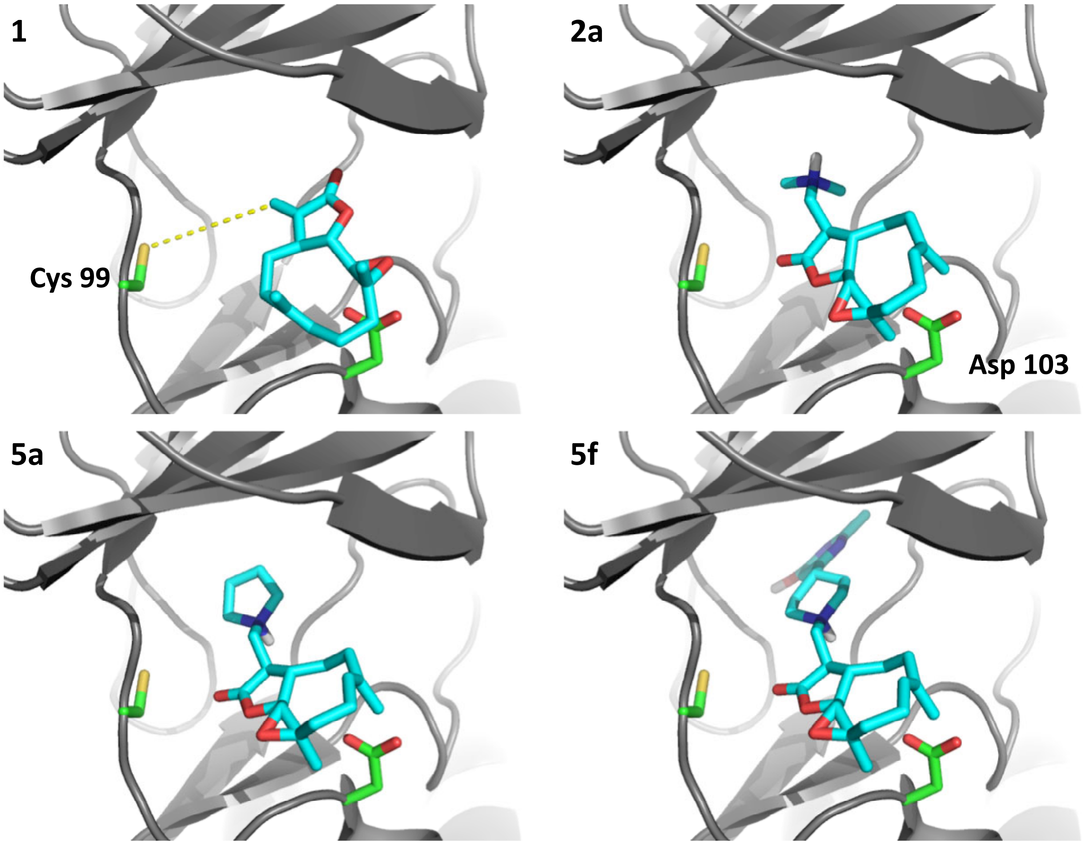

Supplementary Figure 4. *Docking poses arising from docking in proximity to Cys 99 in the protein with structure code 3RZF. The identity of the ligand in each case is indicated next to the illustration: Upper left **1**; upper right **2a**; lower left **5a**; lower right **5f**.*

The docking studies support a mechanism of action *via* liberation of parthenolide because compound **5f** is unlikely to bind better than **1** to either of the proteins considered. Compound **1** could plausibly bind in a covalent fashion to the p65 domain of NF-κB and to IKKβ. Both of these interventions in the same pathway would be consistent with the observed activity.

Docking scores

Docking scores pertaining to the main text computational study are given in Supplementary Table 3.

Supplementary Table 3. *Docking scores from computational binding studies.*

Compound	Best docking score in structure 3RZF	Docking score of selected pose in 3RZF	Best docking score in structure 2RAM	Docking score of selected pose in 2RAM
**1**	-7.7	-5.9	-4.6	-4.6
**2a**	-6.8	-5.8	-4.0	-3.6
**5a**	-7.0	-6.1	-4.3	-4.3
**5f**	-4.6	-4.5	-5.0	-5.0

Inhibitor structure

The compound bound to 3RZF in the literature reported structure of Xu *et al.* is given below (**S5**).[27]

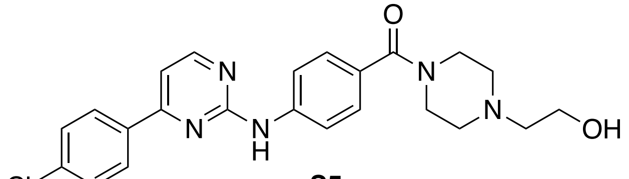

S5

Supplementary Figure 5*. The inhibitor of 3RZF (****S5****) in the structure reported by Xu et al.* [27]

Toxicology efforts hERG liability assay at Apconix Ltd

Compounds were solubilised to 30 mM in DMSO before dilution in PBS to 300 mM. A further 3-fold on-board dilution resulted in a final top test concentration of 100 mM. Eight-point concentration-response curves were generated using 3.16-fold serial dilutions from the top test concentration.

Electrophysiological recordings were made from a Chinese Hamster Ovary cell line stably expressing the full length hERG channel. Single cell ionic currents were measured in the perforated patch clamp configuration (100 μgmL^{-1}) amphotericin) at room temperature (21-23 °C) using an IonWorks Quattro instrument (Molecular Devices). The internal solution contained (mM): 140 KCl, 1 $MgCl_2$, 1 EGTA, 20 HEPES and was buffered to pH 7.3. The external solution (phosphate-buffered saline, PBS) contained (mM): 138 NaCl, 2.7 KCl, 0.9 $CaCl_2$, 0.5 $MgCl_2$, 8 Na_2HPO_4, 1.5 KH_2PO_4 buffered to pH 7.4. Cells were clamped at a holding potential of -70 mV for 30 s and then stepped to +40 mV for 1 s. This was followed by a hyperpolarising step of 1 s to -30 mV to evoke the hERG tail current. Currents were measured from the tail step and referenced to the holding current. Compounds were then incubated for 3-4 minutes prior to a second measurement of the hERG signal using an identical pulsetrain.

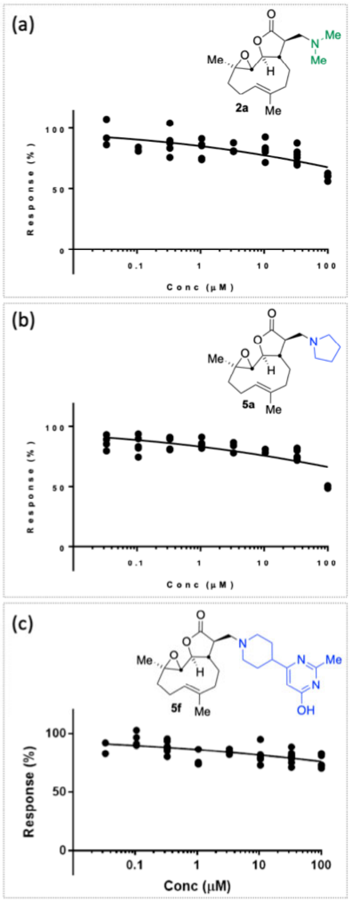

Supplementary Figure 6. *Graphical representation of the log of molar concentration of (a)* ***2a****, (b)* ***5a*** *and (c)* ***5f*** *against the post-compound hERG current, expressed as a percentage of the pre-compound signal. Data are normalised such that a value of 100% = no drug effect. Each point represents data from an individual cell.*

Supplementary Table 4.

Entry	Compound	Top test conc./μM	IC_{50} /μM	pIC_{50}	Inhibition at top conc./%	Flag
1	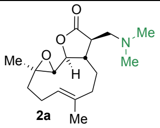 2a	100	>100	<4	40	Active
2	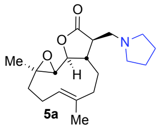 5a	100	>100	<4	49	Active
3	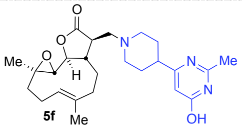 5f	100	>100	<4	24	Inactive
4	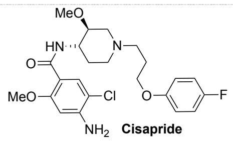 Cisapride	10	0.1	7		Positive Control

Spectroscopy and spectrometry

NMR spectrums obtained from efforts by University of Birmingham

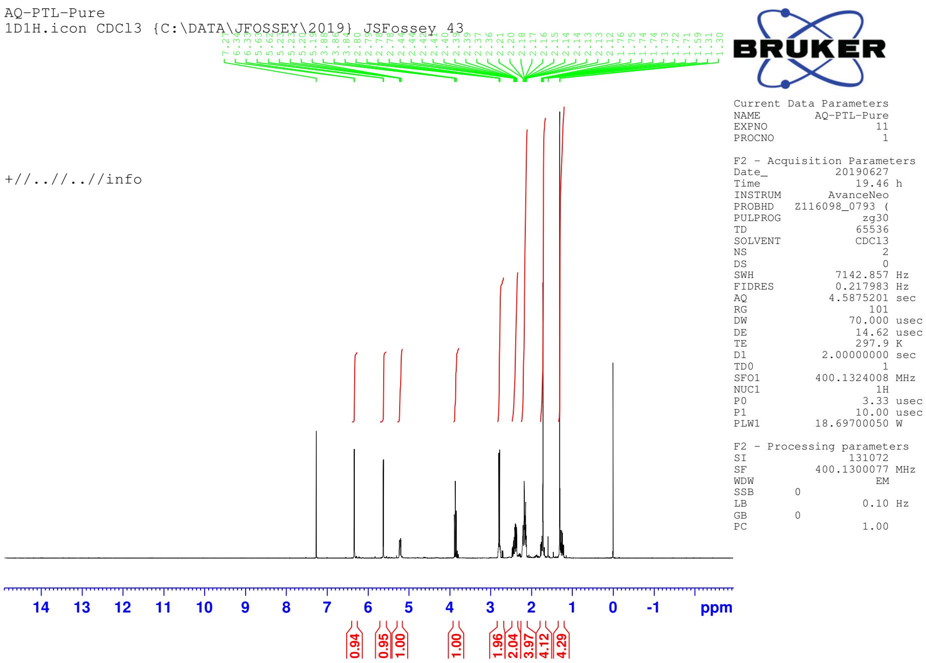

Supplementary Figure 7. *Proton NMR spectrum of parthenolide **1**.*

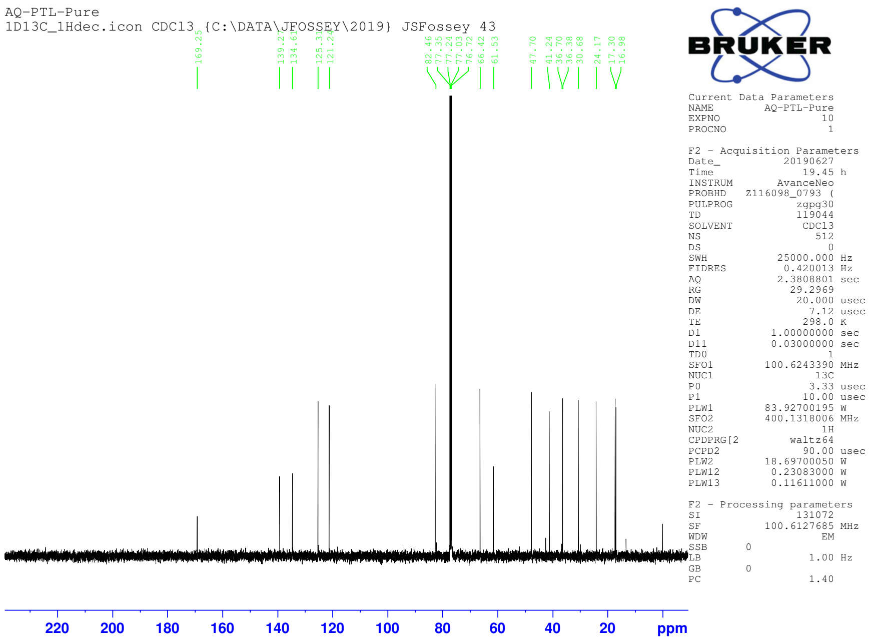

Supplementary Figure 8. *^{13}C NMR spectrum of parthenolide **1**.*

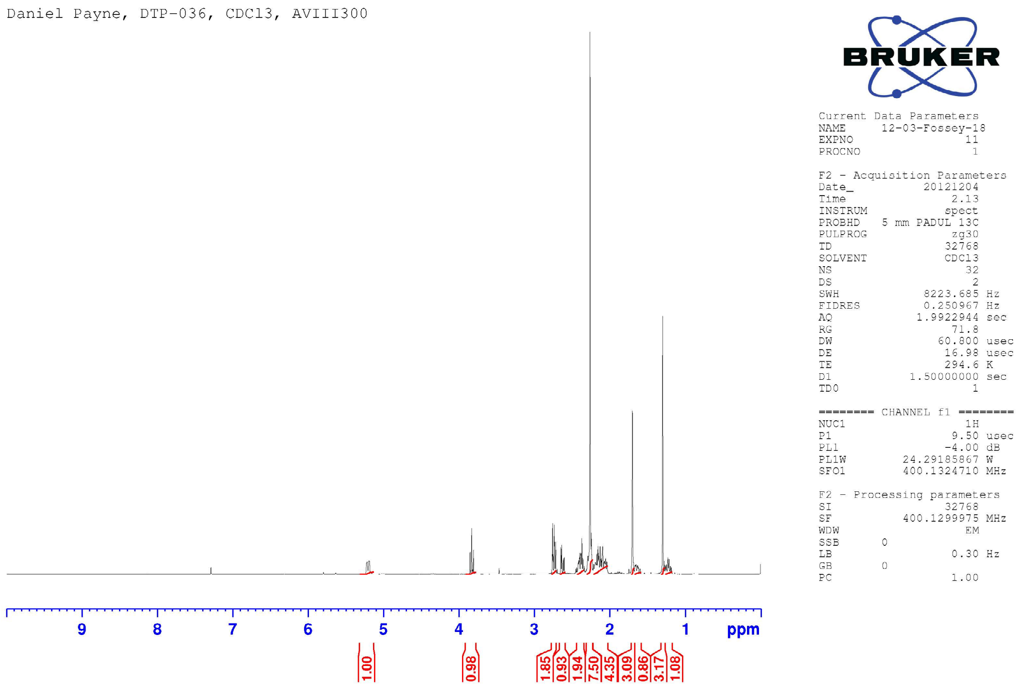

Supplementary Figure 9. *Proton NMR spectrum of **2a**.*

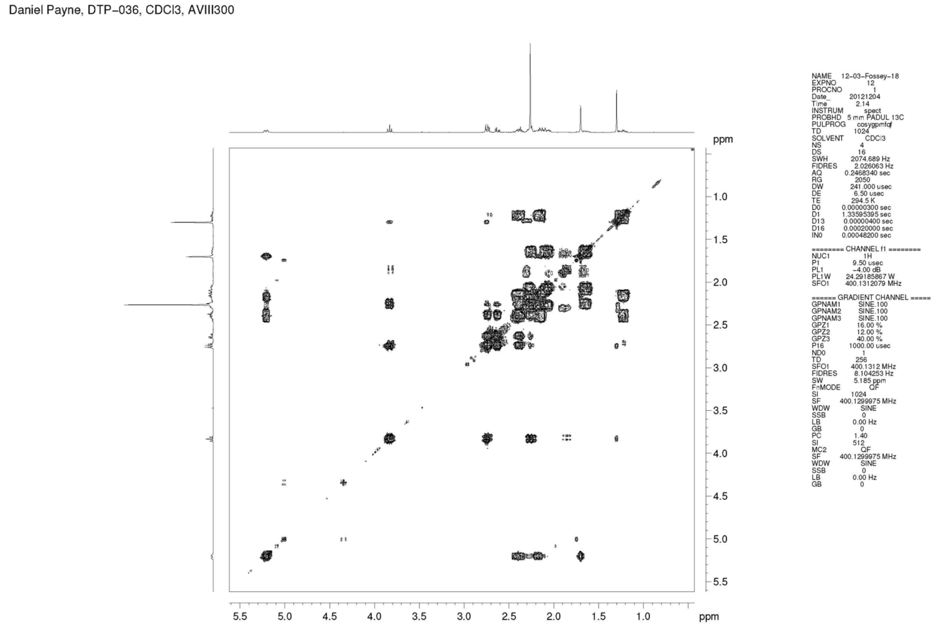

Supplementary Figure 10. *^{1}H-^{1}H 2D COSY NMR spectrum of **2b**.*

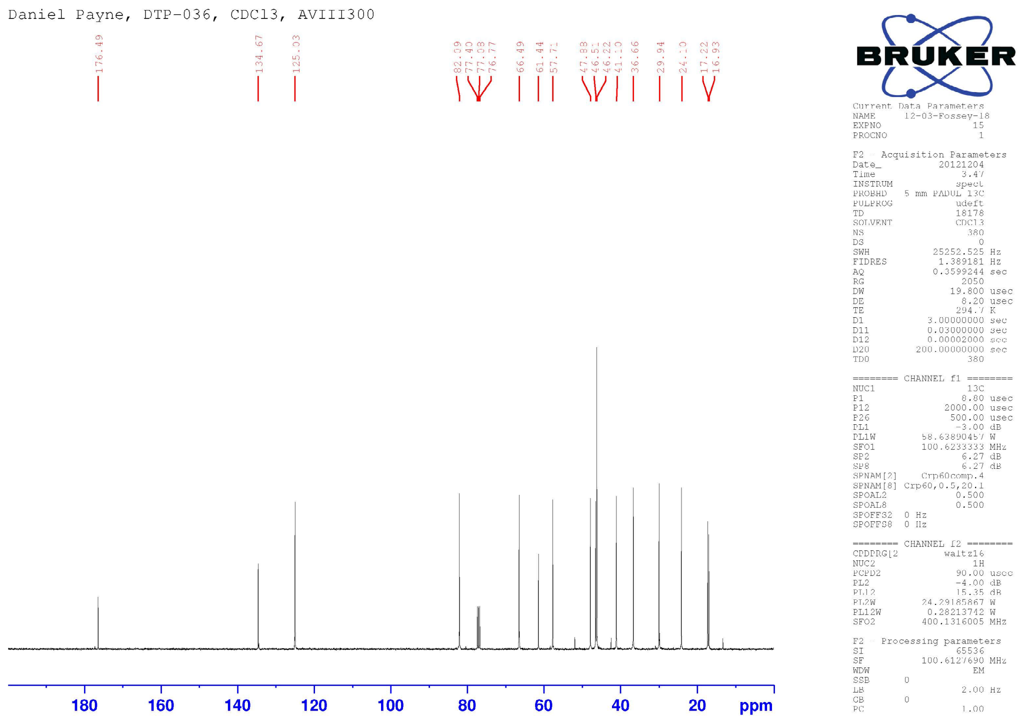

Supplementary Figure 11. ^{13}C NMR spectrum of **2a**.

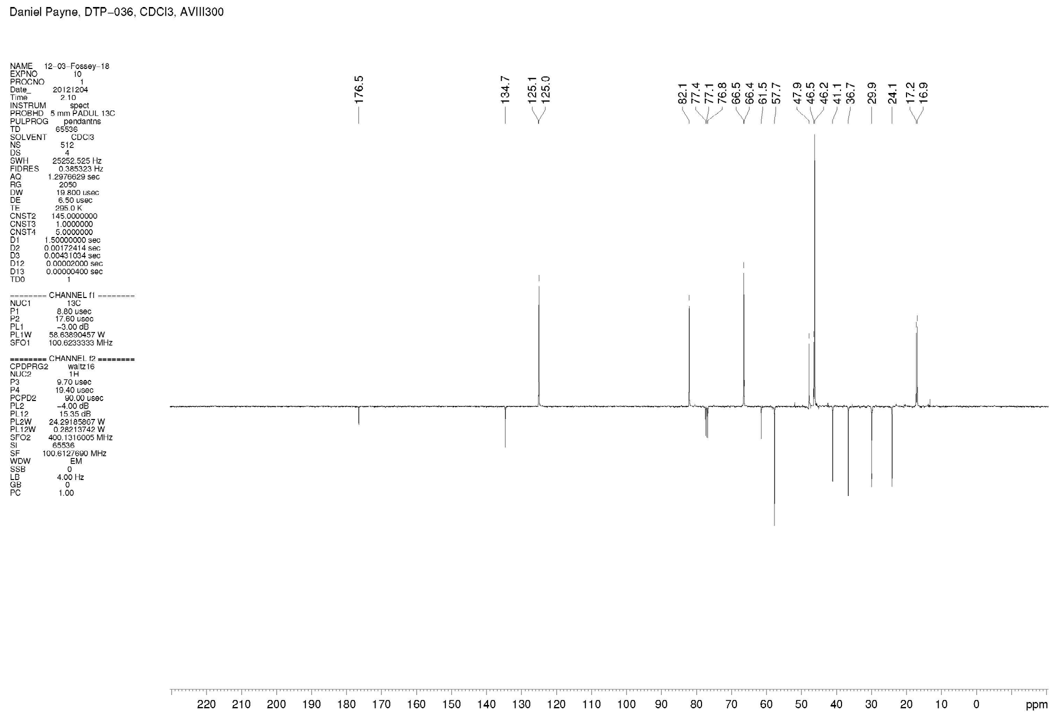

Supplementary Figure 12. PENDANT NMR spectrum of **2a**.

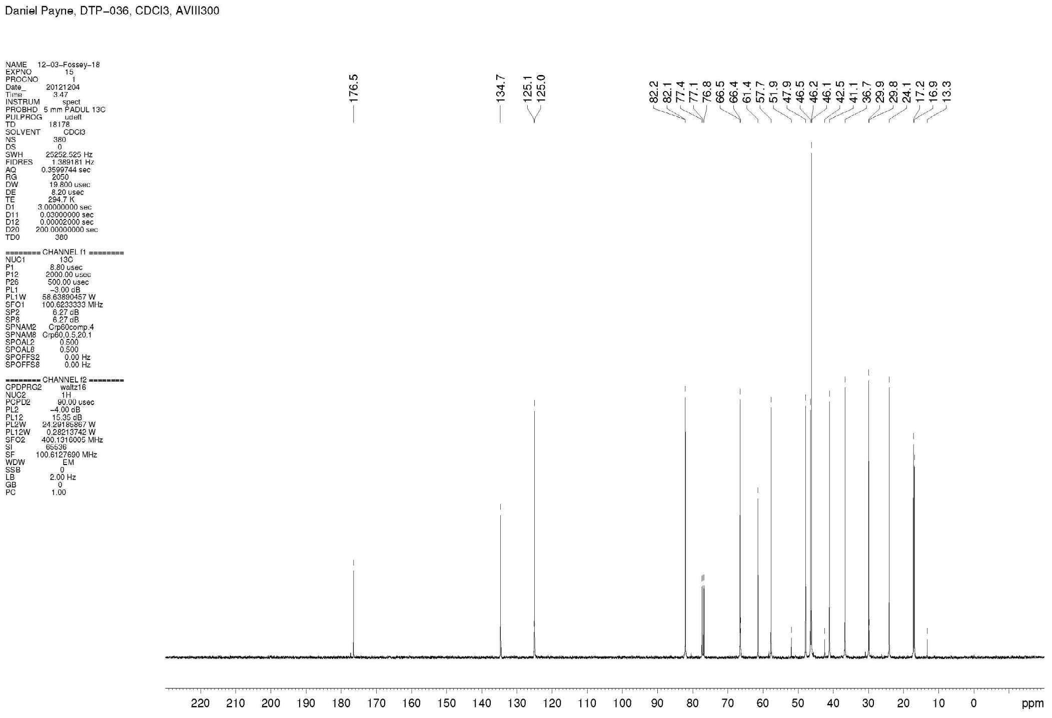

Supplementary Figure 13. *UDEFT NMR spectrum of* ***2a.***

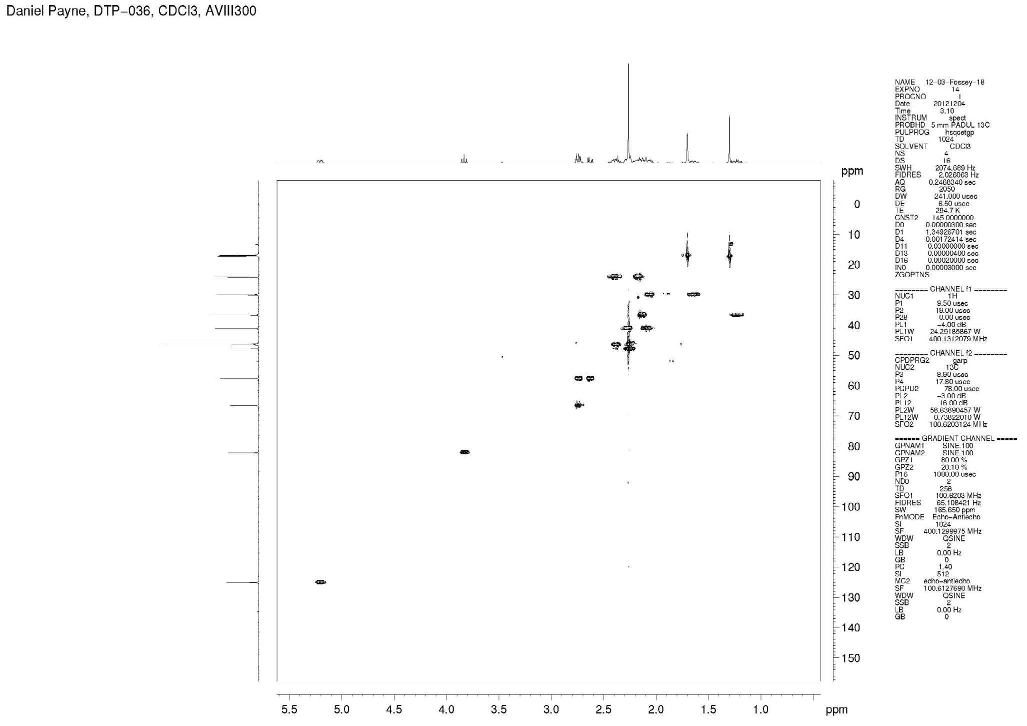

Supplementary Figure 14. *HSQC NMR spectrum of* ***2a.***

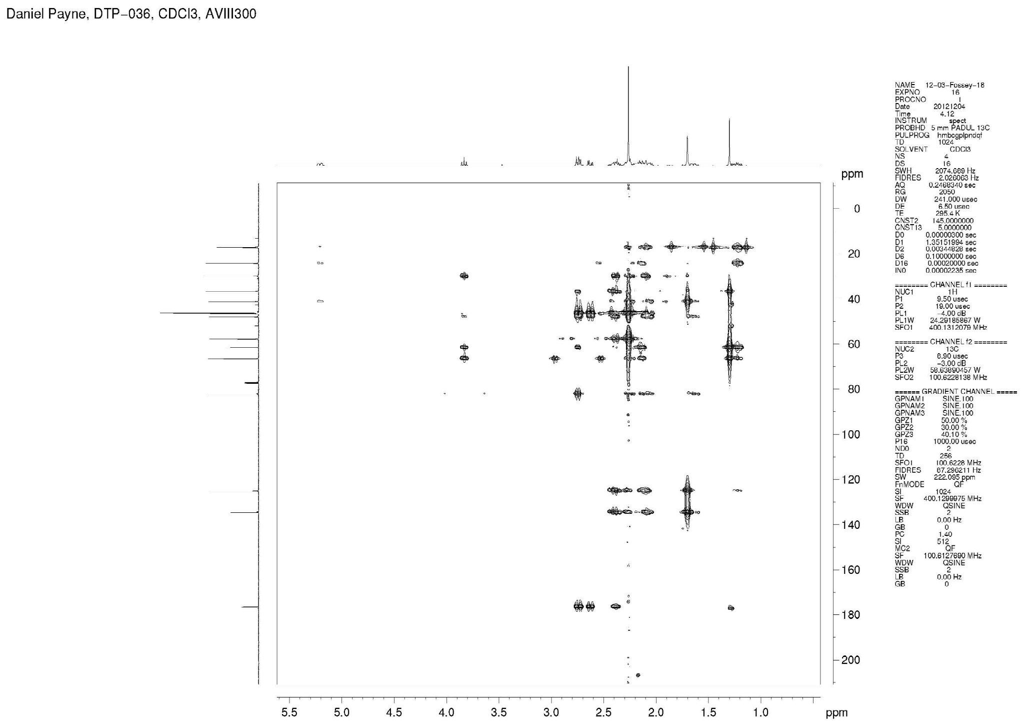

Supplementary Figure 15. *HMBC NMR spectrum of* ***2a****.*

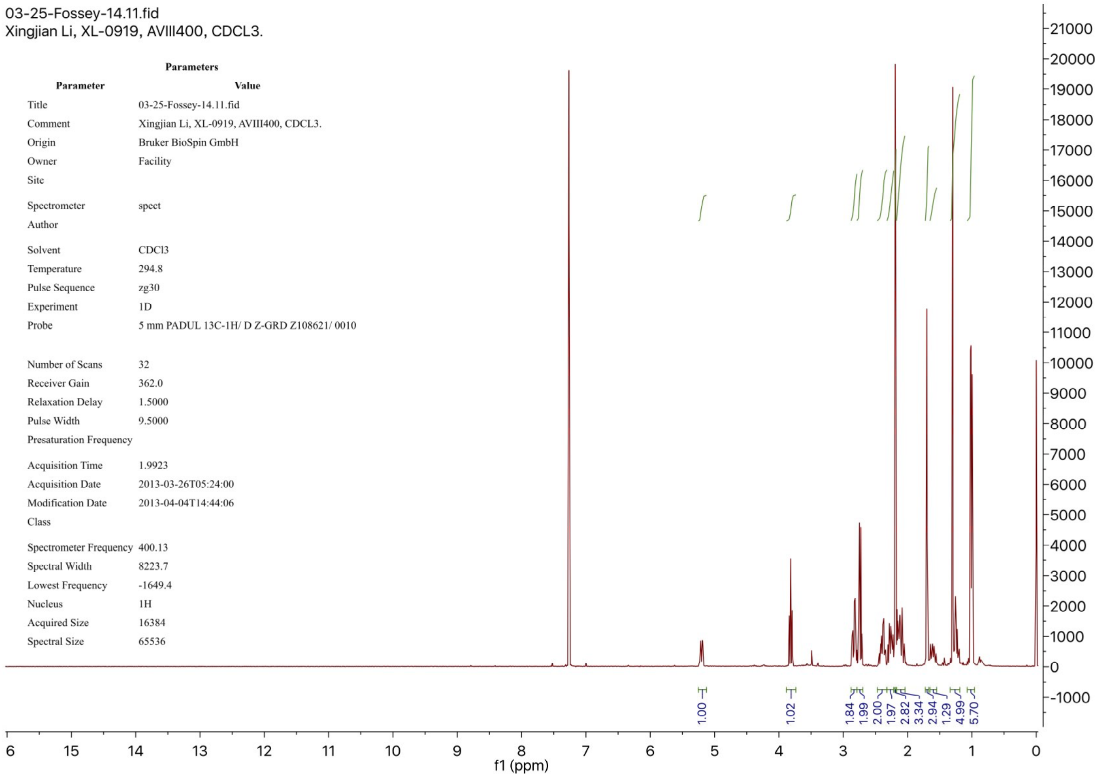

Supplementary Figure 16. *Proton NMR spectrum of* ***2b****.*

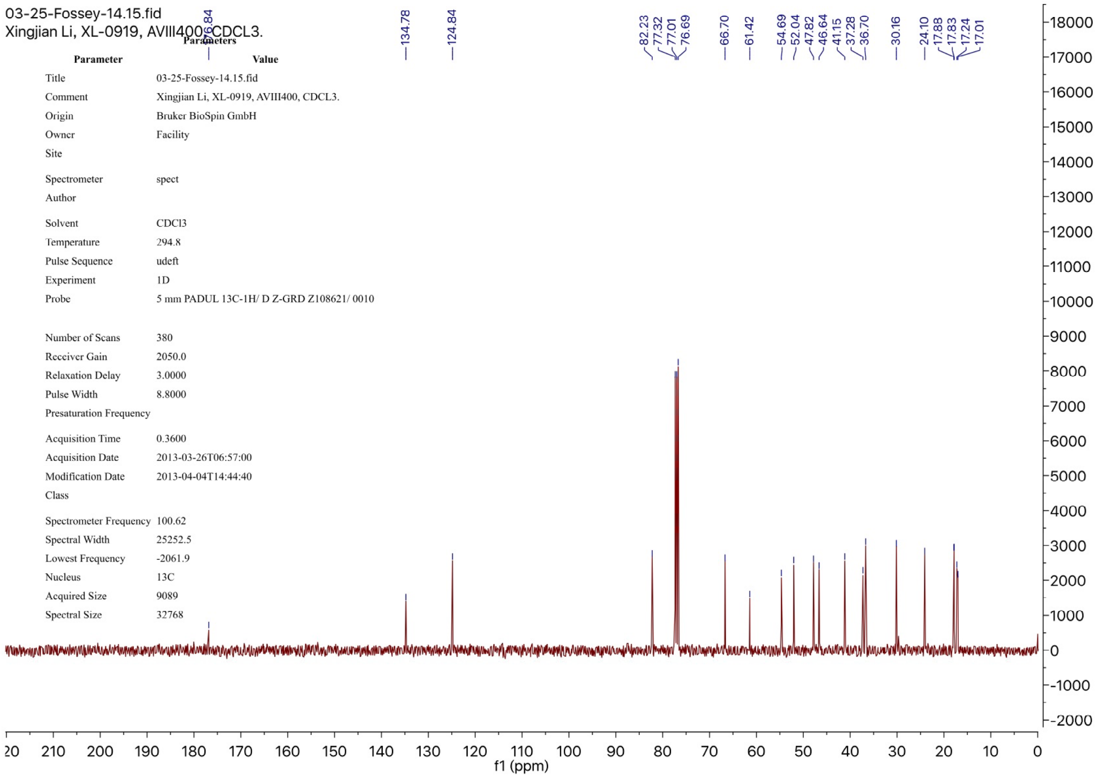

Supplementary Figure 17. *^{13}C NMR spectrum of* ***2b****.*

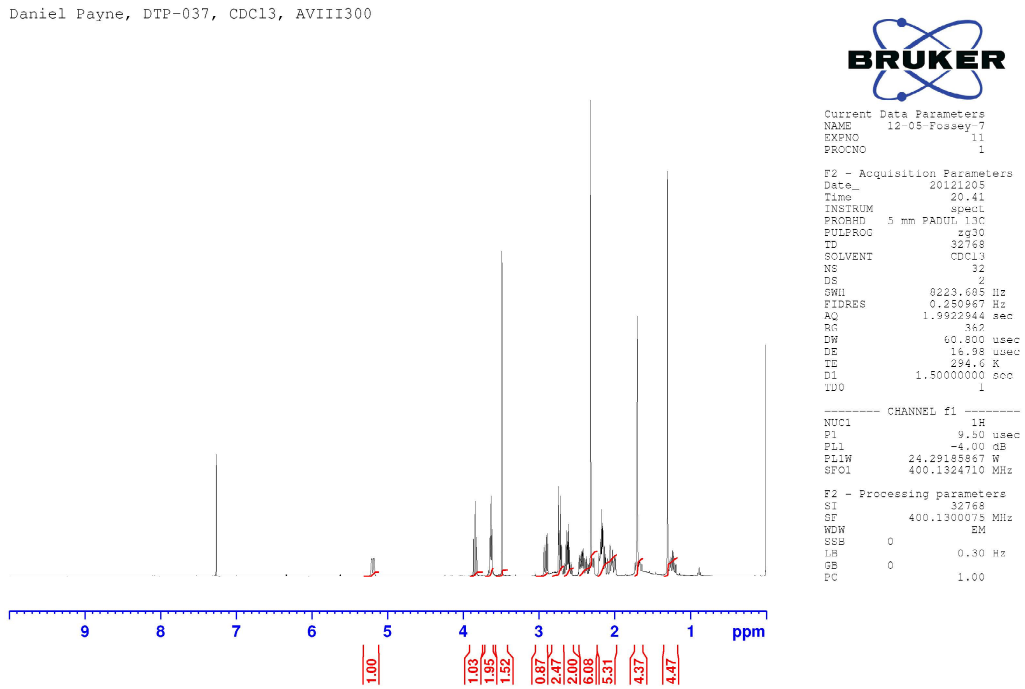

Supplementary Figure 18. *Proton NMR spectrum of* ***2c****.*

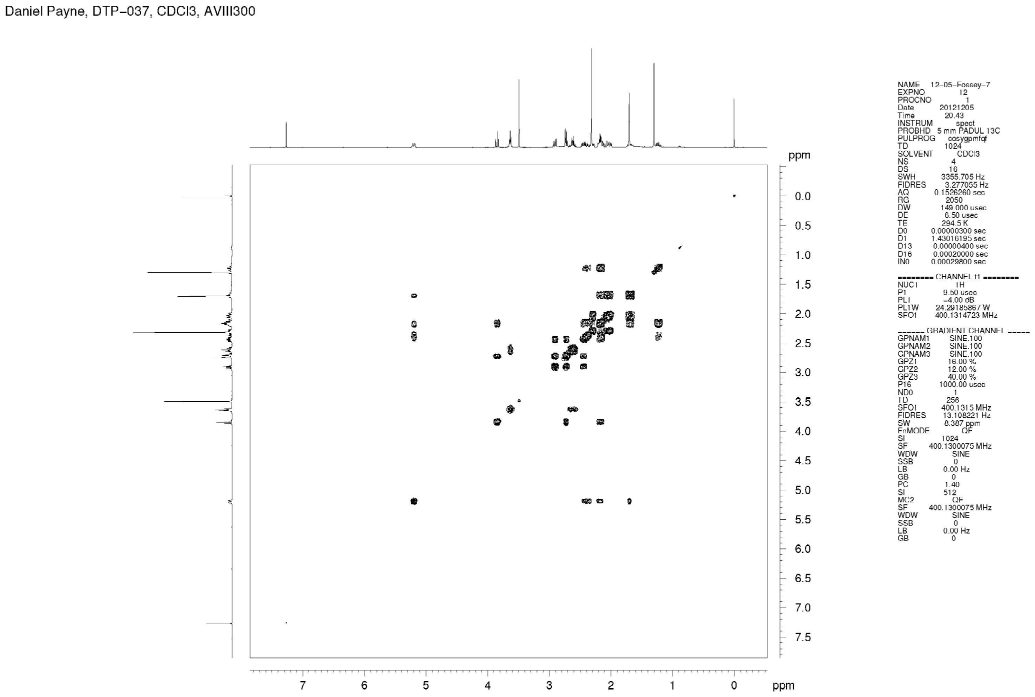

Supplementary Figure 19. *^{1}H-^{1}H COSY NMR spectrum of* ***2c****.*

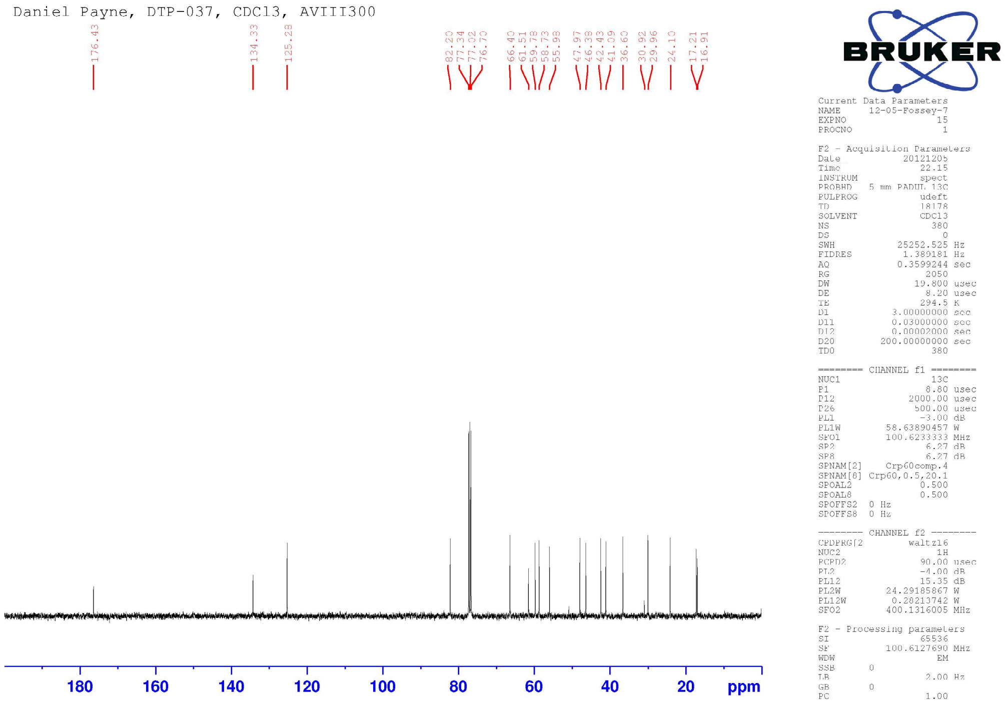

Supplementary Figure 20. ^{13}C NMR spectrum of **2c**.

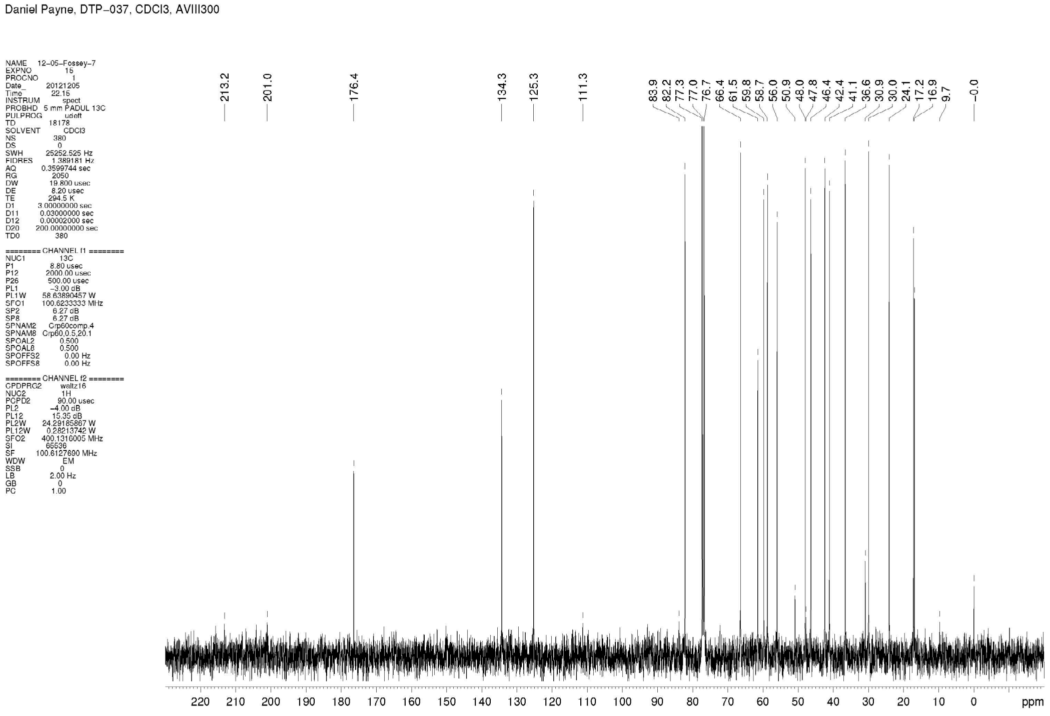

Supplementary Figure 21. UDEFT NMR spectrum of **2c**.

Daniel Payne, DTP–037, CDCl3, AVIII300

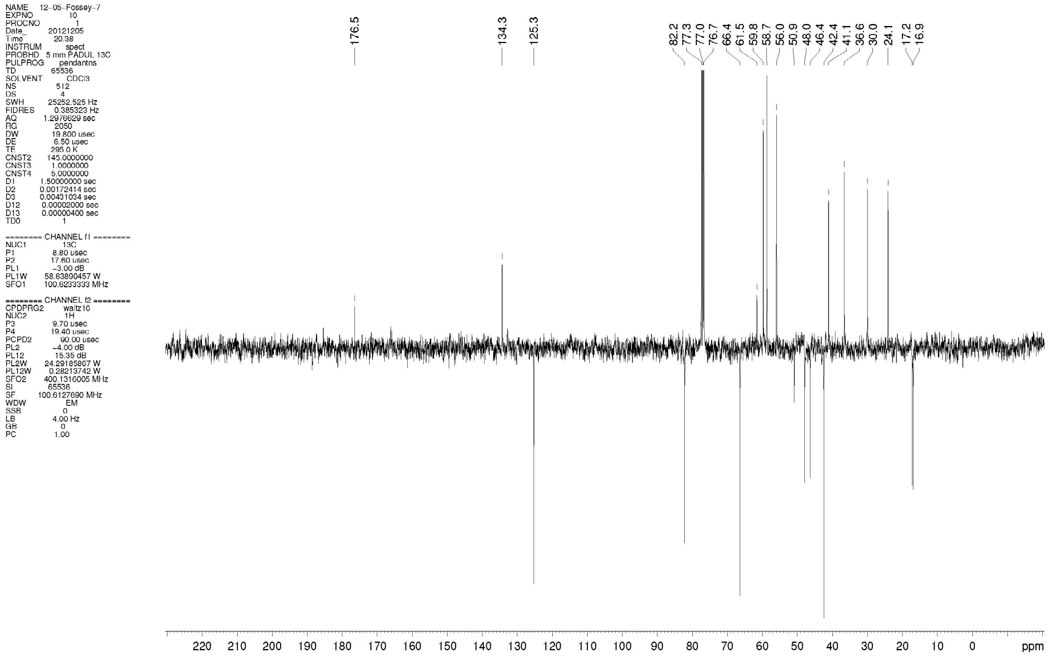

Supplementary Figure 22. *PENDANT NMR spectrum of* ***2c****.*

Daniel Payne, DTP–037, CDCl3, AVIII300

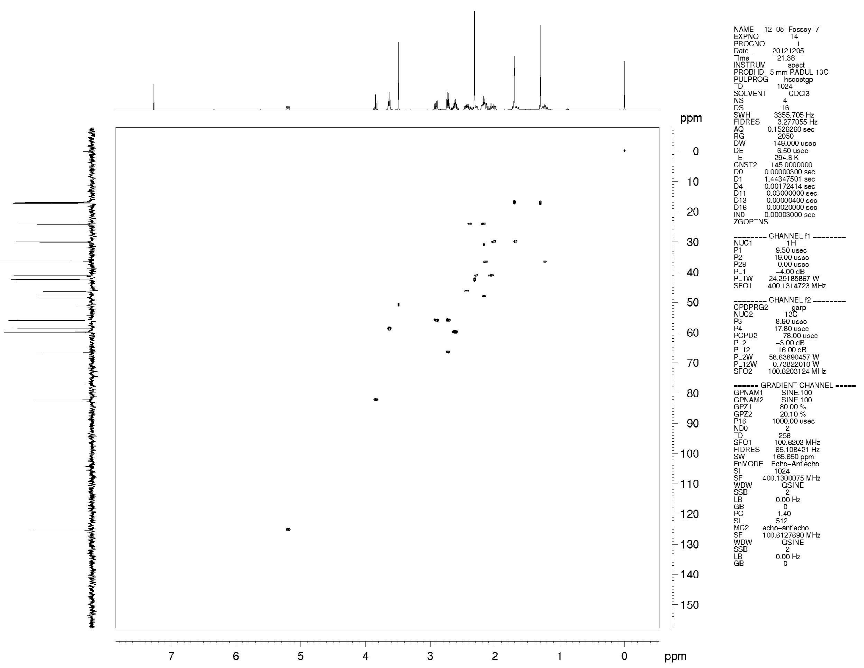

Supplementary Figure 23. *HSQC NMR spectrum of* ***2c****.*

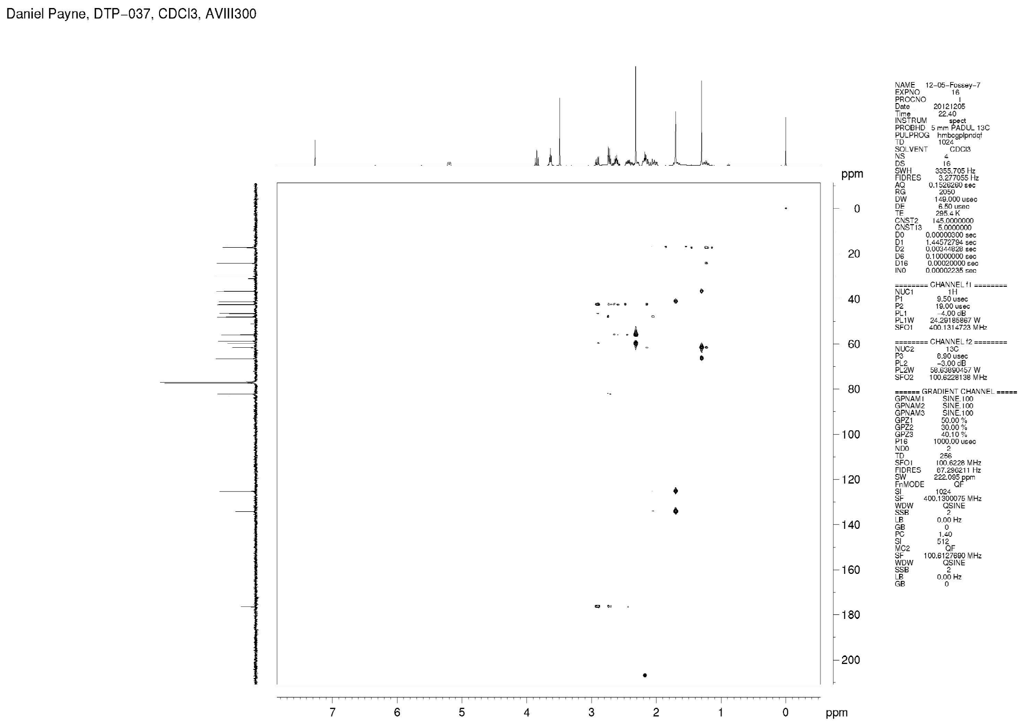

Supplementary Figure 24. *HMBC NMR spectrum of* ***2c****.*

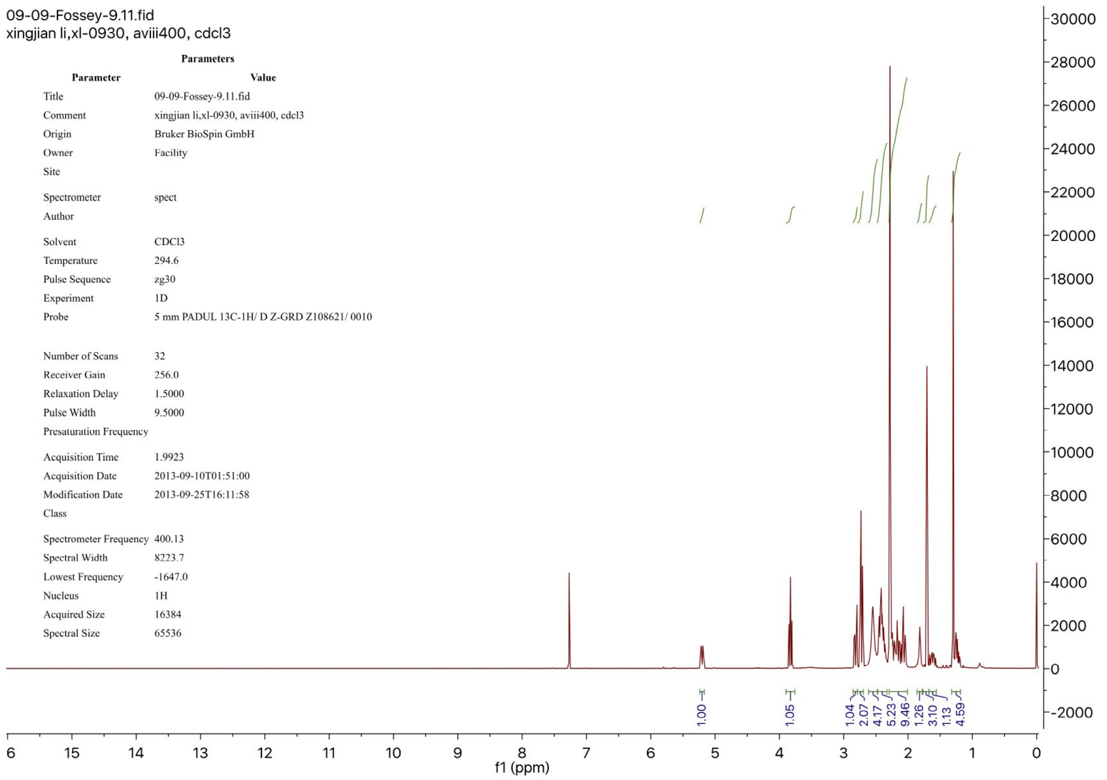

Supplementary Figure 25. Proton NMR spectrum of **2d**.

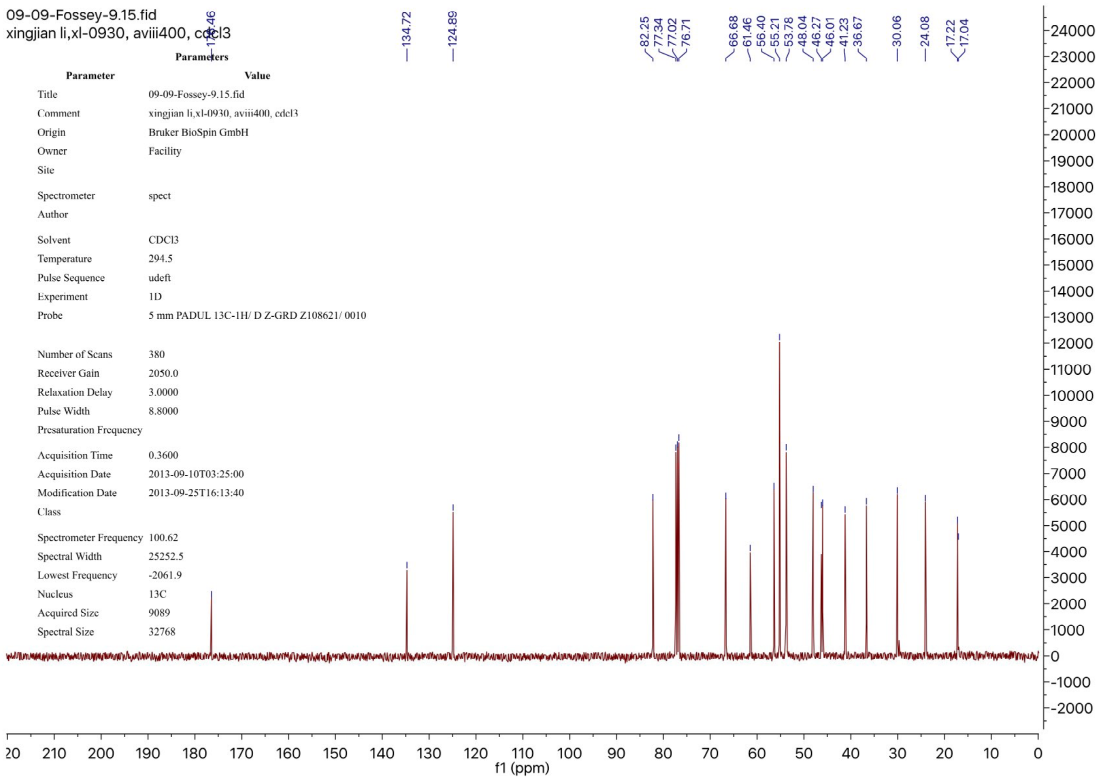

Supplementary Figure 26. ^{13}C NMR spectrum of **2d**.

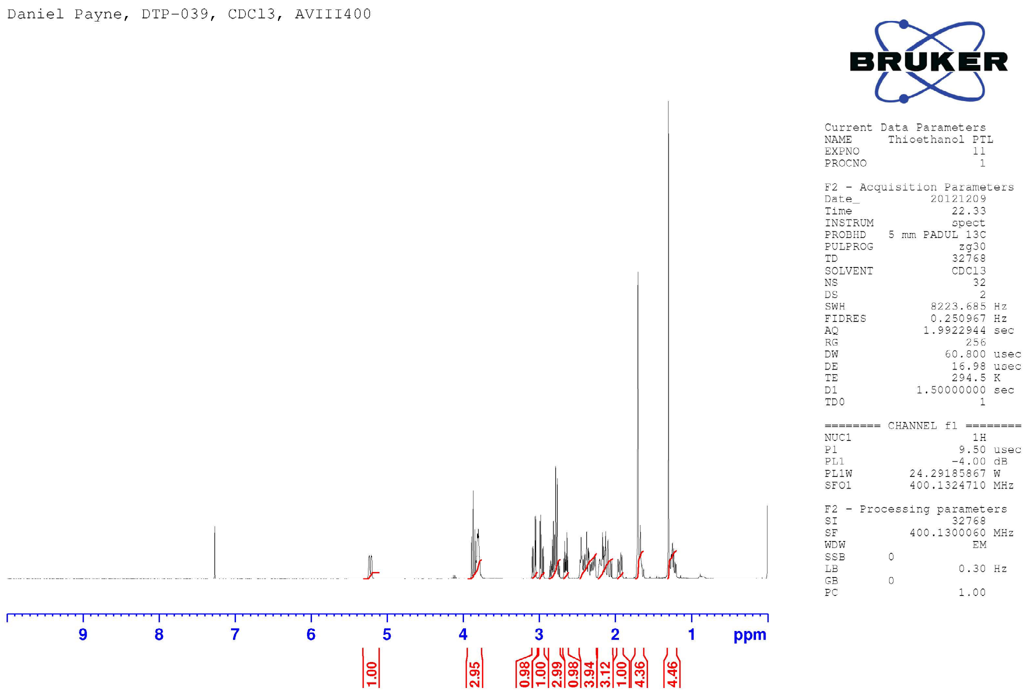

Supplementary Figure 27. Proton NMR spectrum of **3**.

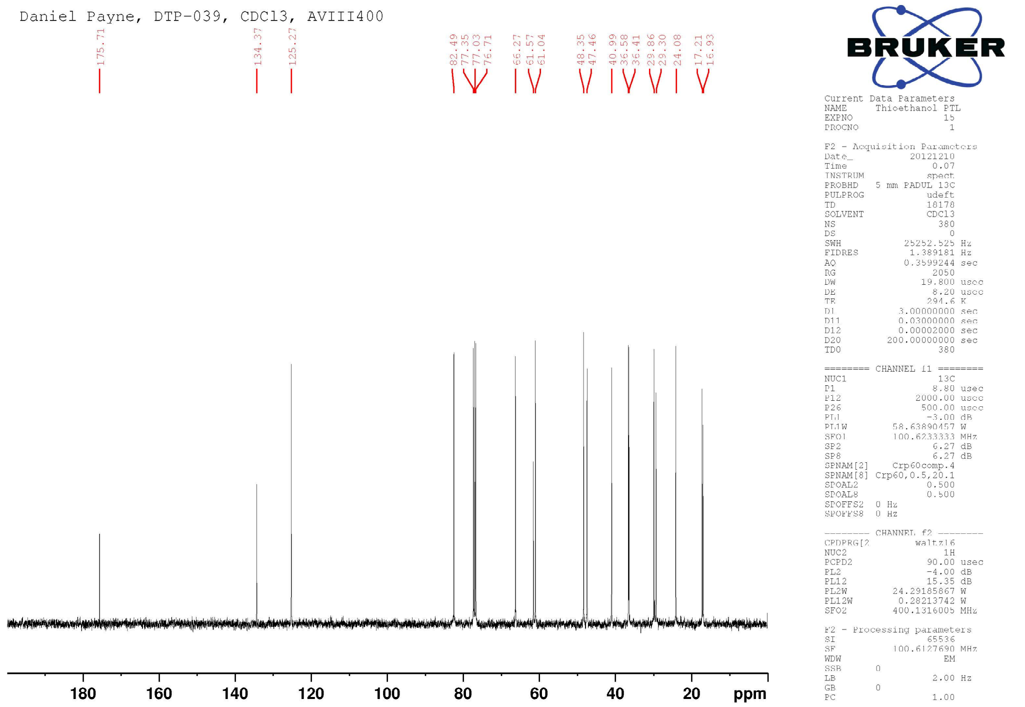

Supplementary Figure 28. ^{13}C NMR spectrum of **3**.

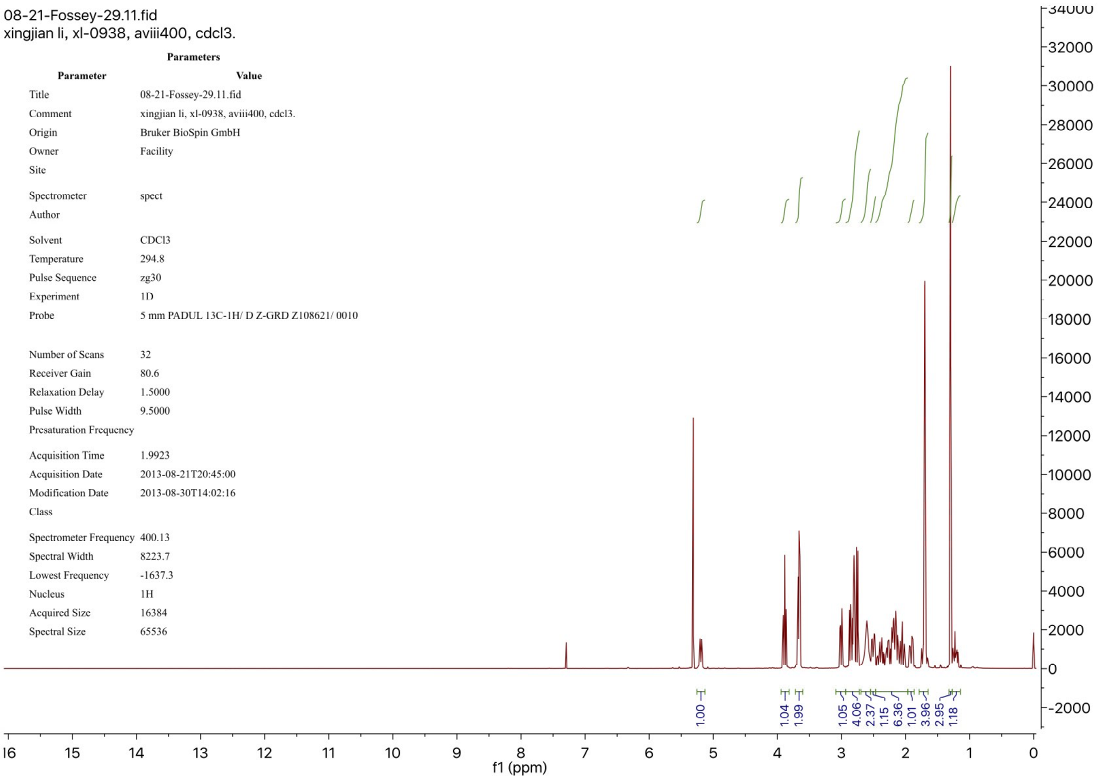

Supplementary Figure 29. Proton NMR spectrum of **4a**.

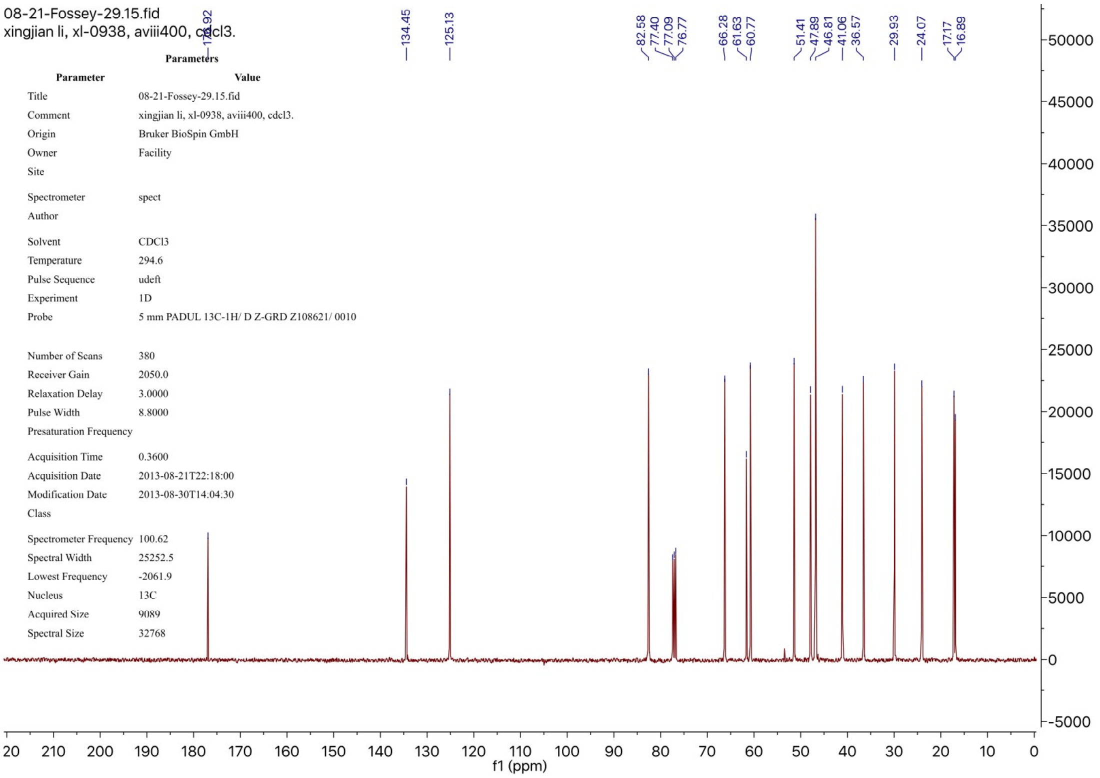

Supplementary Figure 30. ^{13}C NMR spectrum of **4a**.

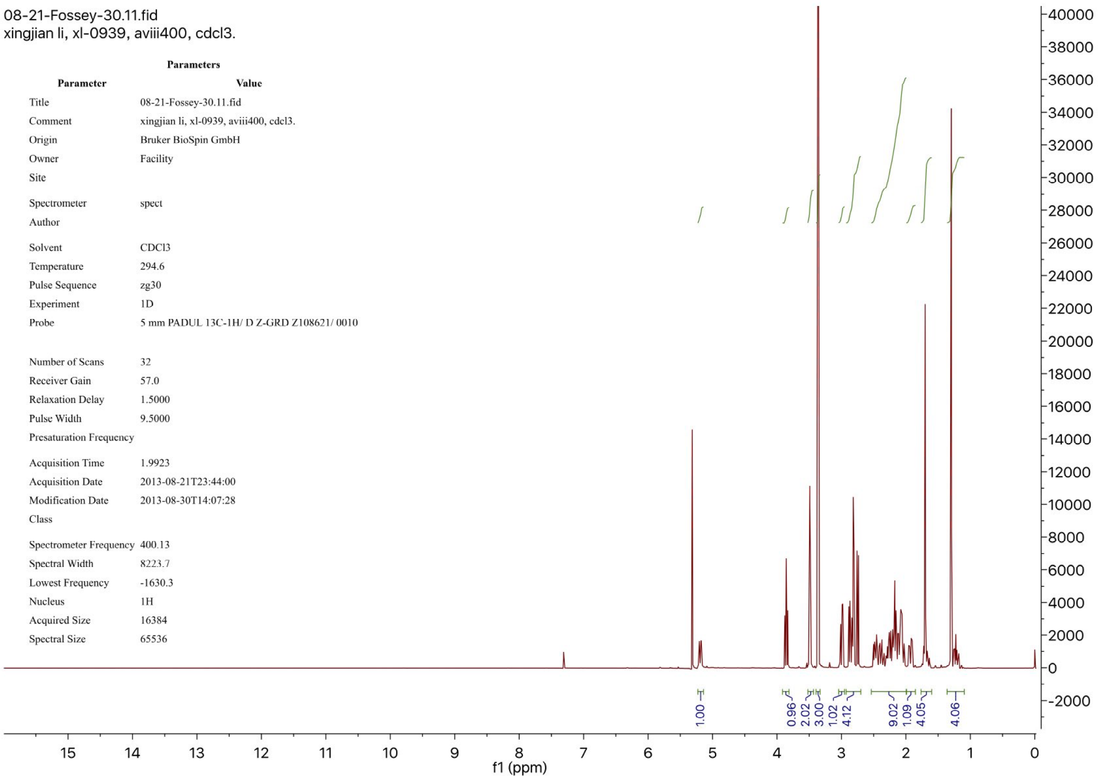

Supplementary Figure 31. *Proton NMR spectrum of* ***4b****.*

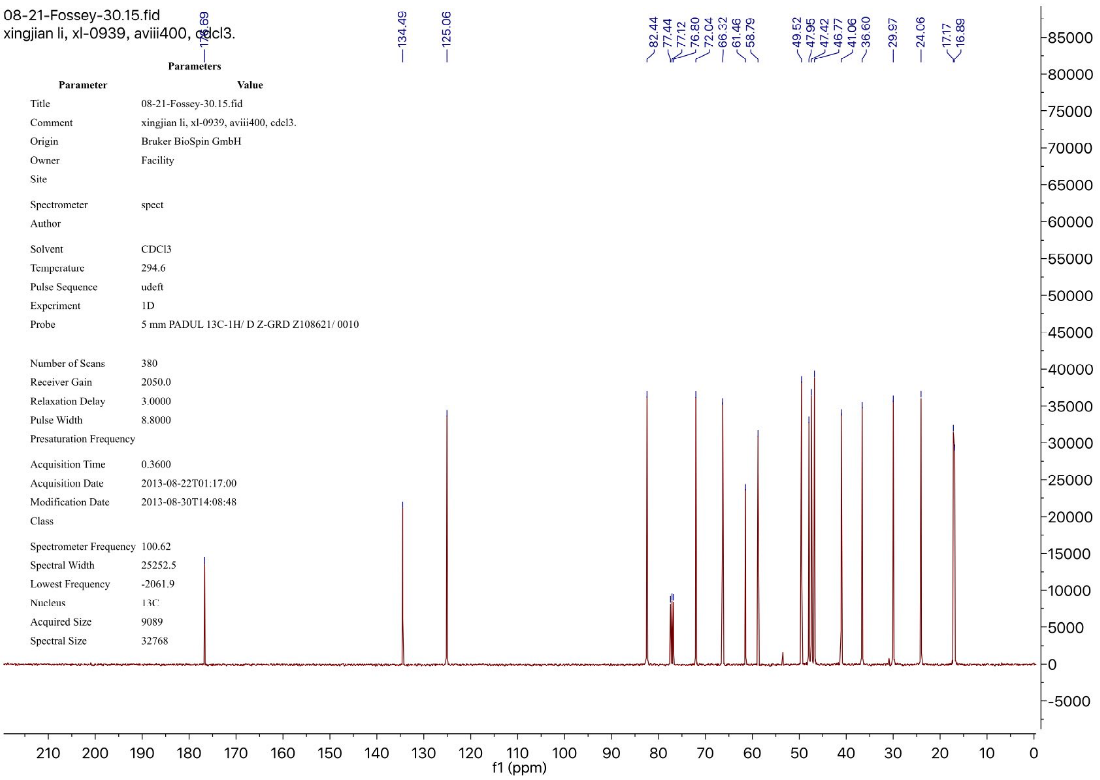

Supplementary Figure 32. *^{13}C NMR spectrum of* ***4b****.*

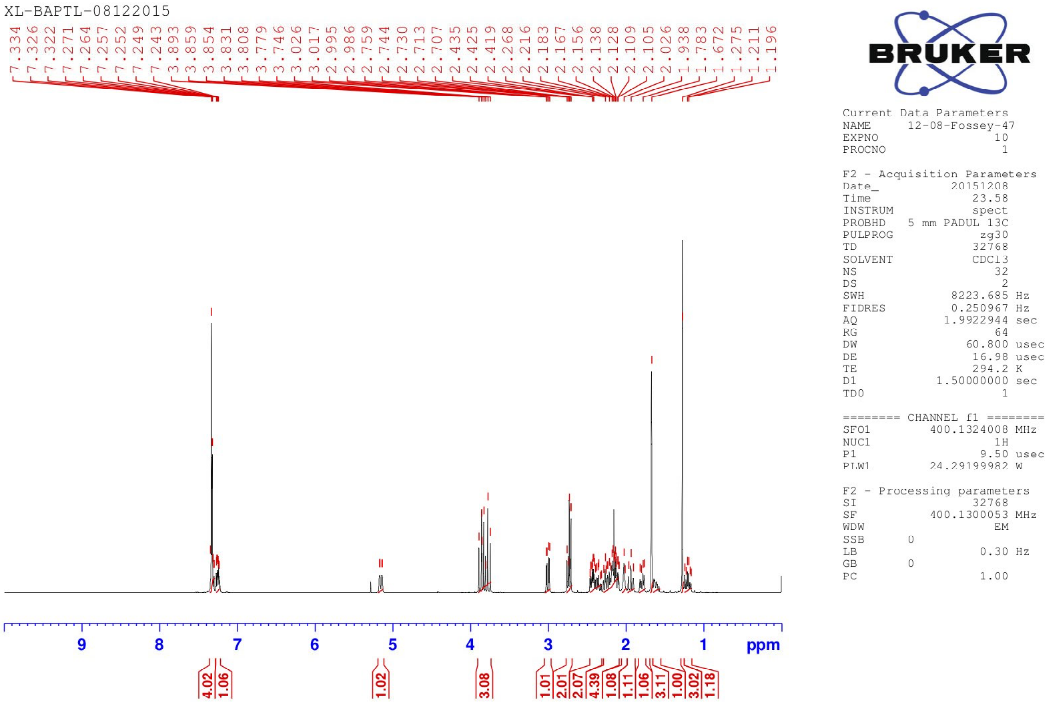

Supplementary Figure 33. *Proton NMR spectrum of* ***4c****.*

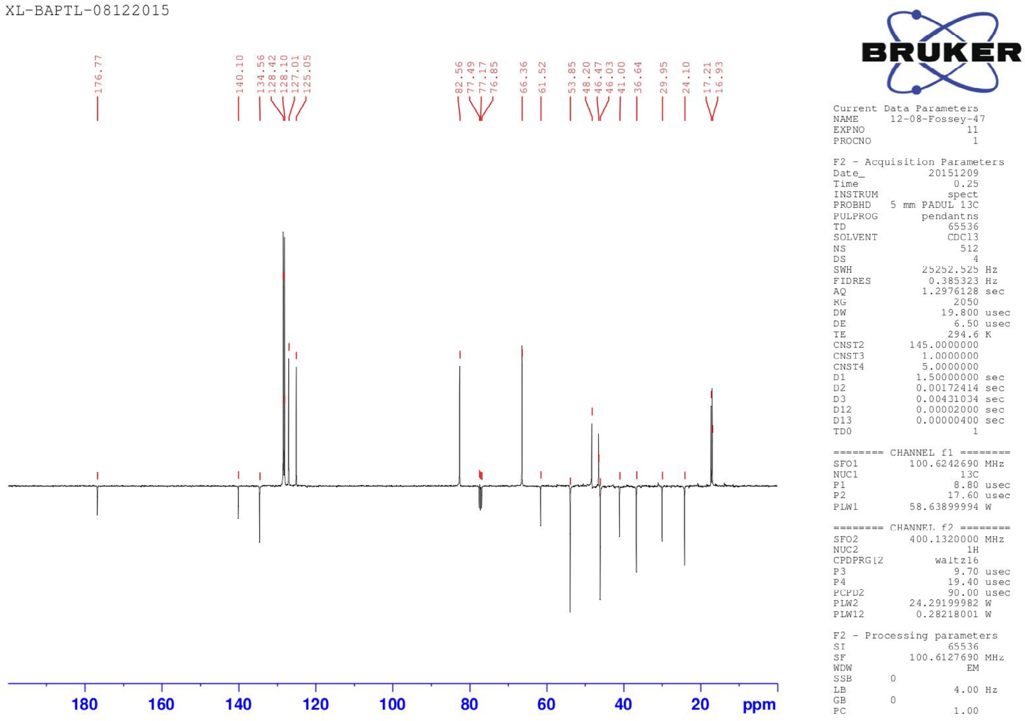

Supplementary Figure 34. *^{13}C (PENDANT) NMR spectrum of* ***4c****.*

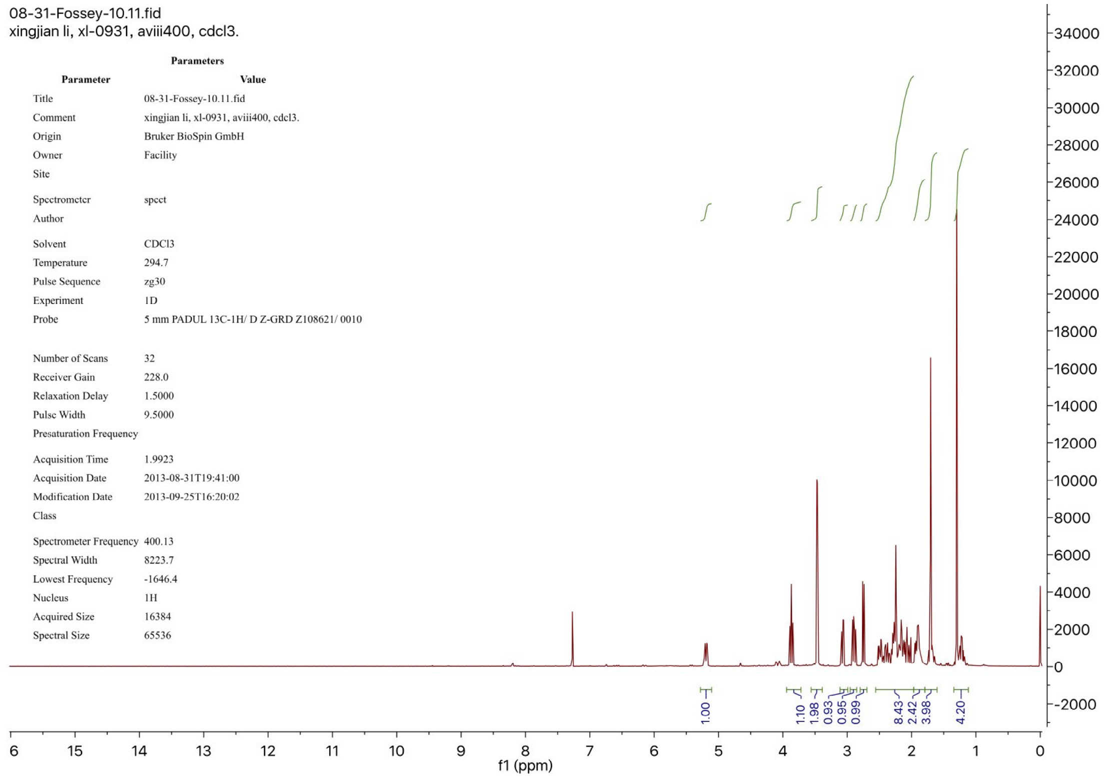

Supplementary Figure 35. *Proton NMR spectrum of **4d**.*

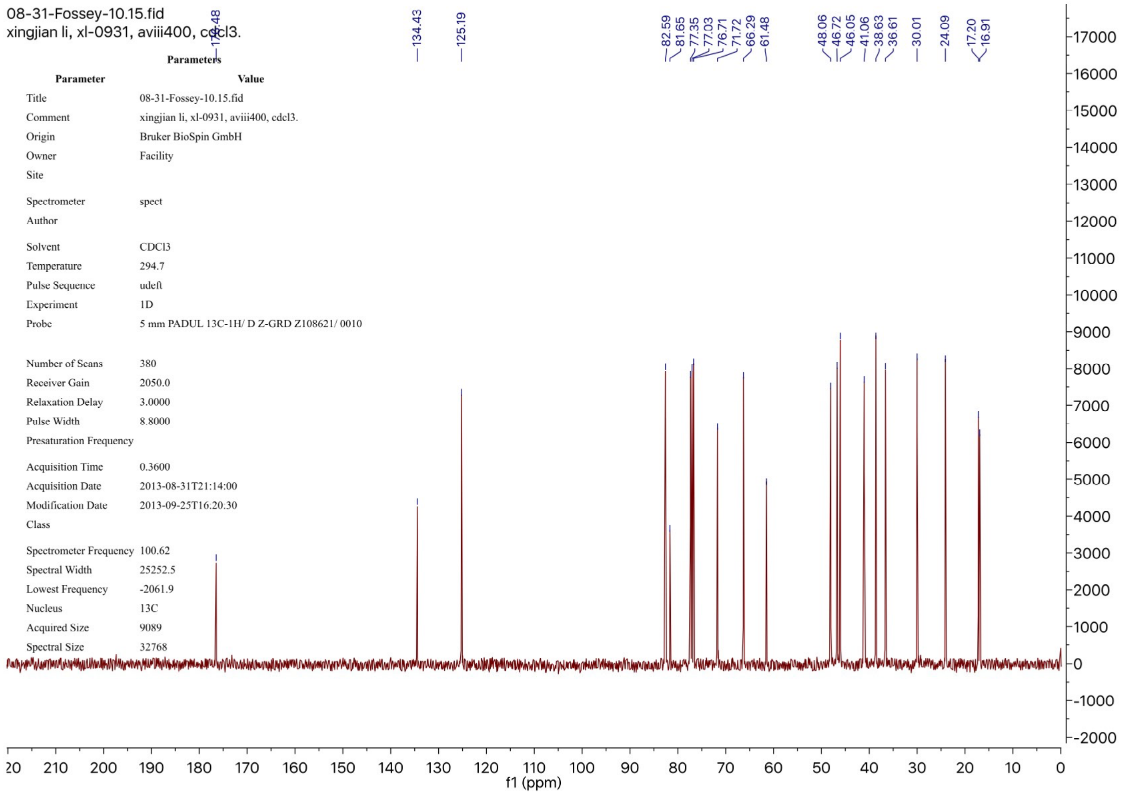

Supplementary Figure 36. *^{13}C NMR spectrum of **4d**.*

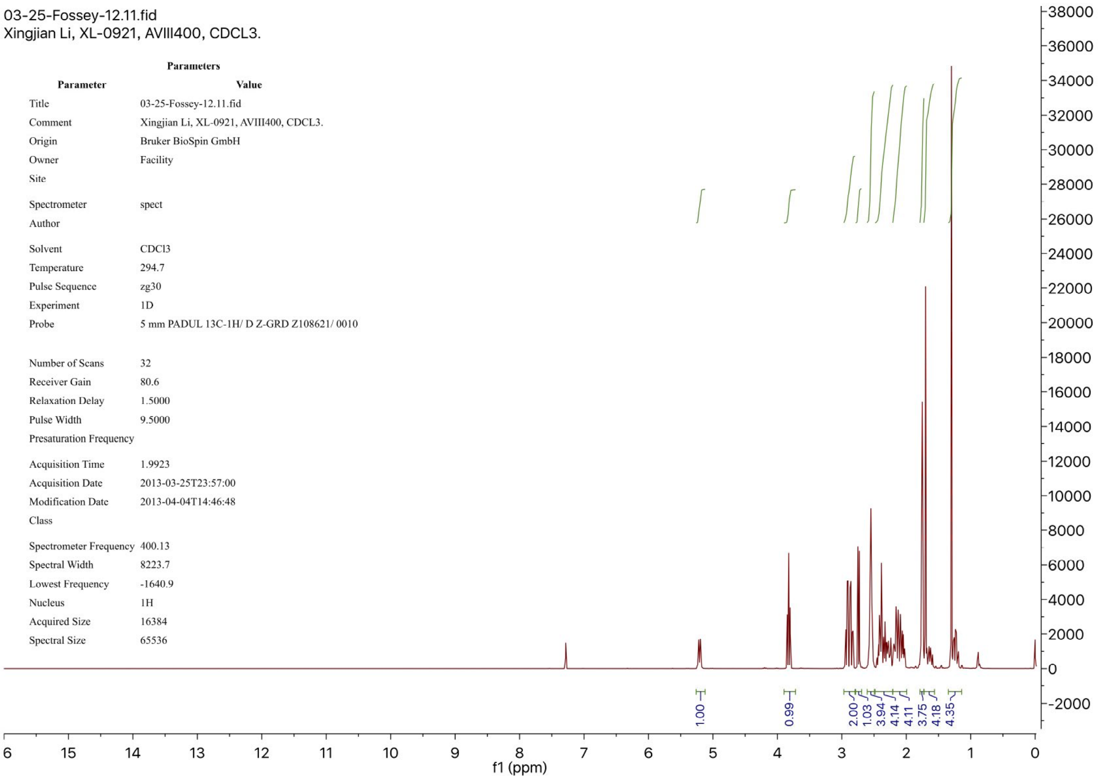

Supplementary Figure 37. *Proton NMR spectrum of* ***5a****.*

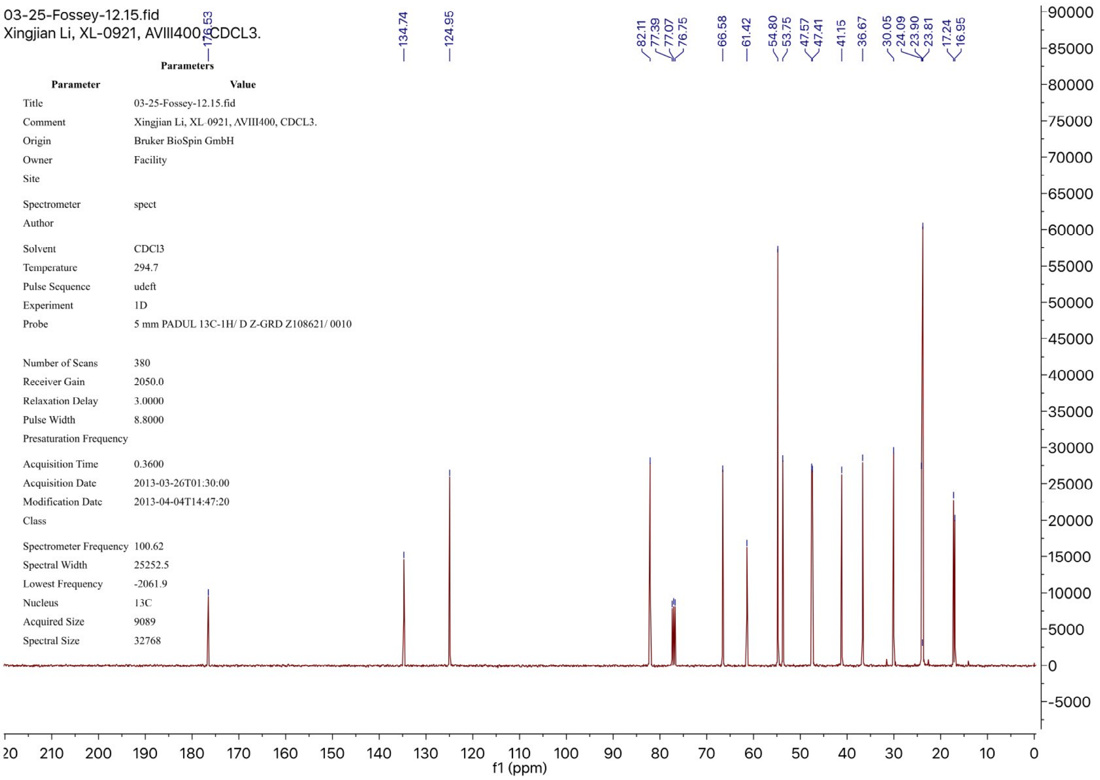

Supplementary Figure 38. *^{13}C NMR spectrum of* ***5a****.*

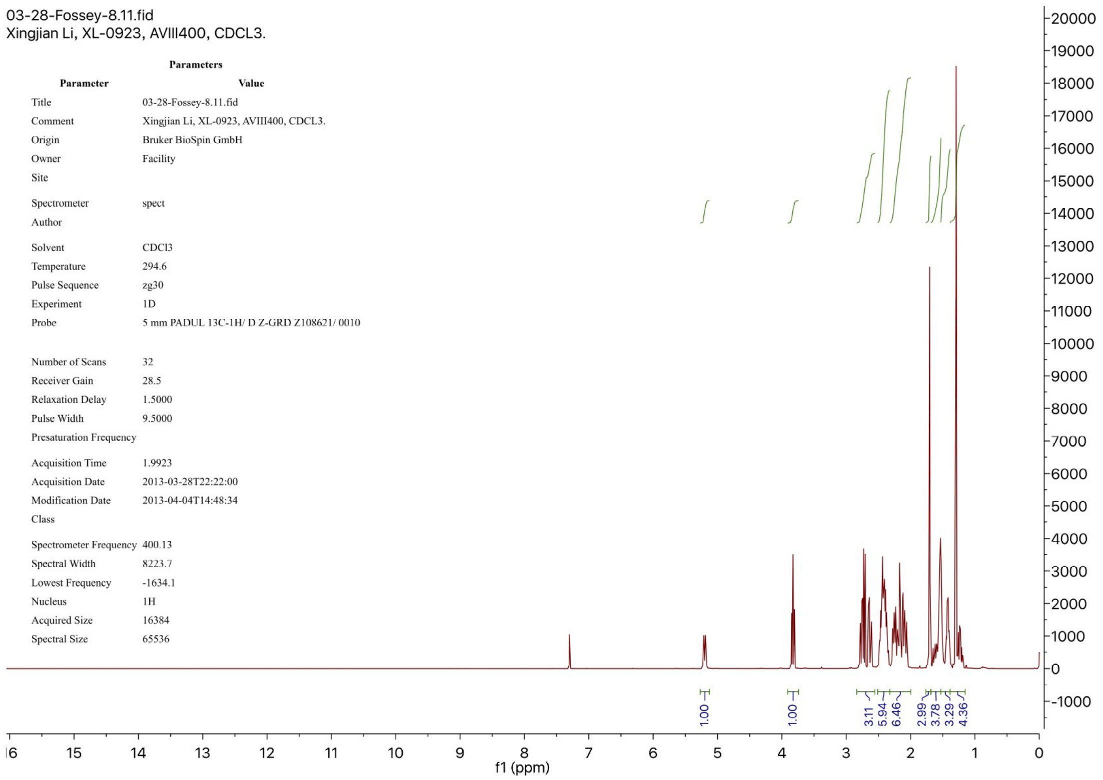

Supplementary Figure 39. *Proton NMR spectrum of **5b**.*

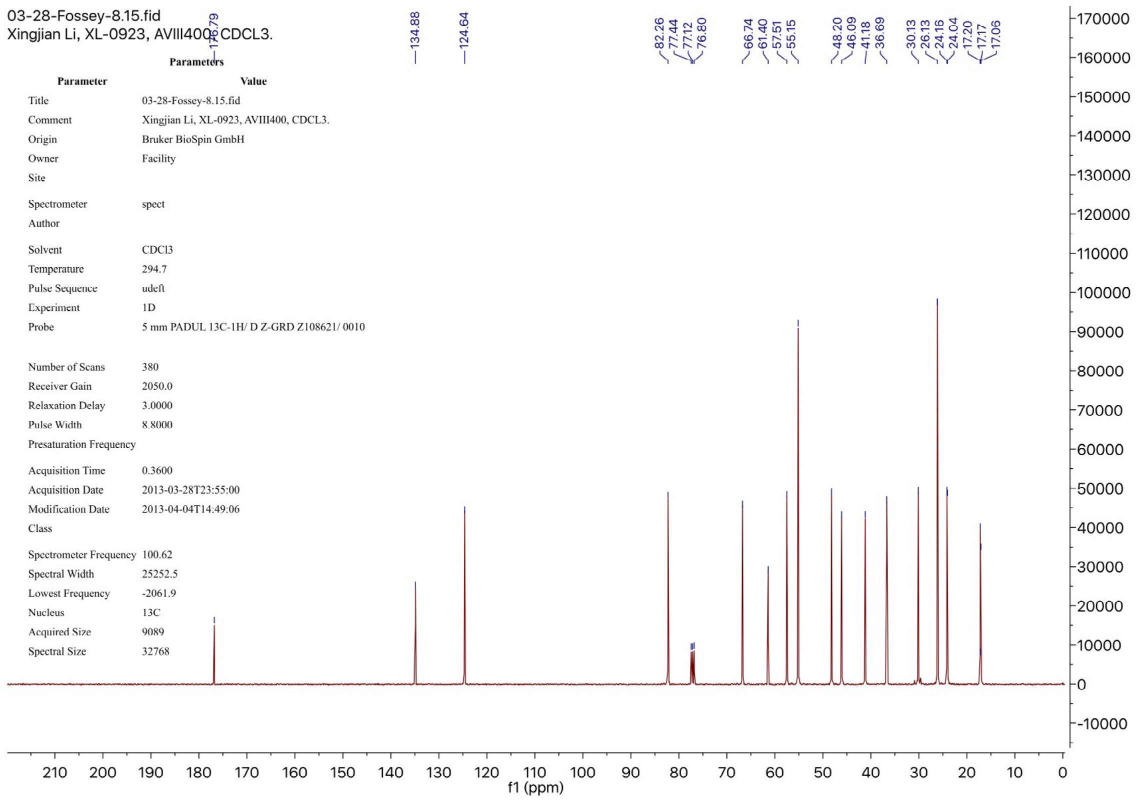

Supplementary Figure 40. *^{13}C NMR spectrum of **5b**.*

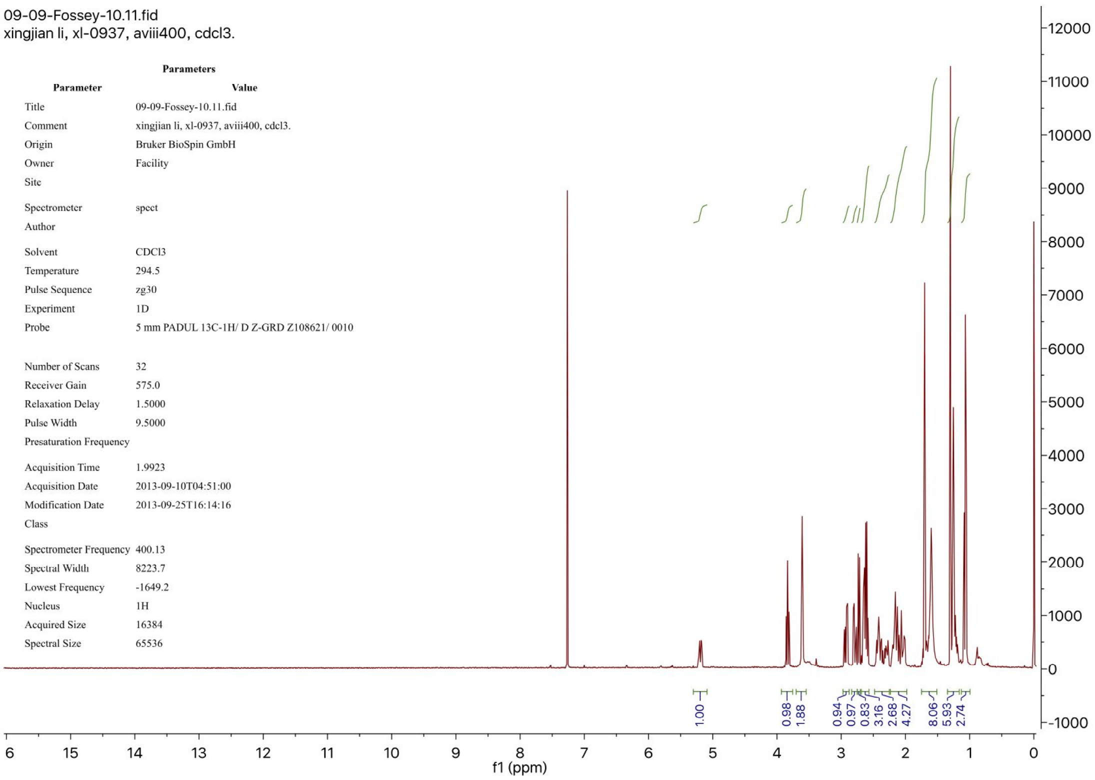

Supplementary Figure 41. *Proton NMR spectrum of **5c**.*

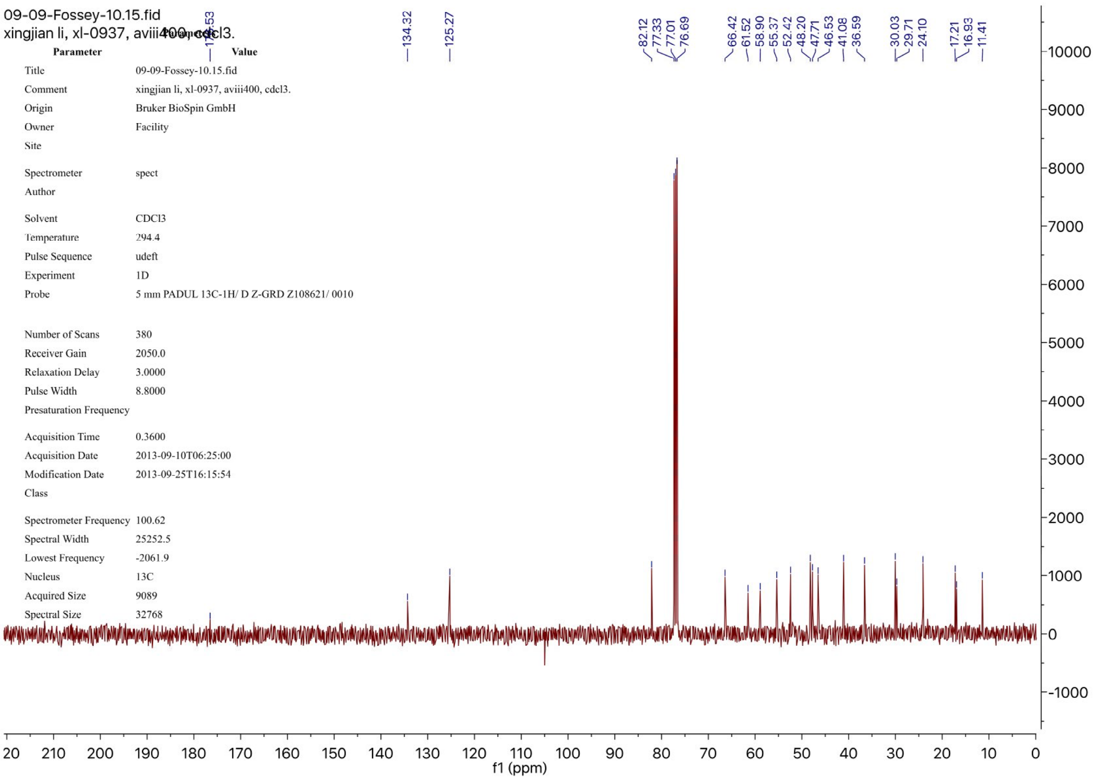

Supplementary Figure 42. *^{13}C NMR spectrum of **5c**.*

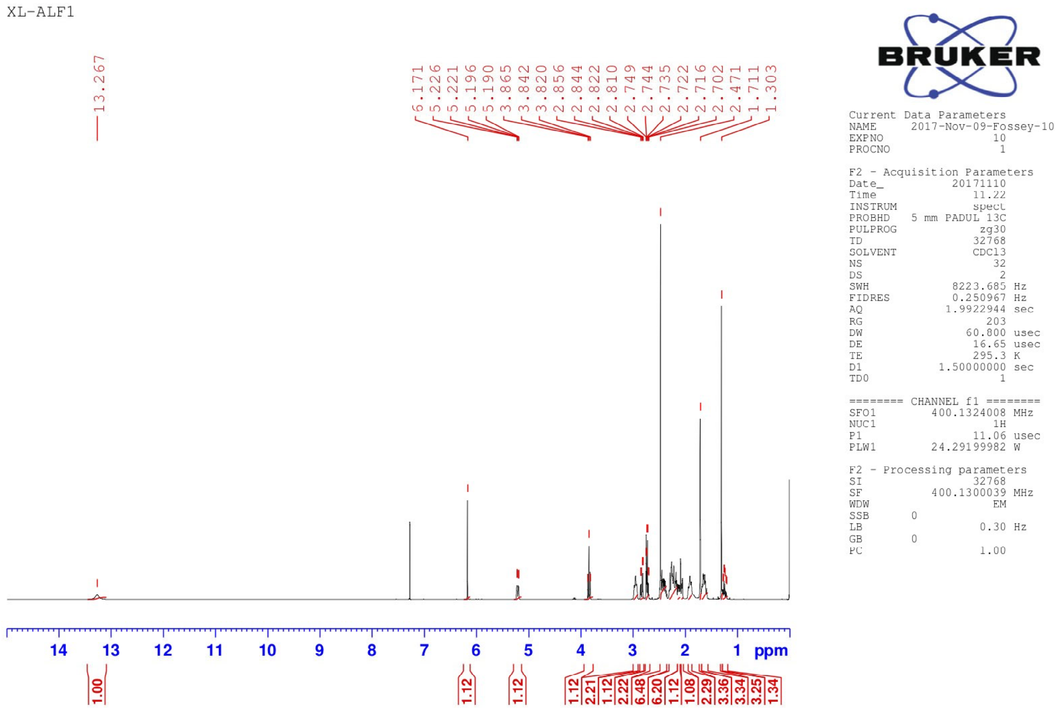

Supplementary Figure 43. *Proton NMR spectrum of* ***5f****.*

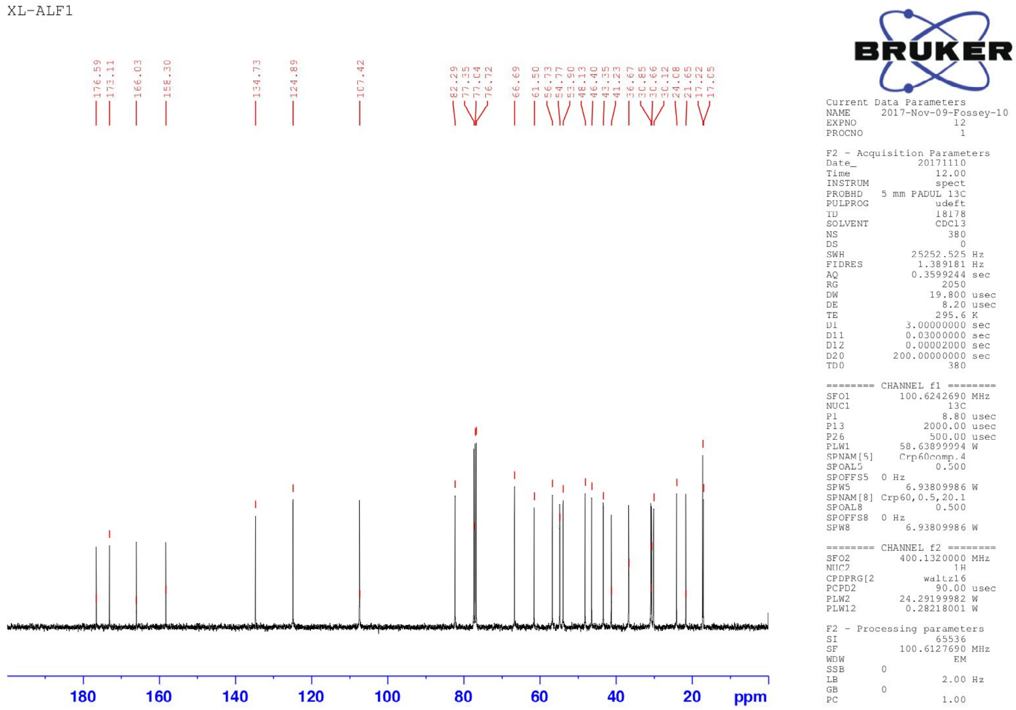

Supplementary Figure 44. *^{13}C (UDEFT) NMR spectrum of* ***5f****.*

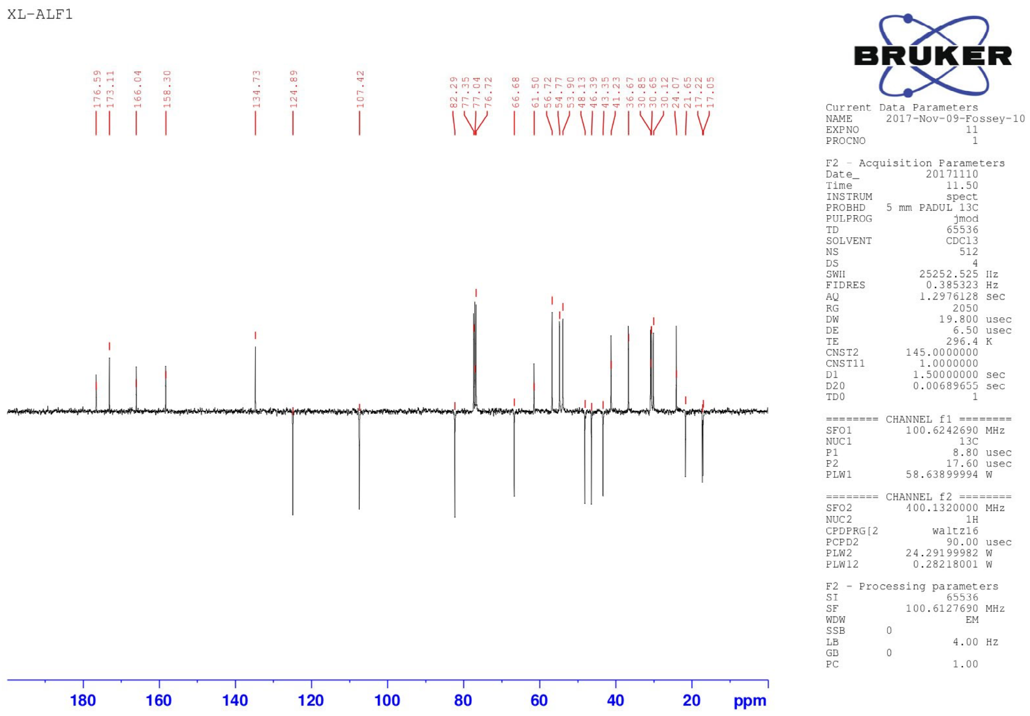

Supplementary Figure 45. *^{13}C (PENDANT) NMR spectrum of* ***5f****.*

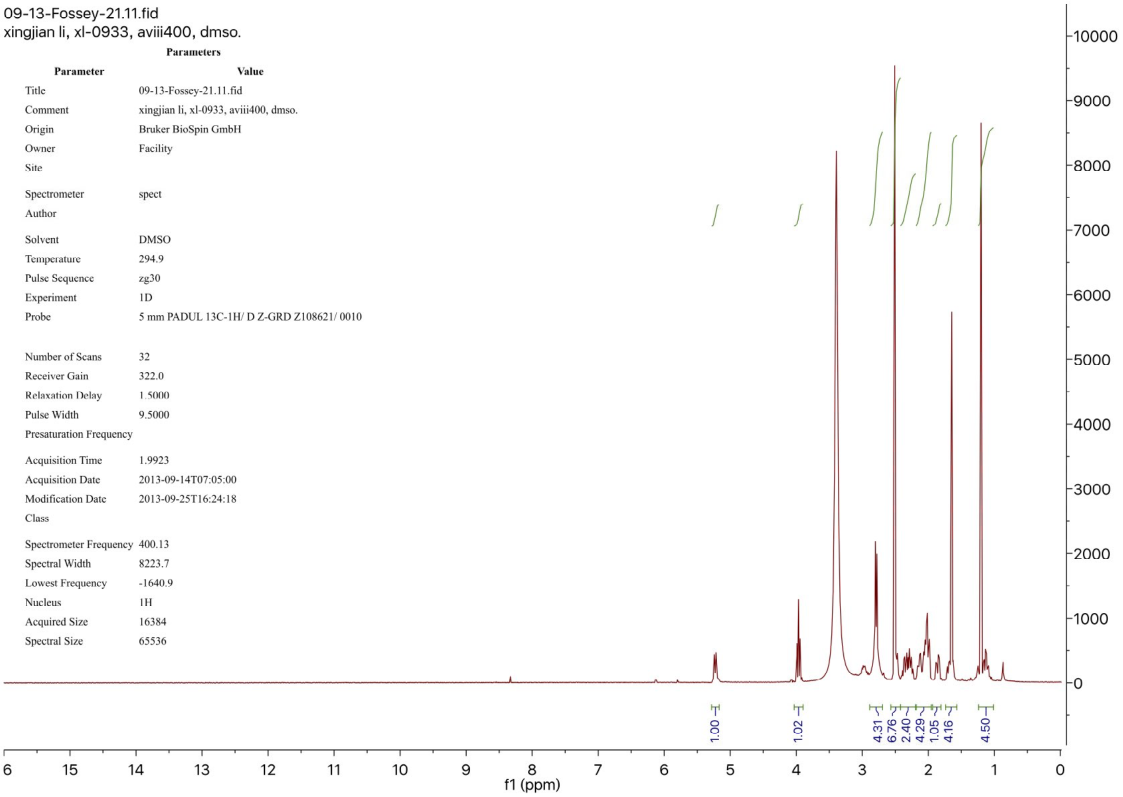

Supplementary Figure 46. *Proton NMR spectrum of* ***6a****.*

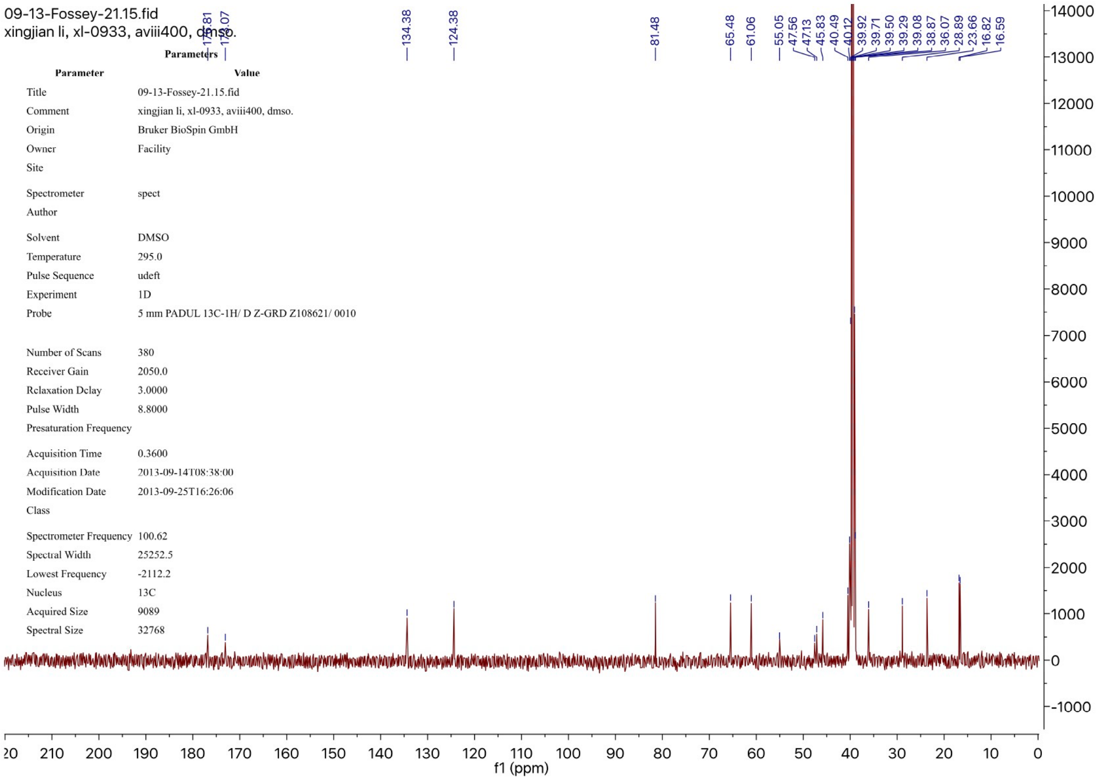

Supplementary Figure 47. *^{13}C NMR spectrum of* ***6a****.*

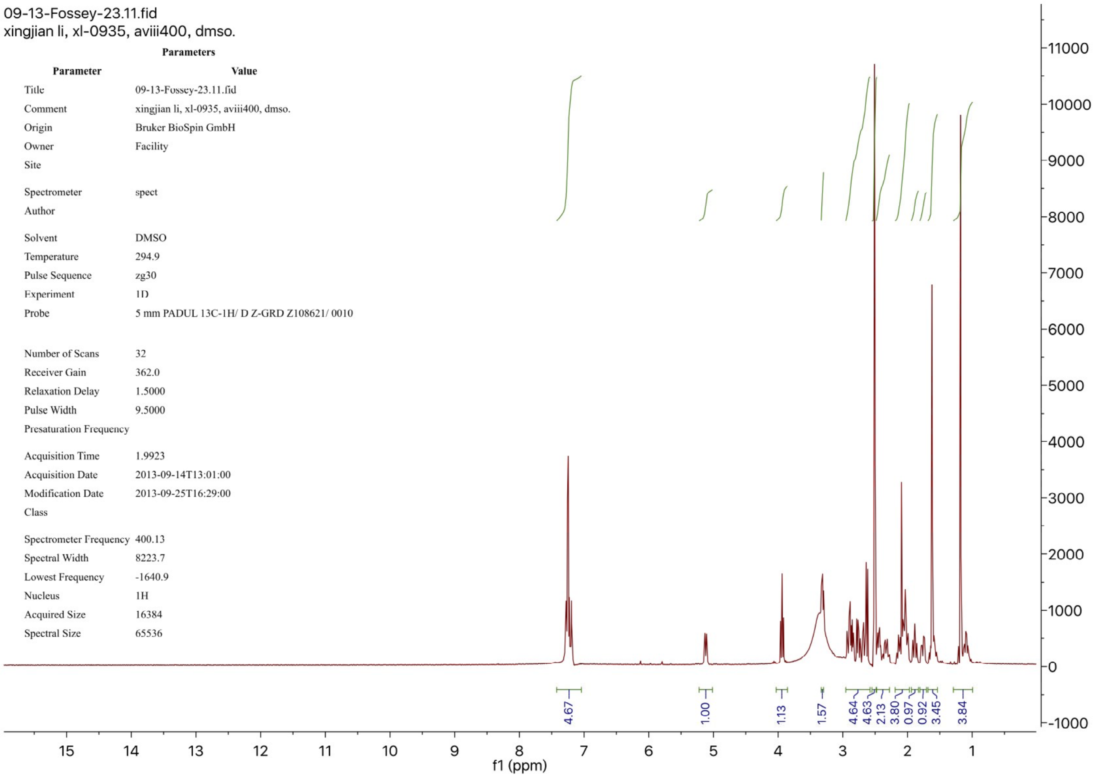

Supplementary Figure 48. *Proton NMR spectrum of* ***6b****.*

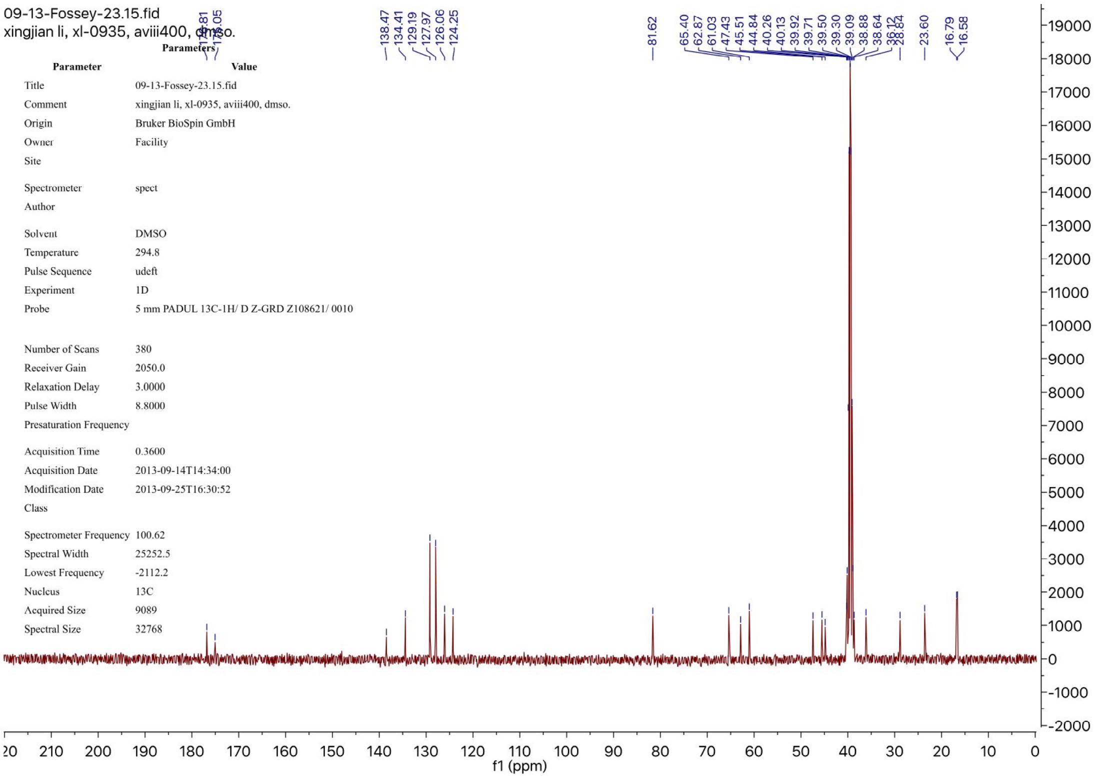

Supplementary Figure 49. *^{13}C NMR spectrum of* ***6b****.*

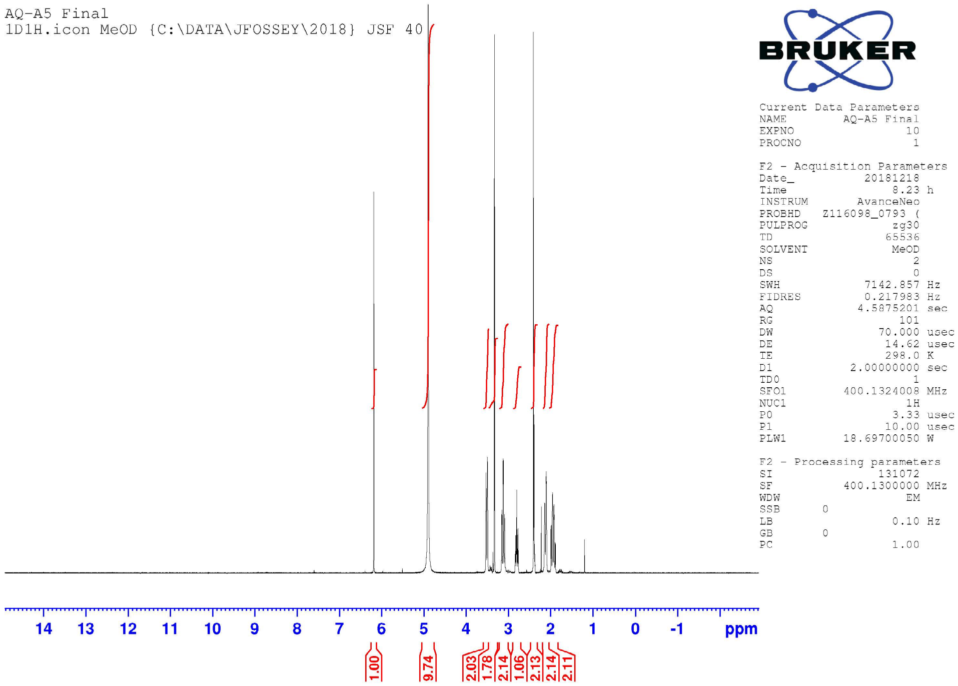

Supplementary Figure 50. *Proton NMR spectrum of 7.*

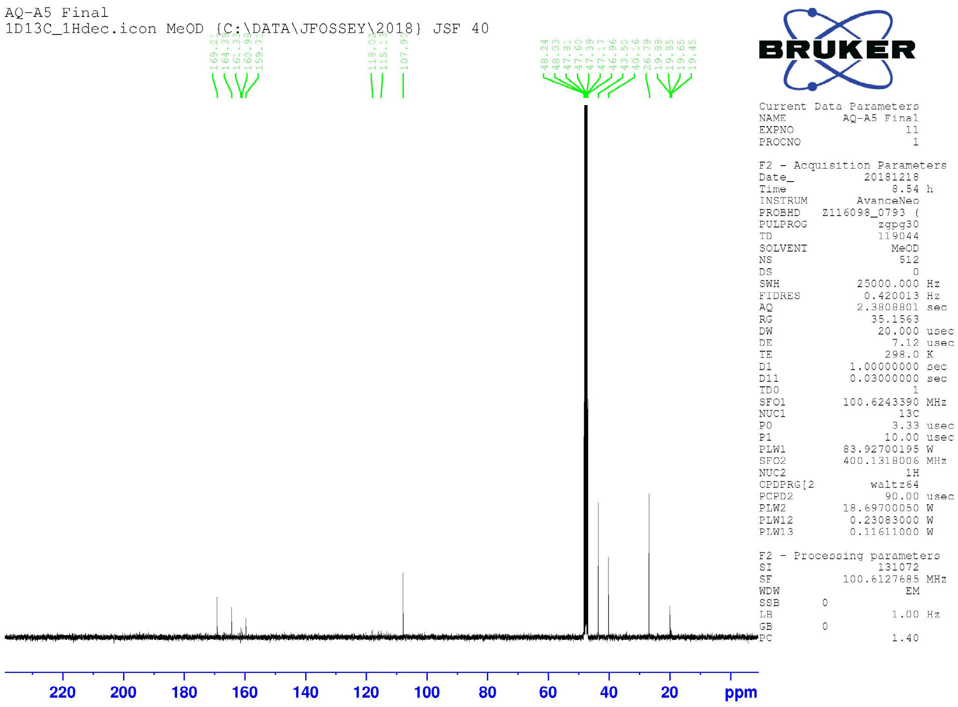

Supplementary Figure 51. *^{13}C NMR spectrum of 7.*

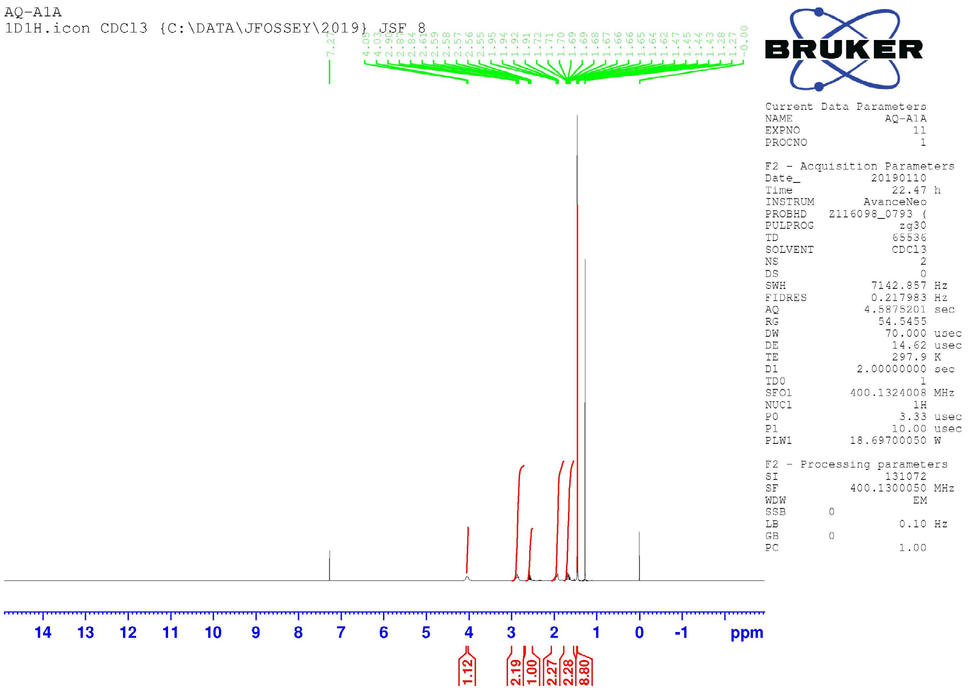

Supplementary Figure 52. *Proton NMR spectrum of* ***8****.*

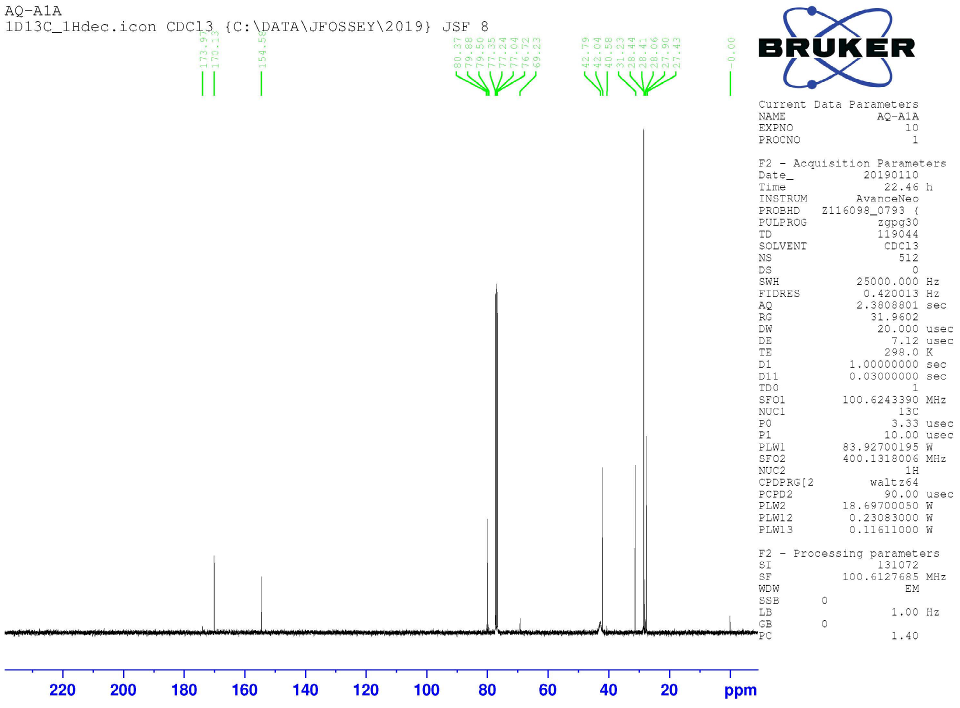

Supplementary Figure 53. *^{13}C NMR spectrum of* ***8****.*

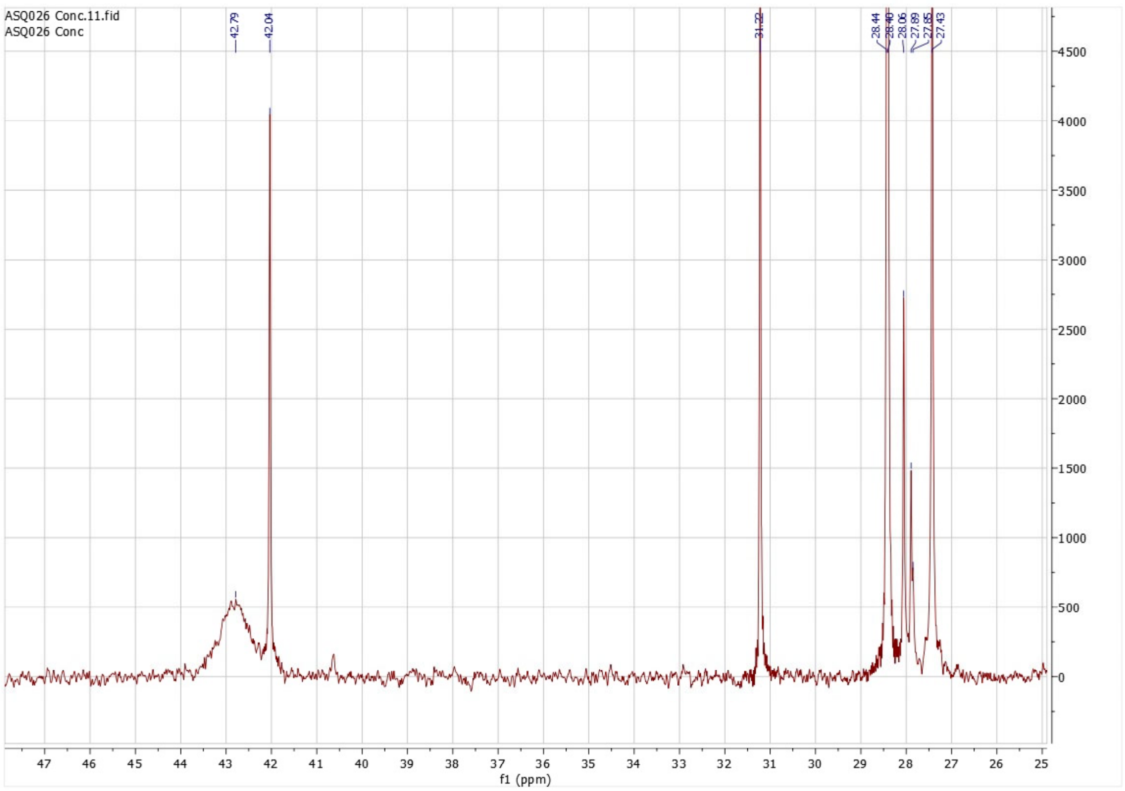

Supplementary Figure 54. *Magnified region of* ^{13}C *NMR spectrum displaying broad resonance centred on 42.8 ppm N(***C***H₂)₂.*

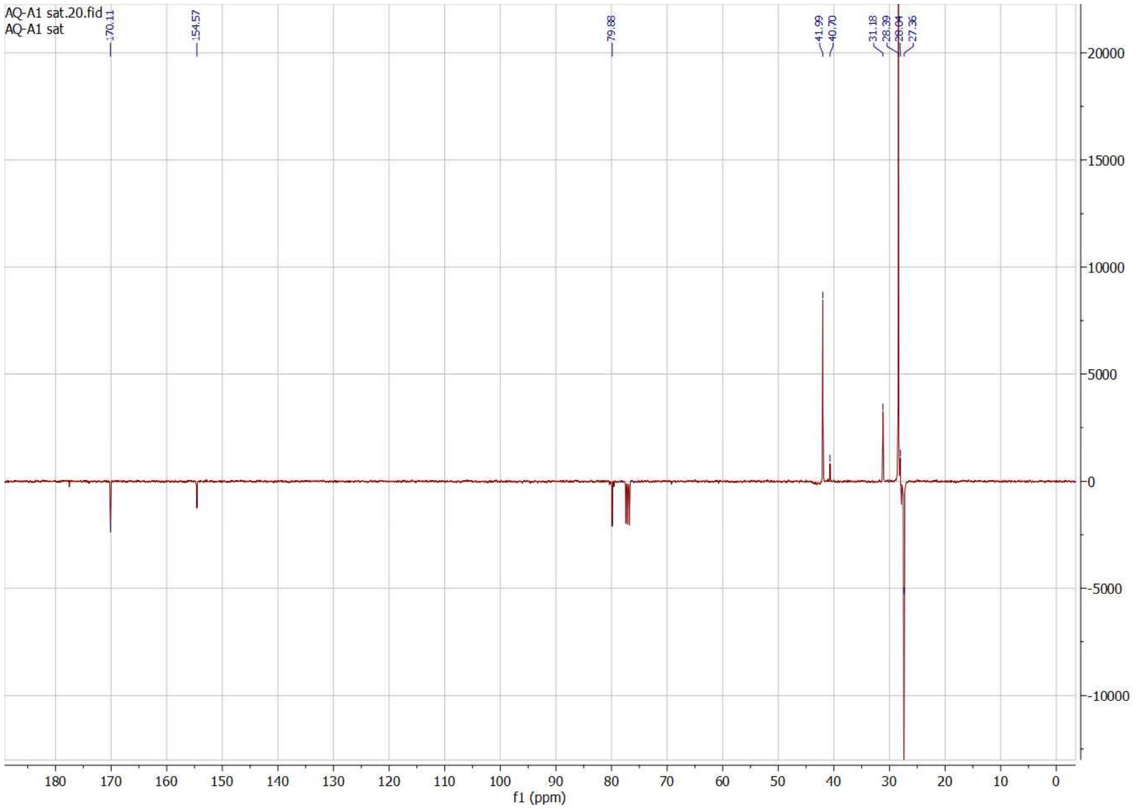

Supplementary Figure 55. ^{13}C *(J-MOD) spectrum of compound* ***8****.*

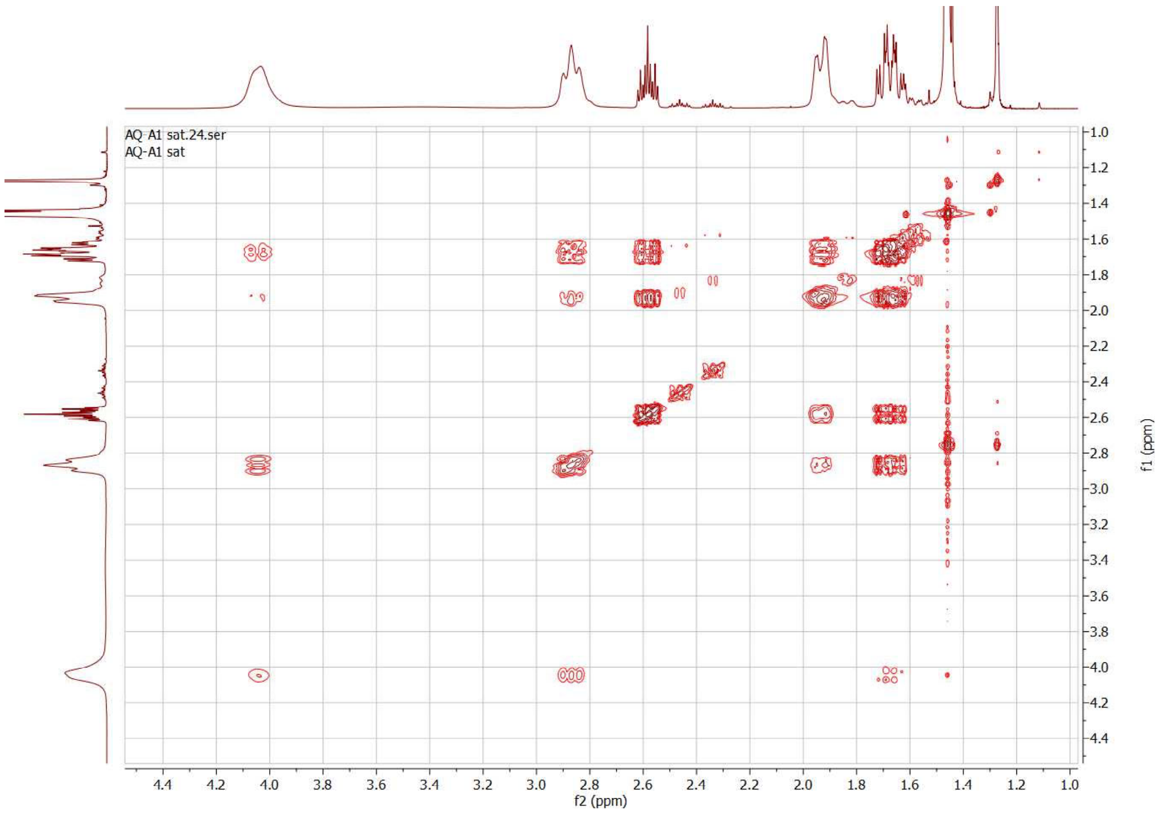

Supplementary Figure 56. *Region of the proton-proton 2D COSY spectrum of* ***8****.*

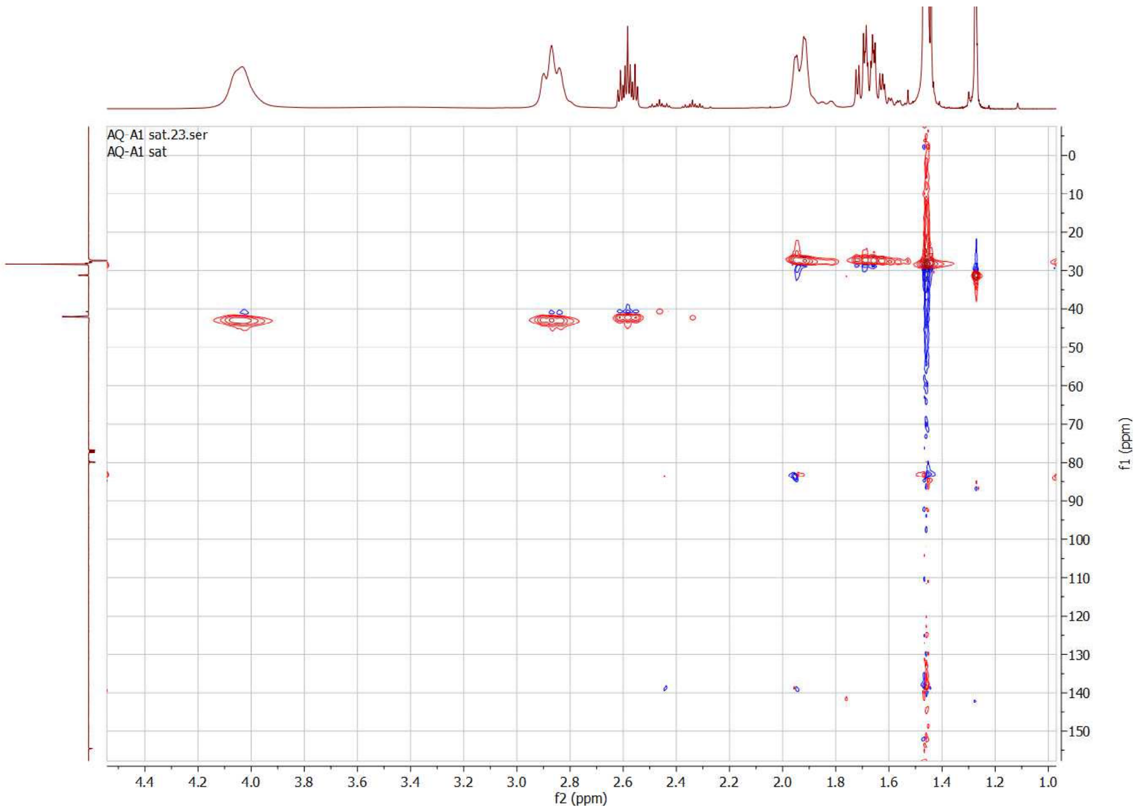

Supplementary Figure 57. *2D-HSQC spectrum of compound* ***8****.*

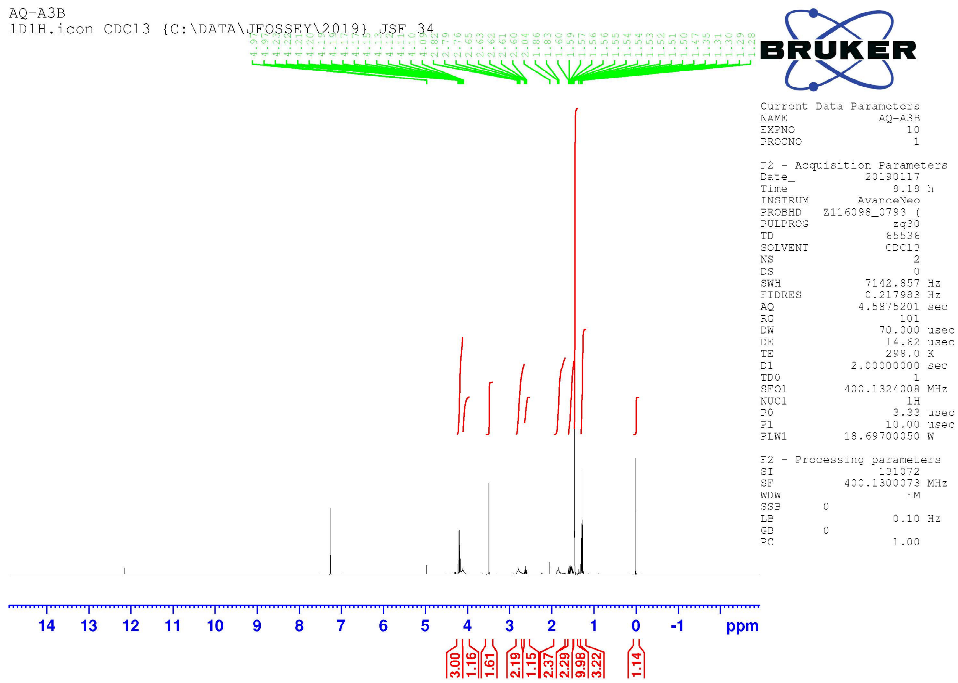

Supplementary Figure 58. Proton NMR spectrum of **9**.

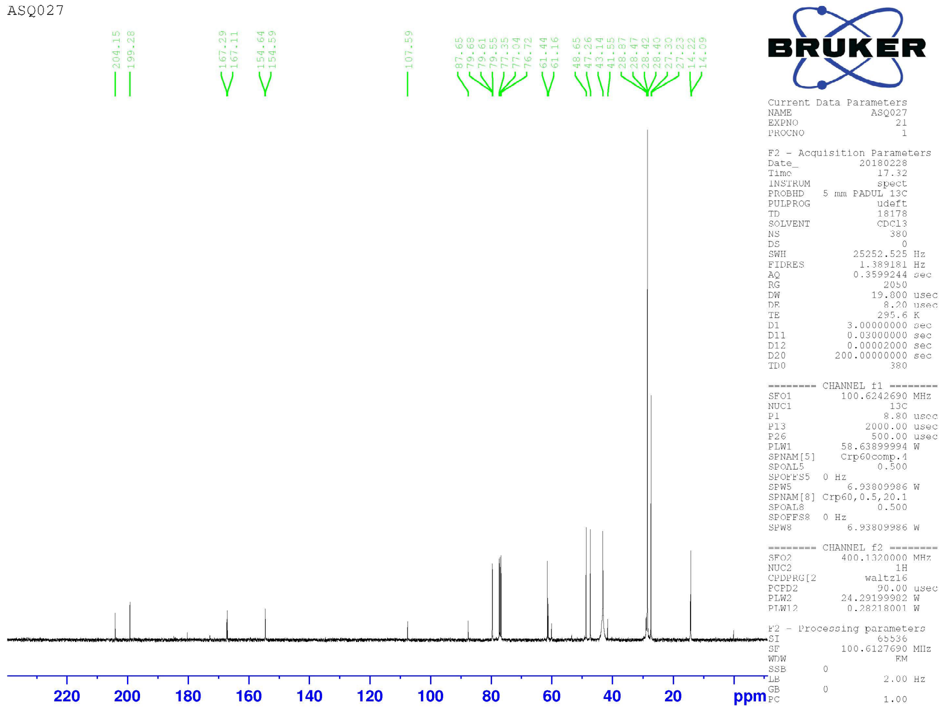

Supplementary Figure 59. ^{13}C NMR spectrum of **9**.

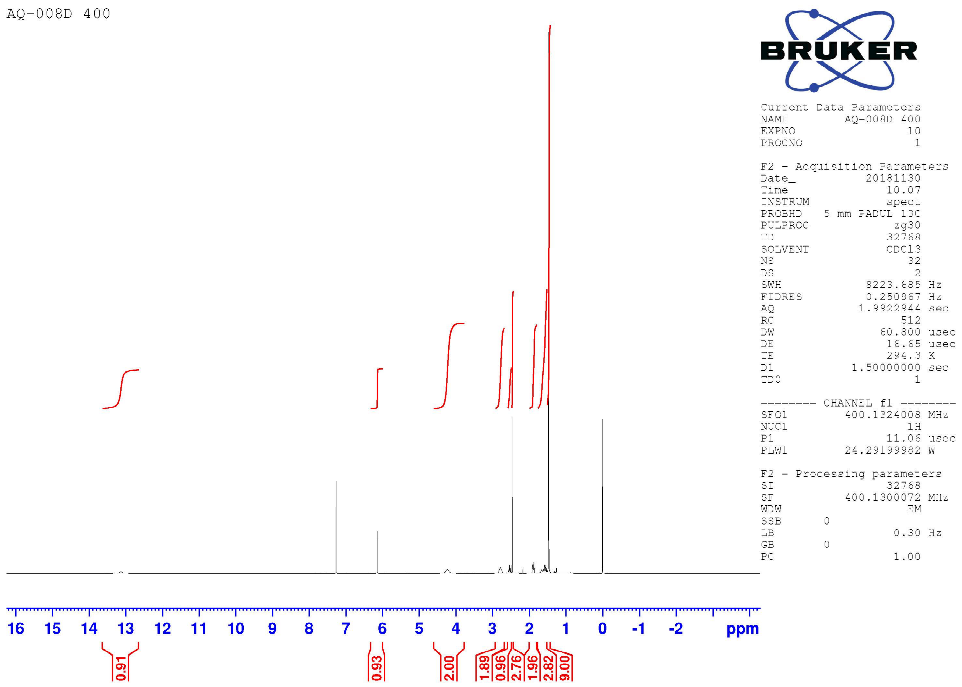

Supplementary Figure 60. *Proton NMR spectrum of* ***10****.*

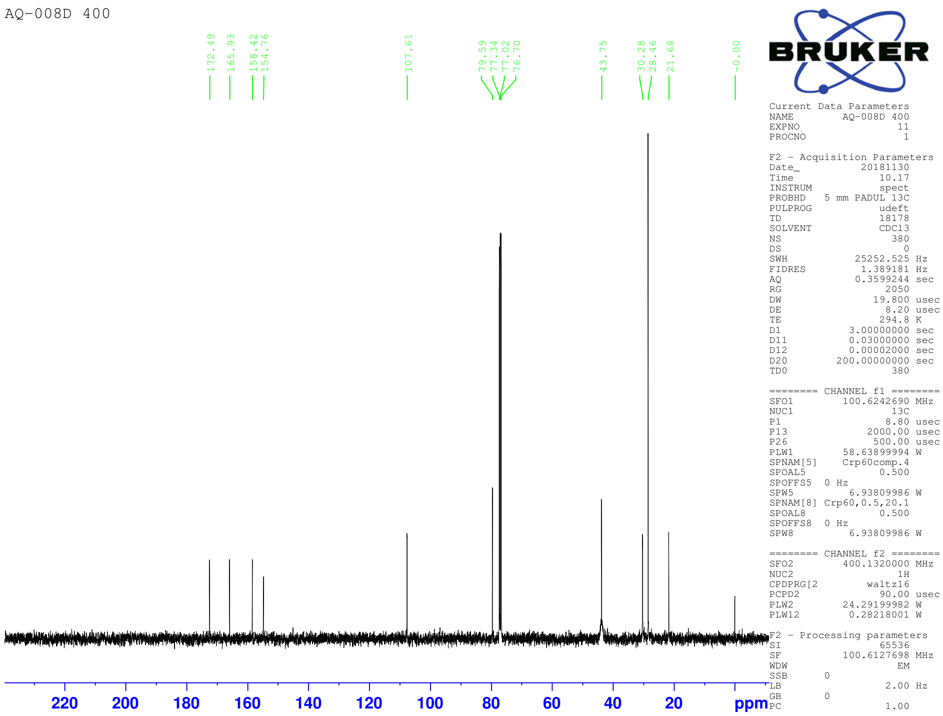

Supplementary Figure 61. *^{13}C NMR spectrum of* ***10****.*

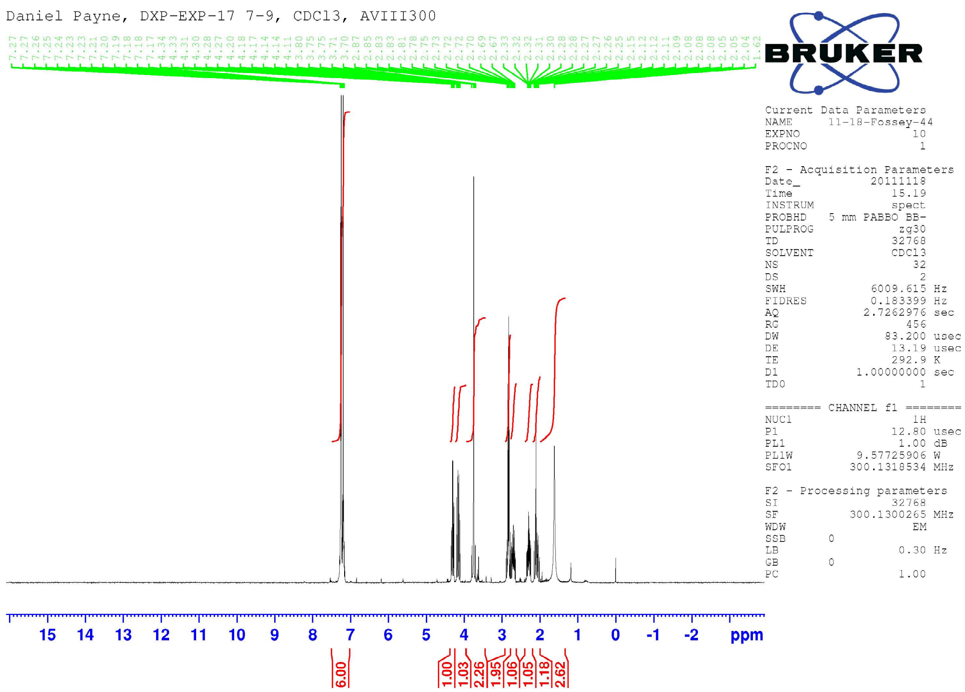

Supplementary Figure 62. *Proton NMR spectrum of S2.*

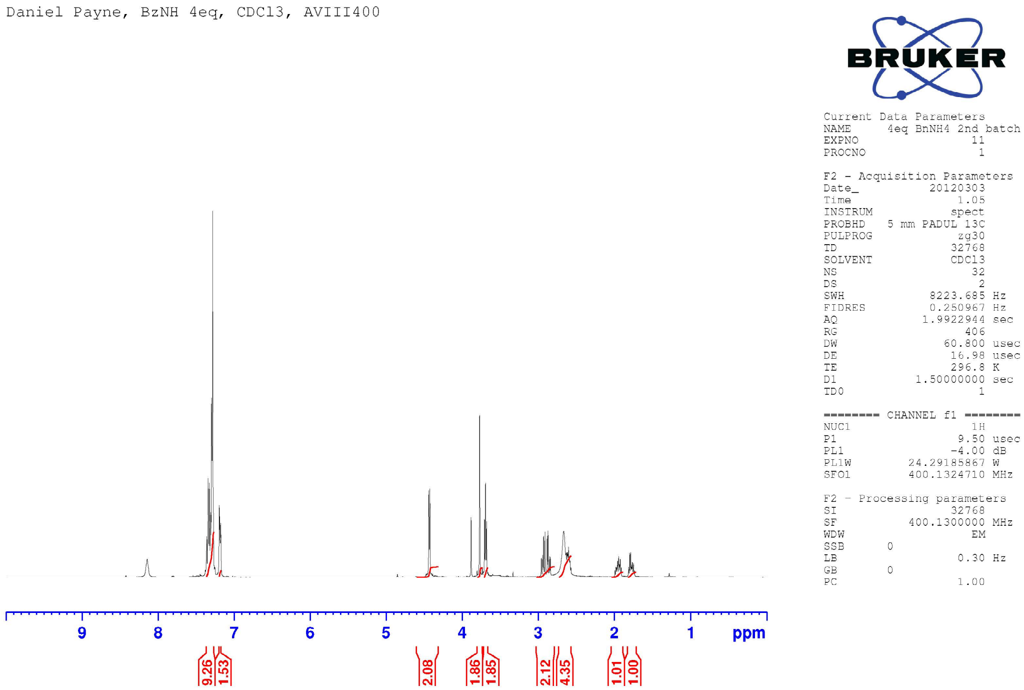

Supplementary Figure 63. *Proton NMR spectrum of S3.*

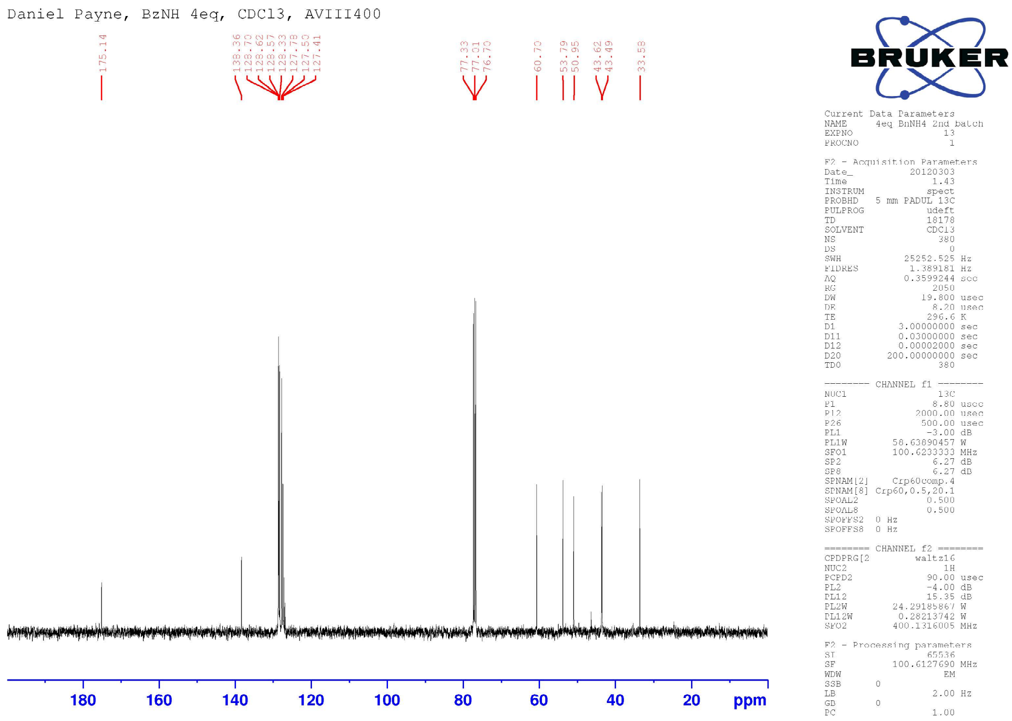

Supplementary Figure 64. *^{13}C NMR spectrum of* ***S3****.*

Proton NMR spectrums obtained from higher throughput synthesis efforts by Sygnature Discovery

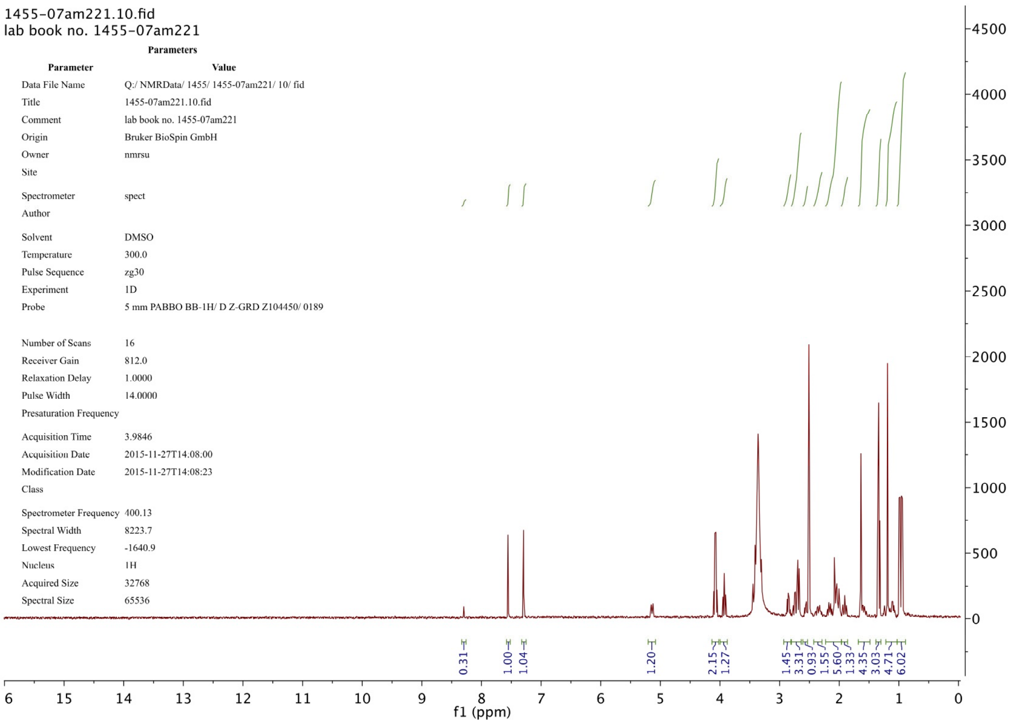

Supplementary Figure 65. *Proton NMR spectrum of* ***2e****.*

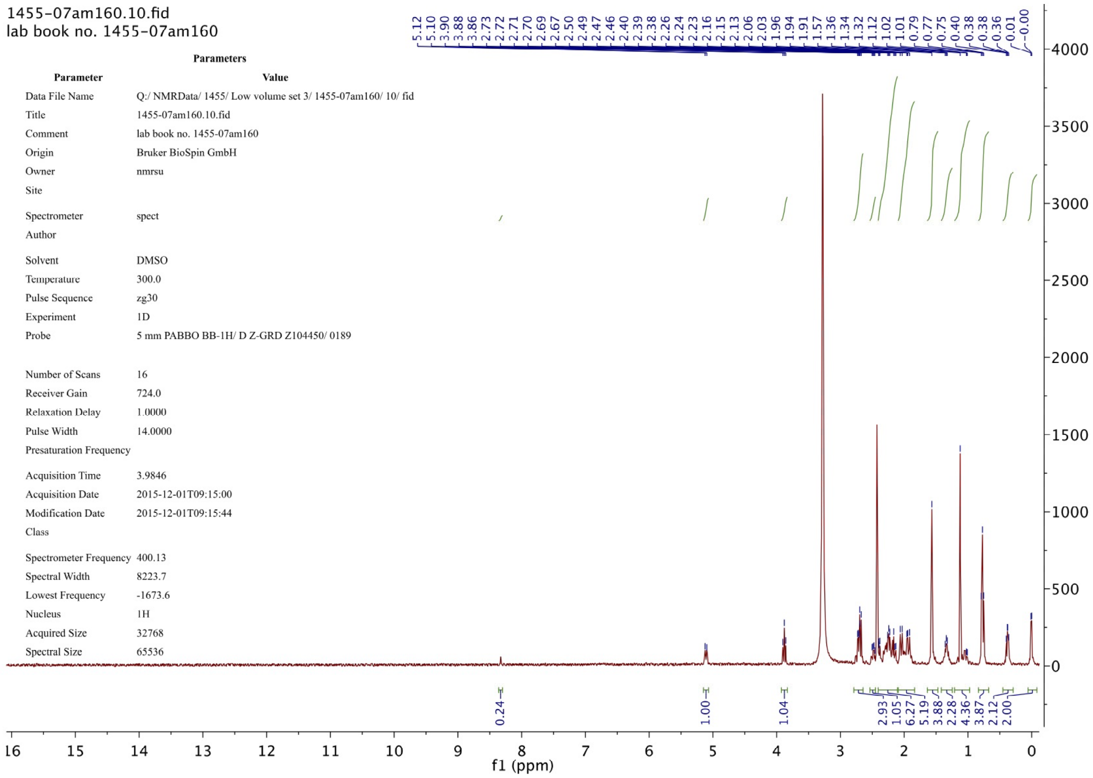

Supplementary Figure 66. *Proton NMR spectrum of* ***2f****.*

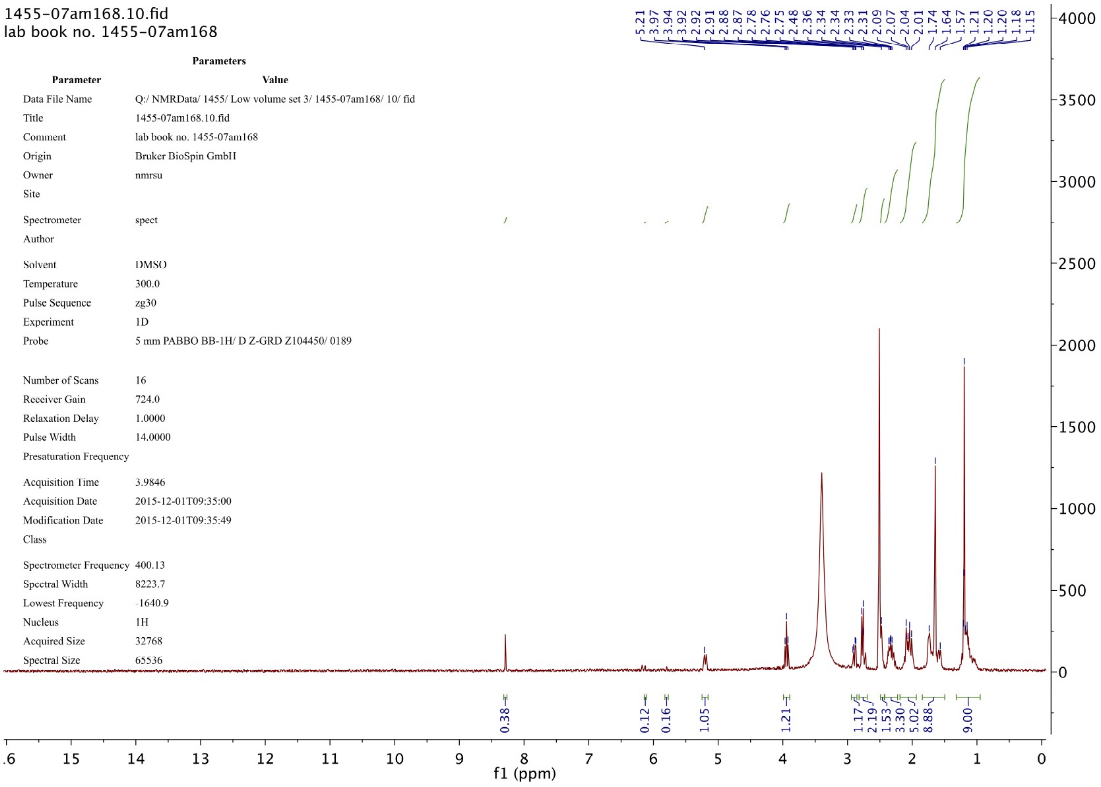

Supplementary Figure 67. *Proton NMR spectrum of* ***2g.***

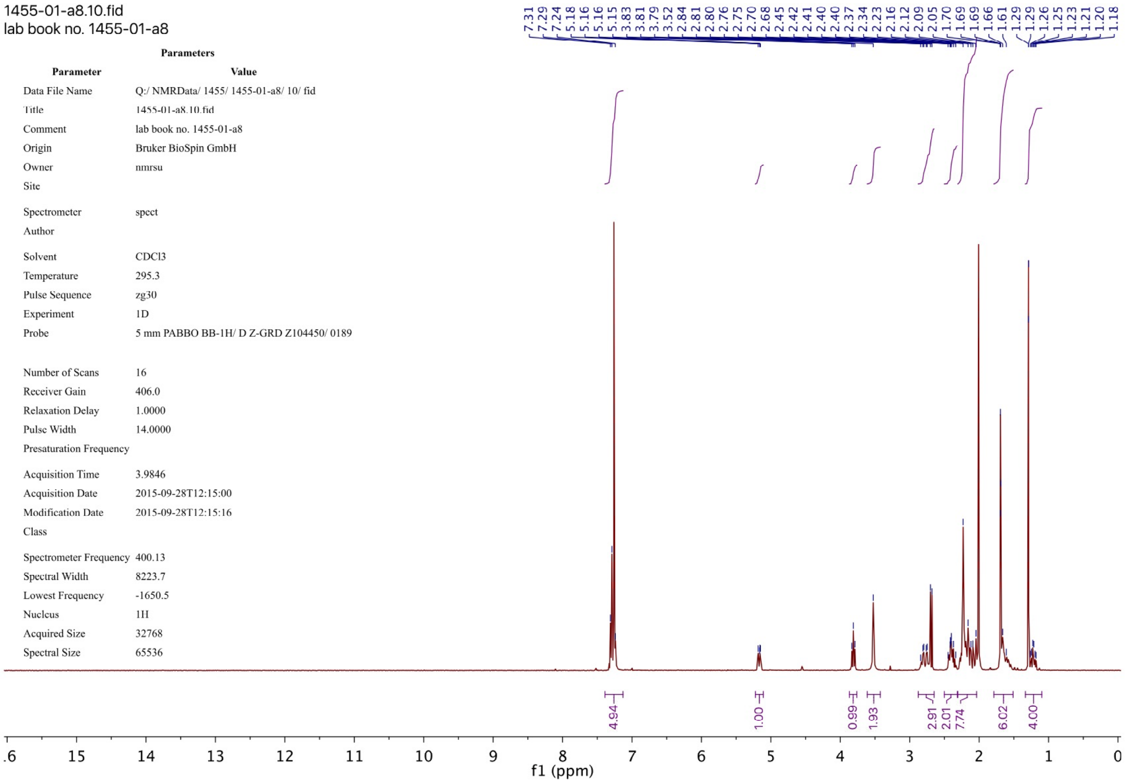

Supplementary Figure 68. *Proton NMR spectrum of* ***2h.***

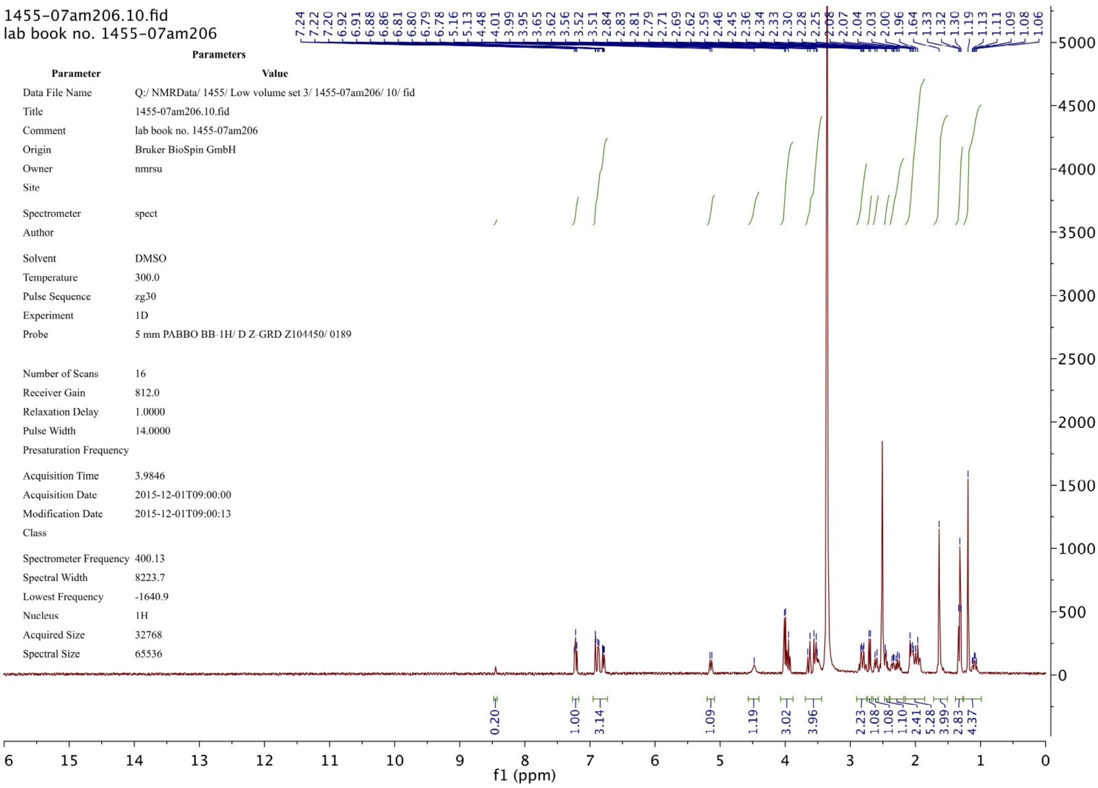

Supplementary Figure 69. *Proton NMR spectrum of* ***2i.***

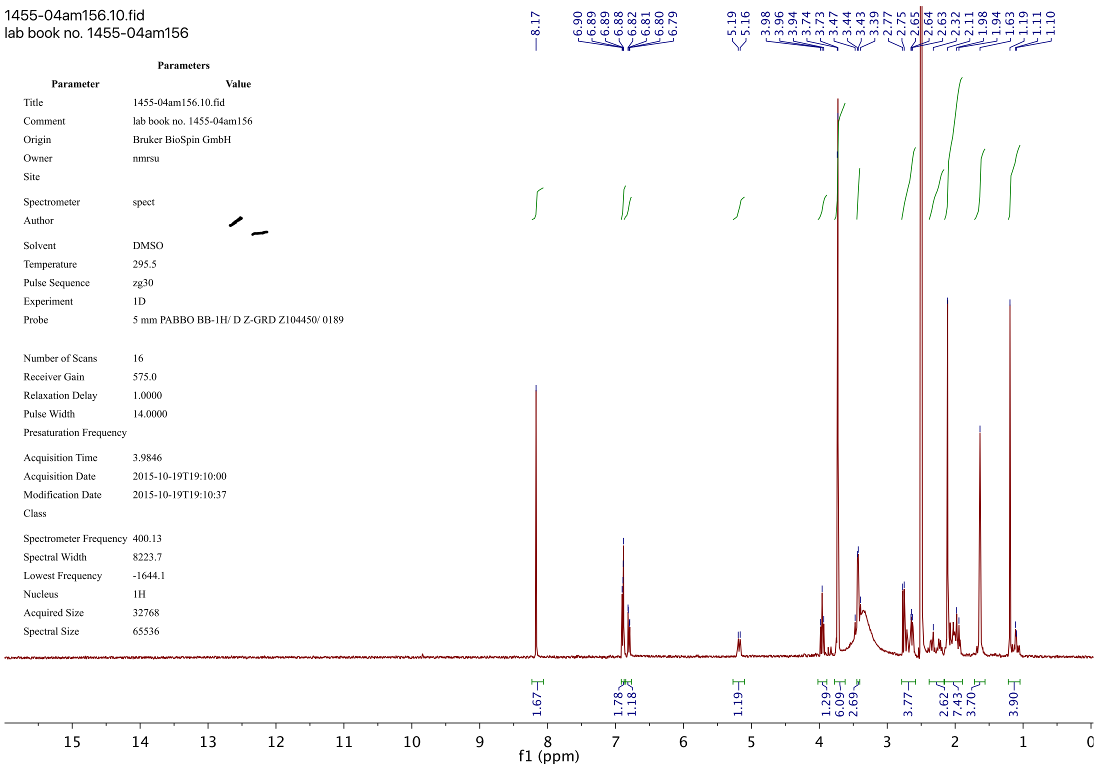

Supplementary Figure 70. *Proton NMR spectrum of* ***2j****.*

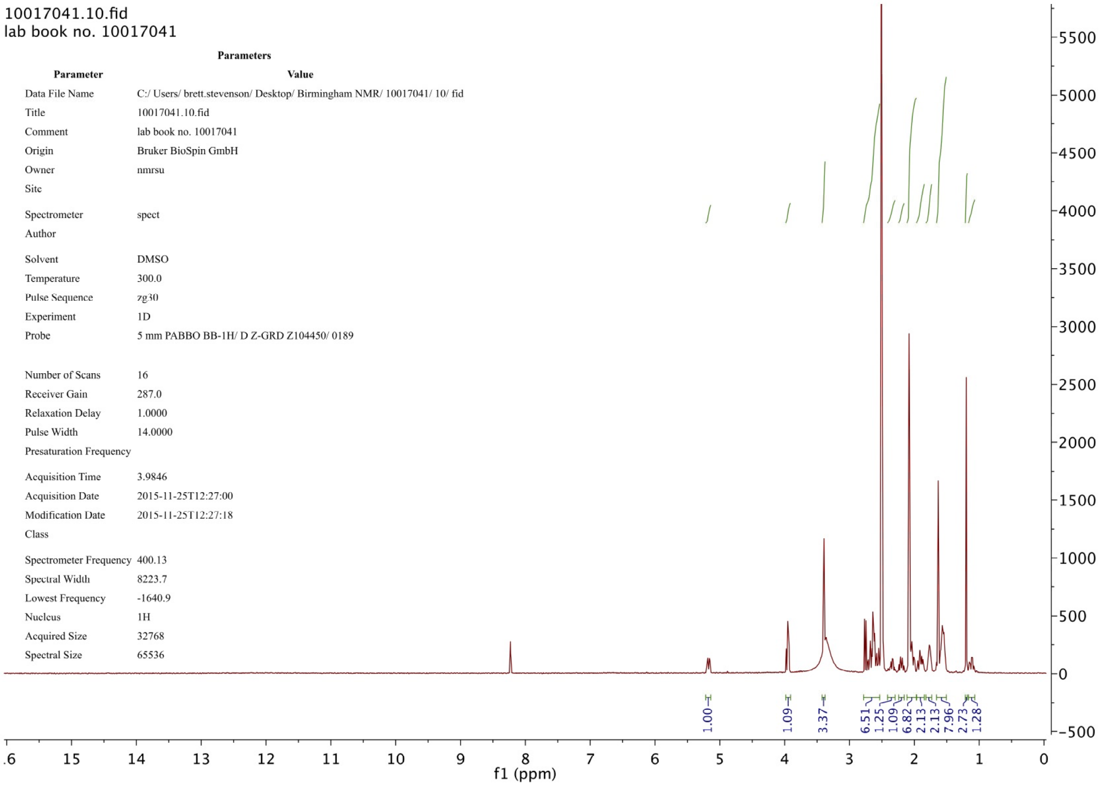

Supplementary Figure 71. *Proton NMR spectrum of* ***2k.***

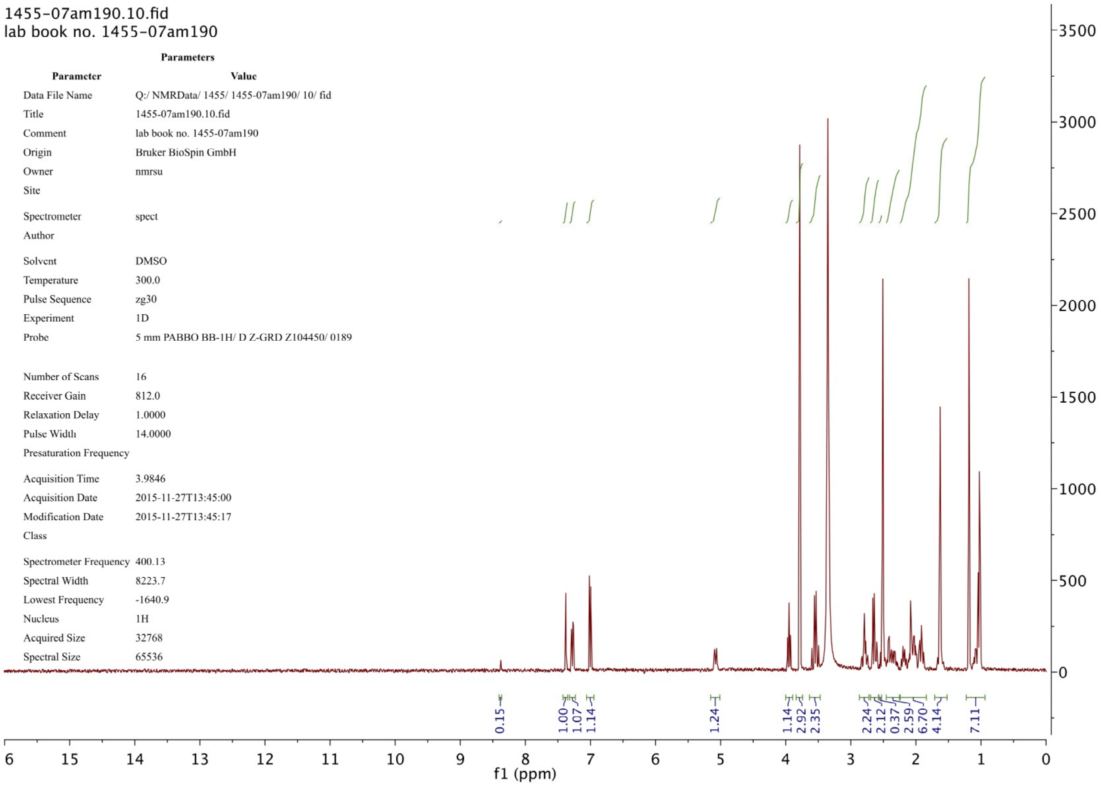

Supplementary Figure 72. *Proton NMR spectrum of* ***2l.***

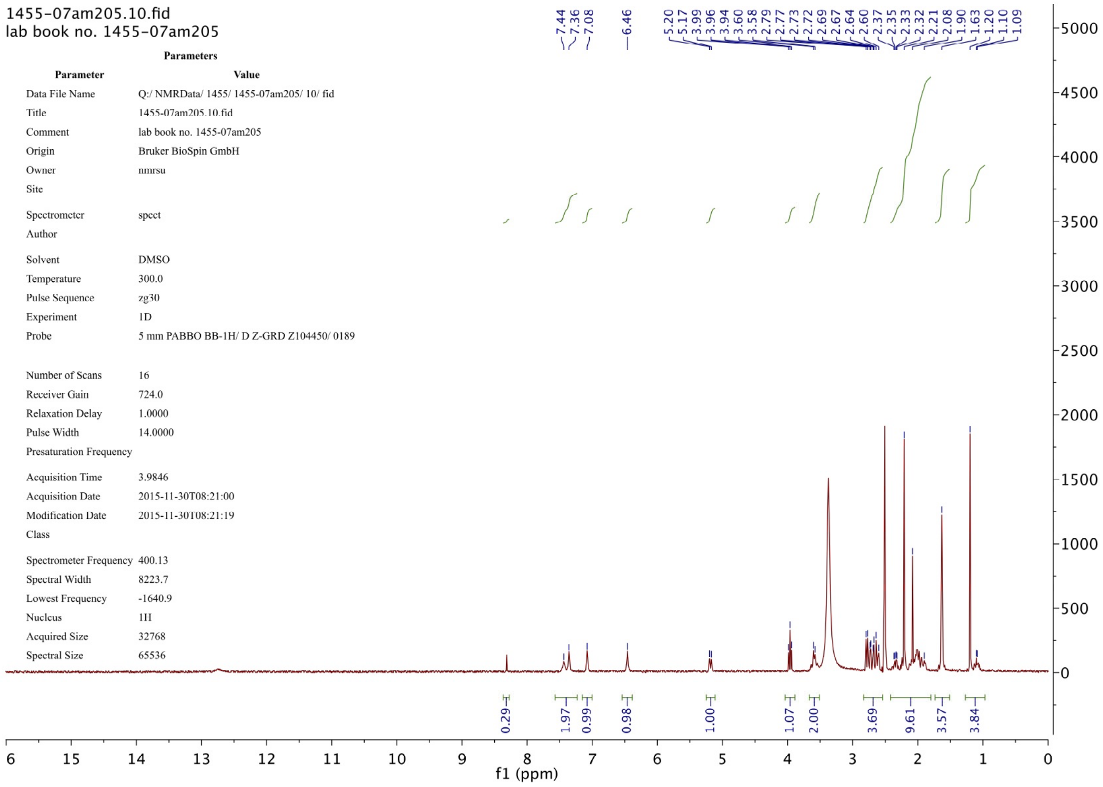

Supplementary Figure 73. *Proton NMR spectrum of* ***2m.***

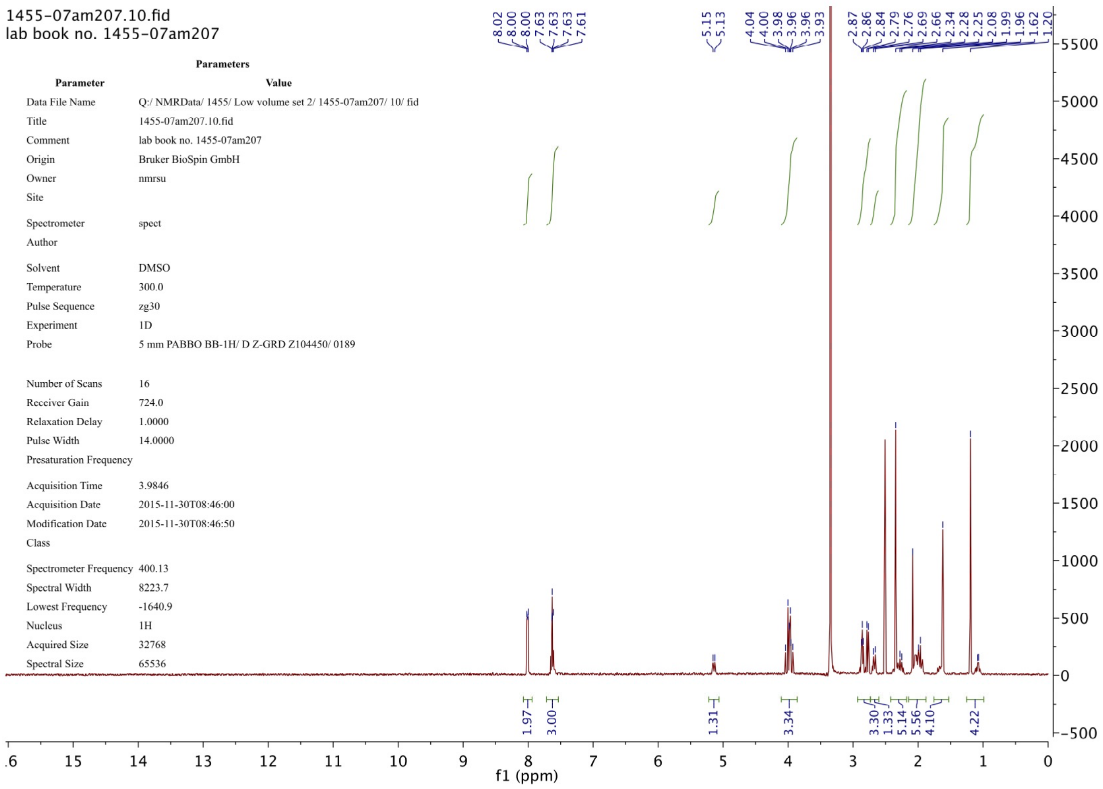

Supplementary Figure 74. *Proton NMR spectrum of* ***2n.***

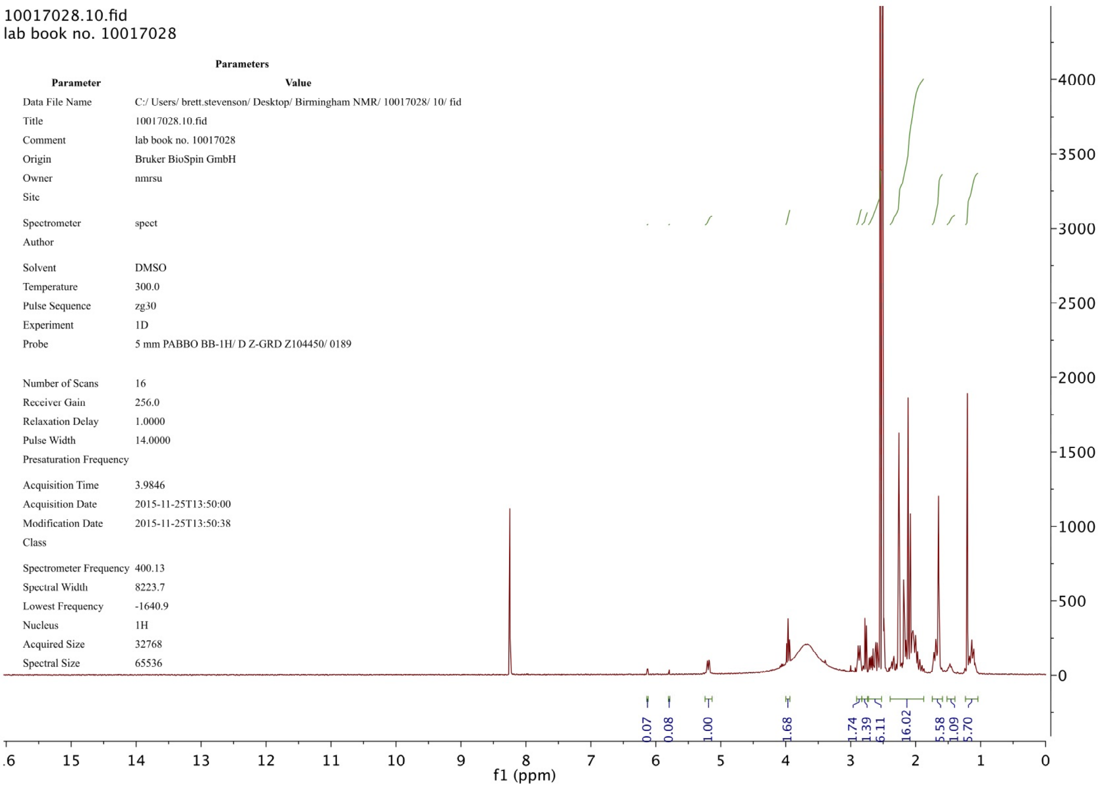

Supplementary Figure 75. *Proton NMR spectrum of **2o**.*

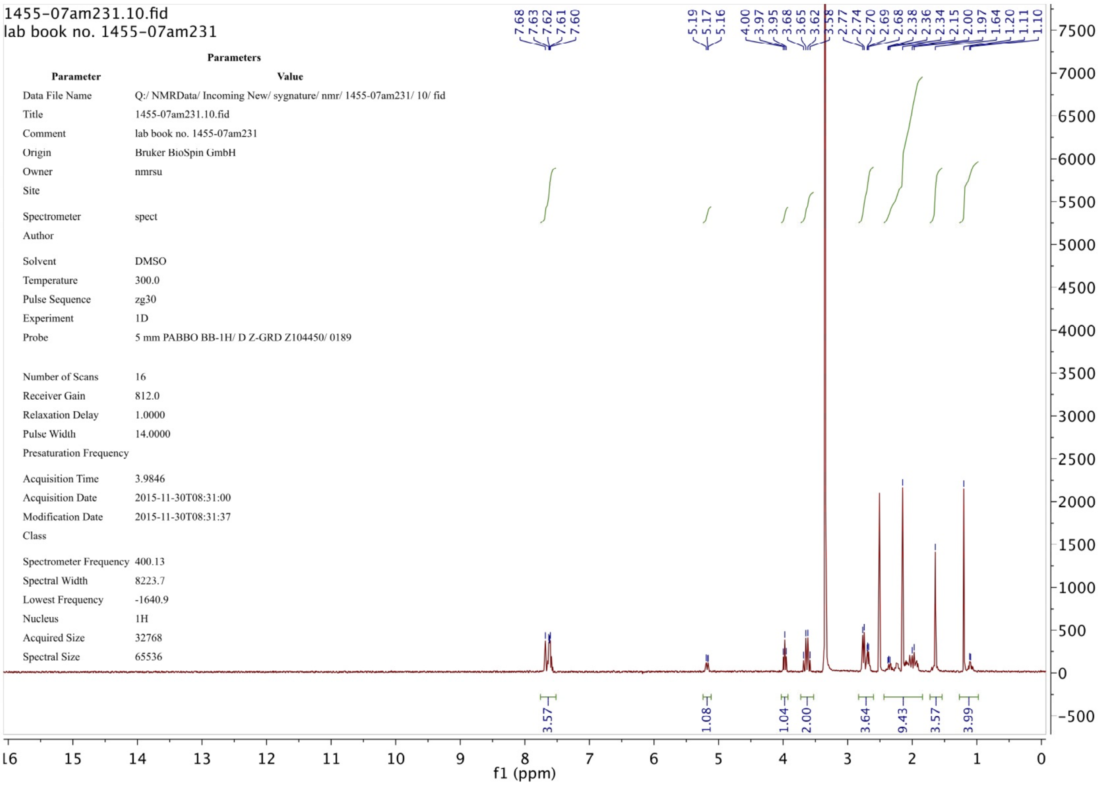

Supplementary Figure 76. *Proton NMR spectrum of **2p**.*

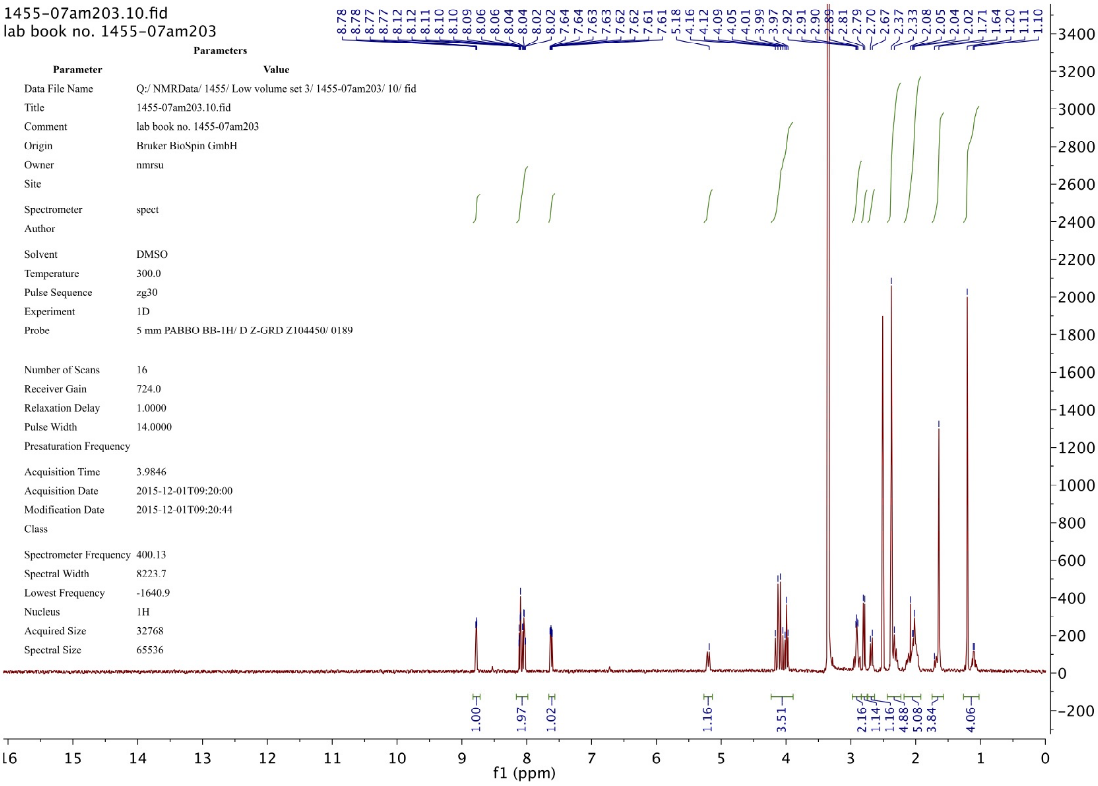

Supplementary Figure 77. *Proton NMR spectrum of* ***2q.***

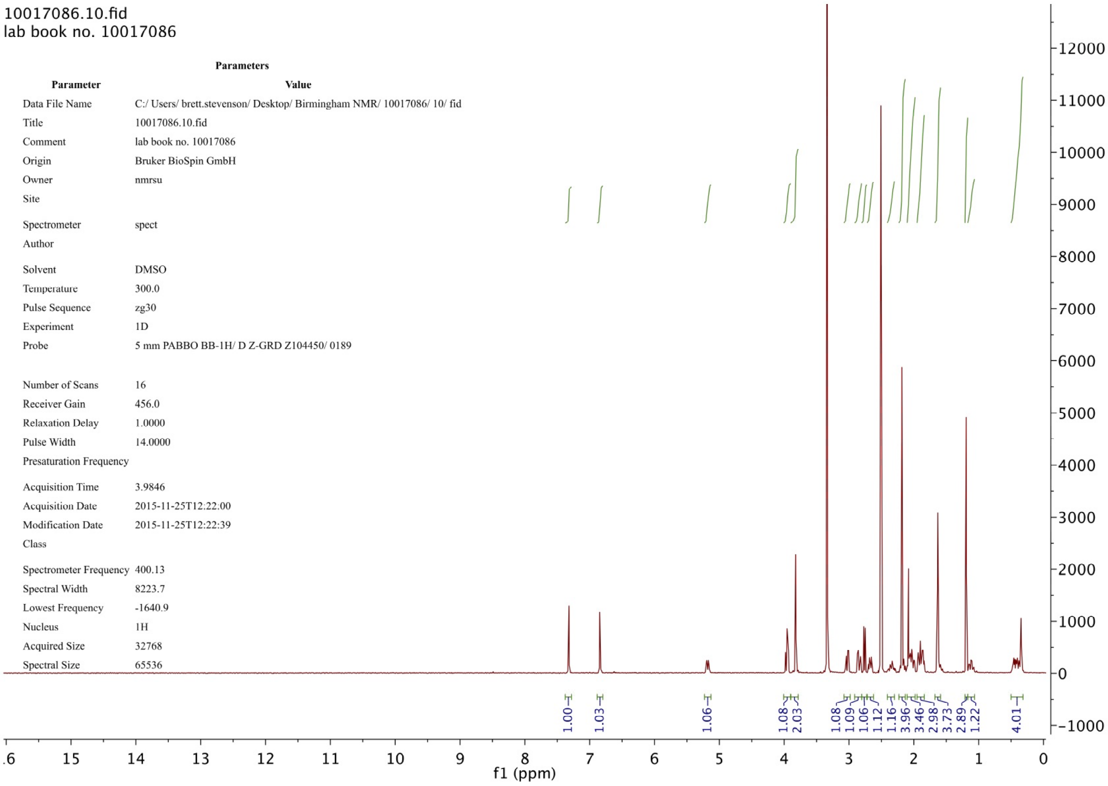

Supplementary Figure 78. *Proton NMR spectrum of* ***2r.***

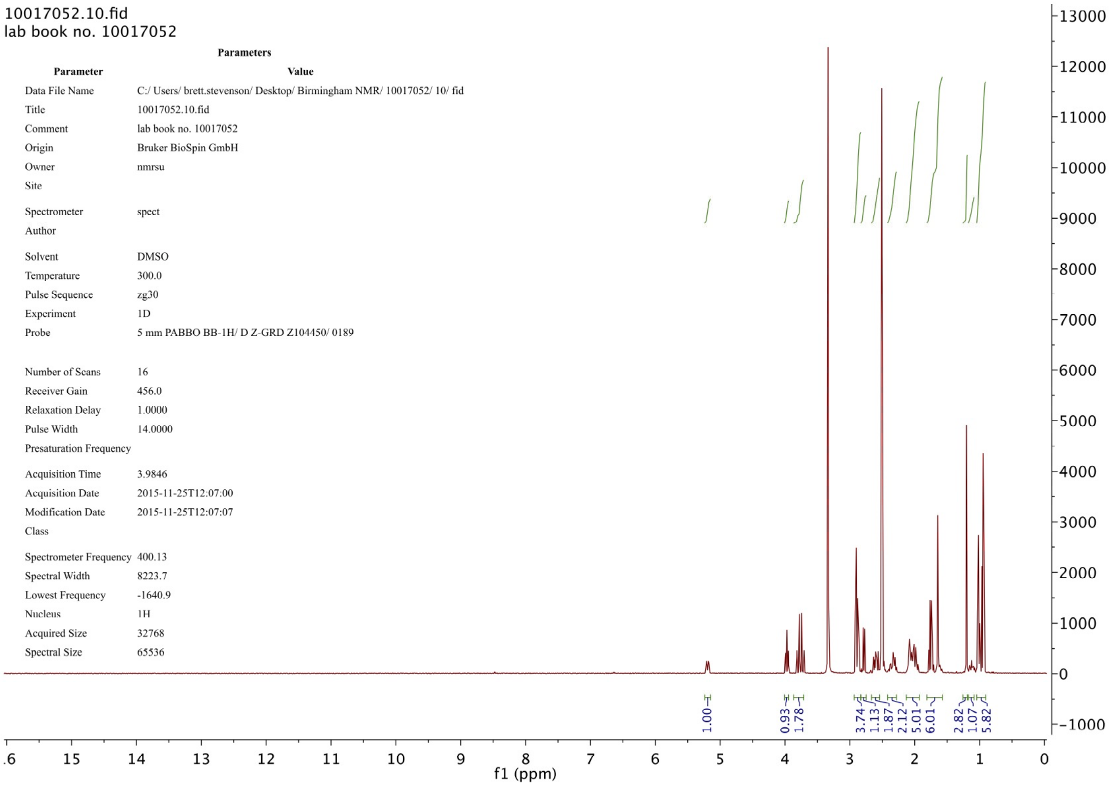

Supplementary Figure 79. Proton NMR spectrum of 2s.

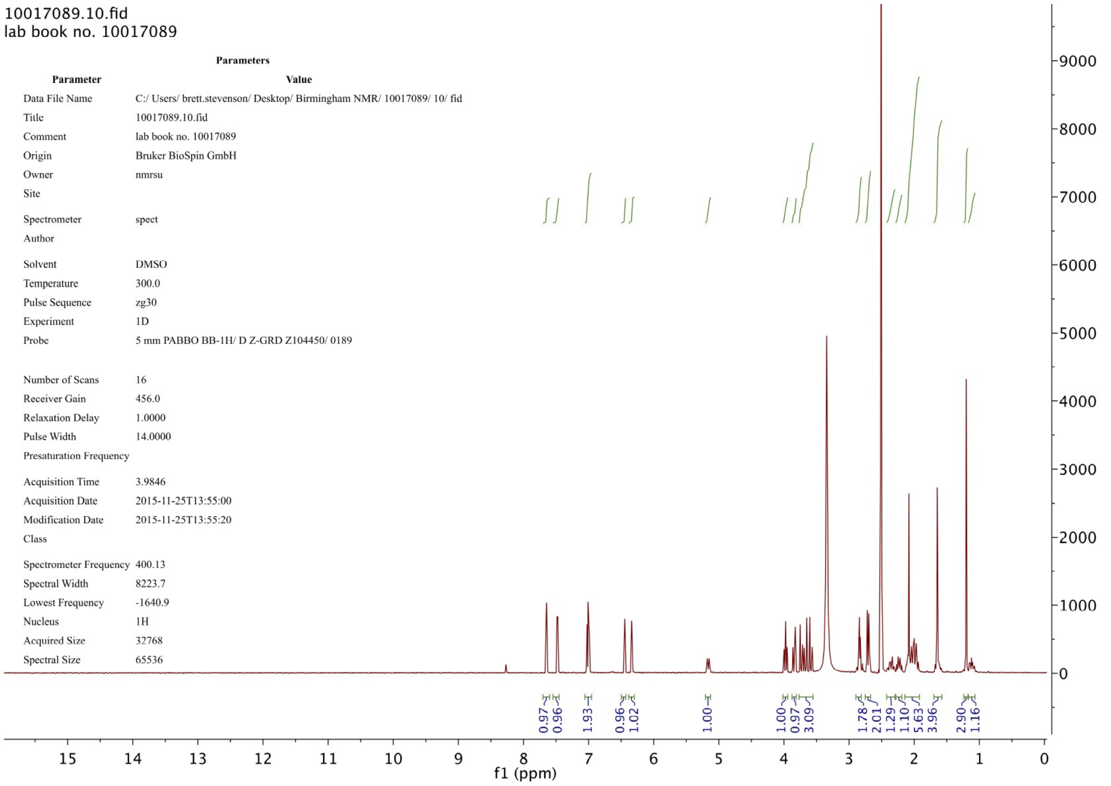

Supplementary Figure 80. *Proton NMR spectrum of* ***2t.***

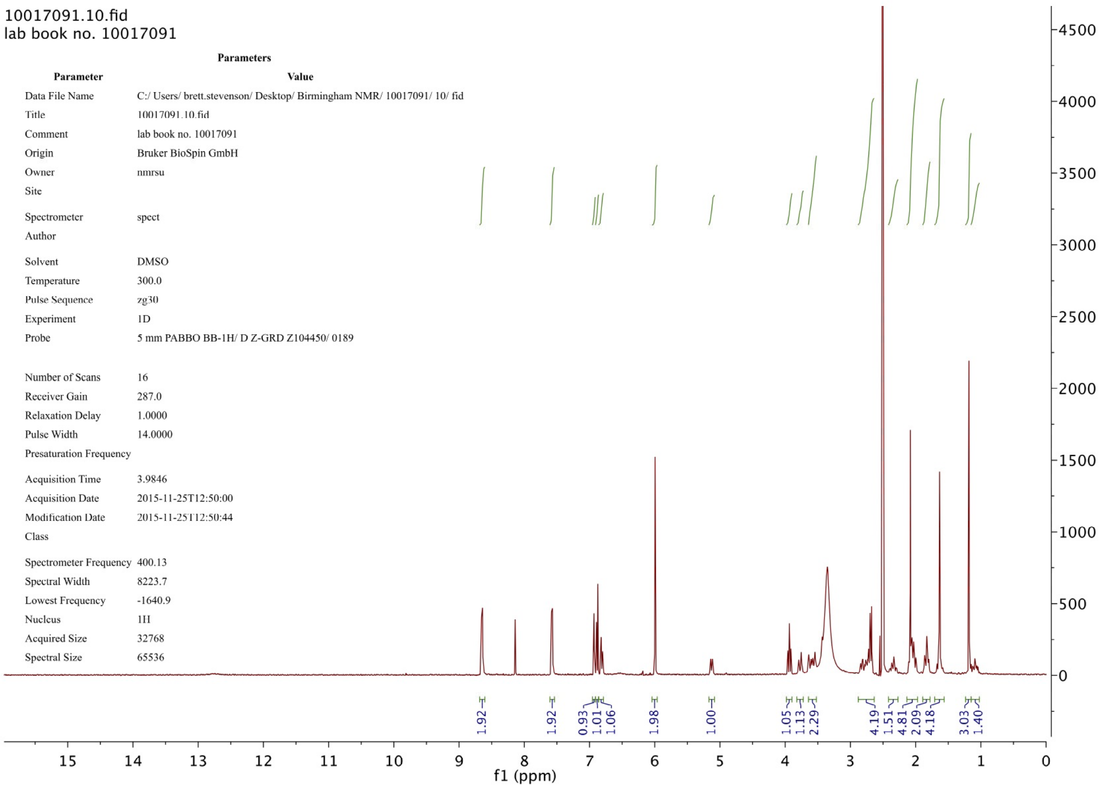

Supplementary Figure 81. *Proton NMR spectrum of* ***2u.***

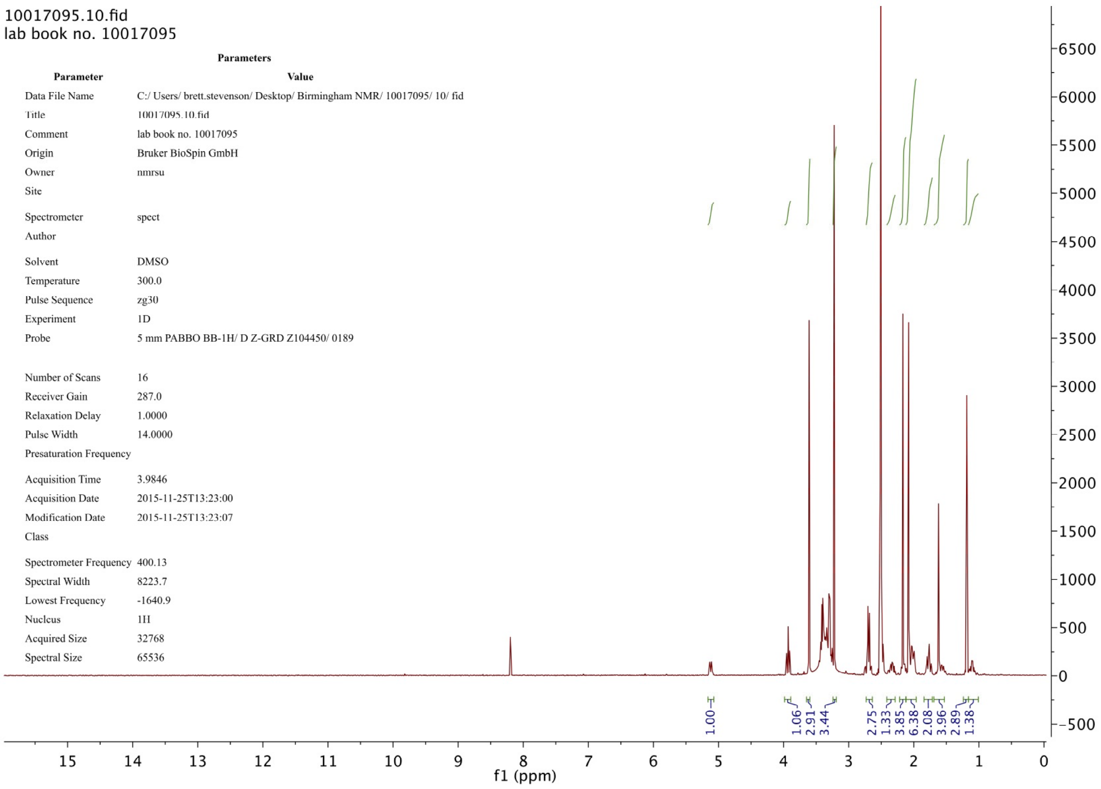

Supplementary Figure 82. *Proton NMR spectrum of* ***2v.***

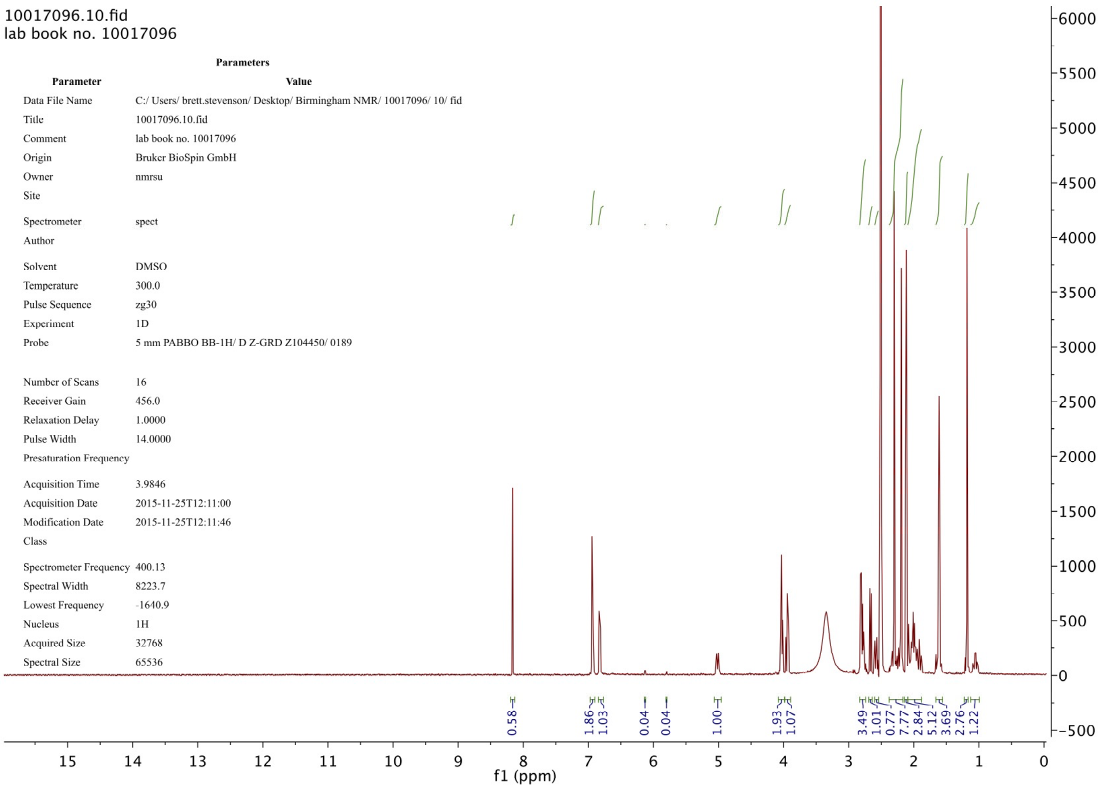

Supplementary Figure 83. *Proton NMR spectrum of* ***2w.***

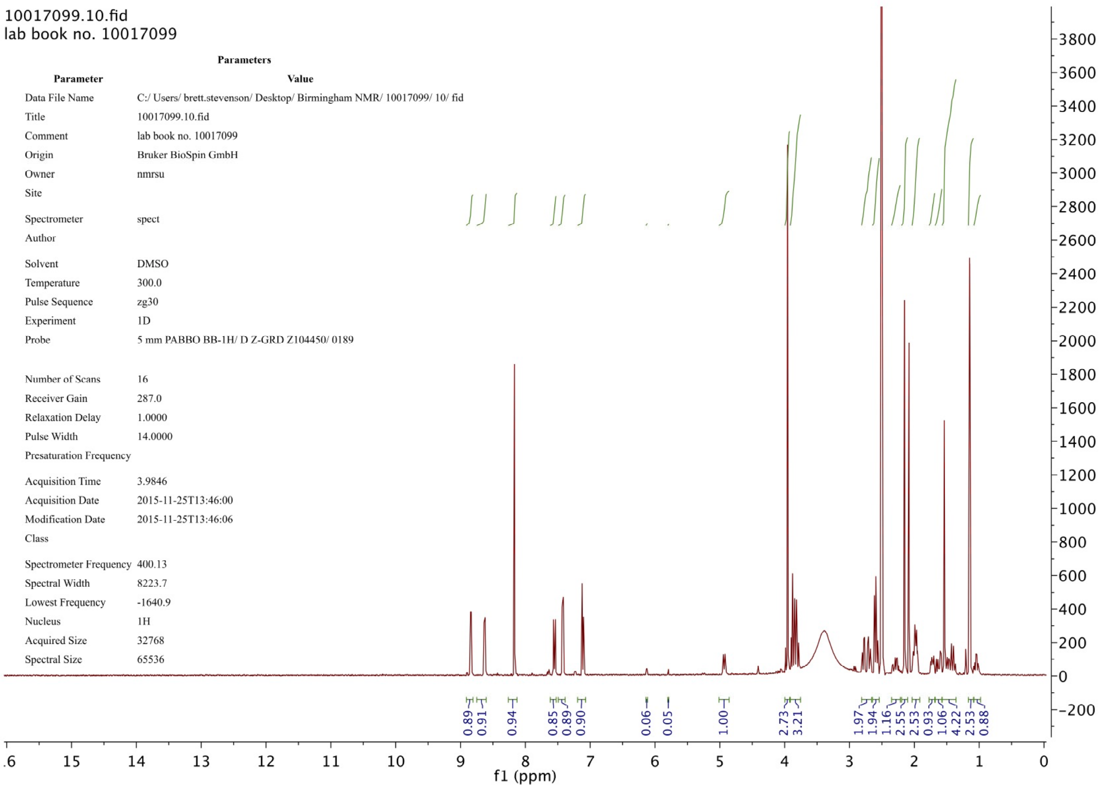

Supplementary Figure 84. *Proton NMR spectrum of* ***2x.***

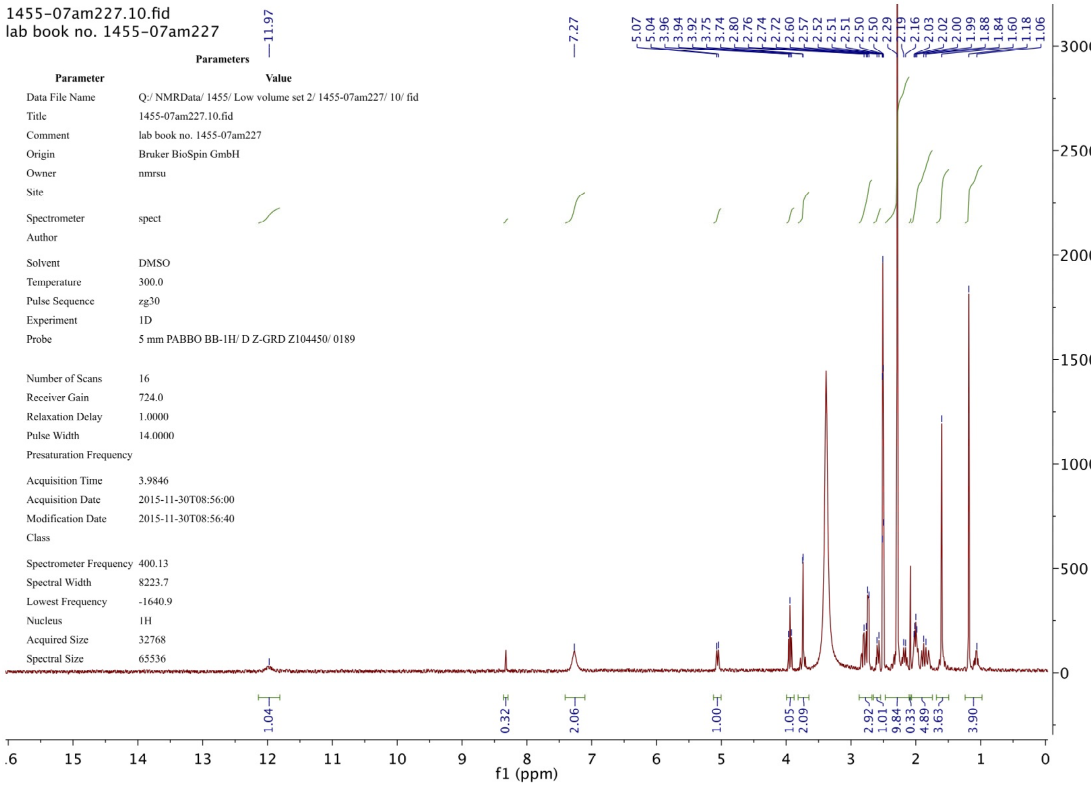

Supplementary Figure 85. *Proton NMR spectrum of* ***2y.***

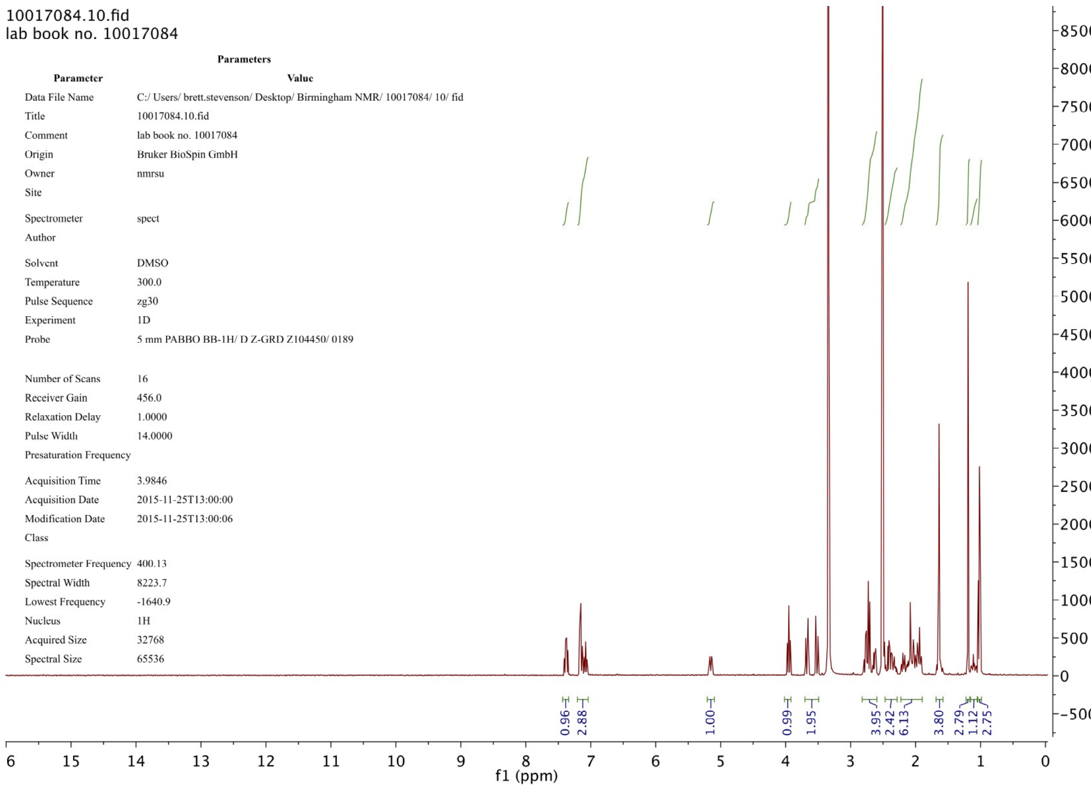

Supplementary Figure 86. *Proton NMR spectrum of* ***2z.***

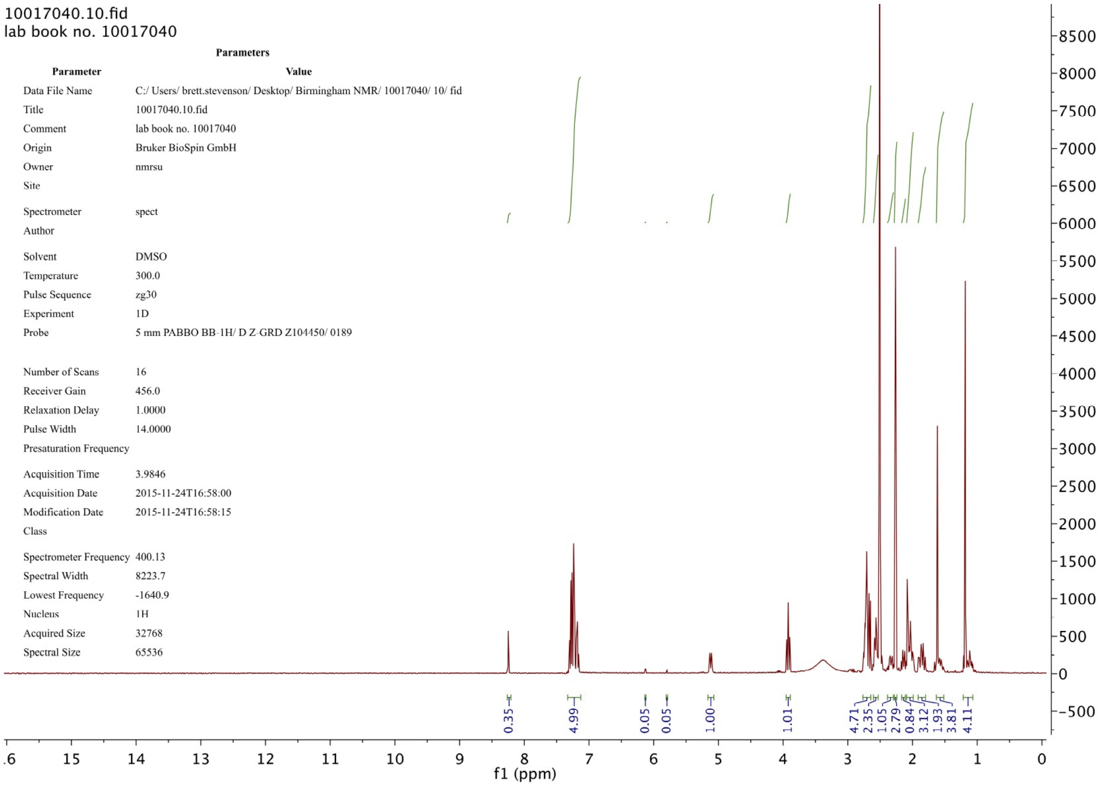

Supplementary Figure 87. *Proton NMR spectrum of* ***2aa****.*

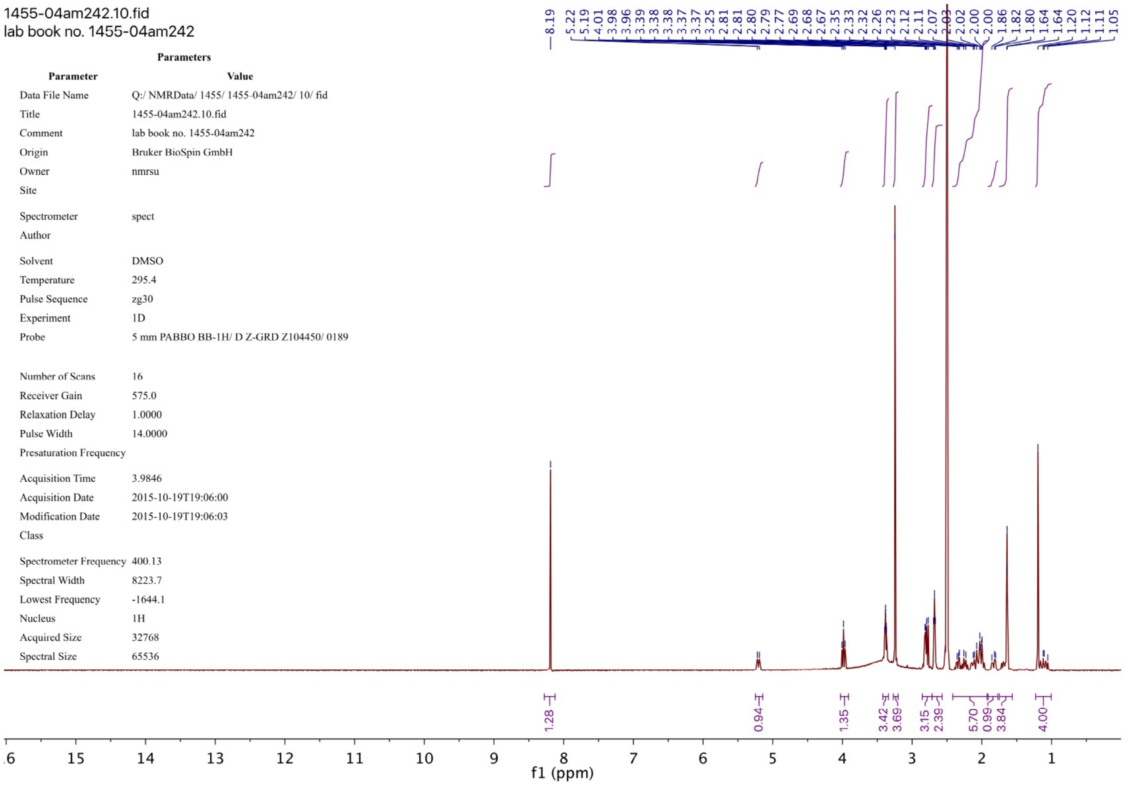

Supplementary Figure 88. *Proton NMR spectrum of* ***4b****.*

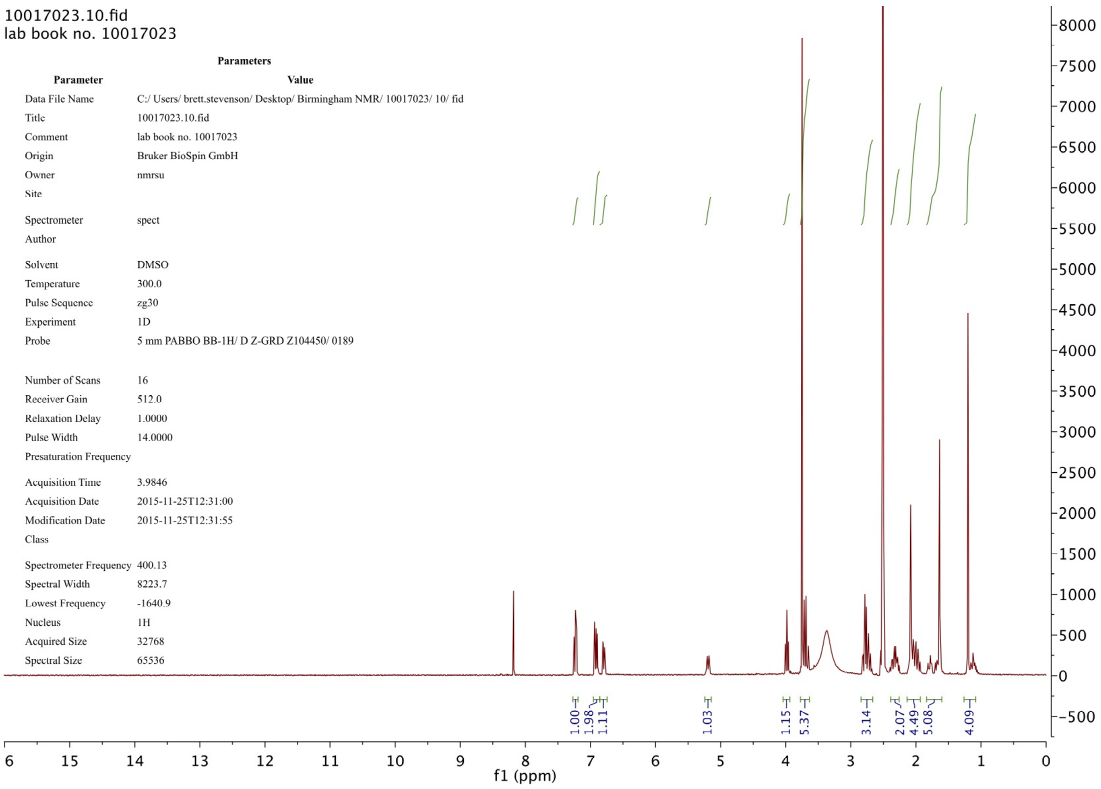

Supplementary Figure 89. *Proton NMR spectrum of* ***4e.***

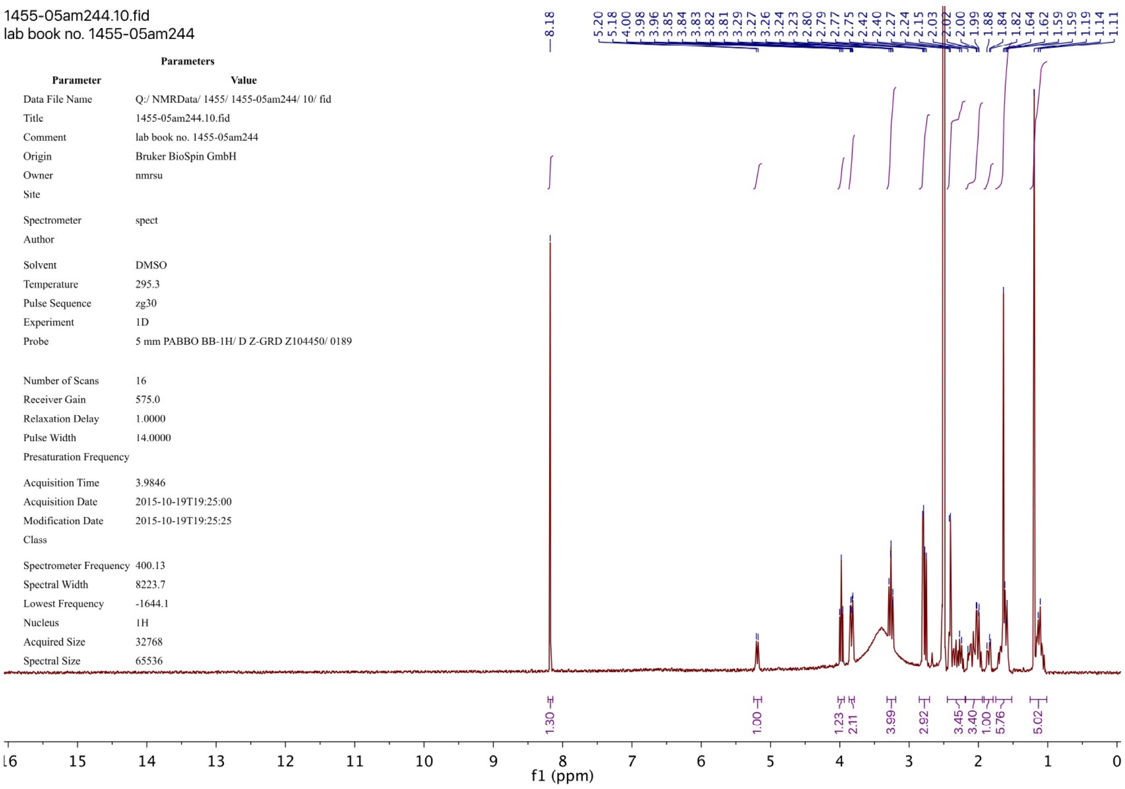

Supplementary Figure 90. *Proton NMR spectrum of* ***4f.***

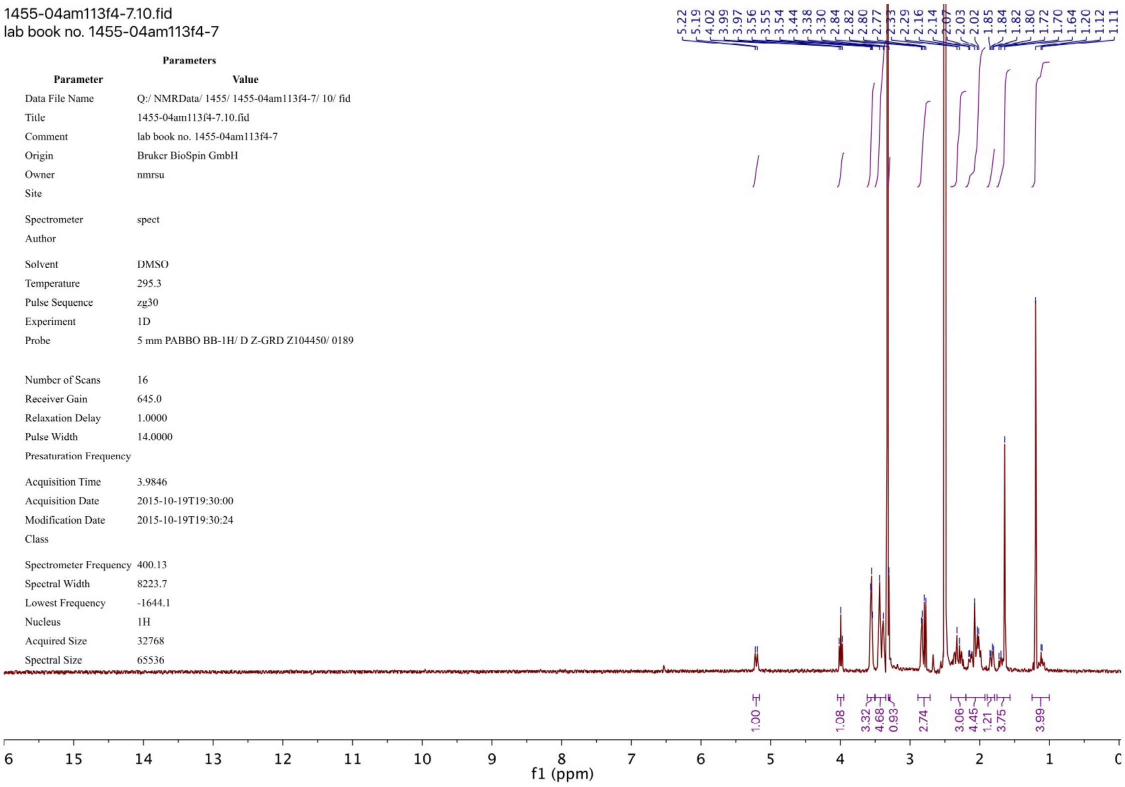

Supplementary Figure 91. *Proton NMR spectrum of* ***4g.***

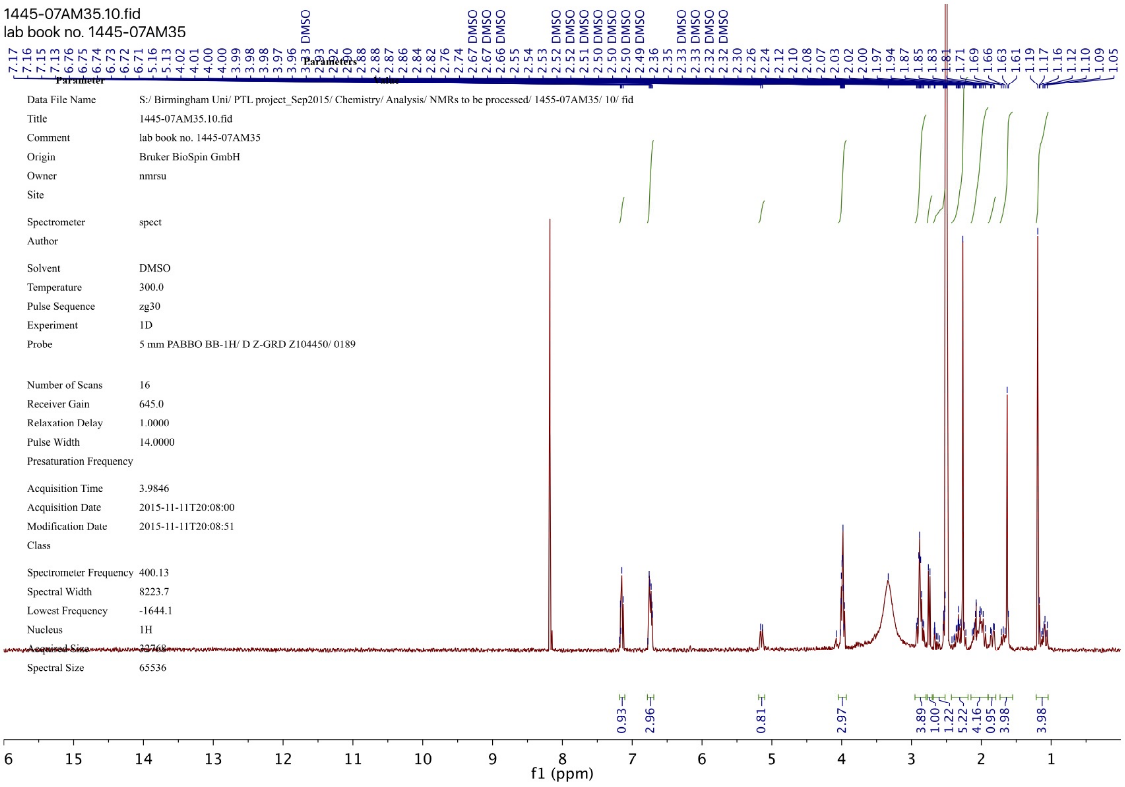

Supplementary Figure 92. *Proton NMR spectrum of* ***4h.***

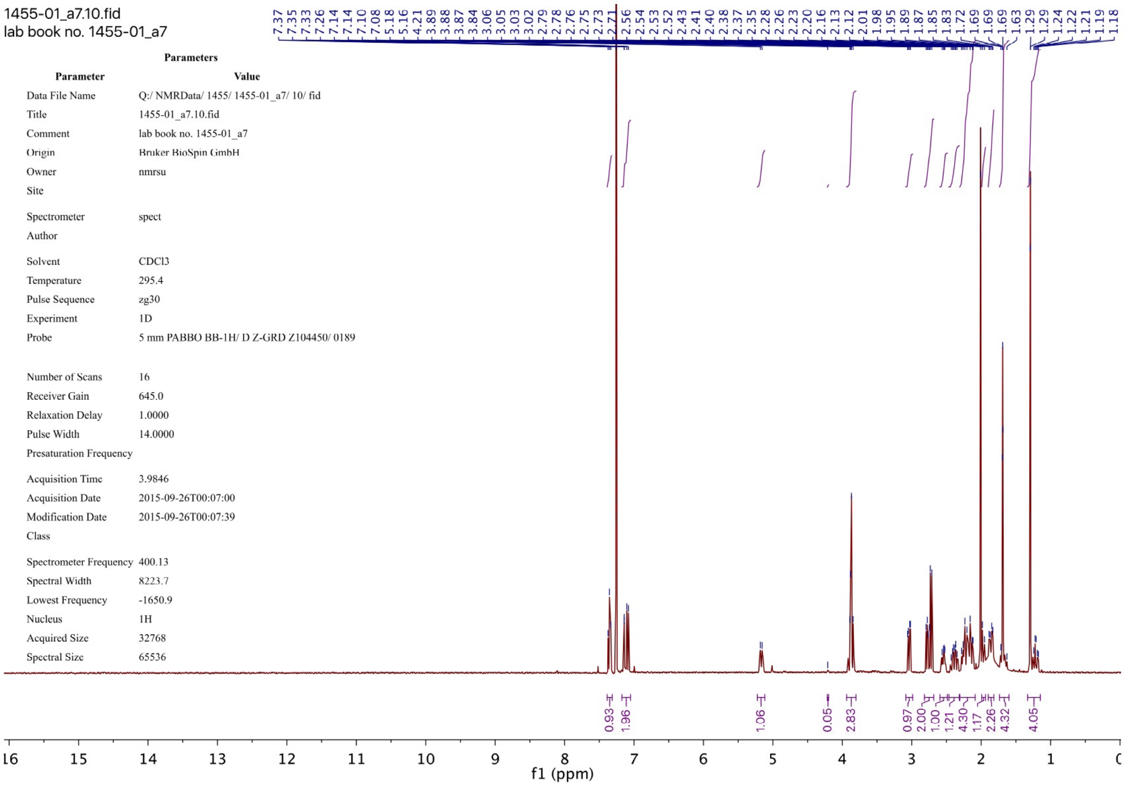

Supplementary Figure 93. *Proton NMR spectrum of* ***4i.***

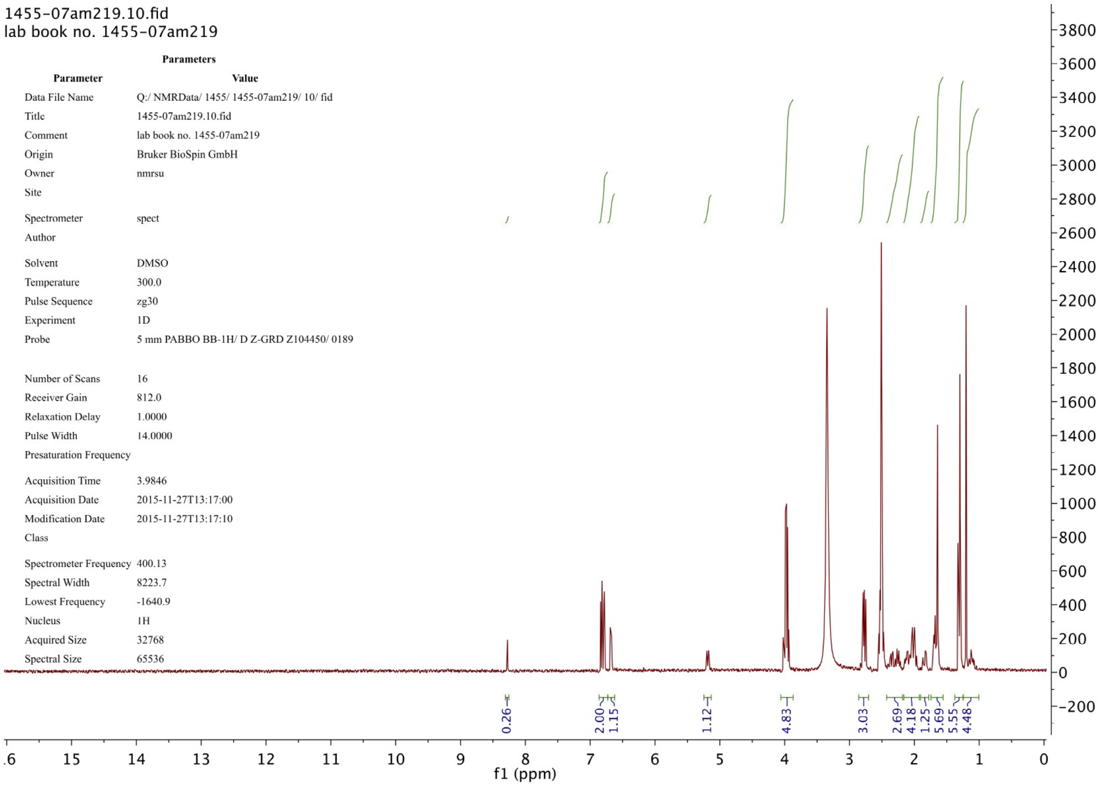

Supplementary Figure 94. *Proton NMR spectrum of* ***4j.***

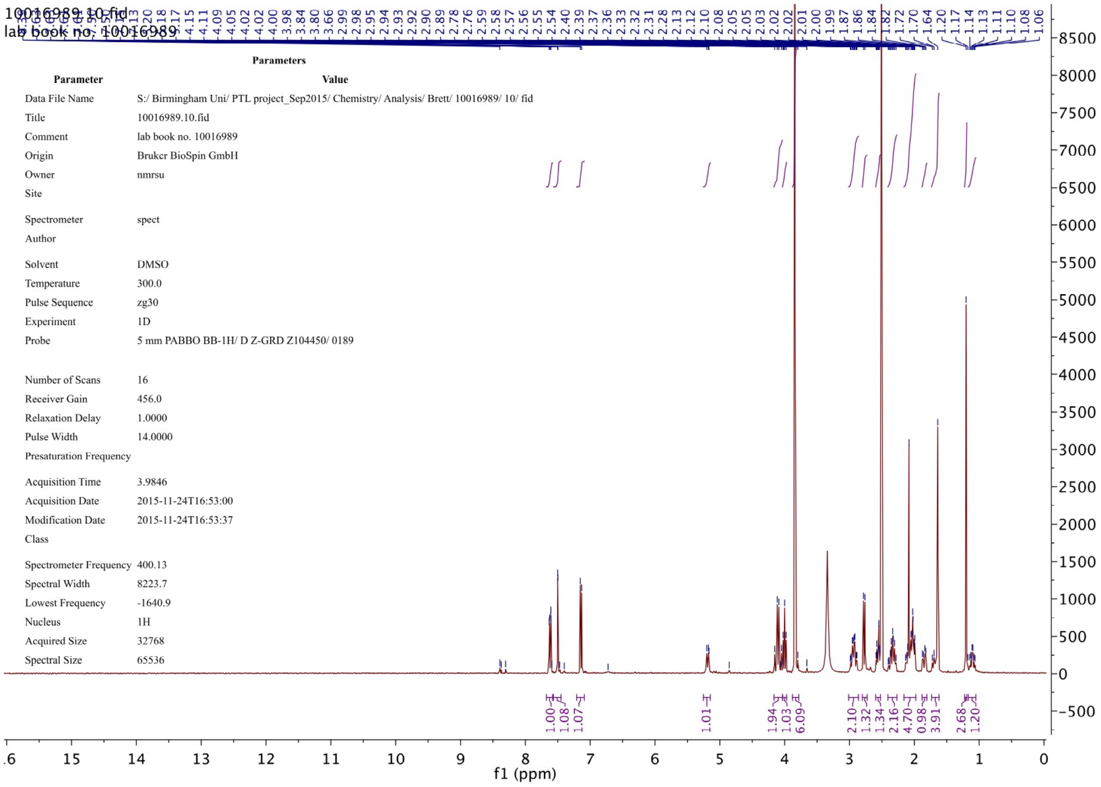

Supplementary Figure 95. *Proton NMR spectrum of* ***4k****.*

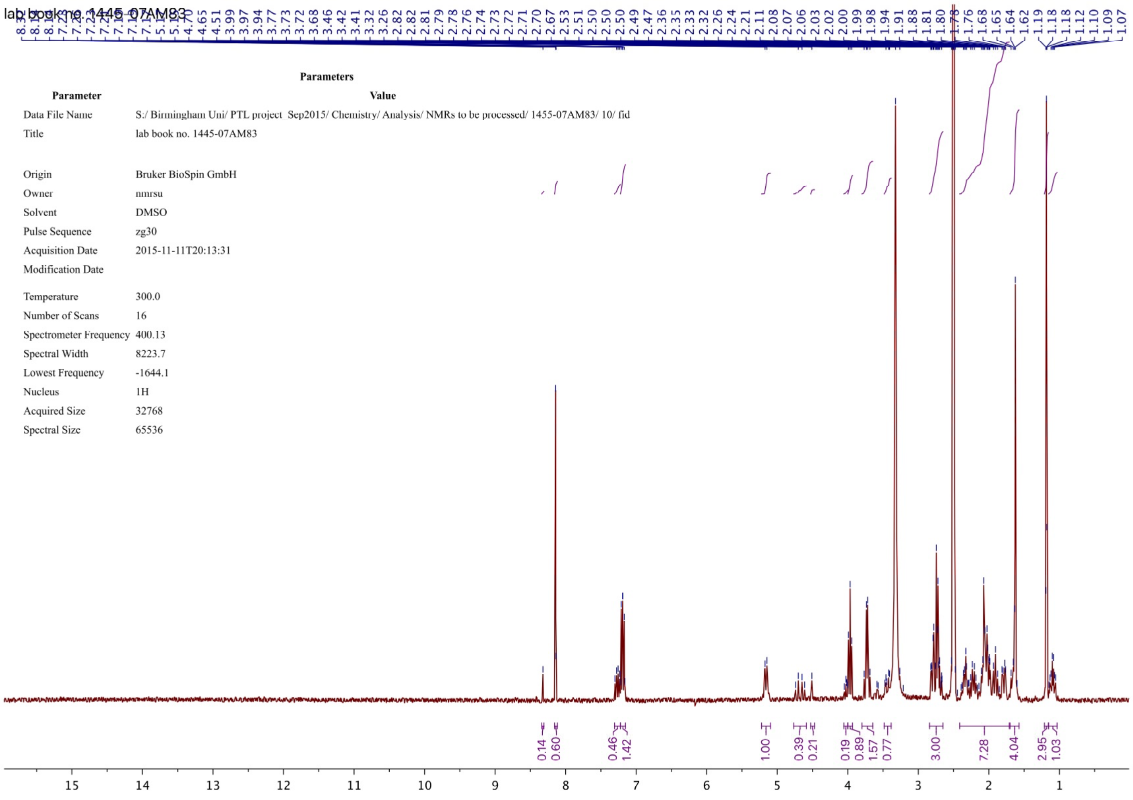

Supplementary Figure 96. *Proton NMR spectrum of* ***4l****.*

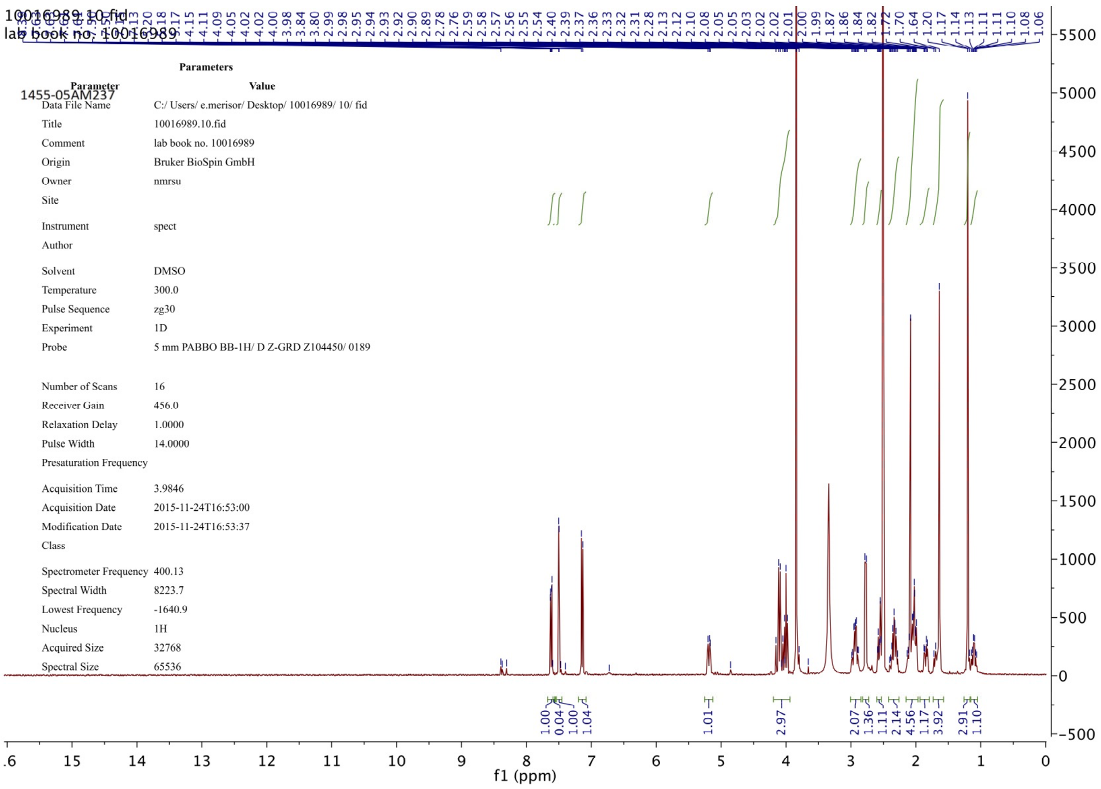

Supplementary Figure 97. *Proton NMR spectrum of* ***4m.***

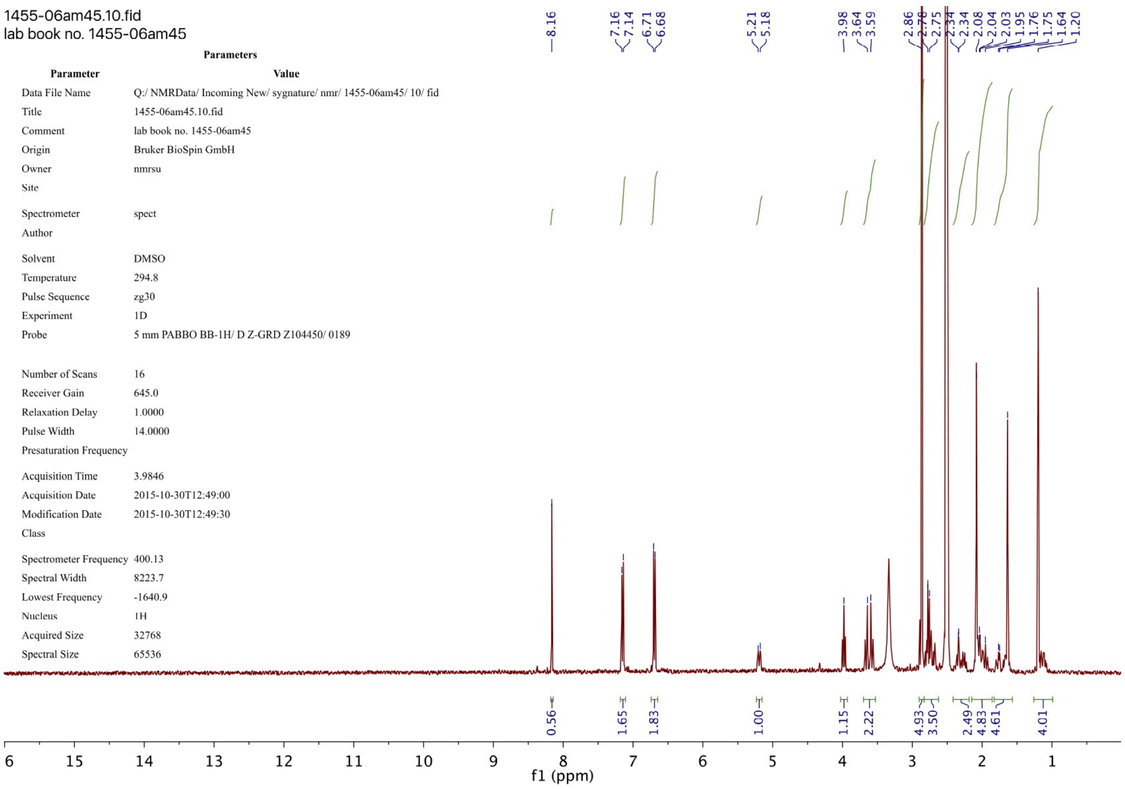

Supplementary Figure 98. *Proton NMR spectrum of* ***4n.***

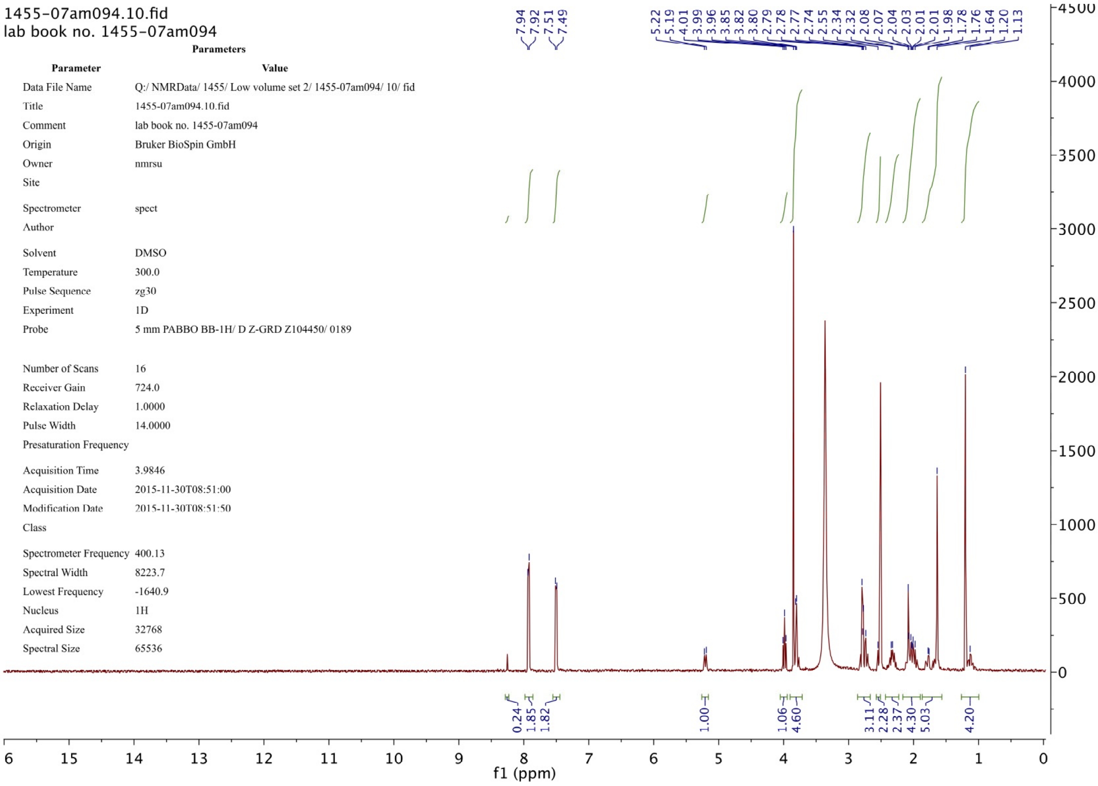

Supplementary Figure 99. *Proton NMR spectrum of* ***4o.***

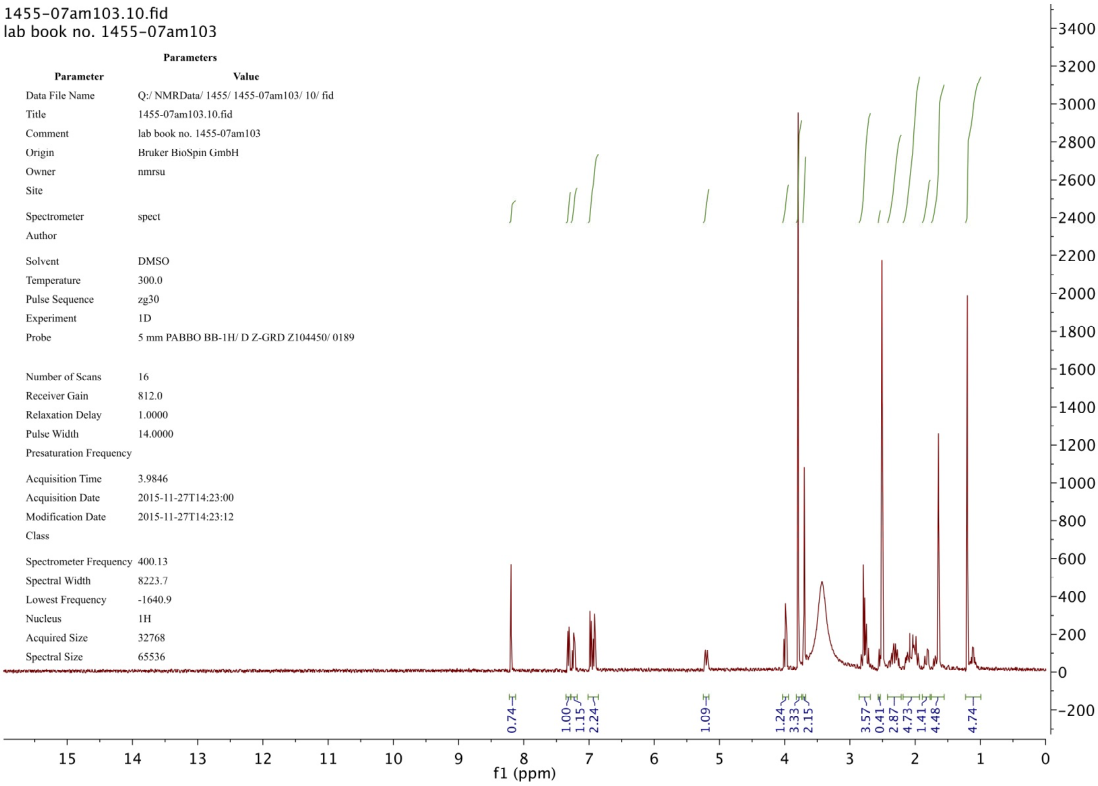

Supplementary Figure 100. *Proton NMR spectrum of* ***4p.***

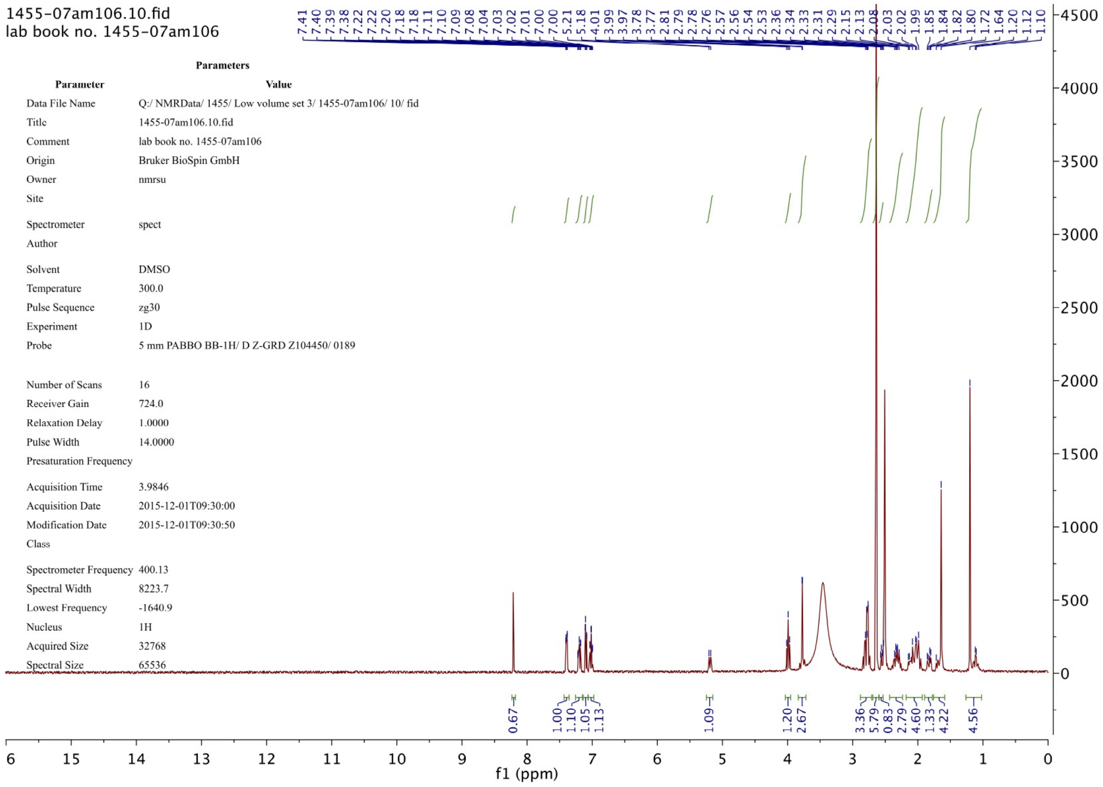

Supplementary Figure 101. *Proton NMR spectrum of* ***4q.***

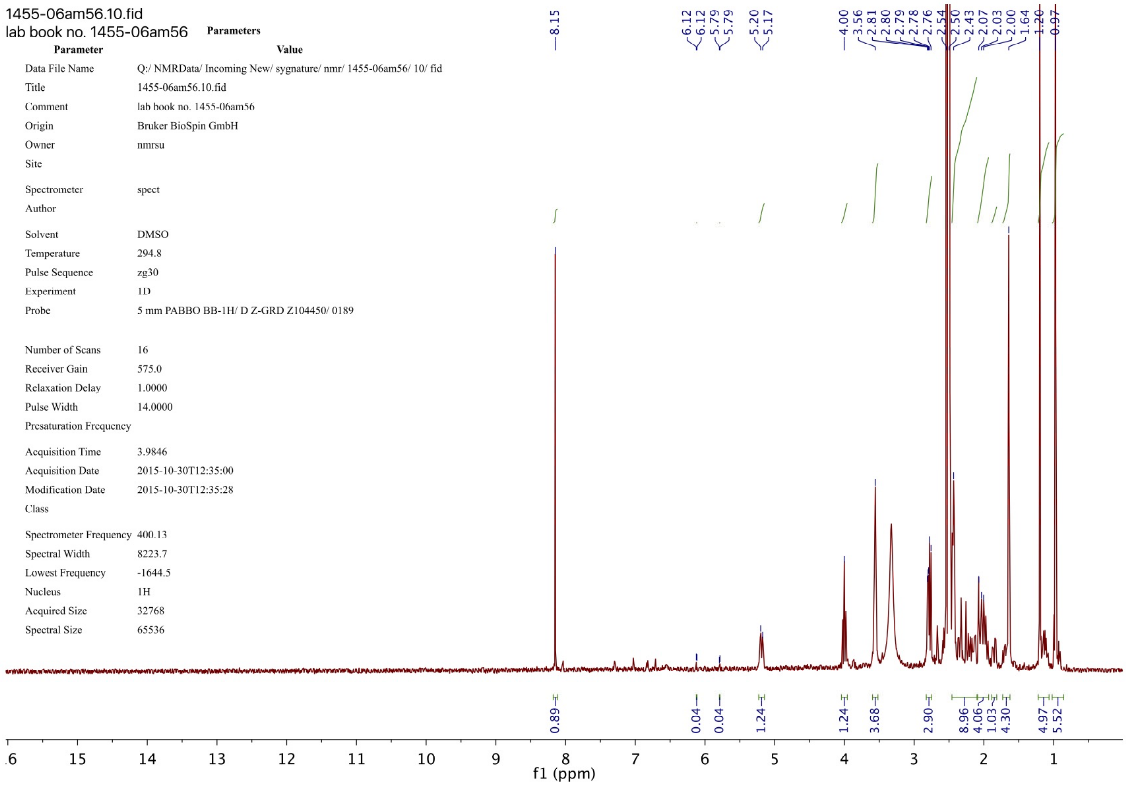

Supplementary Figure 102. *Proton NMR spectrum of* ***4r.***

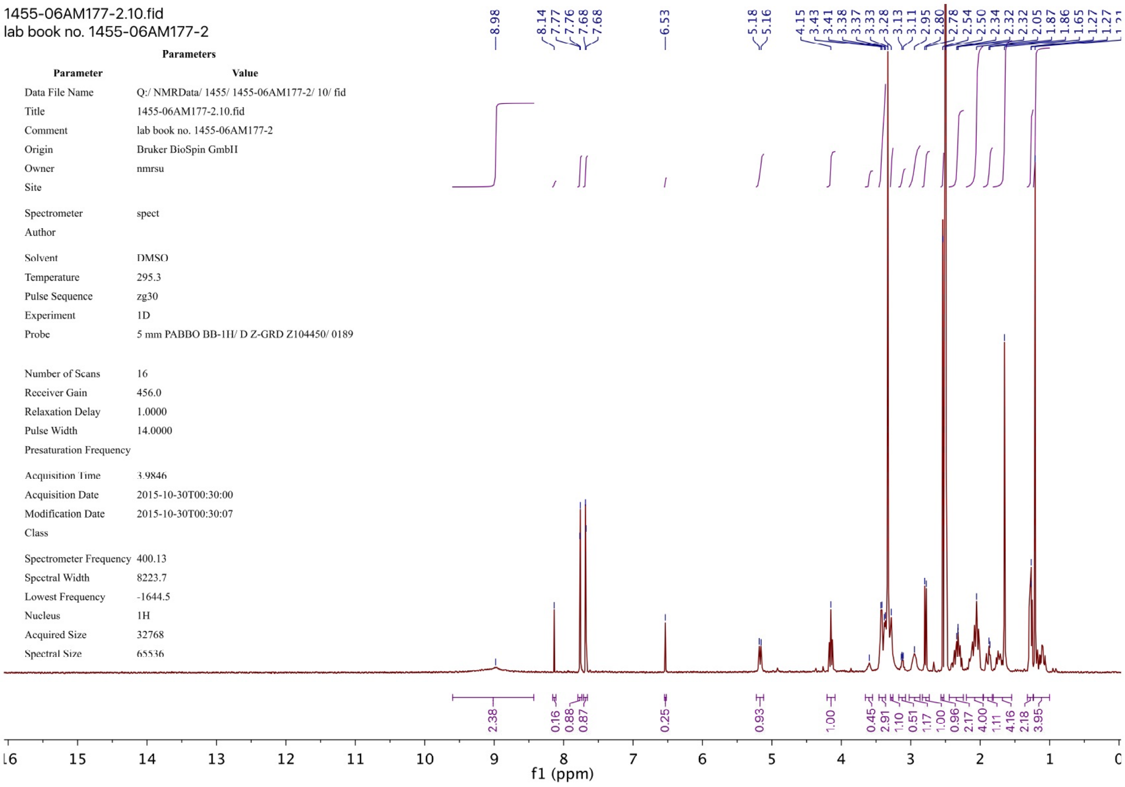

Supplementary Figure 103. *Proton NMR spectrum of* ***4s.***

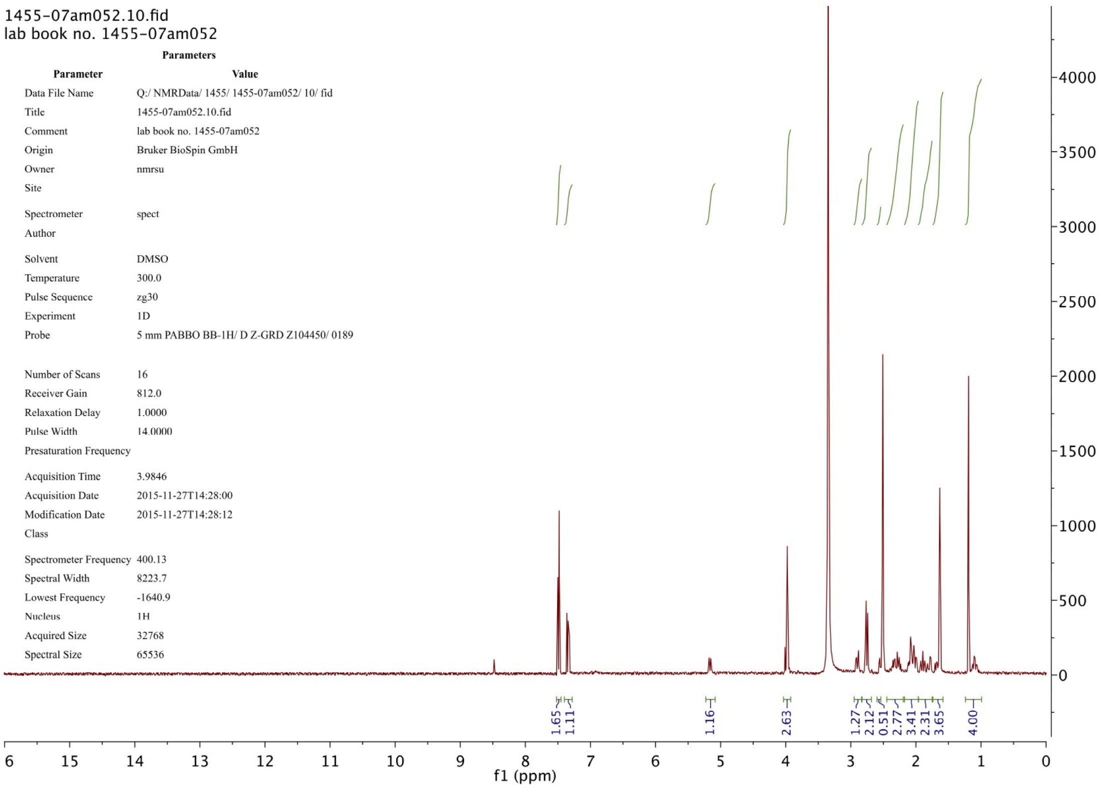

Supplementary Figure 104. *Proton NMR spectrum of* ***4t.***

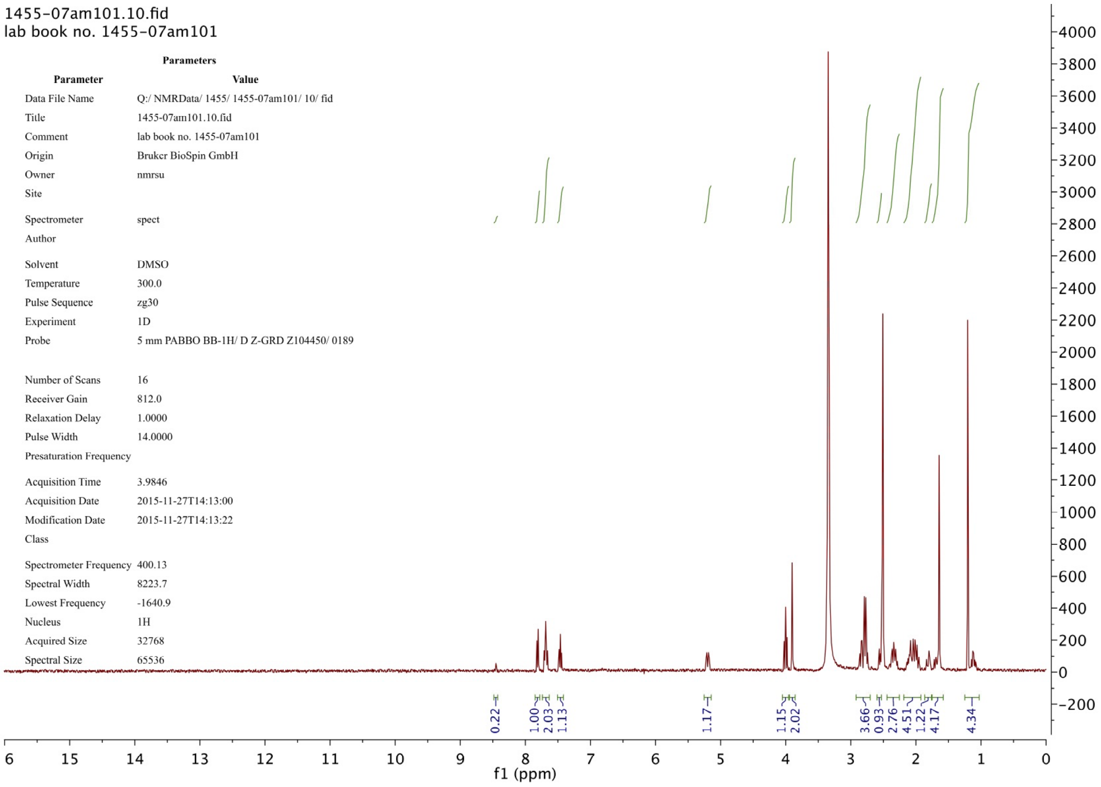

Supplementary Figure 105. *Proton NMR spectrum of* ***4u.***

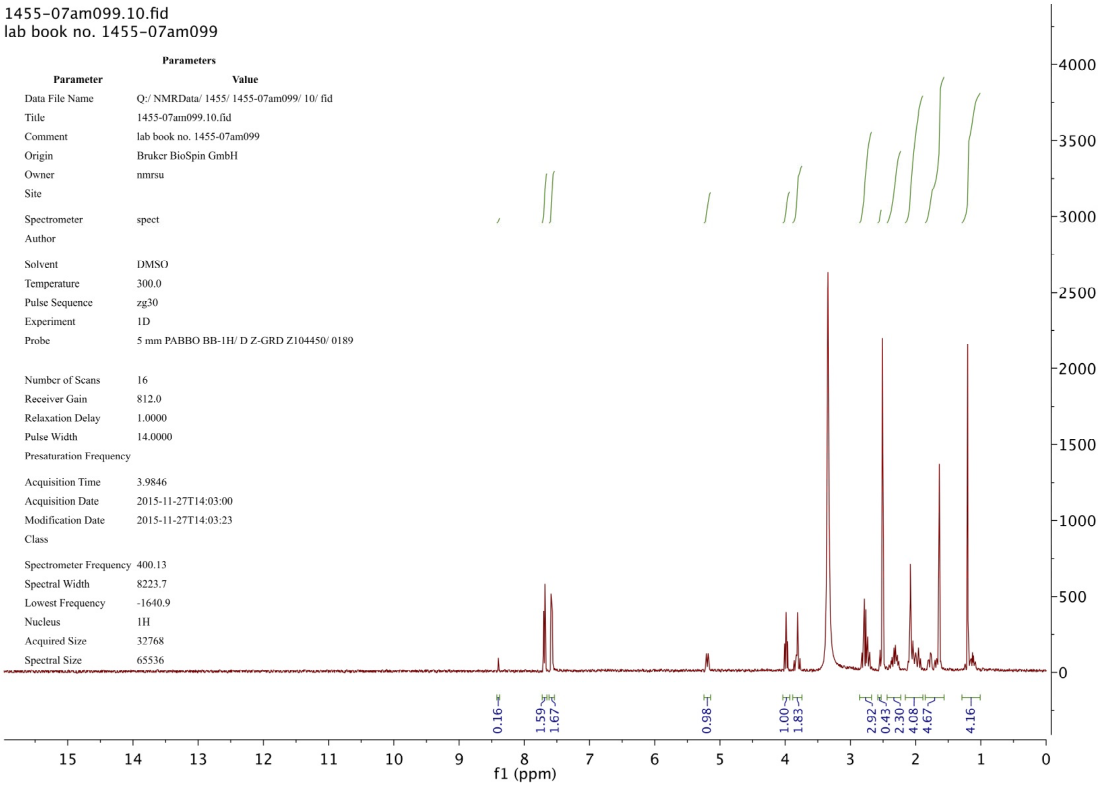

Supplementary Figure 106. *Proton NMR spectrum of* ***4v.***

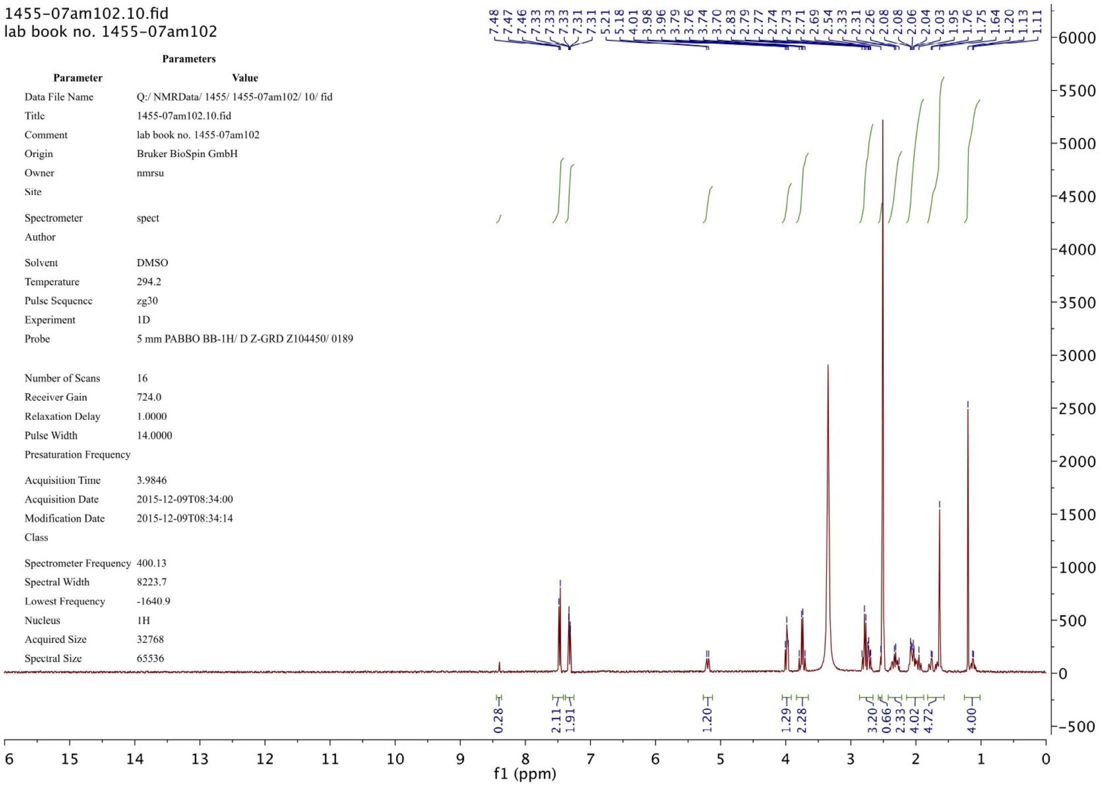

Supplementary Figure 107. *Proton NMR spectrum of* ***4w.***

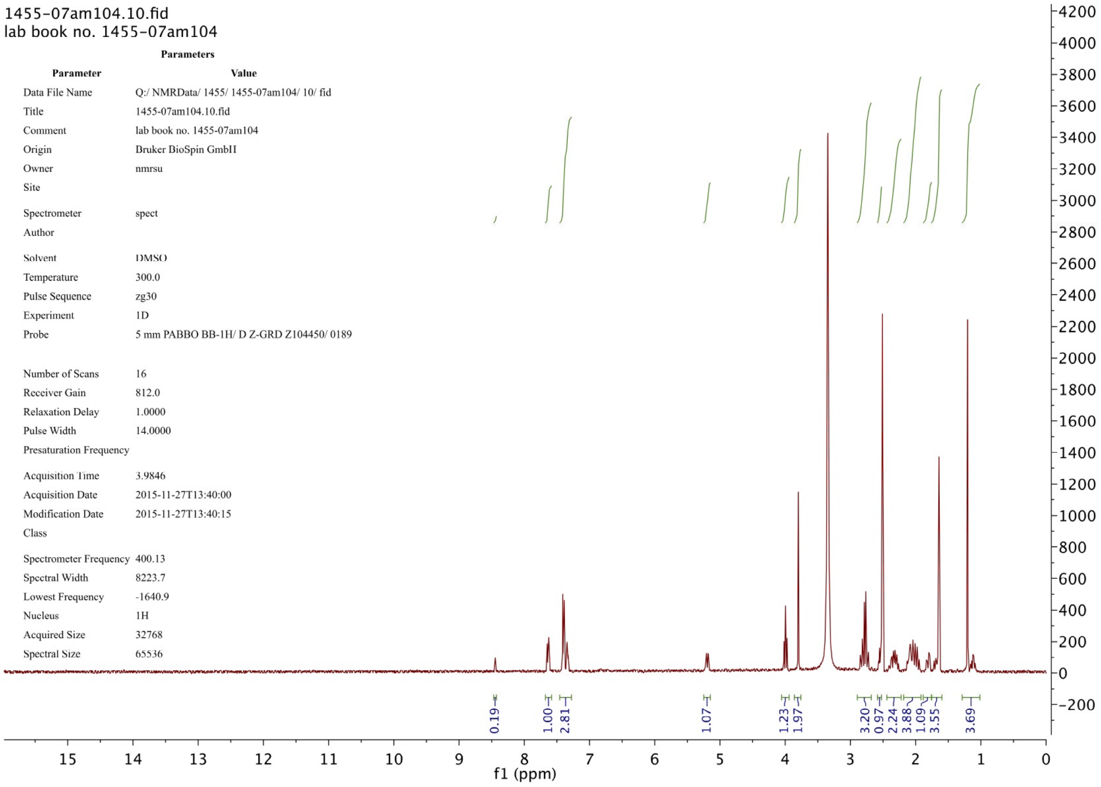

Supplementary Figure 108. *Proton NMR spectrum of* ***4x.***

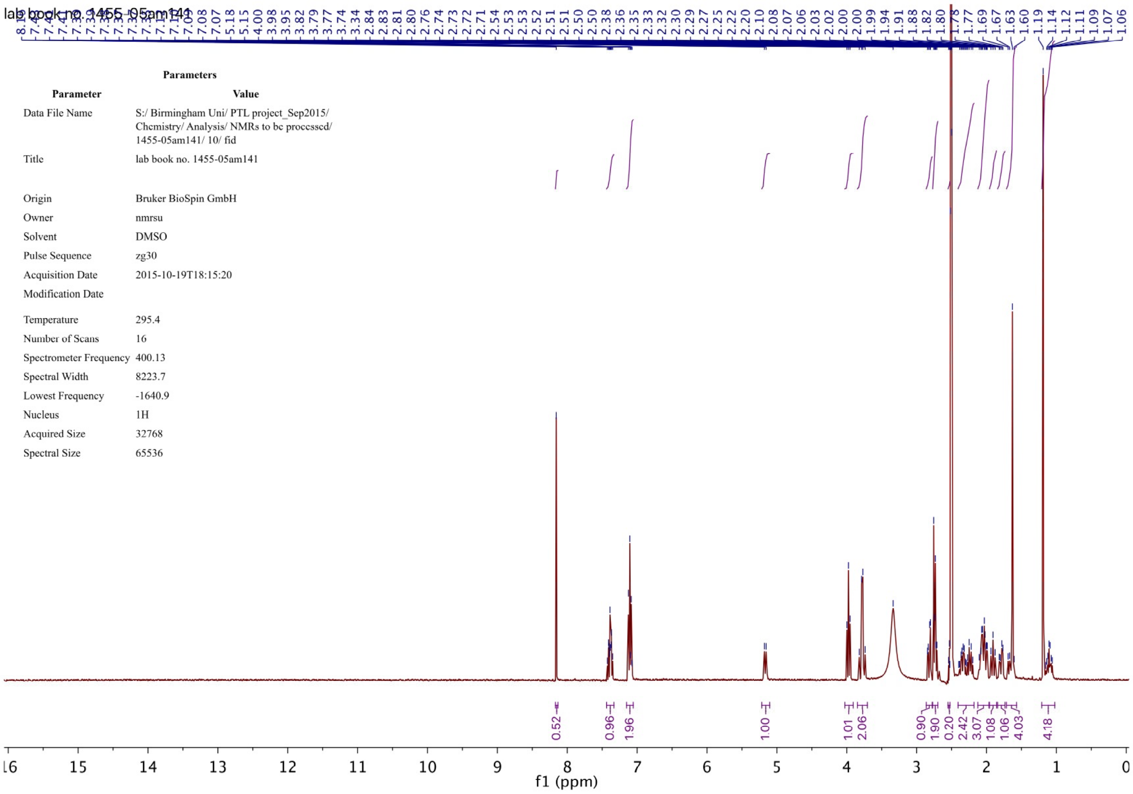

Supplementary Figure 109. *Proton NMR spectrum of* ***4y.***

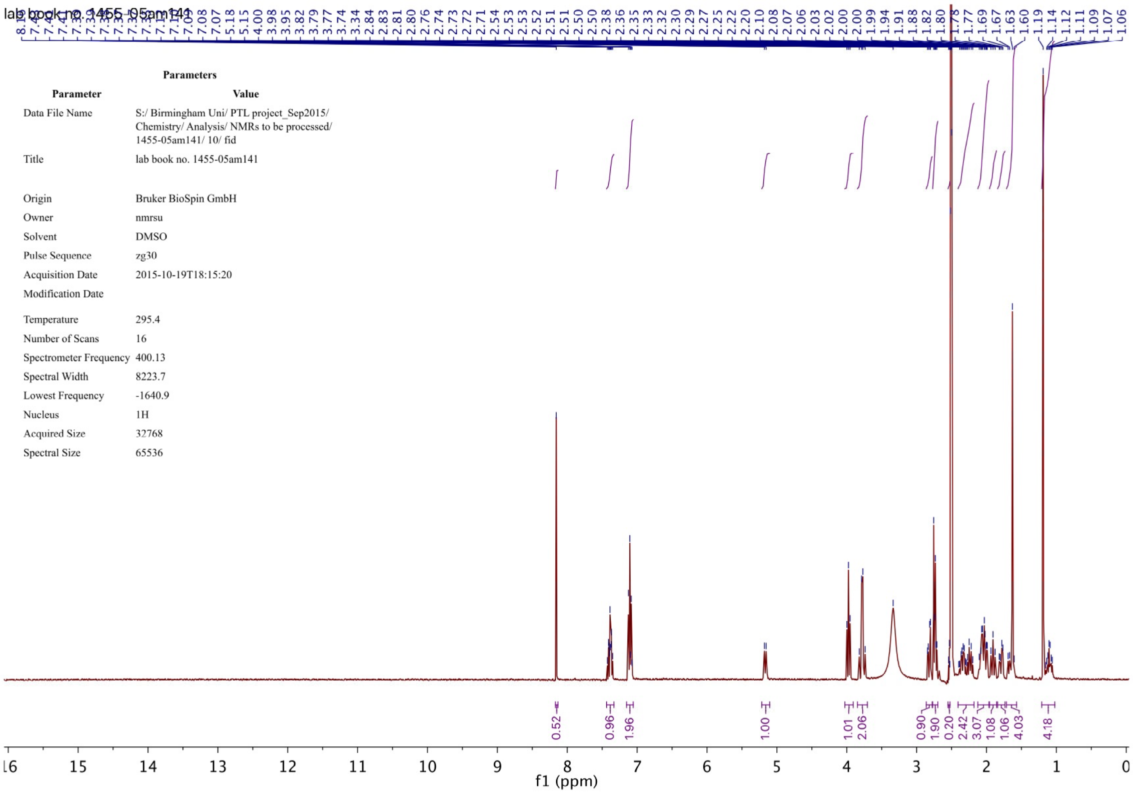

Supplementary Figure 110. *Proton NMR spectrum of* ***4z.***

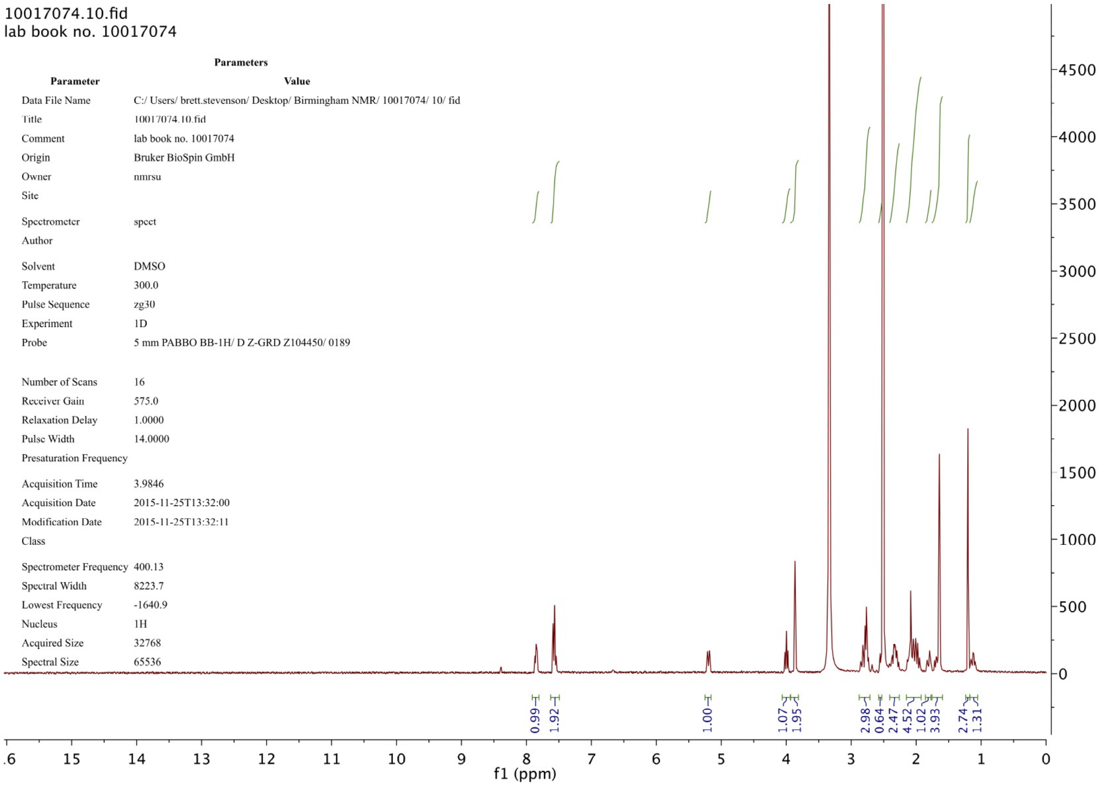

Supplementary Figure 111. *Proton NMR spectrum of* ***4aa.***

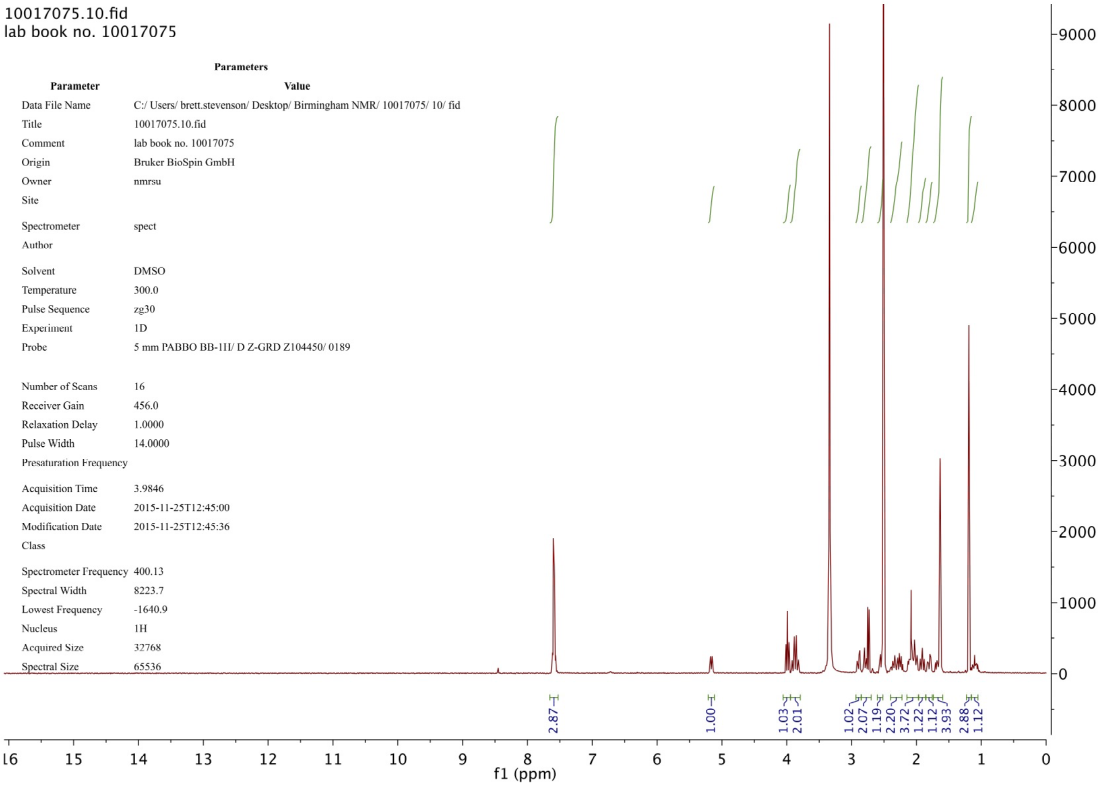

Supplementary Figure 112. *Proton NMR spectrum of* ***4ab.***

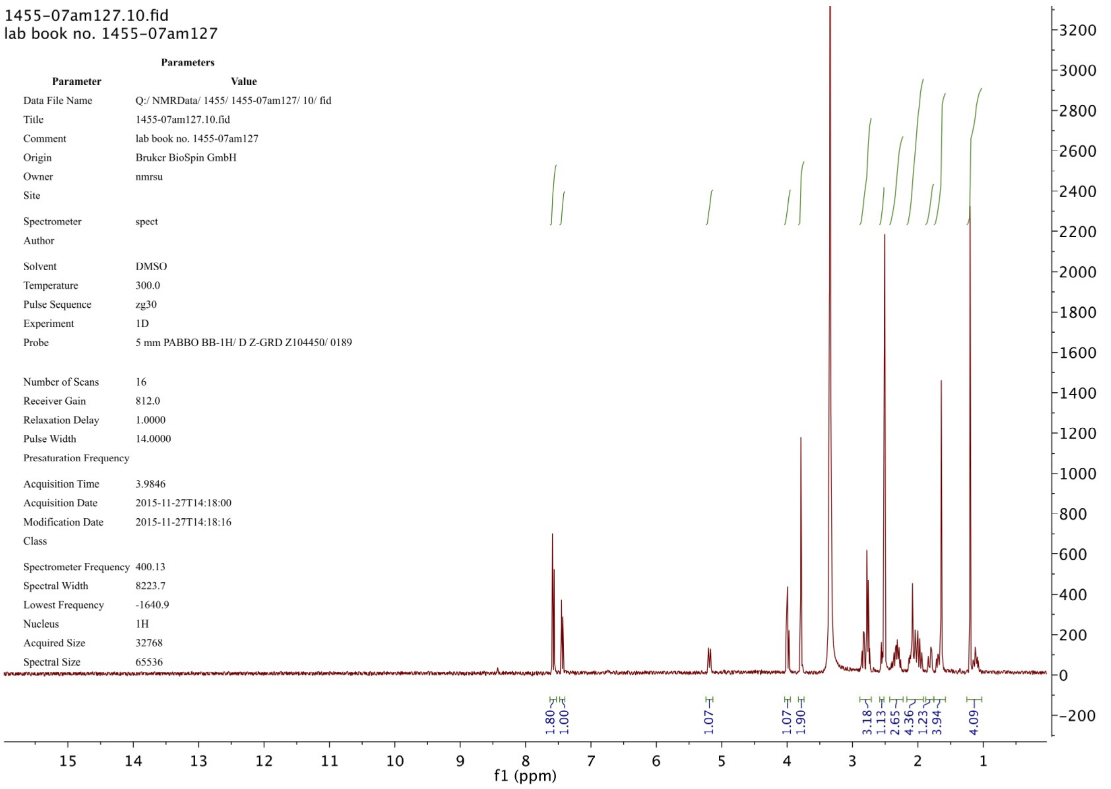

Supplementary Figure 113. *Proton NMR spectrum of* ***4ac.***

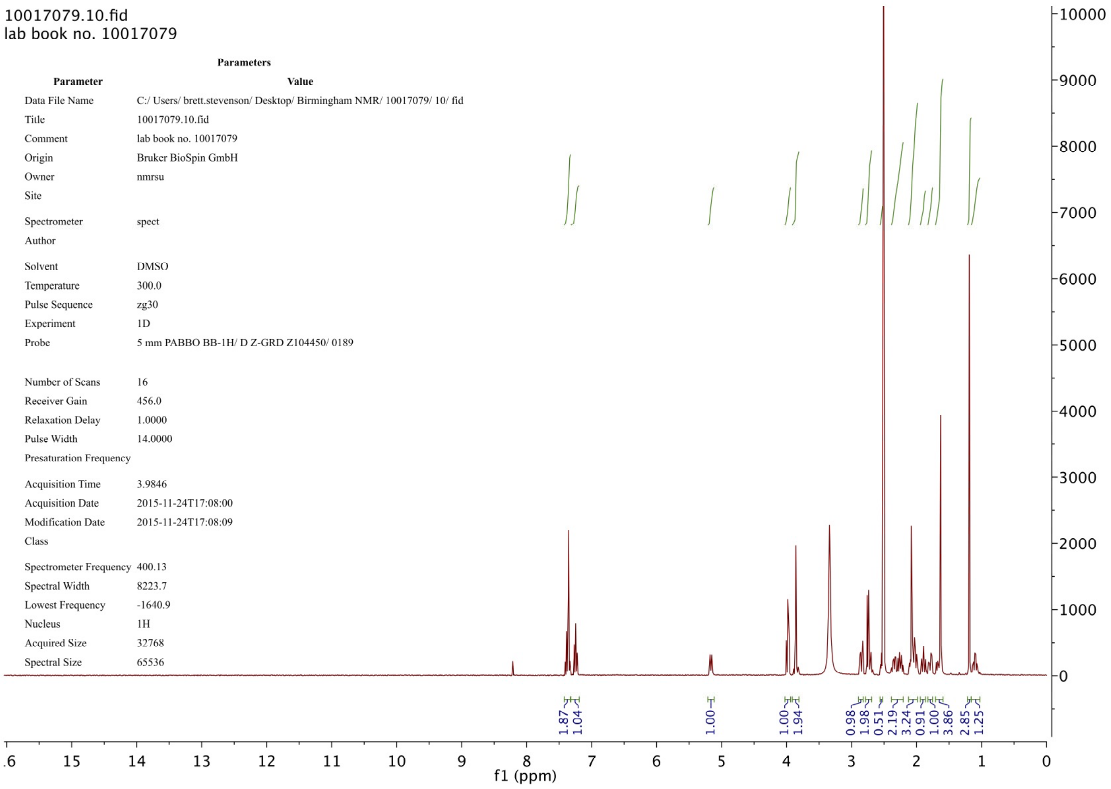

Supplementary Figure 114. *Proton NMR spectrum of* ***4ad.***

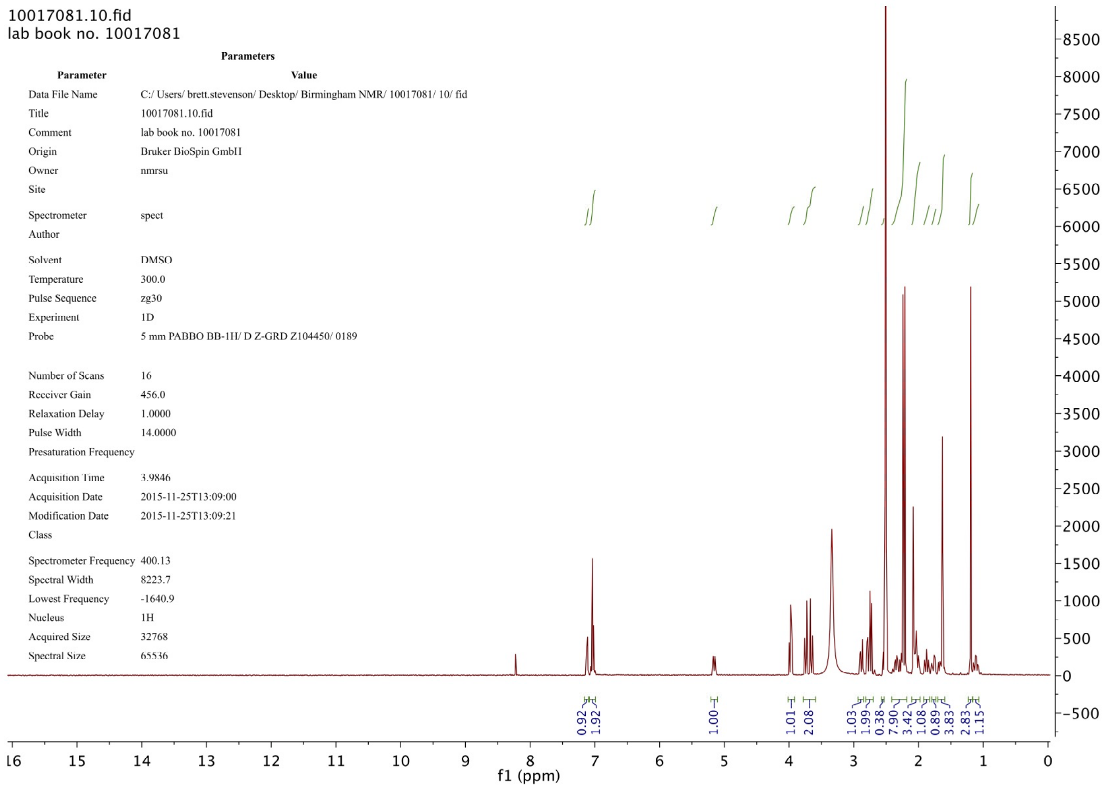

Supplementary Figure 115. *Proton NMR spectrum of* ***4ae.***

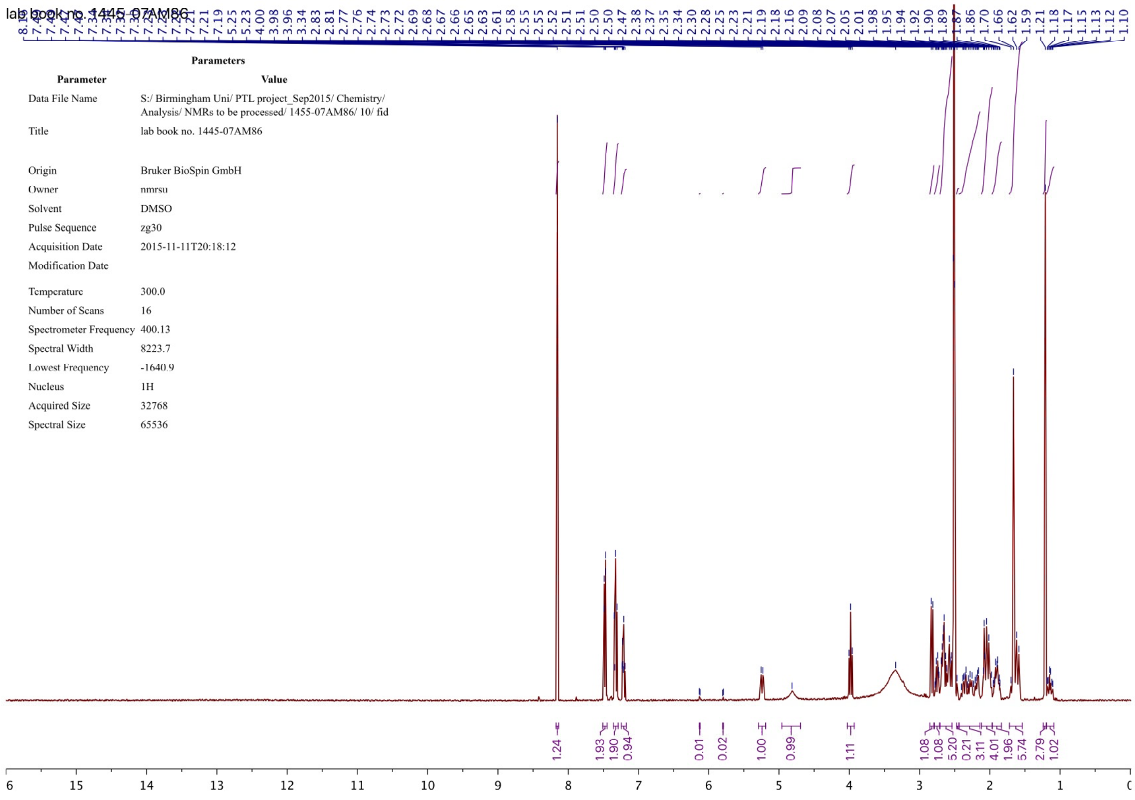

Supplementary Figure 116. *Proton NMR spectrum of* ***5d.***

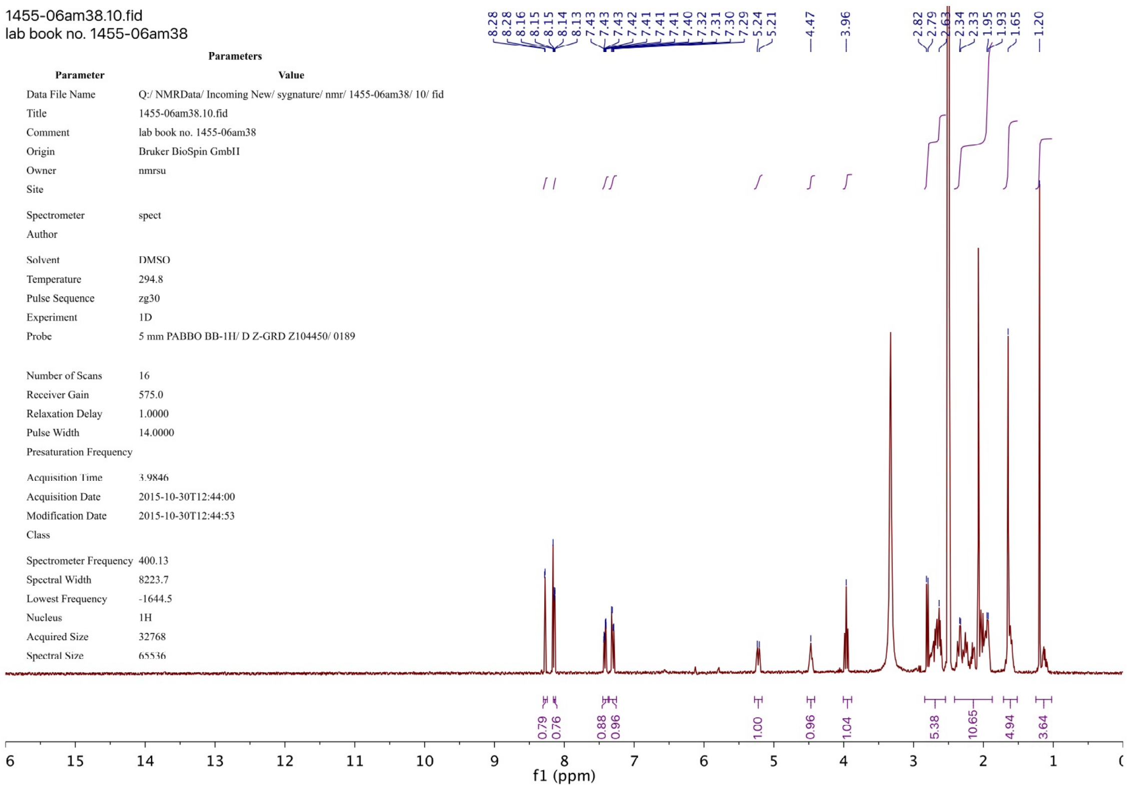

Supplementary Figure 117. *Proton NMR spectrum of* ***5e.***

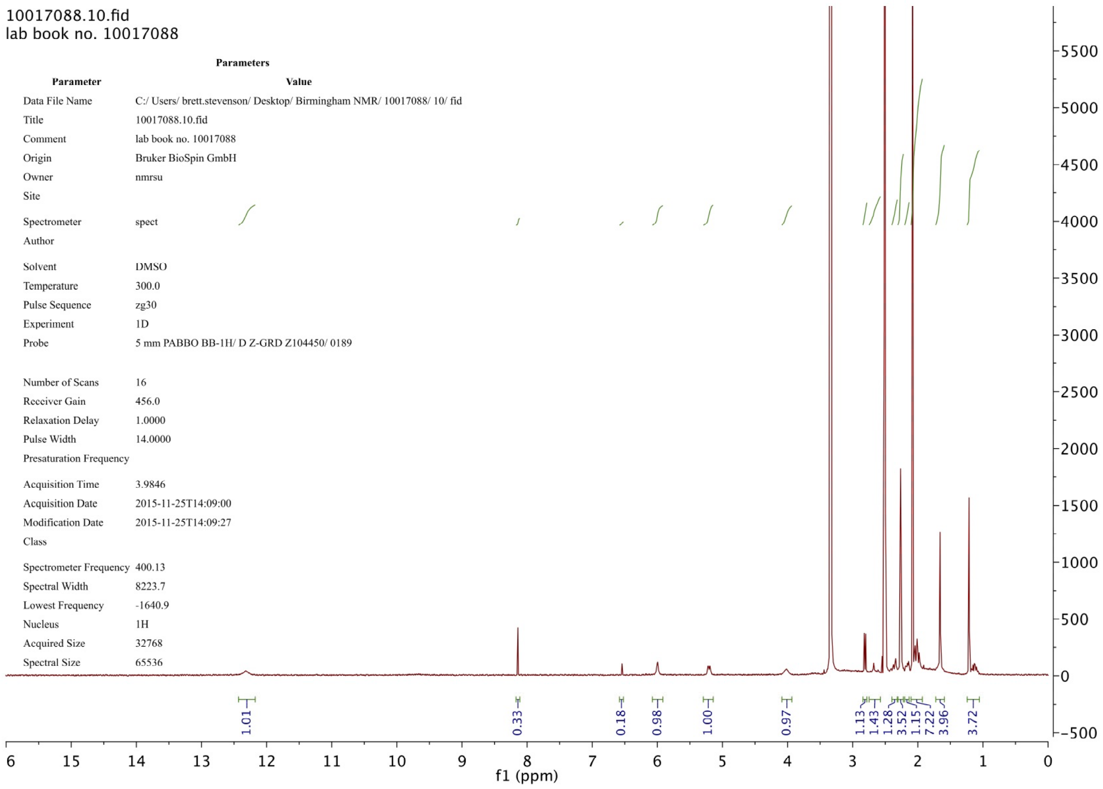

Supplementary Figure 118. *Proton NMR spectrum of* ***5f.***

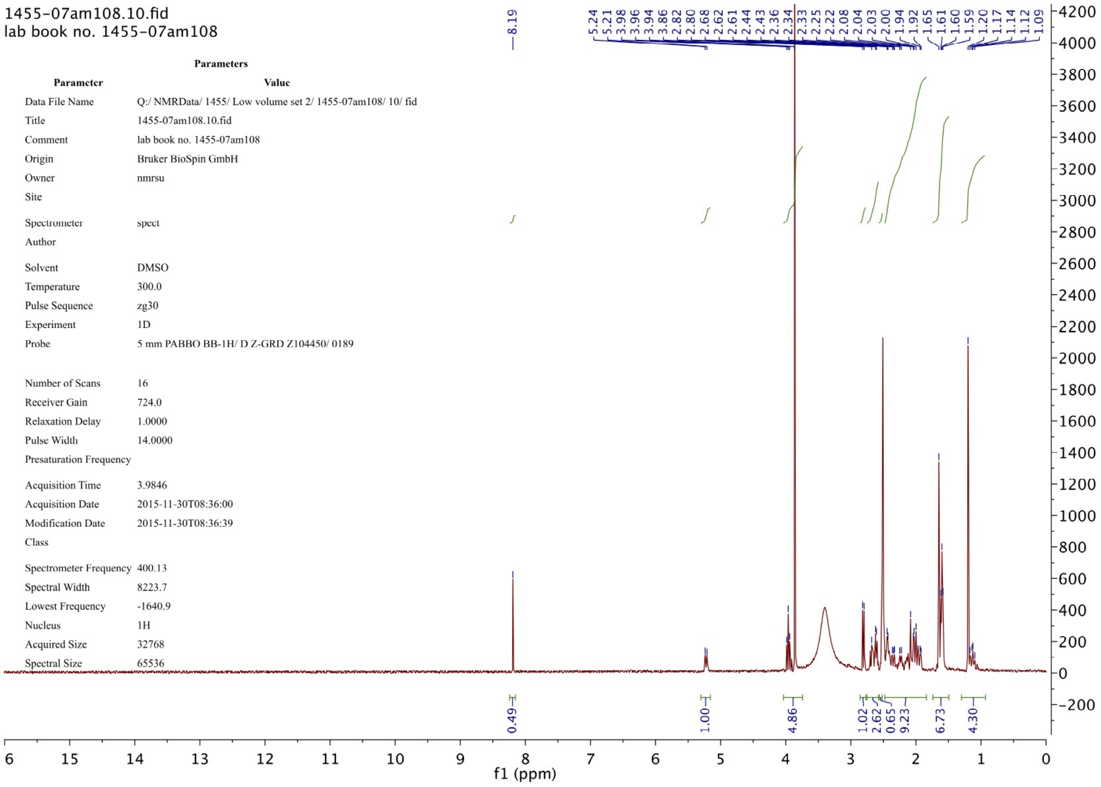

Supplementary Figure 119. *Proton NMR spectrum of* ***5g.***

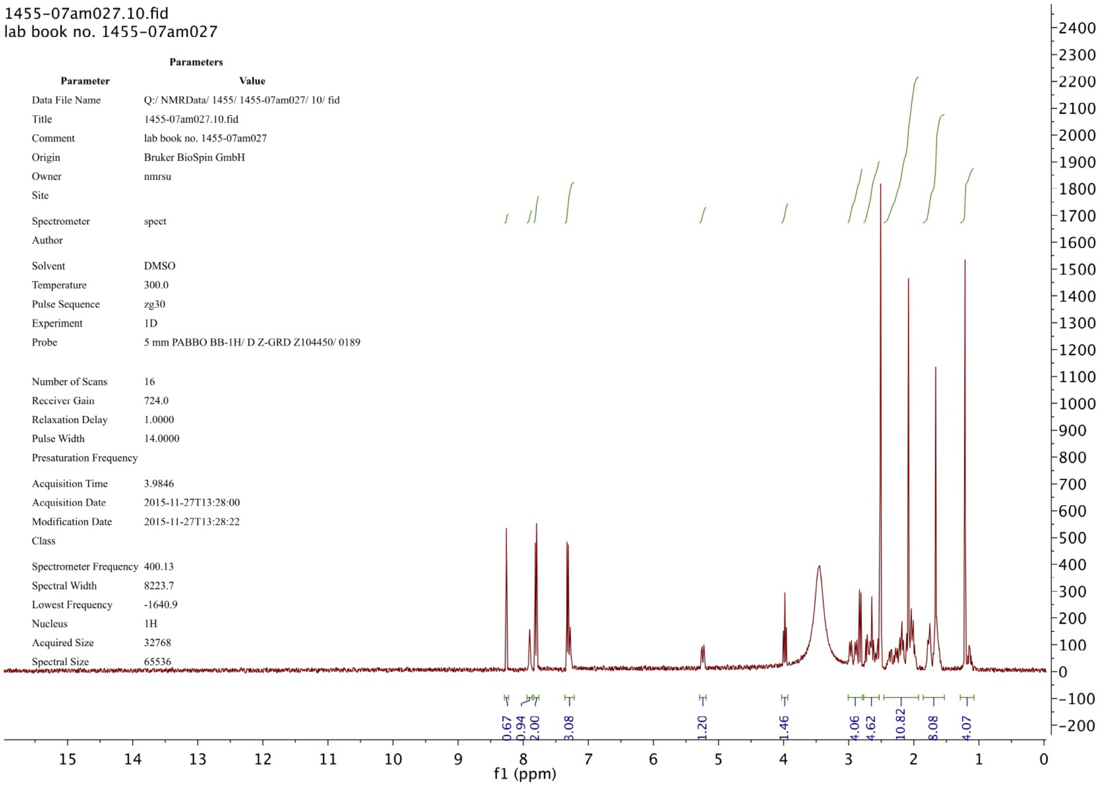

Supplementary Figure 120. *Proton NMR spectrum of* ***5h.***

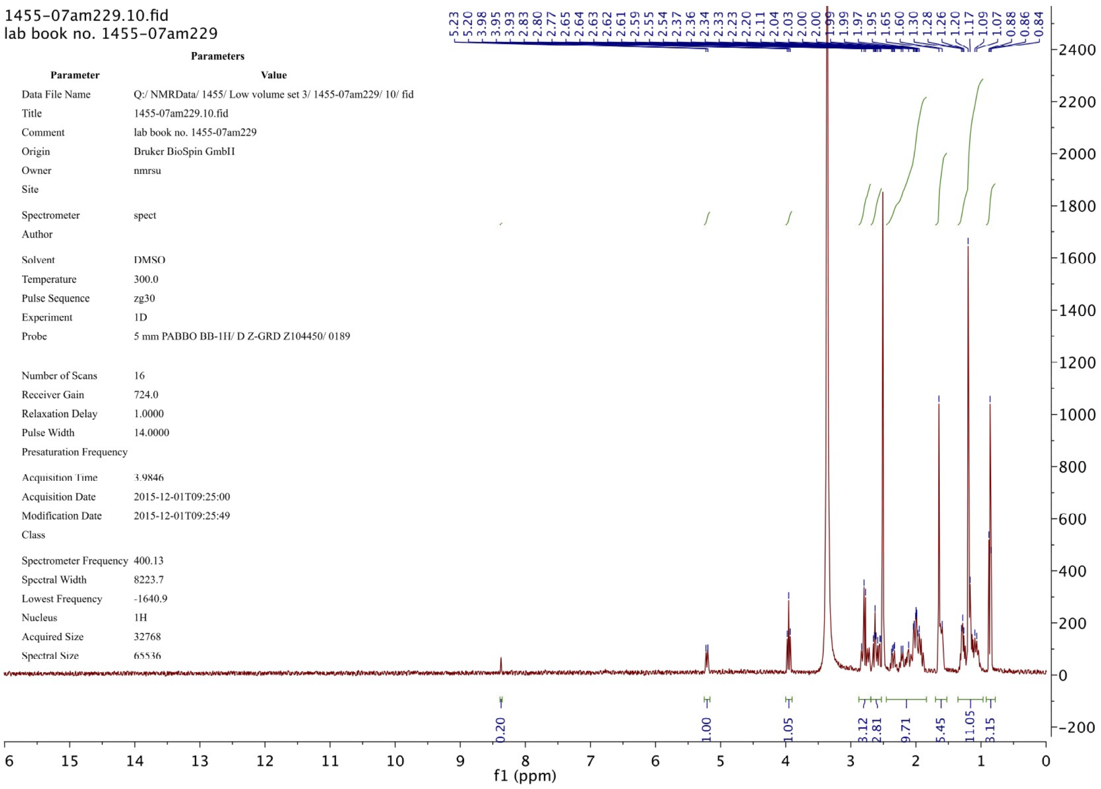

Supplementary Figure 121. Proton NMR spectrum of **5i**.

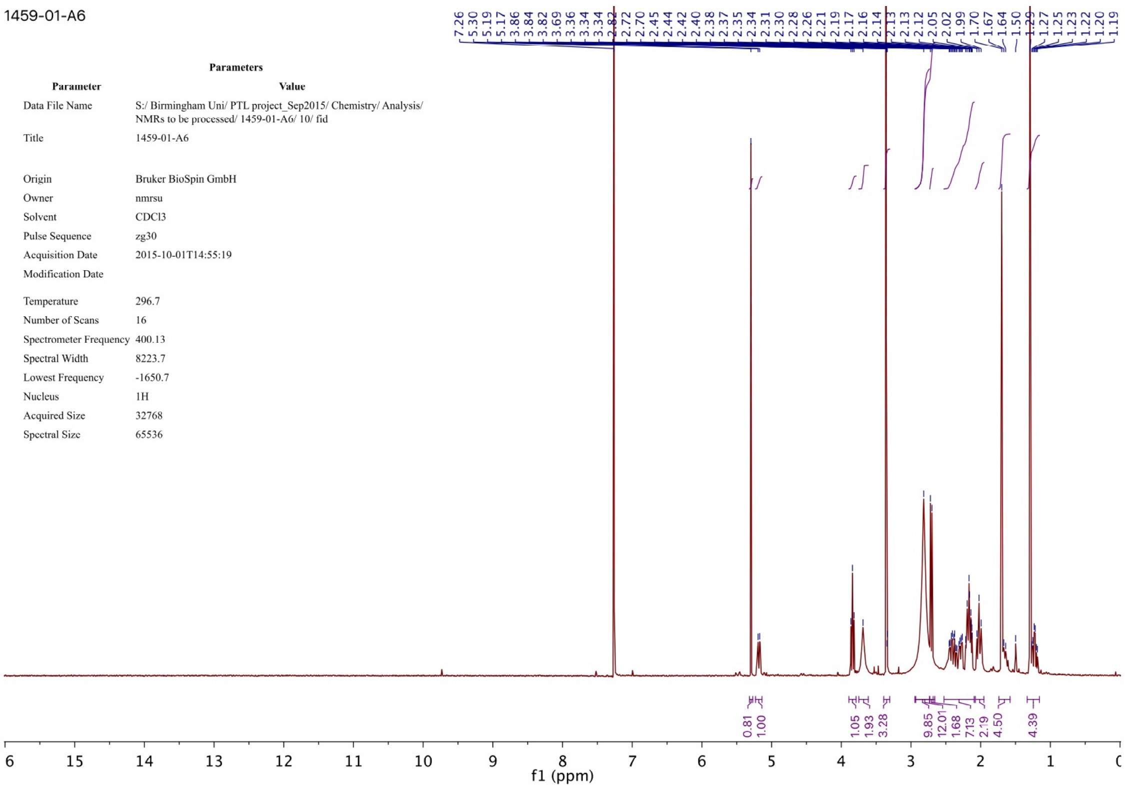

Supplementary Figure 122. Proton NMR spectrum of **5j**.

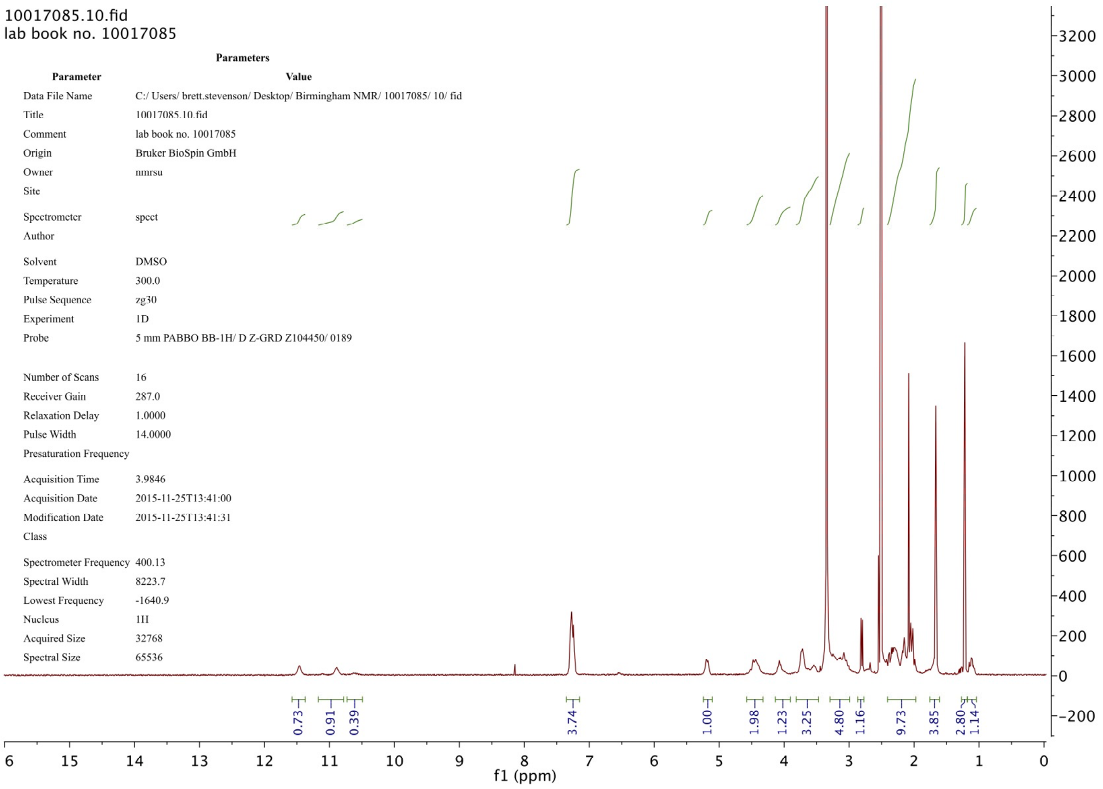

Supplementary Figure 123. *Proton NMR spectrum of* ***5k*****.**

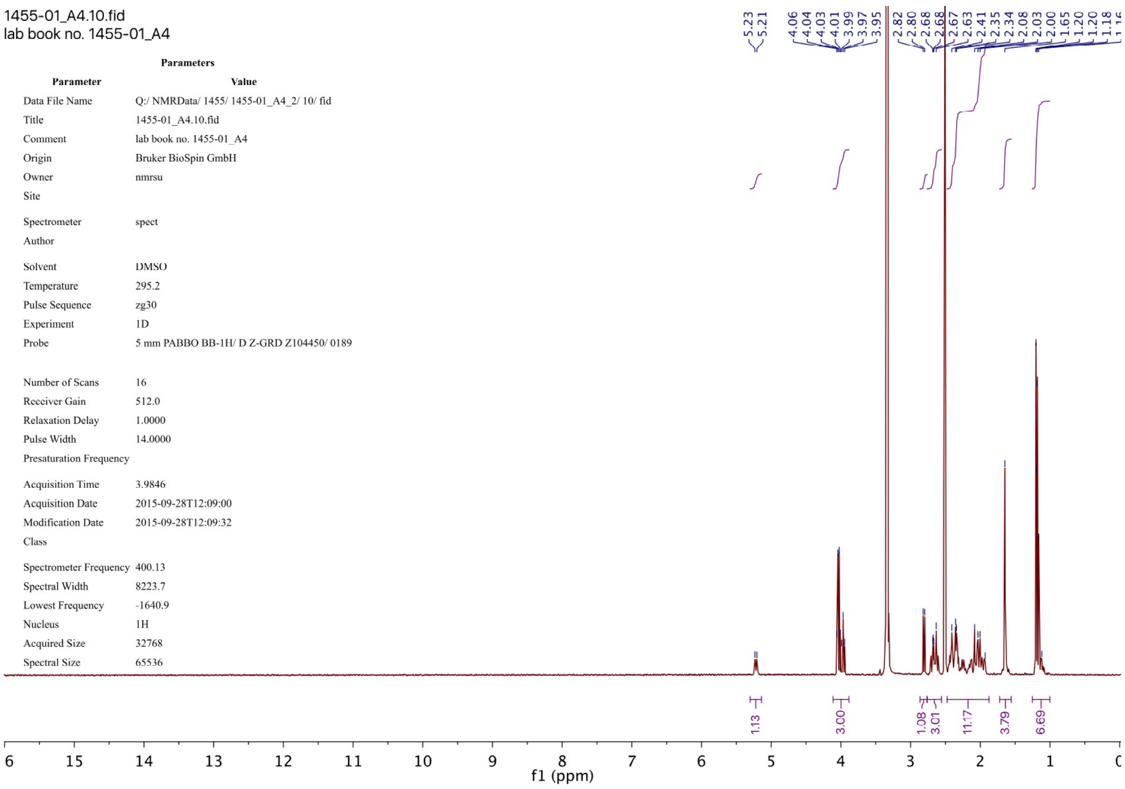

Supplementary Figure 124. *Proton NMR spectrum of* ***5l***

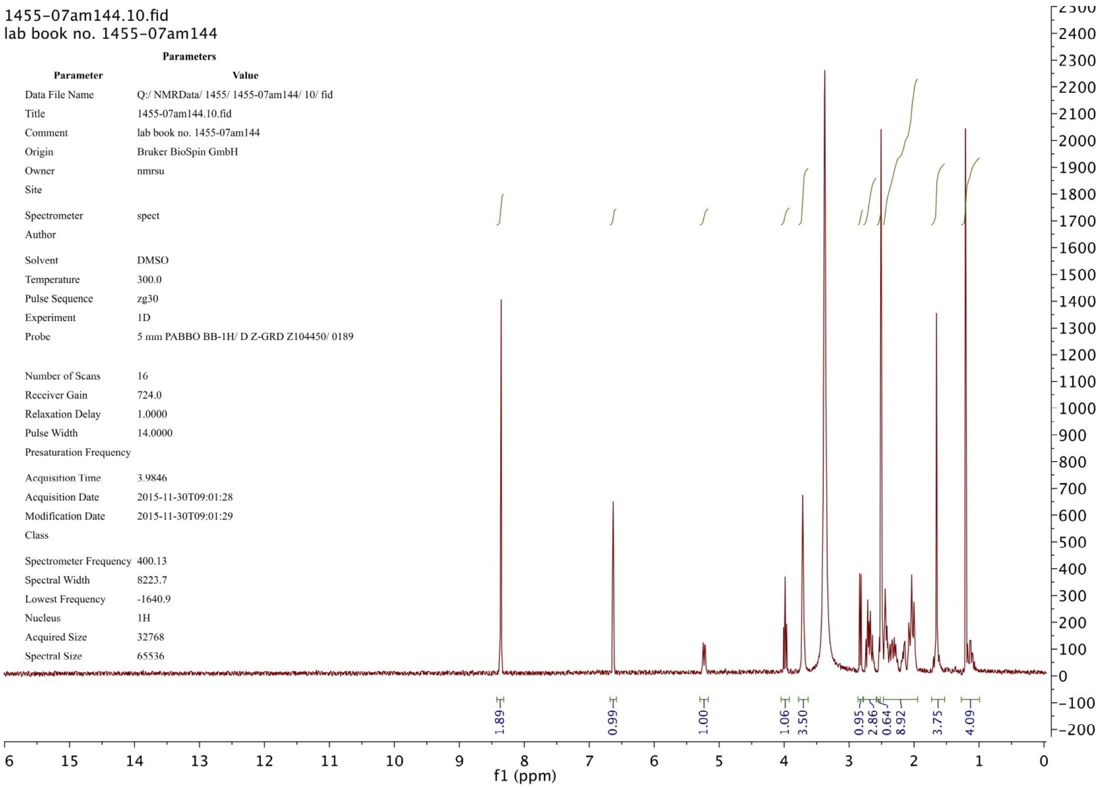

Supplementary Figure 125. *Proton NMR spectrum of* ***5m.***

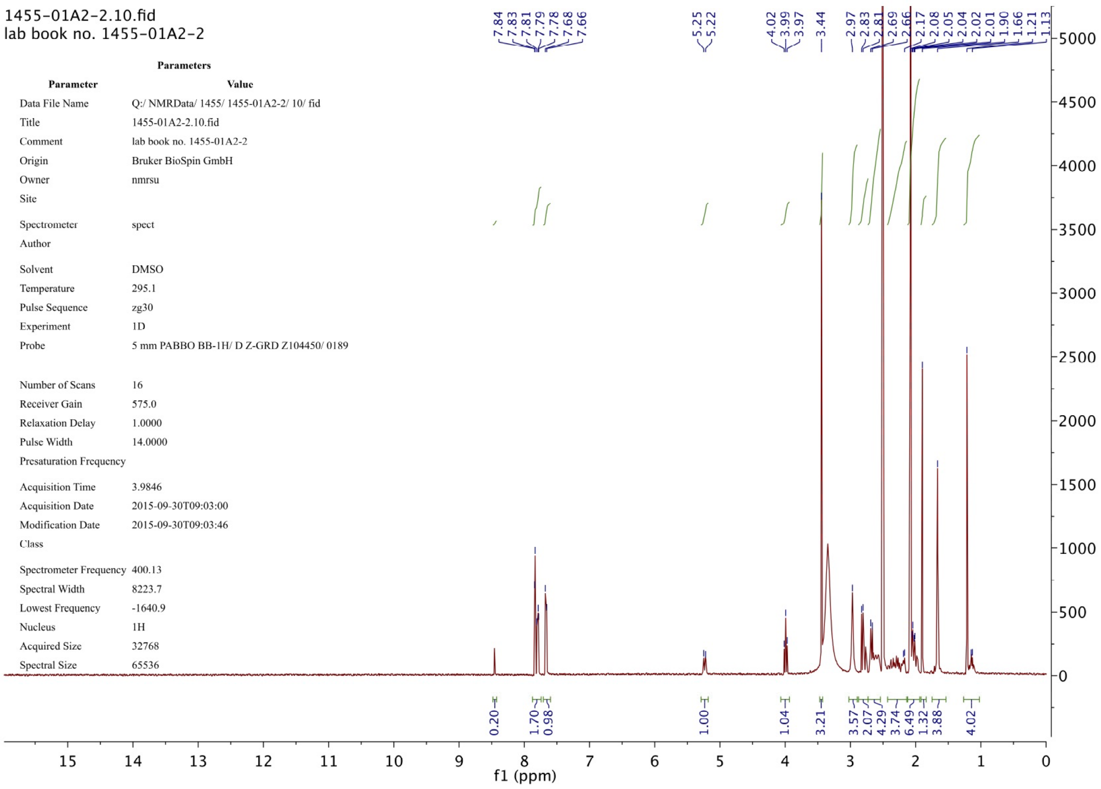

Supplementary Figure 126. *Proton NMR spectrum of* ***5n.***

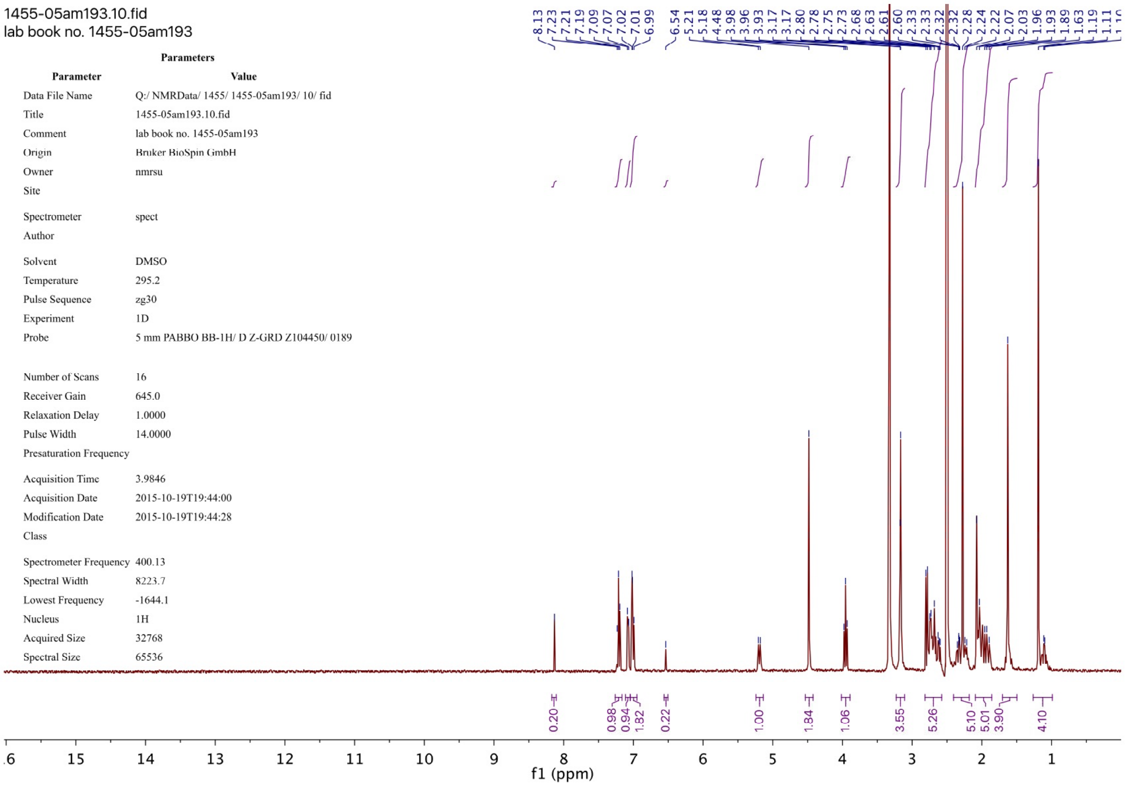

Supplementary Figure 127. *Proton NMR spectrum of* ***5o.***

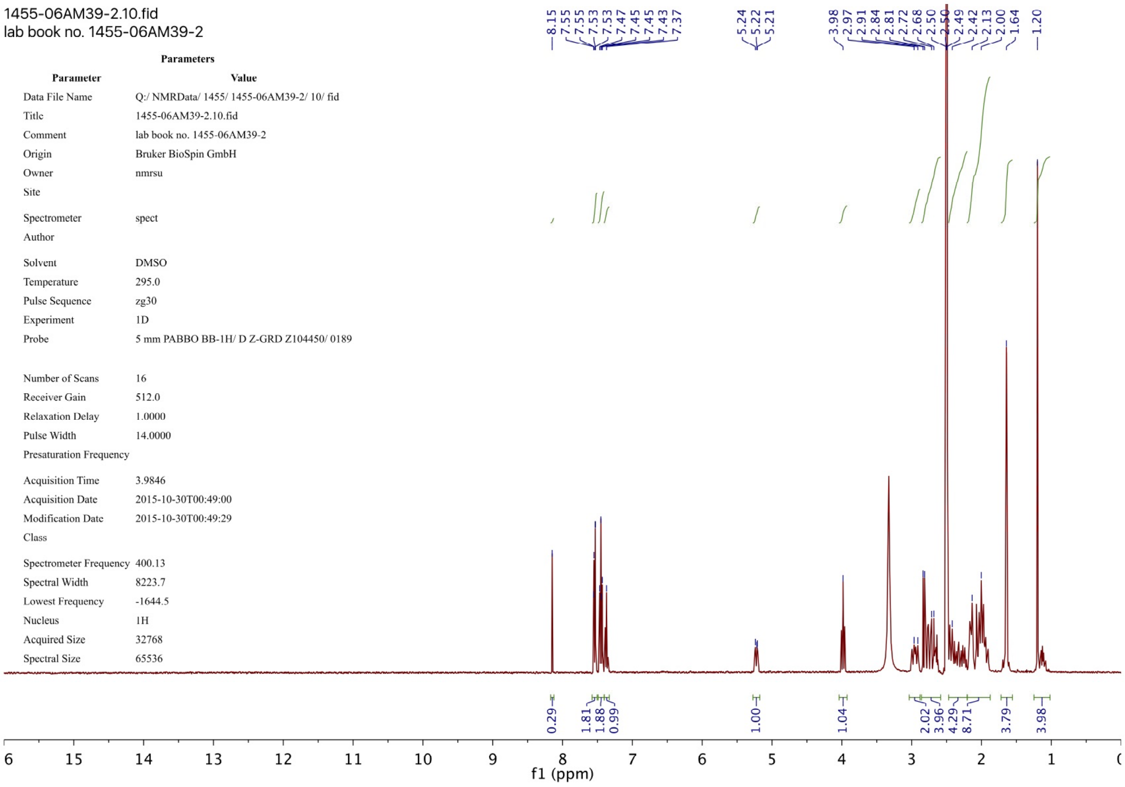

Supplementary Figure 128. *Proton NMR spectrum of* ***5p.***

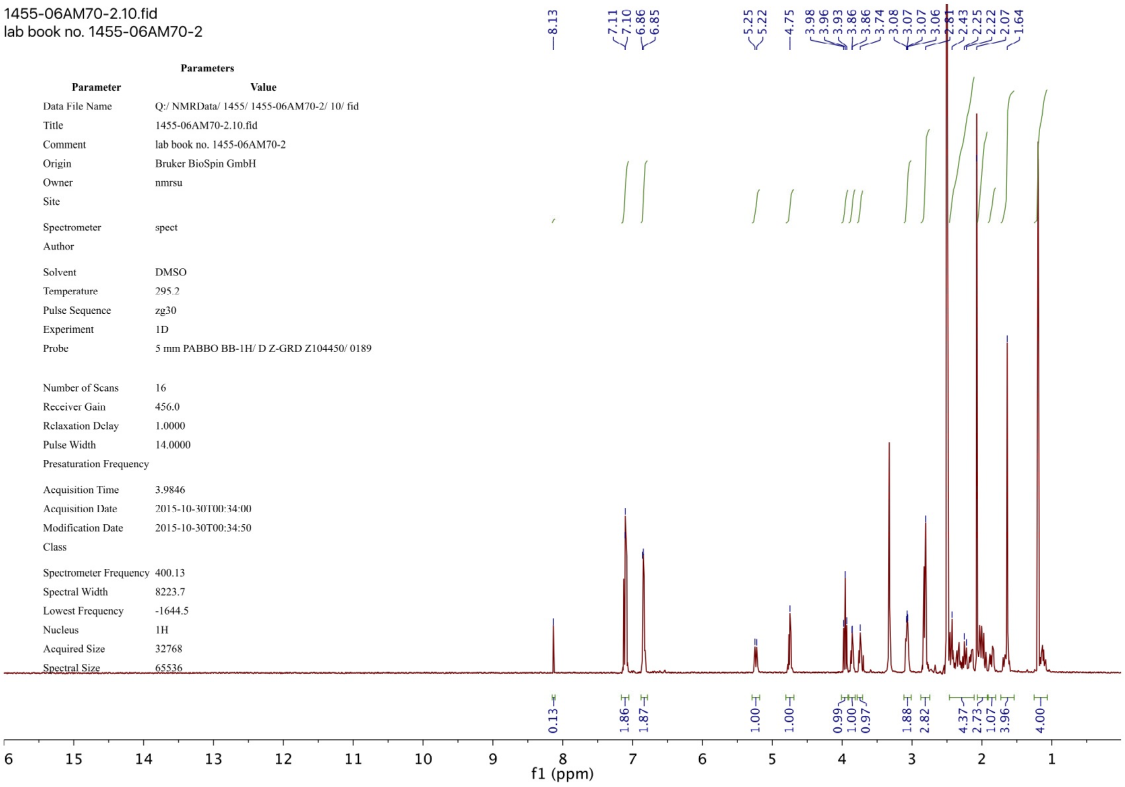

Supplementary Figure 129. *Proton NMR spectrum of* ***5q.***

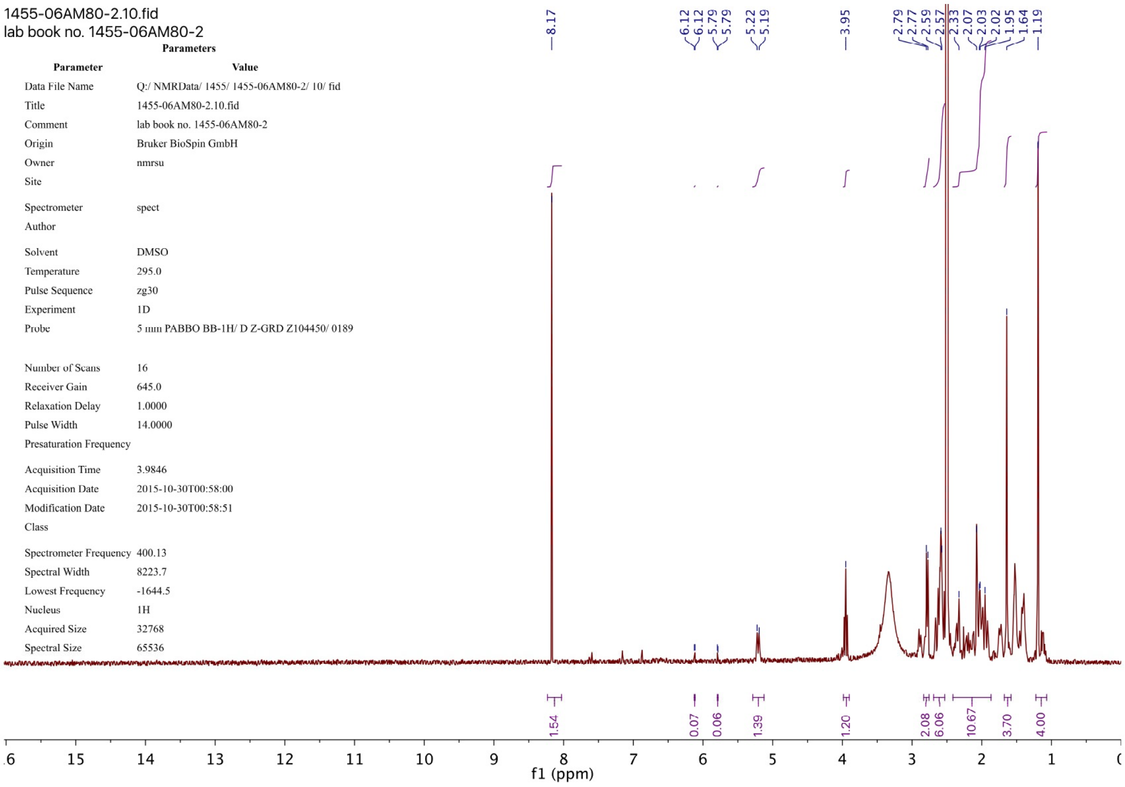

Supplementary Figure 130. *Proton NMR spectrum of* ***5r.***

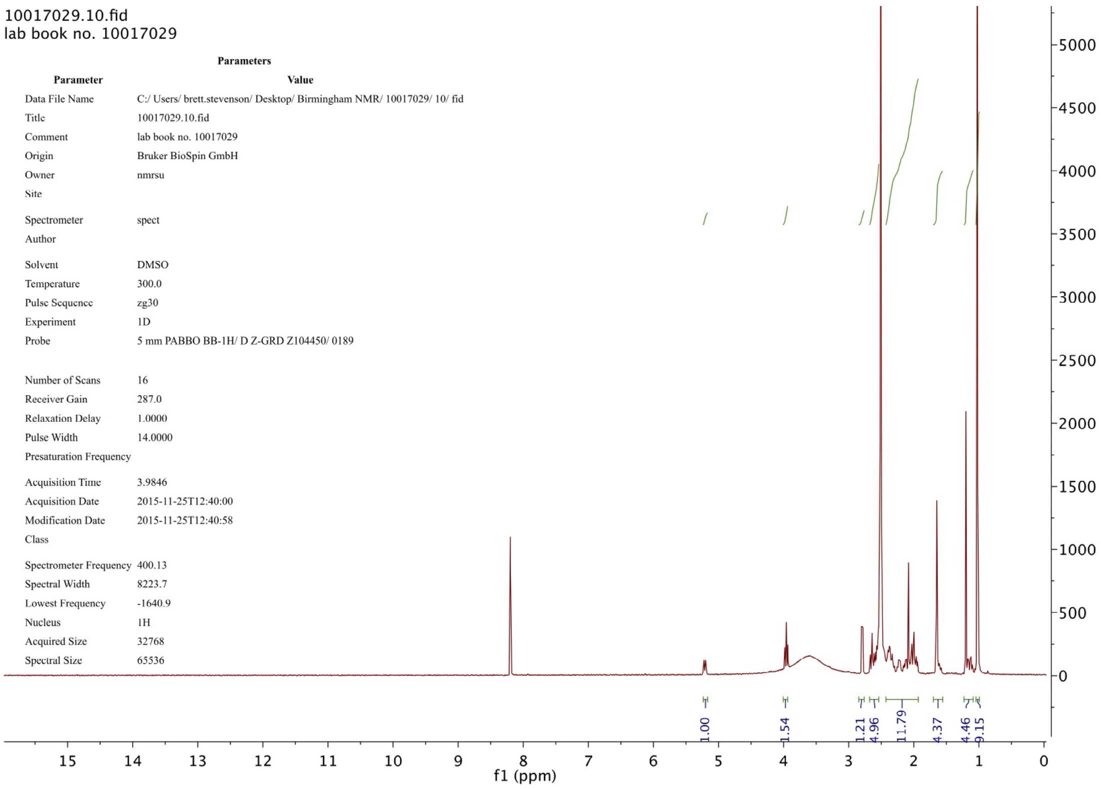

Supplementary Figure 131. *Proton NMR spectrum of **5s**.*

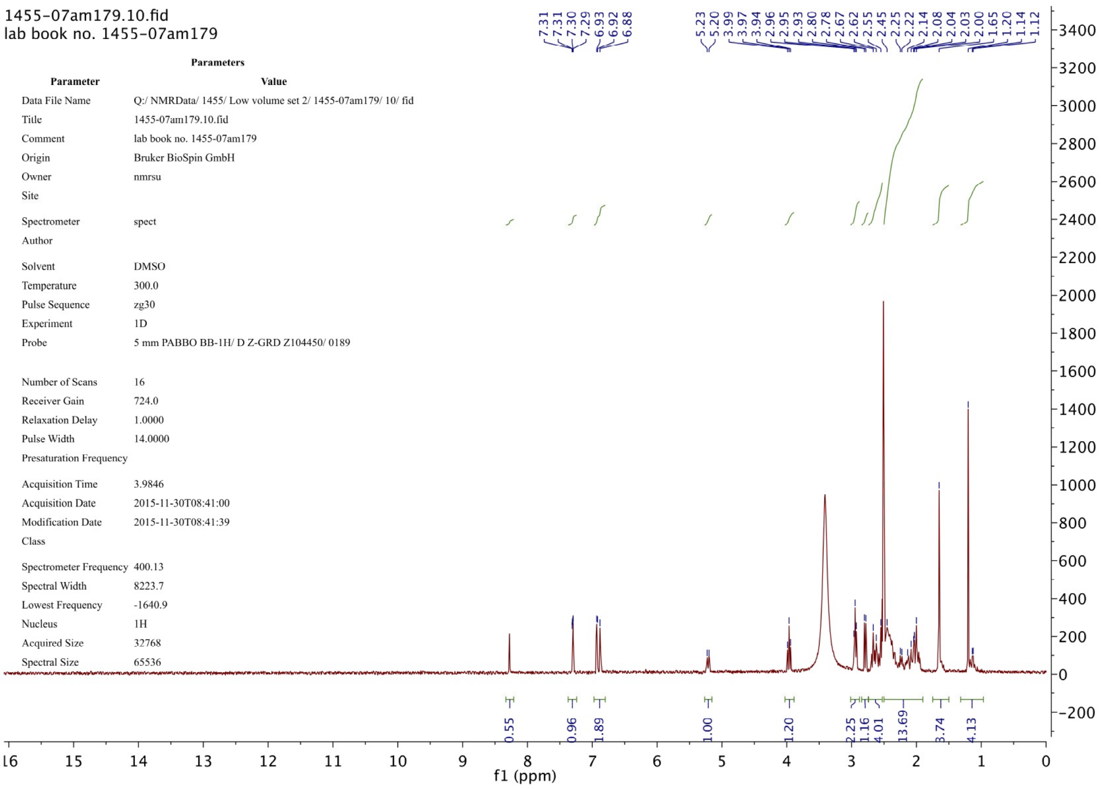

Supplementary Figure 132. *Proton NMR spectrum of **5t**.*

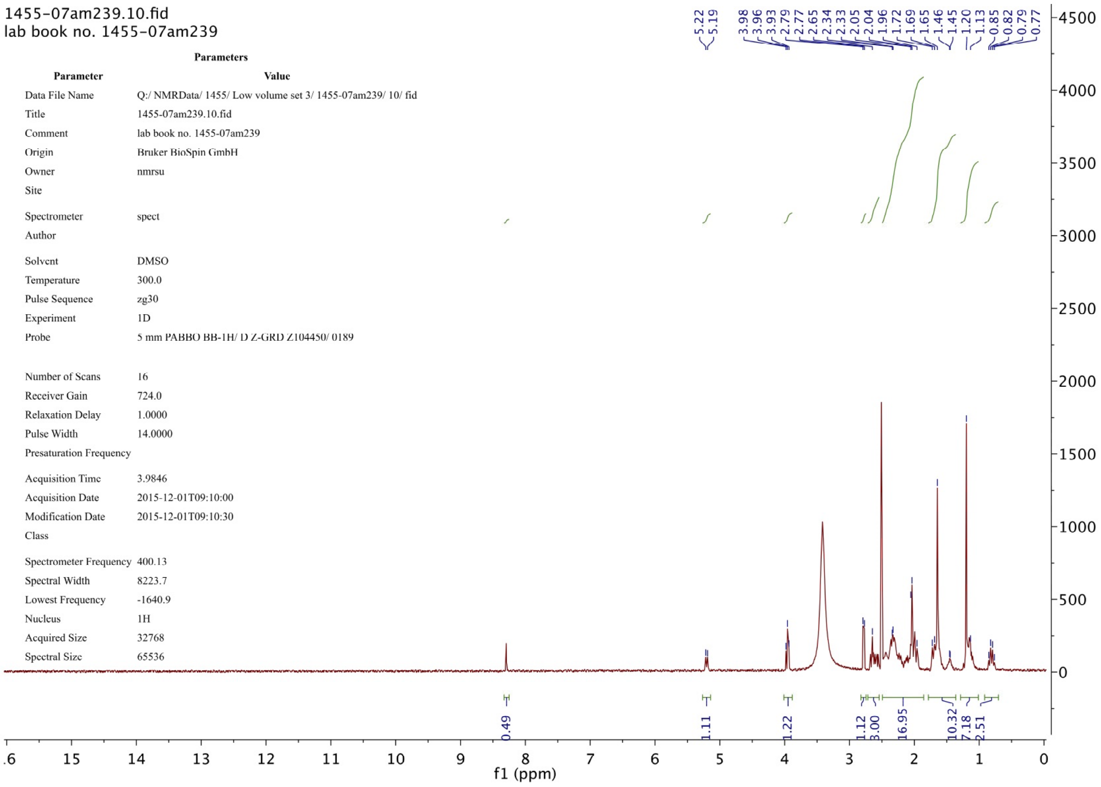

Supplementary Figure 133. *Proton NMR spectrum of* ***5u.***

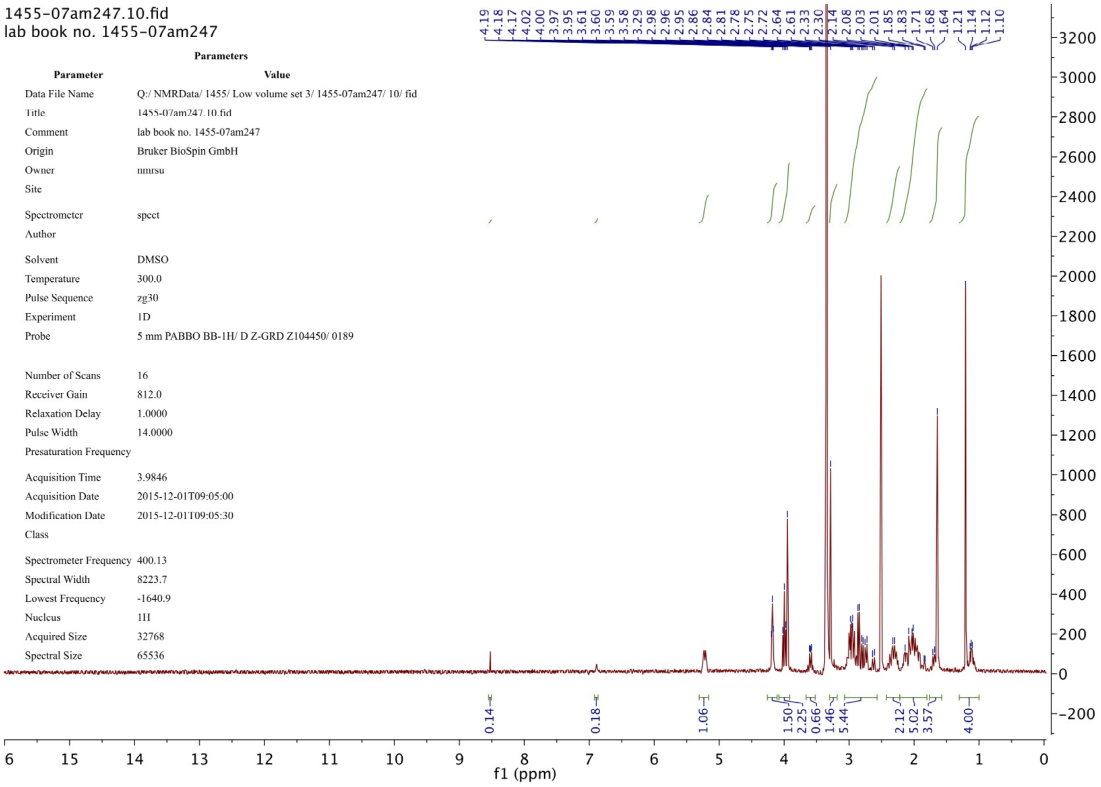

Supplementary Figure 134. *Proton NMR spectrum of* ***5v.***

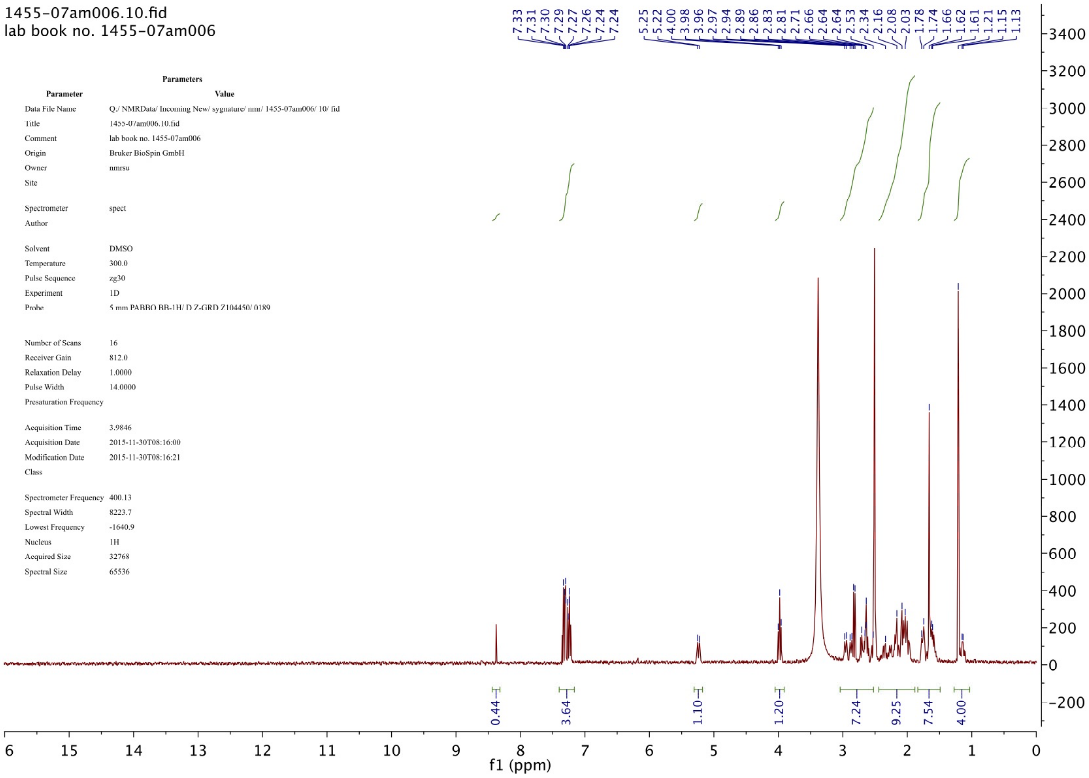

Supplementary Figure 135. *Proton NMR spectrum of* ***5w.***

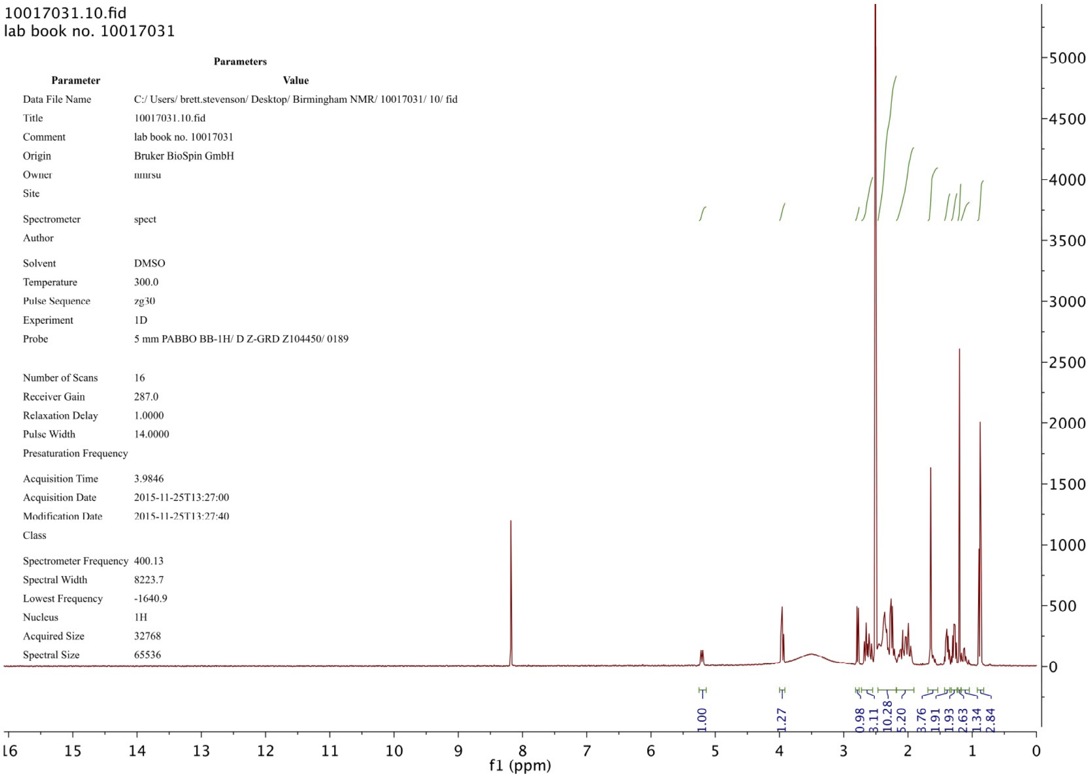

Supplementary Figure 136. *Proton NMR spectrum of* ***5x.***

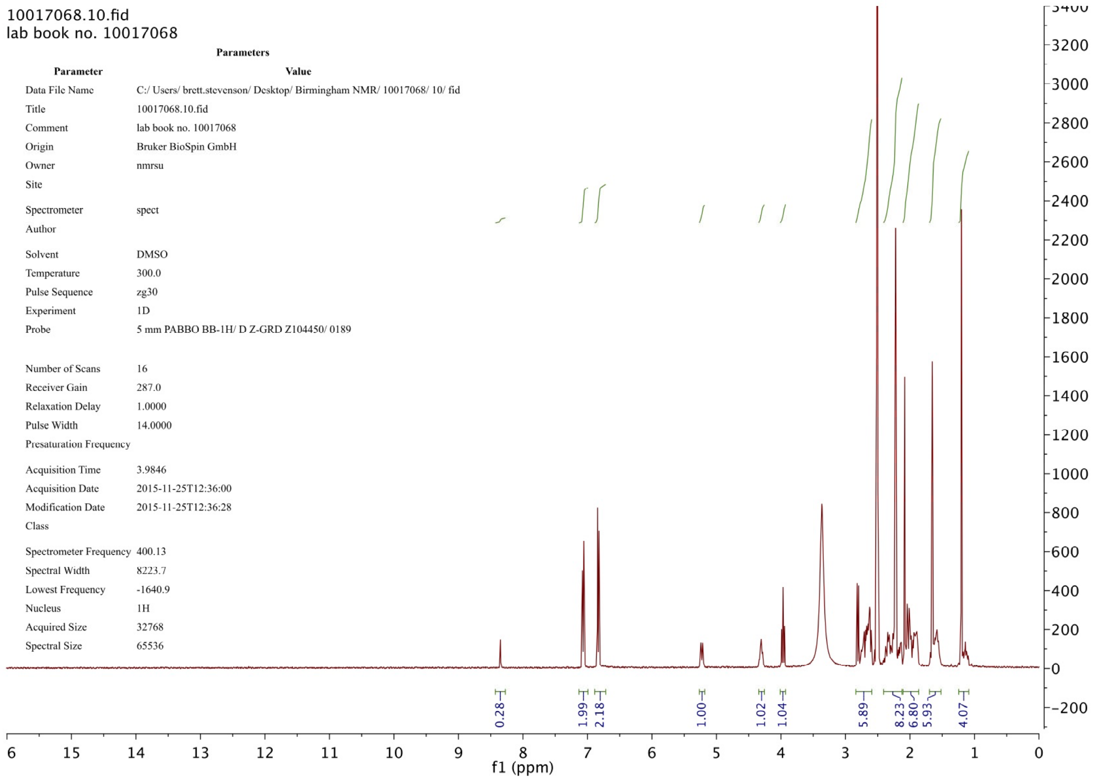

Supplementary Figure 137. *Proton NMR spectrum of* ***5y.***

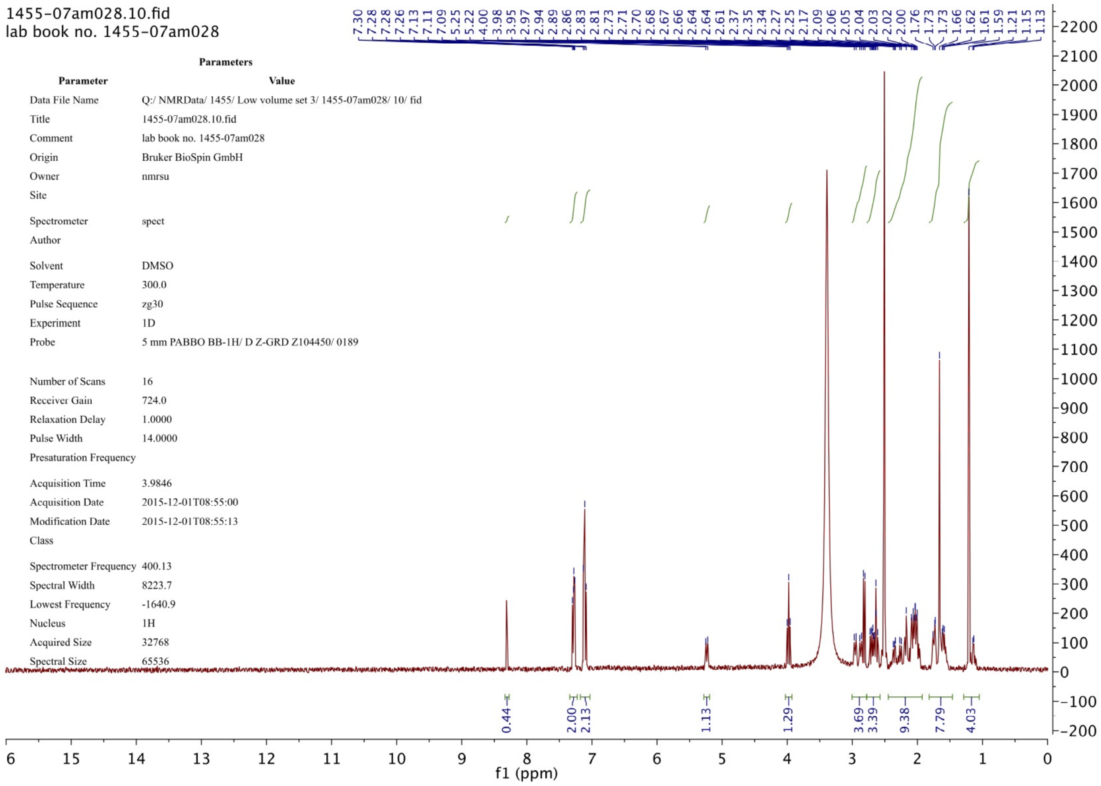

Supplementary Figure 138. *Proton NMR spectrum of* ***5z.***

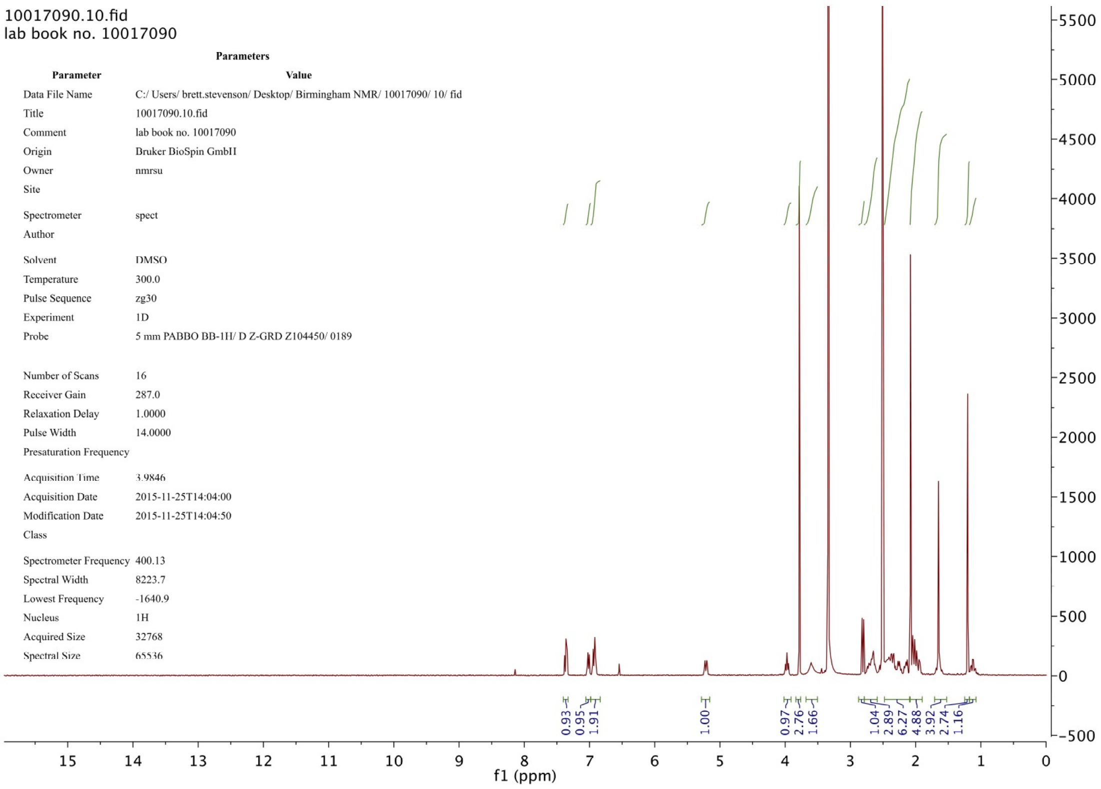

Supplementary Figure 139. *Proton NMR spectrum of* ***5aa.***

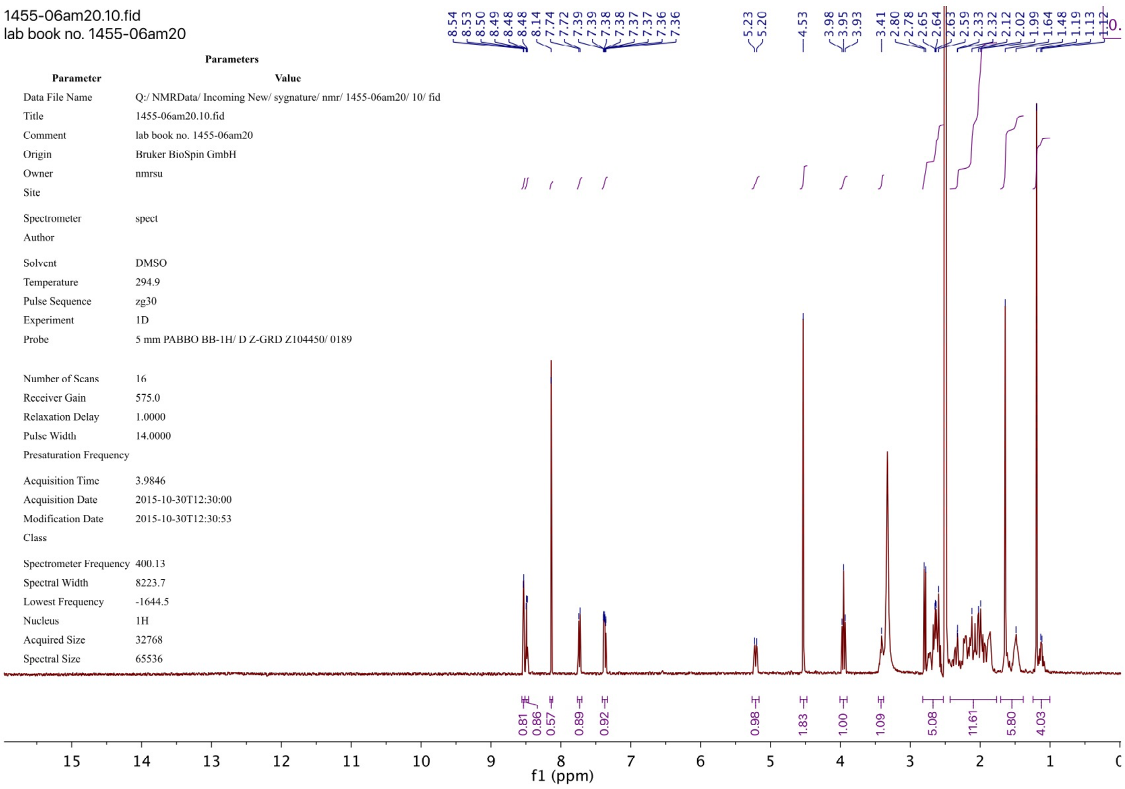

Supplementary Figure 140. *Proton NMR spectrum of* ***5ab.***

Mass spectrometry of lead compound 5f

Included as a representative example of the MS data reported in this manuscript, scanned versions of the MS and HRMS data obtained for **5f** are included herein. Scanned from A4 printouts obtained at the time of sample preparation. The scans of the black and white print outs have been cropped and brightness adjusted. Note the processing software for generating the HRMS report uses neutral formal as predicted mass, this ignores charge and mass of the electron.

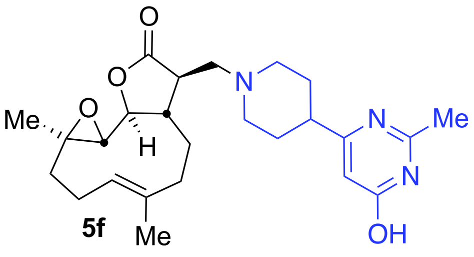

(**5f**+H) $C_{25}H_{36}N_3O_4$ Exact Mass: 442.27058

$(\mathbf{5f}+H)^+$ $C_{25}H_{36}N_3O_4^+$ Exact Mass: 442.27003

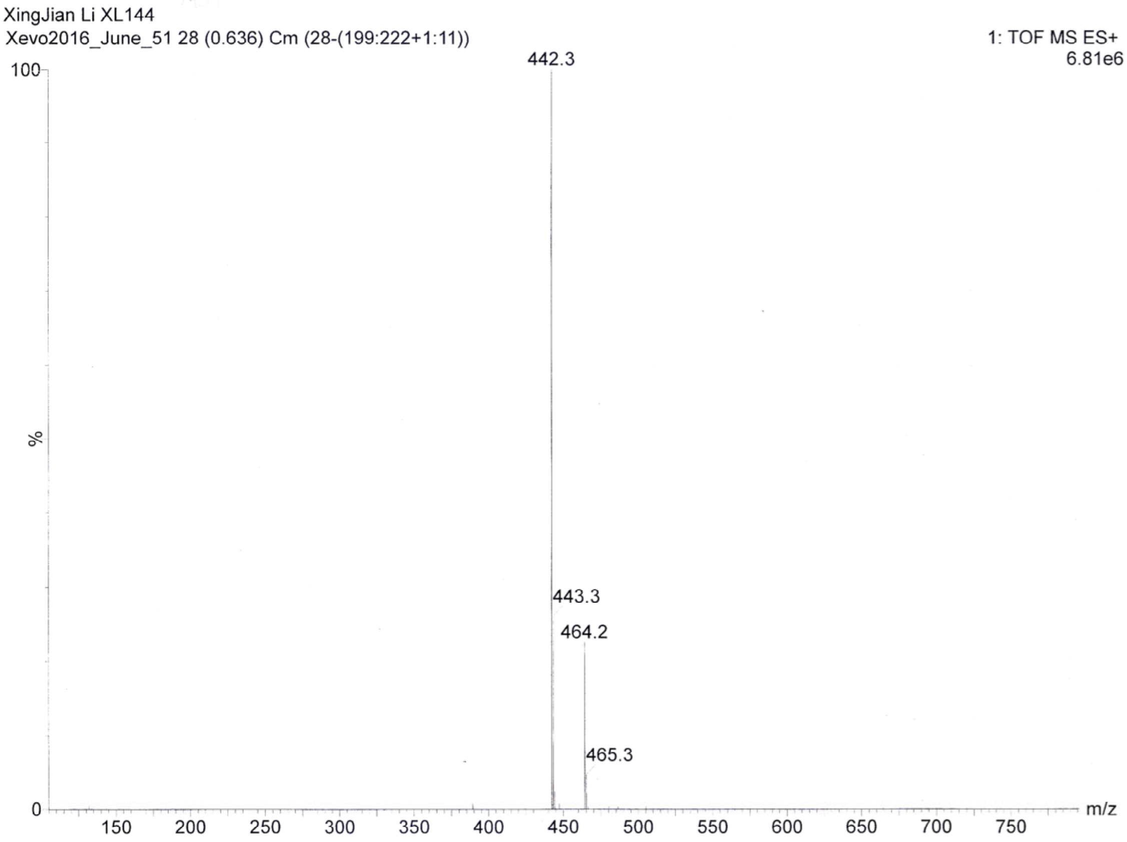

Supplementary Figure 141. *Mass spectrum obtained by electrospray ionisation, low resolution M+H⁺ expected m/z 442.3, observed m/z 442.3. Image scanned, that was cropped, and brightness/contrast adjusted in Microsoft Word.*

Elemental Composition Report **XingJian XL144**

Single Mass Analysis

Tolerance = 5.0 PPM / DBE: min = -1.5, max = 1000.0

Element prediction: Off

Monoisotopic Mass, Even Electron Ions

134 formula(e) evaluated with 1 results within limits (all results (up to 1000) for each mass)

Elements Used:

C: 0-100 H: 0-100 N: 1-5 O: 1-5

Minimum:			-1.5		
Maximum:	5.0	5.0	1000.0		
Mass	Calc. Mass	mDa	PPM	DBE	Formula
**442.2712**	442.2706	0.6	**1.4**	9.5	**C25 H36 N3 O4**

Supplementary Figure 142. *Scanned from print out of HRMS report, that was cropped, and brightness/contrast adjusted in Microsoft Word. Note the original report displays exact mass of the neutral species, whereas comparison should be made to the mass of a charged species, e.g m/z: $[M+H]^+$ calcd. for $C_{25}H_{36}N_3O_4^+$: 442.2700, found: 442.2712 (whilst within an acceptable 5 ppm tolerance the error is of the order of 2.7 ppm not the 1.4 ppm given in the original report, caution should be exercised).*

References

1. Royal Horticultural Society: *Tanacetum parthenium*, feverfew, https://www.rhs.org.uk/Plants/17986/i-Tanacetum-parthenium-i/Details, (accessed 19th December 2018).

2. The Plant List, http://www.theplantlist.org/tpl1.1/record/gcc-36373, (accessed 11th December 2018).

3. G. Bryant, *Plant Propagation A to Z: Growing Plants for Free*, David & Charles Ltd, 2004.

4. A. Agathanggelou, V. J. Weston, T. Perry, N. J. Davies, A. Skowronska, D. T. Payne, J. S. Fossey, C. E. Oldreive, W. Wei, G. Pratt, H. Parry, D. Oscier, S. J. Coles, P. S. Hole, R. L. Darley, M. McMahon, J. D. Hayes, P. Moss, G. S. Stewart, A. M. R. Taylor and T. Stankovic, *Haematologica*, 2015, **100**, 1076-1085.

5. W. Jamal, A. Bari, R. A. Mothana, O. Basudan, M. S. Mohammed and S. W. Ng, *Asian J. Chem.*, 2014, **26**, 5183-5185.

6. M. L. Guzman, R. M. Rossi, S. Neelakantan, X. Li, C. A. Corbett, D. C. Hassane, M. W. Becker, J. M. Bennett, E. Sullivan, J. L. Lachowicz, A. Vaughan, C. J. Sweeney, W. Matthews, M. Carroll, J. L. Liesveld, P. A. Crooks and C. T. Jordan, *Blood*, 2007, **110**, 4427-4435.

7. (a) S. Neelakantan, S. Nasim, M. L. Guzman, C. T. Jordan and P. A. Crooks, *Bioorg. Med. Chem. Lett.*, 2009, **19**, 4346-4349; (b) S. Nasim and P. A. Crooks, *Bioorg. Med. Chem. Lett.*, 2008, **18**, 3870-3873.

8. (a) R. Michelle, S. Marcus Tullius, C. Mauro Vicentini, F. Harold Hilarion, S. Luciana, J. Francisco Jaime Bezerra Mendonca, S. Marcelo Sobral da and E. Vicente de Paulo, *Lett. Drug Des. Discov.*, 2012, **9**, 881-890; (b) D.-R. Hwang, Y.-S. Wu, C.-W. Chang, T.-W. Lien, W.-C. Chen, U.-K. Tan, J. T. A. Hsu and H.-P. Hsieh, *Biorg. Med. Chem.*, 2006, **14**, 83-91; (c) H.-P. Hsieh, T.-A. Hsu and D.-R. Hwang, *US 2004/0229936 A1*, 2004.

9. P. A. Crooks, C. T. Jordan and X. Wei, *US 2005/0272716 A1*, 2005.

10. (a) J. R. Woods, H. Mo, A. A. Bieberich, T. Alavanja and D. A. Colby, *J. Med. Chem.*, 2011, **54**, 7934-7941; (b) P. A. Crooks, C. T. Jordan and X. Wei, *WO 2005/007103 A2*, 2005.

11. S. Zhang and G. Xiong, *CN 101270102A*, 2008.

12. (a) V. D. Nimbarte, H. Murtuza, S. Phaniraj, S. Shrivastava, V. G. M. Naidu, N. Satheesh Kumar and K. R. Atcha, *Med. Chem. Res.*, 2014, **23**, 2178-2197; (b) C. Qu, M. Ding, Y. Zhu, Y. Lu, J. Du, M. Miller, J. Tian, J. Zhu, J. Xu, M. Wen, A. G. A. Er-Bu, J. Wang, Y. Xiao, M. Wu, O. B. McManus, M. Li, J. Wu, H.-R. Luo, Z. Cao, B. Shen, H. Wang, M. X. Zhu and X. Hong, *J. Med. Chem.*, 2017, **60**, 4680-4692.

13. (a) A. Y. Zherebker, D. Airapetyan, A. I. Konstantinov, Y. I. Kostyukevich, A. S. Kononikhin, I. A. Popov, K. V. Zaitsev, E. N. Nikolaev and I. V. Perminova, *Analyst*, 2015, **140**, 4708-4719; (b) G. L. Bolton, *e-EROS Encyclopedia of Reagents for Organic Synthesis*, 2001.

14. (a) L. Huck, M. Berton, A. de la Hoz, A. Díaz-Ortiz and J. Alcázar, *Green Chem.*, 2017, **19**, 1420-1424; (b) F. Wang, J. Li, A. L. Sinn, W. E. Knabe, M. Khanna, I. Jo, J. M. Silver, K. Oh, L. Li, G. E. Sandusky, G. W. Sledge, H. Nakshatri, D. R. Jones, K. E. Pollok and S. O. Meroueh, *J. Med. Chem.*, 2011, **54**, 7193-7205; (c) K. E. Bashford, M. B. Burton, S. Cameron, A. L. Cooper, R. D. Hogg, P. D. Kane, D. A. MacManus, C. A. Matrunola, C. J. Moody, A. A. B. Robertson and M. R. Warne, *Tetrahedron Lett.*, 2003, **44**, 1627-1629.

15. D. N. Kazulkin and V. S. Kochubey, *EP2719696A1*, 2011.

16. M. Vidal, M. García-Arriagada, M. C. Rezende and M. Domínguez, *Synthesis*, 2016, **48**, 4246-4252.

17. Calculated molecular properties determined via the Collaborative Drug Discovery Vault (CDD Vault) web-registry-package within a trial access period. www.collaborativedrug.com

18. P. D. Leeson and A. M. Davis, *J. Med. Chem.*, 2004, **47**, 6338-6348.

19. (a) F. W. Goldberg, J. G. Kettle, T. Kogej, M. W. D. Perry and N. P. Tomkinson, *Drug Discov. Today*, 2015, **20**, 11-17; (b) N. C. Firth, N. Brown and J. Blagg, *J. Chem. Inf. Model.*, 2012, **52**, 2516-2525; (c) J. Meyers, M. Carter, N. Y. Mok and N. Brown, *Future Med. Chem.*, 2016, **8**, 1753-1767.

20. A. J. Garcia-Pineres, V. Castro, G. Mora, T. J. Schmidt, E. Strunck, H. L. Pahl and I. Merfort, *J. Biol. Chem.*, 2001, **276**, 39713-39720.

21. Y.-Q. Chen, S. Ghosh and G. Ghosh, *Nat. Struct. Biol.*, 1998, **5**, 67.

22. O. Trott and A. J. Olson, *J. Comput. Chem.*, 2010, **31**, 455-461.

23. G. M. Morris, R. Huey, W. Lindstrom, M. F. Sanner, R. K. Belew, D. S. Goodsell and A. J. Olson, *J. Comput. Chem.*, 2009, **30**, 2785-2791.

24. R. Dennington, T. A. Keith and J. M. Millam, *Journal*, 2016, **GaussView 6**.

25. N. M. O'Boyle, M. Banck, C. A. James, C. Morley, T. Vandermeersch and G. R. Hutchison, *J. Cheminform.*, 2011, **3**, 33.

26. V. Janganati, J. Ponder, M. Balasubramaniam, P. Bhat-Nakshatri, E. E. Bar, H. Nakshatri, C. T. Jordan and P. A. Crooks, *Eur. J. Med. Chem.*, 2018, **157**, 562-581.

27. G. Xu, Y.-C. Lo, Q. Li, G. Napolitano, X. Wu, X. Jiang, M. Dreano, M. Karin and H. Wu, *Nature*, 2011, **472**, 325.
